# Supplementary material for: MicroRNA mediated regulation in early-onset cardiac hypertrophy: Insights from the hypertrophic heart rat model
Source: PLoS One. 2025 Dec 30;20(12):e0338909. doi: 10.1371/journal.pone.0338909 (PMC12752997; doi:10.1371/journal.pone.0338909)
Supplement: S1 File — S1 Tables. MicroRNA and Genes Microarray Results (HHR/NHR, 2-days). S2 Tables. Target genes and pathway enrichment analysis. S1 Fig. Number of KEGG Pathways potentially regulated by miRNAs under investigation either individually or communally. S2 Fig. Number of GO:BP potentially regulated by microRNAs under investigation either individually or communally. S3 Fig. Comparative analysis of predicted target genes regulated by miR-34a, miR-351, and miR-490*. S4 Fig. Raw Ct values from RT-PCR of h9C2-1 transfected with mimics and inhibitors. (ZIP) [file pone.0338909.s001.zip › Supplementary Data_Major Revision/S4_Fig.docx]

## S3 Fig: Raw Ct values from RT-PCR of h9C2-1 transfected with mimics and inhibitors
