# Supplementary material for: MicroRNA mediated regulation in early-onset cardiac hypertrophy: Insights from the hypertrophic heart rat model
Source: PLoS One. 2025 Dec 30;20(12):e0338909. doi: 10.1371/journal.pone.0338909 (PMC12752997; doi:10.1371/journal.pone.0338909)
Supplement: S1 File — S1 Tables. MicroRNA and Genes Microarray Results (HHR/NHR, 2-days). S2 Tables. Target genes and pathway enrichment analysis. S1 Fig. Number of KEGG Pathways potentially regulated by miRNAs under investigation either individually or communally. S2 Fig. Number of GO:BP potentially regulated by microRNAs under investigation either individually or communally. S3 Fig. Comparative analysis of predicted target genes regulated by miR-34a, miR-351, and miR-490*. S4 Fig. Raw Ct values from RT-PCR of h9C2-1 transfected with mimics and inhibitors. (ZIP) [file pone.0338909.s001.zip › Supplementary Data_Major Revision/S2_Tables.docx]

## S2 Tables: Target genes and pathway enrichment analysis.

**S2A Table: Verified/Predict target genes of miR-34a**

| Source | Status | Gene Abbv |
| --- | --- | --- |
| Ingenuity Expert Findings | Experimentally Observed | CREB1 |
| miRecords | Experimentally Observed | MYC |
| Ingenuity Expert Findings | Experimentally Observed | TP53 |
| Ingenuity Expert Findings | Experimentally Observed | TRPS1 |
| miRecords | Experimentally Observed | VEGFA |
| Ingenuity Expert Findings,TargetScan Human,miRecords | Experimentally Observed,High (predicted) | BCL2 |
| Ingenuity Expert Findings,TarBase,TargetScan Human,miRecords | Experimentally Observed,High (predicted) | CCND1 |
| Ingenuity Expert Findings,TarBase,TargetScan Human,miRecords | Experimentally Observed,High (predicted) | CDK6 |
| TarBase,TargetScan Human,miRecords | Experimentally Observed,High (predicted) | DLL1 |
| Ingenuity Expert Findings,TarBase,TargetScan Human,miRecords | Experimentally Observed,High (predicted) | E2F3 |
| TargetScan Human,miRecords | Experimentally Observed,High (predicted) | E2F5 |
| Ingenuity Expert Findings,TargetScan Human | Experimentally Observed,High (predicted) | FOXP1 |
| Ingenuity Expert Findings,TargetScan Human,miRecords | Experimentally Observed,High (predicted) | JAG1 |
| TargetScan Human,miRecords | Experimentally Observed,High (predicted) | MAP2K1 |
| Ingenuity Expert Findings,TargetScan Human,miRecords | Experimentally Observed,High (predicted) | MET |
| TargetScan Human,miRecords | Experimentally Observed,High (predicted) | MYCN |
| Ingenuity Expert Findings,TarBase,TargetScan Human,miRecords | Experimentally Observed,High (predicted) | NOTCH1 |
| TargetScan Human,miRecords | Experimentally Observed,High (predicted) | NOTCH2 |
| Ingenuity Expert Findings,TargetScan Human,miRecords | Experimentally Observed,High (predicted) | WNT1 |
| TargetScan Human,miRecords | Experimentally Observed,Moderate (predicted) | AXIN2 |
| Ingenuity Expert Findings,TargetScan Human | Experimentally Observed,Moderate (predicted) | CD47 |
| Ingenuity Expert Findings,TargetScan Human | Experimentally Observed,Moderate (predicted) | HDAC1 |
| TargetScan Human,miRecords | Experimentally Observed,Moderate (predicted) | MYB |
| Ingenuity Expert Findings,TargetScan Human,miRecords | Experimentally Observed,Moderate (predicted) | SIRT1 |
| TargetScan Human,miRecords | Experimentally Observed,Moderate (predicted) | TAGLN |
| TargetScan Human,miRecords | Experimentally Observed,Moderate (predicted) | WISP2 |
| TargetScan Human | High (predicted) | AAGAB |
| TargetScan Human | High (predicted) | ABCD1 |
| TargetScan Human | High (predicted) | ABLIM1 |
| TargetScan Human | High (predicted) | ABR |
| TargetScan Human | High (predicted) | ACBD3 |
| TargetScan Human | High (predicted) | ACSL1 |
| TargetScan Human | High (predicted) | ACSL4 |
| TargetScan Human | High (predicted) | ACTL10 |
| TargetScan Human | High (predicted) | ACTR1A |
| TargetScan Human | High (predicted) | ACVR2B |
| TargetScan Human | High (predicted) | ADAM22 |
| TargetScan Human | High (predicted) | ADAMTS10 |
| TargetScan Human | High (predicted) | ADAMTSL4 |
| TargetScan Human | High (predicted) | ADAT2 |
| TargetScan Human | High (predicted) | ADCY5 |
| TargetScan Human | High (predicted) | ADD2 |
| TargetScan Human | High (predicted) | ADGRL1 |
| TargetScan Human | High (predicted) | ADIPOR2 |
| TargetScan Human | High (predicted) | ADO |
| TargetScan Human | High (predicted) | AFF2 |
| TargetScan Human | High (predicted) | AFF3 |
| TargetScan Human | High (predicted) | AFF4 |
| TargetScan Human | High (predicted) | AGAP2 |
| TargetScan Human | High (predicted) | AGO4 |
| TargetScan Human | High (predicted) | AGTRAP |
| TargetScan Human | High (predicted) | AHCYL2 |
| TargetScan Human | High (predicted) | AK3 |
| TargetScan Human | High (predicted) | AK4 |
| TargetScan Human | High (predicted) | AKAP6 |
| TargetScan Human | High (predicted) | AKIP1 |
| TargetScan Human | High (predicted) | ALCAM |
| TargetScan Human | High (predicted) | ALDOA |
| TargetScan Human | High (predicted) | ALG13 |
| TargetScan Human | High (predicted) | ALG6 |
| TargetScan Human | High (predicted) | ALS2CL |
| TargetScan Human | High (predicted) | AMER1 |
| TargetScan Human | High (predicted) | AMPD2 |
| TargetScan Human | High (predicted) | ANHX |
| TargetScan Human | High (predicted) | ANK2 |
| TargetScan Human | High (predicted) | ANK3 |
| TargetScan Human | High (predicted) | ANKRD52 |
| TargetScan Human | High (predicted) | ANKS1A |
| TargetScan Human | High (predicted) | ANP32A |
| TargetScan Human | High (predicted) | AP1B1 |
| TargetScan Human | High (predicted) | AP1S2 |
| TargetScan Human | High (predicted) | APH1A |
| TargetScan Human | High (predicted) | AREG |
| TargetScan Human | High (predicted) | ARHGAP1 |
| TargetScan Human | High (predicted) | ARHGAP26 |
| TargetScan Human | High (predicted) | ARHGAP36 |
| TargetScan Human | High (predicted) | ARHGAP44 |
| TargetScan Human | High (predicted) | ARHGDIB |
| TargetScan Human | High (predicted) | ARHGEF3 |
| TargetScan Human | High (predicted) | ARHGEF33 |
| TargetScan Human | High (predicted) | ARID4A |
| TargetScan Human | High (predicted) | ARID4B |
| TargetScan Human | High (predicted) | ARPP19 |
| TargetScan Human | High (predicted) | ASB1 |
| TargetScan Human | High (predicted) | ASB4 |
| TargetScan Human | High (predicted) | ASIC2 |
| TargetScan Human | High (predicted) | ASPHD2 |
| TargetScan Human | High (predicted) | ATF7 |
| TargetScan Human | High (predicted) | ATG4B |
| TargetScan Human | High (predicted) | ATG5 |
| TargetScan Human | High (predicted) | ATG9A |
| TargetScan Human | High (predicted) | ATMIN |
| TargetScan Human | High (predicted) | ATP1A2 |
| TargetScan Human | High (predicted) | ATXN7 |
| TargetScan Human | High (predicted) | ATXN7L3 |
| TargetScan Human | High (predicted) | ATXN7L3B |
| TargetScan Human | High (predicted) | AXL |
| TargetScan Human | High (predicted) | B3GALNT1 |
| TargetScan Human | High (predicted) | B4GALT2 |
| TargetScan Human | High (predicted) | BAZ2A |
| TargetScan Human | High (predicted) | BCL11B |
| TargetScan Human | High (predicted) | BCL9L |
| TargetScan Human | High (predicted) | BMP3 |
| TargetScan Human | High (predicted) | BMP8B |
| TargetScan Human | High (predicted) | BNC2 |
| TargetScan Human | High (predicted) | BRINP1 |
| TargetScan Human | High (predicted) | BRPF3 |
| TargetScan Human | High (predicted) | BTBD11 |
| TargetScan Human | High (predicted) | BTBD18 |
| TargetScan Human | High (predicted) | C11orf95 |
| TargetScan Human | High (predicted) | C14orf28 |
| TargetScan Human | High (predicted) | C15orf53 |
| TargetScan Human | High (predicted) | C16orf58 |
| TargetScan Human | High (predicted) | C1orf21 |
| TargetScan Human | High (predicted) | C2CD4A |
| TargetScan Human | High (predicted) | C3orf58 |
| TargetScan Human | High (predicted) | C3orf62 |
| TargetScan Human | High (predicted) | C3orf70 |
| TargetScan Human | High (predicted) | C7orf49 |
| TargetScan Human | High (predicted) | C8orf37 |
| TargetScan Human | High (predicted) | C9orf47 |
| TargetScan Human | High (predicted) | C9orf69 |
| TargetScan Human | High (predicted) | CA7 |
| TargetScan Human | High (predicted) | CACNA1E |
| TargetScan Human | High (predicted) | CACNA2D2 |
| TargetScan Human | High (predicted) | CACNB1 |
| TargetScan Human | High (predicted) | CACNB3 |
| TargetScan Human | High (predicted) | CACNG2 |
| TargetScan Human | High (predicted) | CADM4 |
| TargetScan Human | High (predicted) | CALB1 |
| TargetScan Human | High (predicted) | CALCR |
| TargetScan Human | High (predicted) | CALN1 |
| TargetScan Human | High (predicted) | CAMSAP1 |
| TargetScan Human | High (predicted) | CAMTA1 |
| TargetScan Human | High (predicted) | CAPN5 |
| TargetScan Human | High (predicted) | CAPN6 |
| TargetScan Human | High (predicted) | CASP2 |
| TargetScan Human | High (predicted) | CBFA2T3 |
| TargetScan Human | High (predicted) | CCDC50 |
| TargetScan Human | High (predicted) | CCDC85A |
| TargetScan Human | High (predicted) | CCDC88A |
| TargetScan Human | High (predicted) | CCL22 |
| TargetScan Human | High (predicted) | CCNE2 |
| TargetScan Human | High (predicted) | CCNJL |
| TargetScan Human | High (predicted) | CDC25A |
| TargetScan Human | High (predicted) | CDIP1 |
| TargetScan Human | High (predicted) | CDKN1C |
| TargetScan Human | High (predicted) | CDS2 |
| TargetScan Human | High (predicted) | CELF3 |
| TargetScan Human | High (predicted) | CELF6 |
| TargetScan Human | High (predicted) | CEP19 |
| TargetScan Human | High (predicted) | CERS6 |
| TargetScan Human | High (predicted) | CHD1 |
| TargetScan Human | High (predicted) | CHM |
| TargetScan Human | High (predicted) | CHMP7 |
| TargetScan Human | High (predicted) | CHST12 |
| TargetScan Human | High (predicted) | CLDN18 |
| TargetScan Human | High (predicted) | CLOCK |
| TargetScan Human | High (predicted) | CNOT6L |
| TargetScan Human | High (predicted) | CNTN2 |
| TargetScan Human | High (predicted) | CNTNAP1 |
| TargetScan Human | High (predicted) | CNTNAP2 |
| TargetScan Human | High (predicted) | COL12A1 |
| TargetScan Human | High (predicted) | COL4A4 |
| TargetScan Human | High (predicted) | COMMD9 |
| TargetScan Human | High (predicted) | COPS7B |
| TargetScan Human | High (predicted) | COPZ1 |
| TargetScan Human | High (predicted) | CORO1C |
| TargetScan Human | High (predicted) | CPD |
| TargetScan Human | High (predicted) | CPEB2 |
| TargetScan Human | High (predicted) | CPLX2 |
| TargetScan Human | High (predicted) | CR2 |
| TargetScan Human | High (predicted) | CREB3L1 |
| TargetScan Human | High (predicted) | CREB5 |
| TargetScan Human | High (predicted) | CREBRF |
| TargetScan Human | High (predicted) | CRHR1 |
| TargetScan Human | High (predicted) | CRTC1 |
| TargetScan Human | High (predicted) | CRY2 |
| TargetScan Human | High (predicted) | CSF1R |
| TargetScan Human | High (predicted) | CSMD1 |
| TargetScan Human | High (predicted) | CSNK1G1 |
| TargetScan Human | High (predicted) | CTCFL |
| TargetScan Human | High (predicted) | CTDSP2 |
| TargetScan Human | High (predicted) | CTDSPL |
| TargetScan Human | High (predicted) | CTNND1 |
| TargetScan Human | High (predicted) | CTNND2 |
| TargetScan Human | High (predicted) | CTTNBP2NL |
| TargetScan Human | High (predicted) | CUX1 |
| TargetScan Human | High (predicted) | CYB561A3 |
| TargetScan Human | High (predicted) | CYCS |
| TargetScan Human | High (predicted) | CYTH4 |
| TargetScan Human | High (predicted) | DAAM1 |
| TargetScan Human | High (predicted) | DAB2IP |
| TargetScan Human | High (predicted) | DCAF11 |
| TargetScan Human | High (predicted) | DCAF7 |
| TargetScan Human | High (predicted) | DCP1A |
| TargetScan Human | High (predicted) | DCX |
| TargetScan Human | High (predicted) | DDX17 |
| TargetScan Human | High (predicted) | DGAT1 |
| TargetScan Human | High (predicted) | DGKI |
| TargetScan Human | High (predicted) | DGKZ |
| TargetScan Human | High (predicted) | DHRS13 |
| TargetScan Human | High (predicted) | DIXDC1 |
| TargetScan Human | High (predicted) | DMWD |
| TargetScan Human | High (predicted) | DNAJB1 |
| TargetScan Human | High (predicted) | DNAJB2 |
| TargetScan Human | High (predicted) | DNAJC16 |
| TargetScan Human | High (predicted) | DNAJC24 |
| TargetScan Human | High (predicted) | DNM1L |
| TargetScan Human | High (predicted) | DOCK3 |
| TargetScan Human | High (predicted) | DOK6 |
| TargetScan Human | High (predicted) | DPP3 |
| TargetScan Human | High (predicted) | DPP9-AS1 |
| TargetScan Human | High (predicted) | DPYD |
| TargetScan Human | High (predicted) | DPYSL4 |
| TargetScan Human | High (predicted) | DRC7 |
| TargetScan Human | High (predicted) | DSEL |
| TargetScan Human | High (predicted) | EEA1 |
| TargetScan Human | High (predicted) | EEF2K |
| TargetScan Human | High (predicted) | EFNB1 |
| TargetScan Human | High (predicted) | EHD4 |
| TargetScan Human | High (predicted) | ELL2 |
| TargetScan Human | High (predicted) | ELMOD1 |
| TargetScan Human | High (predicted) | ELMSAN1 |
| TargetScan Human | High (predicted) | EME1 |
| TargetScan Human | High (predicted) | EML5 |
| TargetScan Human | High (predicted) | ENAM |
| TargetScan Human | High (predicted) | EPN2 |
| TargetScan Human | High (predicted) | EPS15L1 |
| TargetScan Human | High (predicted) | ERC1 |
| TargetScan Human | High (predicted) | ERGIC1 |
| TargetScan Human | High (predicted) | ERLIN1 |
| TargetScan Human | High (predicted) | ERP44 |
| TargetScan Human | High (predicted) | ESRRA |
| TargetScan Human | High (predicted) | ESYT3 |
| TargetScan Human | High (predicted) | EVI5L |
| TargetScan Human | High (predicted) | F2RL2 |
| TargetScan Human | High (predicted) | F8 |
| TargetScan Human | High (predicted) | FAIM2 |
| TargetScan Human | High (predicted) | FAM107A |
| TargetScan Human | High (predicted) | FAM117B |
| TargetScan Human | High (predicted) | FAM126B |
| TargetScan Human | High (predicted) | FAM162B |
| TargetScan Human | High (predicted) | FAM167A |
| TargetScan Human | High (predicted) | FAM175B |
| TargetScan Human | High (predicted) | FAM208A |
| TargetScan Human | High (predicted) | FAM212B |
| TargetScan Human | High (predicted) | FAM46A |
| TargetScan Human | High (predicted) | FAM71F1 |
| TargetScan Human | High (predicted) | FAM73B |
| TargetScan Human | High (predicted) | FAM76A |
| TargetScan Human | High (predicted) | FAM83A |
| TargetScan Human | High (predicted) | FAT2 |
| TargetScan Human | High (predicted) | FAT3 |
| TargetScan Human | High (predicted) | FAT4 |
| TargetScan Human | High (predicted) | FBXO10 |
| TargetScan Human | High (predicted) | FBXO30 |
| TargetScan Human | High (predicted) | FBXO41 |
| TargetScan Human | High (predicted) | FGD6 |
| TargetScan Human | High (predicted) | FGF23 |
| TargetScan Human | High (predicted) | FKBP1B |
| TargetScan Human | High (predicted) | FLOT2 |
| TargetScan Human | High (predicted) | FNDC3B |
| TargetScan Human | High (predicted) | FNDC5 |
| TargetScan Human | High (predicted) | FNDC8 |
| TargetScan Human | High (predicted) | FOSB |
| TargetScan Human | High (predicted) | FOSL1 |
| TargetScan Human | High (predicted) | FOXG1 |
| TargetScan Human | High (predicted) | FOXJ2 |
| TargetScan Human | High (predicted) | FOXN2 |
| TargetScan Human | High (predicted) | FOXN3 |
| TargetScan Human | High (predicted) | FOXP2 |
| TargetScan Human | High (predicted) | FOXQ1 |
| TargetScan Human | High (predicted) | FRMD4A |
| TargetScan Human | High (predicted) | FUCA2 |
| TargetScan Human | High (predicted) | FUK |
| TargetScan Human | High (predicted) | FUT1 |
| TargetScan Human | High (predicted) | FUT8 |
| TargetScan Human | High (predicted) | FUT9 |
| TargetScan Human | High (predicted) | GAB1 |
| TargetScan Human | High (predicted) | GABBR2 |
| TargetScan Human | High (predicted) | GABRA3 |
| TargetScan Human | High (predicted) | GALNT7 |
| TargetScan Human | High (predicted) | GAS1 |
| TargetScan Human | High (predicted) | GAS8 |
| TargetScan Human | High (predicted) | GATA3 |
| TargetScan Human | High (predicted) | GATAD2B |
| TargetScan Human | High (predicted) | GBA3 |
| TargetScan Human | High (predicted) | GCH1 |
| TargetScan Human | High (predicted) | GDAP1L1 |
| TargetScan Human | High (predicted) | GFRA1 |
| TargetScan Human | High (predicted) | GIGYF1 |
| TargetScan Human | High (predicted) | GINS3 |
| TargetScan Human | High (predicted) | GK5 |
| TargetScan Human | High (predicted) | GLCE |
| TargetScan Human | High (predicted) | GLRA3 |
| TargetScan Human | High (predicted) | GLRX5 |
| TargetScan Human | High (predicted) | GMFB |
| TargetScan Human | High (predicted) | GMNC |
| TargetScan Human | High (predicted) | GNAI2 |
| TargetScan Human | High (predicted) | GNAO1 |
| TargetScan Human | High (predicted) | GOLPH3L |
| TargetScan Human | High (predicted) | GP5 |
| TargetScan Human | High (predicted) | GPR12 |
| TargetScan Human | High (predicted) | GPR158 |
| TargetScan Human | High (predicted) | GPR22 |
| TargetScan Human | High (predicted) | GPR85 |
| TargetScan Human | High (predicted) | GPX8 |
| TargetScan Human | High (predicted) | GREM2 |
| TargetScan Human | High (predicted) | GRHL2 |
| TargetScan Human | High (predicted) | GRID1 |
| TargetScan Human | High (predicted) | GRM7 |
| TargetScan Human | High (predicted) | GRSF1 |
| TargetScan Human | High (predicted) | GSDMB |
| TargetScan Human | High (predicted) | GSG1 |
| TargetScan Human | High (predicted) | HACE1 |
| TargetScan Human | High (predicted) | HCN3 |
| TargetScan Human | High (predicted) | HECW2 |
| TargetScan Human | High (predicted) | HK1 |
| TargetScan Human | High (predicted) | HNF4A |
| TargetScan Human | High (predicted) | HNF4G |
| TargetScan Human | High (predicted) | HNRNPUL2 |
| TargetScan Human | High (predicted) | HOOK3 |
| TargetScan Human | High (predicted) | HOXA13 |
| TargetScan Human | High (predicted) | HS2ST1 |
| TargetScan Human | High (predicted) | HSBP1 |
| TargetScan Human | High (predicted) | HSPA1A/HSPA1B |
| TargetScan Human | High (predicted) | HTR2A |
| TargetScan Human | High (predicted) | HTR2C |
| TargetScan Human | High (predicted) | HYAL3 |
| TargetScan Human | High (predicted) | IGFBP3 |
| TargetScan Human | High (predicted) | IGSF1 |
| TargetScan Human | High (predicted) | IKBKE |
| TargetScan Human | High (predicted) | IL6R |
| TargetScan Human | High (predicted) | INA |
| TargetScan Human | High (predicted) | INF2 |
| TargetScan Human | High (predicted) | INHBB |
| TargetScan Human | High (predicted) | IRF1 |
| TargetScan Human | High (predicted) | IRF2BP2 |
| TargetScan Human | High (predicted) | IRGQ |
| TargetScan Human | High (predicted) | ISY1 |
| TargetScan Human | High (predicted) | ITGA10 |
| TargetScan Human | High (predicted) | ITGB8 |
| TargetScan Human | High (predicted) | ITSN1 |
| TargetScan Human | High (predicted) | JADE2 |
| TargetScan Human | High (predicted) | JAKMIP1 |
| TargetScan Human | High (predicted) | JMJD1C |
| TargetScan Human | High (predicted) | JPH3 |
| TargetScan Human | High (predicted) | KCNH7 |
| TargetScan Human | High (predicted) | KCNK3 |
| TargetScan Human | High (predicted) | KCNN3 |
| TargetScan Human | High (predicted) | KCNQ3 |
| TargetScan Human | High (predicted) | KDM4C |
| TargetScan Human | High (predicted) | KDM5D |
| TargetScan Human | High (predicted) | KDM7A |
| TargetScan Human | High (predicted) | KIAA0753 |
| TargetScan Human | High (predicted) | KIAA1024 |
| TargetScan Human | High (predicted) | KIAA1210 |
| TargetScan Human | High (predicted) | KIAA1217 |
| TargetScan Human | High (predicted) | KIAA1462 |
| TargetScan Human | High (predicted) | KIF17 |
| TargetScan Human | High (predicted) | KIT |
| TargetScan Human | High (predicted) | KITLG |
| TargetScan Human | High (predicted) | KLC2 |
| TargetScan Human | High (predicted) | KLF4 |
| TargetScan Human | High (predicted) | KLF6 |
| TargetScan Human | High (predicted) | KLHDC3 |
| TargetScan Human | High (predicted) | KLHL25 |
| TargetScan Human | High (predicted) | KLRC4-KLRK1/KLRK1 |
| TargetScan Human | High (predicted) | KLRD1 |
| TargetScan Human | High (predicted) | KMT2D |
| TargetScan Human | High (predicted) | KMT5B |
| TargetScan Human | High (predicted) | KRI1 |
| TargetScan Human | High (predicted) | LDHA |
| TargetScan Human | High (predicted) | LEF1 |
| TargetScan Human | High (predicted) | LGI1 |
| TargetScan Human | High (predicted) | LGR4 |
| TargetScan Human | High (predicted) | LHCGR |
| TargetScan Human | High (predicted) | LHPP |
| TargetScan Human | High (predicted) | LIMD2 |
| TargetScan Human | High (predicted) | LIN28A |
| TargetScan Human | High (predicted) | LMAN1 |
| TargetScan Human | High (predicted) | LMAN2L |
| TargetScan Human | High (predicted) | LMBR1L |
| TargetScan Human | High (predicted) | LMNB2 |
| TargetScan Human | High (predicted) | LMTK3 |
| TargetScan Human | High (predicted) | LPAR2 |
| TargetScan Human | High (predicted) | LPO |
| TargetScan Human | High (predicted) | LRCH1 |
| TargetScan Human | High (predicted) | LRPAP1 |
| TargetScan Human | High (predicted) | LRRC40 |
| TargetScan Human | High (predicted) | LRRC55 |
| TargetScan Human | High (predicted) | LRRC56 |
| TargetScan Human | High (predicted) | LRRC7 |
| TargetScan Human | High (predicted) | LRRFIP1 |
| TargetScan Human | High (predicted) | LRRTM2 |
| TargetScan Human | High (predicted) | LYPLAL1 |
| TargetScan Human | High (predicted) | LYST |
| TargetScan Human | High (predicted) | MAOA |
| TargetScan Human | High (predicted) | MAP1A |
| TargetScan Human | High (predicted) | MAP4K4 |
| TargetScan Human | High (predicted) | MAP7D3 |
| TargetScan Human | High (predicted) | MAPK13 |
| TargetScan Human | High (predicted) | MAPT |
| TargetScan Human | High (predicted) | MARCH5 |
| TargetScan Human | High (predicted) | MARCH8 |
| TargetScan Human | High (predicted) | MARCKSL1 |
| TargetScan Human | High (predicted) | MAZ |
| TargetScan Human | High (predicted) | MBD6 |
| TargetScan Human | High (predicted) | MBLAC1 |
| TargetScan Human | High (predicted) | MBP |
| TargetScan Human | High (predicted) | MCFD2 |
| TargetScan Human | High (predicted) | MCIDAS |
| TargetScan Human | High (predicted) | MDM4 |
| TargetScan Human | High (predicted) | MED8 |
| TargetScan Human | High (predicted) | MEX3C |
| TargetScan Human | High (predicted) | MFSD12 |
| TargetScan Human | High (predicted) | MGAT4A |
| TargetScan Human | High (predicted) | MGAT5B |
| TargetScan Human | High (predicted) | MLEC |
| TargetScan Human | High (predicted) | MLLT1 |
| TargetScan Human | High (predicted) | MLLT3 |
| TargetScan Human | High (predicted) | MMAB |
| TargetScan Human | High (predicted) | MOAP1 |
| TargetScan Human | High (predicted) | MON2 |
| TargetScan Human | High (predicted) | MPP2 |
| TargetScan Human | High (predicted) | MPPED2 |
| TargetScan Human | High (predicted) | MRPL10 |
| TargetScan Human | High (predicted) | MRPL17 |
| TargetScan Human | High (predicted) | MRPL52 |
| TargetScan Human | High (predicted) | MSL2 |
| TargetScan Human | High (predicted) | MTA2 |
| TargetScan Human | High (predicted) | MTF1 |
| TargetScan Human | High (predicted) | MTMR9 |
| TargetScan Human | High (predicted) | MTUS1 |
| TargetScan Human | High (predicted) | MYADM |
| TargetScan Human | High (predicted) | MYH9 |
| TargetScan Human | High (predicted) | MYRIP |
| TargetScan Human | High (predicted) | NAA50 |
| TargetScan Human | High (predicted) | NAP1L5 |
| TargetScan Human | High (predicted) | NAPEPLD |
| TargetScan Human | High (predicted) | NAT8L |
| TargetScan Human | High (predicted) | NAV1 |
| TargetScan Human | High (predicted) | NAV2 |
| TargetScan Human | High (predicted) | NAV3 |
| TargetScan Human | High (predicted) | NCDN |
| TargetScan Human | High (predicted) | NCEH1 |
| TargetScan Human | High (predicted) | NCOA1 |
| TargetScan Human | High (predicted) | NCOR2 |
| TargetScan Human | High (predicted) | NDST1 |
| TargetScan Human | High (predicted) | NDUFC2 |
| TargetScan Human | High (predicted) | NEDD4L |
| TargetScan Human | High (predicted) | NETO1 |
| TargetScan Human | High (predicted) | NEURL1B |
| TargetScan Human | High (predicted) | NEUROD2 |
| TargetScan Human | High (predicted) | NFATC4 |
| TargetScan Human | High (predicted) | NFE2L1 |
| TargetScan Human | High (predicted) | NKTR |
| TargetScan Human | High (predicted) | NMT2 |
| TargetScan Human | High (predicted) | NMUR1 |
| TargetScan Human | High (predicted) | NOP2 |
| TargetScan Human | High (predicted) | NOS1 |
| TargetScan Human | High (predicted) | NOS1AP |
| TargetScan Human | High (predicted) | NPTX1 |
| TargetScan Human | High (predicted) | NR2C2 |
| TargetScan Human | High (predicted) | NR4A2 |
| TargetScan Human | High (predicted) | NRIP3 |
| TargetScan Human | High (predicted) | NRN1 |
| TargetScan Human | High (predicted) | NRXN2 |
| TargetScan Human | High (predicted) | NSD1 |
| TargetScan Human | High (predicted) | NTN4 |
| TargetScan Human | High (predicted) | NUMBL |
| TargetScan Human | High (predicted) | OBFC1 |
| TargetScan Human | High (predicted) | OLFML1 |
| TargetScan Human | High (predicted) | OLIG3 |
| TargetScan Human | High (predicted) | ONECUT2 |
| TargetScan Human | High (predicted) | OR2H1 |
| TargetScan Human | High (predicted) | ORAI3 |
| TargetScan Human | High (predicted) | ORMDL3 |
| TargetScan Human | High (predicted) | OSGIN2 |
| TargetScan Human | High (predicted) | OTUD3 |
| TargetScan Human | High (predicted) | OVOL2 |
| TargetScan Human | High (predicted) | OXSR1 |
| TargetScan Human | High (predicted) | P2RY14 |
| TargetScan Human | High (predicted) | PACS1 |
| TargetScan Human | High (predicted) | PAG1 |
| TargetScan Human | High (predicted) | PALM2 |
| TargetScan Human | High (predicted) | PAPPA |
| TargetScan Human | High (predicted) | PAQR7 |
| TargetScan Human | High (predicted) | PARD6B |
| TargetScan Human | High (predicted) | PARP8 |
| TargetScan Human | High (predicted) | PCLO |
| TargetScan Human | High (predicted) | PCNX |
| TargetScan Human | High (predicted) | PCYOX1 |
| TargetScan Human | High (predicted) | PDCD4 |
| TargetScan Human | High (predicted) | PDE4B |
| TargetScan Human | High (predicted) | PDE7B |
| TargetScan Human | High (predicted) | PDGFRA |
| TargetScan Human | High (predicted) | PDGFRB |
| TargetScan Human | High (predicted) | PDK3 |
| TargetScan Human | High (predicted) | PDXK |
| TargetScan Human | High (predicted) | PEA15 |
| TargetScan Human | High (predicted) | PEAK1 |
| TargetScan Human | High (predicted) | PEG10 |
| TargetScan Human | High (predicted) | PFKM |
| TargetScan Human | High (predicted) | PGAP2 |
| TargetScan Human | High (predicted) | PGF |
| TargetScan Human | High (predicted) | PGM1 |
| TargetScan Human | High (predicted) | PGRMC2 |
| TargetScan Human | High (predicted) | PHF19 |
| TargetScan Human | High (predicted) | PHF24 |
| TargetScan Human | High (predicted) | PID1 |
| TargetScan Human | High (predicted) | PIGZ |
| TargetScan Human | High (predicted) | PIP5K1A |
| TargetScan Human | High (predicted) | PKIA |
| TargetScan Human | High (predicted) | PKNOX1 |
| TargetScan Human | High (predicted) | PKP4 |
| TargetScan Human | High (predicted) | PLA2G15 |
| TargetScan Human | High (predicted) | PLA2G2F |
| TargetScan Human | High (predicted) | PLAG1 |
| TargetScan Human | High (predicted) | PLCG1 |
| TargetScan Human | High (predicted) | PLEKHH2 |
| TargetScan Human | High (predicted) | PLET1 |
| TargetScan Human | High (predicted) | PLIN4 |
| TargetScan Human | High (predicted) | PLN |
| TargetScan Human | High (predicted) | PLOD1 |
| TargetScan Human | High (predicted) | PLPP5 |
| TargetScan Human | High (predicted) | PNOC |
| TargetScan Human | High (predicted) | PODXL |
| TargetScan Human | High (predicted) | POFUT1 |
| TargetScan Human | High (predicted) | POGZ |
| TargetScan Human | High (predicted) | POU2F1 |
| TargetScan Human | High (predicted) | POU6F1 |
| TargetScan Human | High (predicted) | PPARGC1B |
| TargetScan Human | High (predicted) | PPFIA1 |
| TargetScan Human | High (predicted) | PPM1A |
| TargetScan Human | High (predicted) | PPP1R10 |
| TargetScan Human | High (predicted) | PPP1R11 |
| TargetScan Human | High (predicted) | PPP1R16B |
| TargetScan Human | High (predicted) | PPP1R9B |
| TargetScan Human | High (predicted) | PPP2R3A |
| TargetScan Human | High (predicted) | PREB |
| TargetScan Human | High (predicted) | PRKCE |
| TargetScan Human | High (predicted) | PRKCQ |
| TargetScan Human | High (predicted) | PRKD1 |
| TargetScan Human | High (predicted) | PSD3 |
| TargetScan Human | High (predicted) | PTGIS |
| TargetScan Human | High (predicted) | PTPRD |
| TargetScan Human | High (predicted) | PTPRM |
| TargetScan Human | High (predicted) | PURB |
| TargetScan Human | High (predicted) | PVRL1 |
| TargetScan Human | High (predicted) | PXDC1 |
| TargetScan Human | High (predicted) | RAB11FIP4 |
| TargetScan Human | High (predicted) | RAB36 |
| TargetScan Human | High (predicted) | RAD9B |
| TargetScan Human | High (predicted) | RAE1 |
| TargetScan Human | High (predicted) | RALGDS |
| TargetScan Human | High (predicted) | RALGPS1 |
| TargetScan Human | High (predicted) | RALGPS2 |
| TargetScan Human | High (predicted) | RANBP10 |
| TargetScan Human | High (predicted) | RAP1GAP |
| TargetScan Human | High (predicted) | RAP1GDS1 |
| TargetScan Human | High (predicted) | RAPH1 |
| TargetScan Human | High (predicted) | RARB |
| TargetScan Human | High (predicted) | RASGEF1C |
| TargetScan Human | High (predicted) | RASGRP4 |
| TargetScan Human | High (predicted) | RASL12 |
| TargetScan Human | High (predicted) | RBFOX2 |
| TargetScan Human | High (predicted) | RCAN1 |
| TargetScan Human | High (predicted) | RECK |
| TargetScan Human | High (predicted) | RELN |
| TargetScan Human | High (predicted) | RELT |
| TargetScan Human | High (predicted) | RET |
| TargetScan Human | High (predicted) | RGMB |
| TargetScan Human | High (predicted) | RGS17 |
| TargetScan Human | High (predicted) | RHOV |
| TargetScan Human | High (predicted) | RIC8B |
| TargetScan Human | High (predicted) | RIMS3 |
| TargetScan Human | High (predicted) | RIMS4 |
| TargetScan Human | High (predicted) | RNF165 |
| TargetScan Human | High (predicted) | RNF169 |
| TargetScan Human | High (predicted) | RNF34 |
| TargetScan Human | High (predicted) | RNF41 |
| TargetScan Human | High (predicted) | RNF44 |
| TargetScan Human | High (predicted) | ROCK1 |
| TargetScan Human | High (predicted) | RORA |
| TargetScan Human | High (predicted) | RPS6KA4 |
| TargetScan Human | High (predicted) | RPS6KL1 |
| TargetScan Human | High (predicted) | RRAS |
| TargetScan Human | High (predicted) | RSPO4 |
| TargetScan Human | High (predicted) | RTF1 |
| TargetScan Human | High (predicted) | RTN4RL1 |
| TargetScan Human | High (predicted) | RUFY2 |
| TargetScan Human | High (predicted) | SAMD12 |
| TargetScan Human | High (predicted) | SAR1A |
| TargetScan Human | High (predicted) | SATB1 |
| TargetScan Human | High (predicted) | SATB2 |
| TargetScan Human | High (predicted) | SBK1 |
| TargetScan Human | High (predicted) | SCML2 |
| TargetScan Human | High (predicted) | SCN1A |
| TargetScan Human | High (predicted) | SCN2B |
| TargetScan Human | High (predicted) | SCNN1G |
| TargetScan Human | High (predicted) | SDHC |
| TargetScan Human | High (predicted) | SDK2 |
| TargetScan Human | High (predicted) | SDR9C7 |
| TargetScan Human | High (predicted) | SEMA4B |
| TargetScan Human | High (predicted) | SEMA4C |
| TargetScan Human | High (predicted) | SEMA4F |
| TargetScan Human | High (predicted) | SEPT3 |
| TargetScan Human | High (predicted) | SERPINE1 |
| TargetScan Human | High (predicted) | SERPINF2 |
| TargetScan Human | High (predicted) | SF3B3 |
| TargetScan Human | High (predicted) | SFMBT2 |
| TargetScan Human | High (predicted) | SGPP1 |
| TargetScan Human | High (predicted) | SGSM2 |
| TargetScan Human | High (predicted) | SGTA |
| TargetScan Human | High (predicted) | SHE |
| TargetScan Human | High (predicted) | SHISA7 |
| TargetScan Human | High (predicted) | SHKBP1 |
| TargetScan Human | High (predicted) | SIDT1 |
| TargetScan Human | High (predicted) | SIDT2 |
| TargetScan Human | High (predicted) | SIPA1 |
| TargetScan Human | High (predicted) | SIRPB1 |
| TargetScan Human | High (predicted) | SIRT6 |
| TargetScan Human | High (predicted) | SIX3 |
| TargetScan Human | High (predicted) | SKI |
| TargetScan Human | High (predicted) | SLC12A2 |
| TargetScan Human | High (predicted) | SLC16A2 |
| TargetScan Human | High (predicted) | SLC25A27 |
| TargetScan Human | High (predicted) | SLC27A4 |
| TargetScan Human | High (predicted) | SLC2A13 |
| TargetScan Human | High (predicted) | SLC2A4RG |
| TargetScan Human | High (predicted) | SLC30A3 |
| TargetScan Human | High (predicted) | SLC31A2 |
| TargetScan Human | High (predicted) | SLC44A2 |
| TargetScan Human | High (predicted) | SLC4A7 |
| TargetScan Human | High (predicted) | SLC5A3 |
| TargetScan Human | High (predicted) | SLC6A1 |
| TargetScan Human | High (predicted) | SLC6A17 |
| TargetScan Human | High (predicted) | SLC7A2 |
| TargetScan Human | High (predicted) | SLC7A6 |
| TargetScan Human | High (predicted) | SLCO3A1 |
| TargetScan Human | High (predicted) | SMAD4 |
| TargetScan Human | High (predicted) | SMCO1 |
| TargetScan Human | High (predicted) | SMIM15 |
| TargetScan Human | High (predicted) | SMIM24 |
| TargetScan Human | High (predicted) | SNAI1 |
| TargetScan Human | High (predicted) | SNTB2 |
| TargetScan Human | High (predicted) | SNX15 |
| TargetScan Human | High (predicted) | SNX30 |
| TargetScan Human | High (predicted) | SNX4 |
| TargetScan Human | High (predicted) | SOCS4 |
| TargetScan Human | High (predicted) | SOGA1 |
| TargetScan Human | High (predicted) | SOX4 |
| TargetScan Human | High (predicted) | SP2 |
| TargetScan Human | High (predicted) | SPCS2 |
| TargetScan Human | High (predicted) | SPEG |
| TargetScan Human | High (predicted) | SPICE1 |
| TargetScan Human | High (predicted) | SPRED1 |
| TargetScan Human | High (predicted) | SPRN |
| TargetScan Human | High (predicted) | SPRY3 |
| TargetScan Human | High (predicted) | SRC |
| TargetScan Human | High (predicted) | SRCIN1 |
| TargetScan Human | High (predicted) | SRPRA |
| TargetScan Human | High (predicted) | SSX5 |
| TargetScan Human | High (predicted) | ST8SIA3 |
| TargetScan Human | High (predicted) | STAB2 |
| TargetScan Human | High (predicted) | STAC2 |
| TargetScan Human | High (predicted) | STK38L |
| TargetScan Human | High (predicted) | STRN3 |
| TargetScan Human | High (predicted) | STX17 |
| TargetScan Human | High (predicted) | STX1A |
| TargetScan Human | High (predicted) | SURF4 |
| TargetScan Human | High (predicted) | SVIP |
| TargetScan Human | High (predicted) | SVOP |
| TargetScan Human | High (predicted) | SYN2 |
| TargetScan Human | High (predicted) | SYNJ1 |
| TargetScan Human | High (predicted) | SYT1 |
| TargetScan Human | High (predicted) | SYT9 |
| TargetScan Human | High (predicted) | SYVN1 |
| TargetScan Human | High (predicted) | SZRD1 |
| TargetScan Human | High (predicted) | TAF4B |
| TargetScan Human | High (predicted) | TAF5 |
| TargetScan Human | High (predicted) | TANC2 |
| TargetScan Human | High (predicted) | TBC1D13 |
| TargetScan Human | High (predicted) | TBC1D25 |
| TargetScan Human | High (predicted) | TBC1D30 |
| TargetScan Human | High (predicted) | TBCK |
| TargetScan Human | High (predicted) | TBL1XR1 |
| TargetScan Human | High (predicted) | TCF12 |
| TargetScan Human | High (predicted) | TDRD6 |
| TargetScan Human | High (predicted) | TFCP2L1 |
| TargetScan Human | High (predicted) | TFDP2 |
| TargetScan Human | High (predicted) | TGIF2 |
| TargetScan Human | High (predicted) | THSD4 |
| TargetScan Human | High (predicted) | THUMPD3 |
| TargetScan Human | High (predicted) | TM7SF3 |
| TargetScan Human | High (predicted) | TM9SF3 |
| TargetScan Human | High (predicted) | TMCC3 |
| TargetScan Human | High (predicted) | TMEM104 |
| TargetScan Human | High (predicted) | TMEM109 |
| TargetScan Human | High (predicted) | TMEM130 |
| TargetScan Human | High (predicted) | TMEM164 |
| TargetScan Human | High (predicted) | TMEM167B |
| TargetScan Human | High (predicted) | TMEM184B |
| TargetScan Human | High (predicted) | TMEM200B |
| TargetScan Human | High (predicted) | TMEM201 |
| TargetScan Human | High (predicted) | TMEM246 |
| TargetScan Human | High (predicted) | TMEM255A |
| TargetScan Human | High (predicted) | TMEM33 |
| TargetScan Human | High (predicted) | TMEM35 |
| TargetScan Human | High (predicted) | TMEM52B |
| TargetScan Human | High (predicted) | TMEM55A |
| TargetScan Human | High (predicted) | TMEM97 |
| TargetScan Human | High (predicted) | TMOD2 |
| TargetScan Human | High (predicted) | TMTC3 |
| TargetScan Human | High (predicted) | TNKS |
| TargetScan Human | High (predicted) | TNP1 |
| TargetScan Human | High (predicted) | TNRC18 |
| TargetScan Human | High (predicted) | TNRC6B |
| TargetScan Human | High (predicted) | TOB2 |
| TargetScan Human | High (predicted) | TOM1 |
| TargetScan Human | High (predicted) | TOX |
| TargetScan Human | High (predicted) | TP53INP2 |
| TargetScan Human | High (predicted) | TP73 |
| TargetScan Human | High (predicted) | TPCN2 |
| TargetScan Human | High (predicted) | TPD52 |
| TargetScan Human | High (predicted) | TPD52L3 |
| TargetScan Human | High (predicted) | TRANK1 |
| TargetScan Human | High (predicted) | TRIM41 |
| TargetScan Human | High (predicted) | TRIM67 |
| TargetScan Human | High (predicted) | TRIM9 |
| TargetScan Human | High (predicted) | TSEN15 |
| TargetScan Human | High (predicted) | TSHZ2 |
| TargetScan Human | High (predicted) | TSN |
| TargetScan Human | High (predicted) | TSPAN14 |
| TargetScan Human | High (predicted) | TSPAN18 |
| TargetScan Human | High (predicted) | TSSC4 |
| TargetScan Human | High (predicted) | TTC19 |
| TargetScan Human | High (predicted) | TUFT1 |
| TargetScan Human | High (predicted) | TUSC5 |
| TargetScan Human | High (predicted) | TWIST2 |
| TargetScan Human | High (predicted) | UBE2G1 |
| TargetScan Human | High (predicted) | UBE2QL1 |
| TargetScan Human | High (predicted) | UBN2 |
| TargetScan Human | High (predicted) | UBP1 |
| TargetScan Human | High (predicted) | UBR1 |
| TargetScan Human | High (predicted) | UBXN10 |
| TargetScan Human | High (predicted) | UCK2 |
| TargetScan Human | High (predicted) | UCN2 |
| TargetScan Human | High (predicted) | UCP3 |
| TargetScan Human | High (predicted) | UHRF2 |
| TargetScan Human | High (predicted) | UNG |
| TargetScan Human | High (predicted) | USF1 |
| TargetScan Human | High (predicted) | USP24 |
| TargetScan Human | High (predicted) | USP31 |
| TargetScan Human | High (predicted) | VAMP2 |
| TargetScan Human | High (predicted) | VAT1 |
| TargetScan Human | High (predicted) | VCL |
| TargetScan Human | High (predicted) | VPS37A |
| TargetScan Human | High (predicted) | VPS37B |
| TargetScan Human | High (predicted) | VPS37D |
| TargetScan Human | High (predicted) | VPS4A |
| TargetScan Human | High (predicted) | VTCN1 |
| TargetScan Human | High (predicted) | VTI1A |
| TargetScan Human | High (predicted) | VWA5B2 |
| TargetScan Human | High (predicted) | WASF1 |
| TargetScan Human | High (predicted) | WDR37 |
| TargetScan Human | High (predicted) | WHSC1 |
| TargetScan Human | High (predicted) | WSCD2 |
| TargetScan Human | High (predicted) | WTAP |
| TargetScan Human | High (predicted) | XIRP1 |
| TargetScan Human | High (predicted) | XPO5 |
| TargetScan Human | High (predicted) | XYLT1 |
| TargetScan Human | High (predicted) | YTHDC1 |
| TargetScan Human | High (predicted) | YY1 |
| TargetScan Human | High (predicted) | ZBTB46 |
| TargetScan Human | High (predicted) | ZBTB9 |
| TargetScan Human | High (predicted) | ZC3H4 |
| TargetScan Human | High (predicted) | ZC3H7B |
| TargetScan Human | High (predicted) | ZCCHC17 |
| TargetScan Human | High (predicted) | ZDHHC16 |
| TargetScan Human | High (predicted) | ZDHHC17 |
| TargetScan Human | High (predicted) | ZDHHC23 |
| TargetScan Human | High (predicted) | ZER1 |
| TargetScan Human | High (predicted) | ZFHX4 |
| TargetScan Human | High (predicted) | ZFP41 |
| TargetScan Human | High (predicted) | ZHX2 |
| TargetScan Human | High (predicted) | ZIC5 |
| TargetScan Human | High (predicted) | ZMYM4 |
| TargetScan Human | High (predicted) | ZNF16 |
| TargetScan Human | High (predicted) | ZNF275 |
| TargetScan Human | High (predicted) | ZNF281 |
| TargetScan Human | High (predicted) | ZNF282 |
| TargetScan Human | High (predicted) | ZNF285 |
| TargetScan Human | High (predicted) | ZNF287 |
| TargetScan Human | High (predicted) | ZNF304 |
| TargetScan Human | High (predicted) | ZNF551 |
| TargetScan Human | High (predicted) | ZNF641 |
| TargetScan Human | High (predicted) | ZNF644 |
| TargetScan Human | High (predicted) | ZNF831 |
| TargetScan Human | High (predicted) | ZNF844 |
| TargetScan Human | High (predicted) | ZSCAN22 |
| TargetScan Human | High (predicted) | ZSCAN29 |
| TargetScan Human | High (predicted) | ZSCAN9 |
| TargetScan Human | High (predicted) | ZSWIM5 |
| TargetScan Human | High (predicted) | ZYG11B |
| TargetScan Human | High (predicted),Moderate (predicted) | DSC3 |
| TargetScan Human | Moderate (predicted) | ABCC12 |
| TargetScan Human | Moderate (predicted) | ABCG4 |
| TargetScan Human | Moderate (predicted) | ABHD12 |
| TargetScan Human | Moderate (predicted) | ABLIM3 |
| TargetScan Human | Moderate (predicted) | ABRACL |
| TargetScan Human | Moderate (predicted) | ACE |
| TargetScan Human | Moderate (predicted) | ACKR1 |
| TargetScan Human | Moderate (predicted) | ACKR4 |
| TargetScan Human | Moderate (predicted) | ACTR8 |
| TargetScan Human | Moderate (predicted) | ADAM11 |
| TargetScan Human | Moderate (predicted) | ADAM12 |
| TargetScan Human | Moderate (predicted) | ADGRE2 |
| TargetScan Human | Moderate (predicted) | ADGRF2 |
| TargetScan Human | Moderate (predicted) | ADK |
| TargetScan Human | Moderate (predicted) | ADPRH |
| TargetScan Human | Moderate (predicted) | ADRA1D |
| TargetScan Human | Moderate (predicted) | AGTR1 |
| TargetScan Human | Moderate (predicted) | AIF1L |
| TargetScan Human | Moderate (predicted) | AK9 |
| TargetScan Human | Moderate (predicted) | AKTIP |
| TargetScan Human | Moderate (predicted) | AMOTL1 |
| TargetScan Human | Moderate (predicted) | AMOTL2 |
| TargetScan Human | Moderate (predicted) | AMZ1 |
| TargetScan Human | Moderate (predicted) | ANGEL1 |
| TargetScan Human | Moderate (predicted) | ANKRD23 |
| TargetScan Human | Moderate (predicted) | ANP32B |
| TargetScan Human | Moderate (predicted) | AP3S2 |
| TargetScan Human | Moderate (predicted) | APLNR |
| TargetScan Human | Moderate (predicted) | ARHGEF9 |
| TargetScan Human | Moderate (predicted) | ARMC5 |
| TargetScan Human | Moderate (predicted) | ARPIN/C15orf38-AP3S2 |
| TargetScan Human | Moderate (predicted) | ARSB |
| TargetScan Human | Moderate (predicted) | ASXL2 |
| TargetScan Human | Moderate (predicted) | ATG13 |
| TargetScan Human | Moderate (predicted) | ATG2B |
| TargetScan Human | Moderate (predicted) | ATP13A3 |
| TargetScan Human | Moderate (predicted) | ATP5SL |
| TargetScan Human | Moderate (predicted) | ATP6V1E1 |
| TargetScan Human | Moderate (predicted) | ATPAF1 |
| TargetScan Human | Moderate (predicted) | ATPIF1 |
| TargetScan Human | Moderate (predicted) | ATXN1L |
| TargetScan Human | Moderate (predicted) | BBS1 |
| TargetScan Human | Moderate (predicted) | BCAN |
| TargetScan Human | Moderate (predicted) | BCCIP |
| TargetScan Human | Moderate (predicted) | BCL6 |
| TargetScan Human | Moderate (predicted) | BCO2 |
| TargetScan Human | Moderate (predicted) | BDKRB2 |
| TargetScan Human | Moderate (predicted) | BEST1 |
| TargetScan Human | Moderate (predicted) | BET1 |
| TargetScan Human | Moderate (predicted) | BMF |
| TargetScan Human | Moderate (predicted) | BORCS8 |
| TargetScan Human | Moderate (predicted) | BRAT1 |
| TargetScan Human | Moderate (predicted) | BSN |
| TargetScan Human | Moderate (predicted) | BSPRY |
| TargetScan Human | Moderate (predicted) | C10orf131 |
| TargetScan Human | Moderate (predicted) | C10orf88 |
| TargetScan Human | Moderate (predicted) | C12orf29 |
| TargetScan Human | Moderate (predicted) | C12orf57 |
| TargetScan Human | Moderate (predicted) | C15orf54 |
| TargetScan Human | Moderate (predicted) | C1orf95 |
| TargetScan Human | Moderate (predicted) | C2CD2 |
| TargetScan Human | Moderate (predicted) | C2orf44 |
| TargetScan Human | Moderate (predicted) | C2orf61 |
| TargetScan Human | Moderate (predicted) | C2orf71 |
| TargetScan Human | Moderate (predicted) | C3orf17 |
| TargetScan Human | Moderate (predicted) | C5orf64 |
| TargetScan Human | Moderate (predicted) | C6orf222 |
| TargetScan Human | Moderate (predicted) | CA9 |
| TargetScan Human | Moderate (predicted) | CABLES1 |
| TargetScan Human | Moderate (predicted) | CACNG4 |
| TargetScan Human | Moderate (predicted) | CALCA |
| TargetScan Human | Moderate (predicted) | CALCB |
| TargetScan Human | Moderate (predicted) | CALCOCO2 |
| TargetScan Human | Moderate (predicted) | CALHM1 |
| TargetScan Human | Moderate (predicted) | CALU |
| TargetScan Human | Moderate (predicted) | CAMLG |
| TargetScan Human | Moderate (predicted) | CAP1 |
| TargetScan Human | Moderate (predicted) | CAPN14 |
| TargetScan Human | Moderate (predicted) | CAPN9 |
| TargetScan Human | Moderate (predicted) | CAV3 |
| TargetScan Human | Moderate (predicted) | CBX3 |
| TargetScan Human | Moderate (predicted) | CCR1 |
| TargetScan Human | Moderate (predicted) | CD99L2 |
| TargetScan Human | Moderate (predicted) | CDA |
| TargetScan Human | Moderate (predicted) | CDAN1 |
| TargetScan Human | Moderate (predicted) | CDC23 |
| TargetScan Human | Moderate (predicted) | CDC42SE2 |
| TargetScan Human | Moderate (predicted) | CDH13 |
| TargetScan Human | Moderate (predicted) | CDH9 |
| TargetScan Human | Moderate (predicted) | CDKL2 |
| TargetScan Human | Moderate (predicted) | CDRT1 |
| TargetScan Human | Moderate (predicted) | CELF2 |
| TargetScan Human | Moderate (predicted) | CERS5 |
| TargetScan Human | Moderate (predicted) | CES4A |
| TargetScan Human | Moderate (predicted) | CFTR |
| TargetScan Human | Moderate (predicted) | CHCHD7 |
| TargetScan Human | Moderate (predicted) | CHD3 |
| TargetScan Human | Moderate (predicted) | CHMP3 |
| TargetScan Human | Moderate (predicted) | CHRAC1 |
| TargetScan Human | Moderate (predicted) | CHST10 |
| TargetScan Human | Moderate (predicted) | CHST13 |
| TargetScan Human | Moderate (predicted) | CHST3 |
| TargetScan Human | Moderate (predicted) | CIAO1 |
| TargetScan Human | Moderate (predicted) | CLDN22 |
| TargetScan Human | Moderate (predicted) | CLIC5 |
| TargetScan Human | Moderate (predicted) | CLIP3 |
| TargetScan Human | Moderate (predicted) | CNOT4 |
| TargetScan Human | Moderate (predicted) | CNST |
| TargetScan Human | Moderate (predicted) | CNTN3 |
| TargetScan Human | Moderate (predicted) | CNTNAP4 |
| TargetScan Human | Moderate (predicted) | COG3 |
| TargetScan Human | Moderate (predicted) | COG6 |
| TargetScan Human | Moderate (predicted) | COL25A1 |
| TargetScan Human | Moderate (predicted) | COL26A1 |
| TargetScan Human | Moderate (predicted) | COL5A2 |
| TargetScan Human | Moderate (predicted) | CPA4 |
| TargetScan Human | Moderate (predicted) | CPEB3 |
| TargetScan Human | Moderate (predicted) | CRB3 |
| TargetScan Human | Moderate (predicted) | CRNKL1 |
| TargetScan Human | Moderate (predicted) | CSDC2 |
| TargetScan Human | Moderate (predicted) | CSRNP3 |
| TargetScan Human | Moderate (predicted) | CWC25 |
| TargetScan Human | Moderate (predicted) | CXCL10 |
| TargetScan Human | Moderate (predicted) | CXCL11 |
| TargetScan Human | Moderate (predicted) | CXCL16 |
| TargetScan Human | Moderate (predicted) | CXCR1 |
| TargetScan Human | Moderate (predicted) | CYB5RL |
| TargetScan Human | Moderate (predicted) | CYBRD1 |
| TargetScan Human | Moderate (predicted) | CYP4F8 |
| TargetScan Human | Moderate (predicted) | DAG1 |
| TargetScan Human | Moderate (predicted) | DAPK2 |
| TargetScan Human | Moderate (predicted) | DBNDD1 |
| TargetScan Human | Moderate (predicted) | DBNL |
| TargetScan Human | Moderate (predicted) | DDN |
| TargetScan Human | Moderate (predicted) | DDX10 |
| TargetScan Human | Moderate (predicted) | DGCR14 |
| TargetScan Human | Moderate (predicted) | DGKB |
| TargetScan Human | Moderate (predicted) | DIEXF |
| TargetScan Human | Moderate (predicted) | DISC1 |
| TargetScan Human | Moderate (predicted) | DKK1 |
| TargetScan Human | Moderate (predicted) | DLG3 |
| TargetScan Human | Moderate (predicted) | DLX1 |
| TargetScan Human | Moderate (predicted) | DMRTC1/DMRTC1B |
| TargetScan Human | Moderate (predicted) | DOCK8 |
| TargetScan Human | Moderate (predicted) | DOCK9 |
| TargetScan Human | Moderate (predicted) | DPH6 |
| TargetScan Human | Moderate (predicted) | DRD2 |
| TargetScan Human | Moderate (predicted) | DUSP4 |
| TargetScan Human | Moderate (predicted) | DUSP8 |
| TargetScan Human | Moderate (predicted) | DYNC1LI1 |
| TargetScan Human | Moderate (predicted) | ECE1 |
| TargetScan Human | Moderate (predicted) | EDAR |
| TargetScan Human | Moderate (predicted) | EFNB3 |
| TargetScan Human | Moderate (predicted) | EGFLAM |
| TargetScan Human | Moderate (predicted) | EI24 |
| TargetScan Human | Moderate (predicted) | EIF2S2 |
| TargetScan Human | Moderate (predicted) | ELMO1 |
| TargetScan Human | Moderate (predicted) | EPHA10 |
| TargetScan Human | Moderate (predicted) | EPHA4 |
| TargetScan Human | Moderate (predicted) | EPHB2 |
| TargetScan Human | Moderate (predicted) | FAM120AOS |
| TargetScan Human | Moderate (predicted) | FAM124A |
| TargetScan Human | Moderate (predicted) | FAM127C |
| TargetScan Human | Moderate (predicted) | FAM131B |
| TargetScan Human | Moderate (predicted) | FAM207A |
| TargetScan Human | Moderate (predicted) | FAM234A |
| TargetScan Human | Moderate (predicted) | FARP2 |
| TargetScan Human | Moderate (predicted) | FASTKD2 |
| TargetScan Human | Moderate (predicted) | FBXO16 |
| TargetScan Human | Moderate (predicted) | FBXW2 |
| TargetScan Human | Moderate (predicted) | FBXW4 |
| TargetScan Human | Moderate (predicted) | FCHO2 |
| TargetScan Human | Moderate (predicted) | FES |
| TargetScan Human | Moderate (predicted) | FETUB |
| TargetScan Human | Moderate (predicted) | FGD3 |
| TargetScan Human | Moderate (predicted) | FGFRL1 |
| TargetScan Human | Moderate (predicted) | FKBP11 |
| TargetScan Human | Moderate (predicted) | FKBP1A |
| TargetScan Human | Moderate (predicted) | FKBP9 |
| TargetScan Human | Moderate (predicted) | FLJ45513 |
| TargetScan Human | Moderate (predicted) | FLVCR2 |
| TargetScan Human | Moderate (predicted) | FMNL3 |
| TargetScan Human | Moderate (predicted) | FMO5 |
| TargetScan Human | Moderate (predicted) | FOXI1 |
| TargetScan Human | Moderate (predicted) | FOXP3 |
| TargetScan Human | Moderate (predicted) | FOXR2 |
| TargetScan Human | Moderate (predicted) | FRA10AC1 |
| TargetScan Human | Moderate (predicted) | FRK |
| TargetScan Human | Moderate (predicted) | FUT5 |
| TargetScan Human | Moderate (predicted) | FXYD2 |
| TargetScan Human | Moderate (predicted) | FXYD6-FXYD2 |
| TargetScan Human | Moderate (predicted) | FZR1 |
| TargetScan Human | Moderate (predicted) | GABRG3 |
| TargetScan Human | Moderate (predicted) | GALM |
| TargetScan Human | Moderate (predicted) | GALNT10 |
| TargetScan Human | Moderate (predicted) | GATC |
| TargetScan Human | Moderate (predicted) | GBP5 |
| TargetScan Human | Moderate (predicted) | GCNT2 |
| TargetScan Human | Moderate (predicted) | GEMIN5 |
| TargetScan Human | Moderate (predicted) | GFAP |
| TargetScan Human | Moderate (predicted) | GJD3 |
| TargetScan Human | Moderate (predicted) | GLP1R |
| TargetScan Human | Moderate (predicted) | GLTP |
| TargetScan Human | Moderate (predicted) | GLYATL3 |
| TargetScan Human | Moderate (predicted) | GMIP |
| TargetScan Human | Moderate (predicted) | GNPDA1 |
| TargetScan Human | Moderate (predicted) | GOLGB1 |
| TargetScan Human | Moderate (predicted) | GPD2 |
| TargetScan Human | Moderate (predicted) | GPHB5 |
| TargetScan Human | Moderate (predicted) | GPIHBP1 |
| TargetScan Human | Moderate (predicted) | GPR107 |
| TargetScan Human | Moderate (predicted) | GPR143 |
| TargetScan Human | Moderate (predicted) | GPR156 |
| TargetScan Human | Moderate (predicted) | GPR55 |
| TargetScan Human | Moderate (predicted) | GPX3 |
| TargetScan Human | Moderate (predicted) | GRAPL |
| TargetScan Human | Moderate (predicted) | GRID2IP |
| TargetScan Human | Moderate (predicted) | GRK6 |
| TargetScan Human | Moderate (predicted) | GTF2F1 |
| TargetScan Human | Moderate (predicted) | GUCY1A3 |
| TargetScan Human | Moderate (predicted) | GYPE |
| TargetScan Human | Moderate (predicted) | HBS1L |
| TargetScan Human | Moderate (predicted) | HERC6 |
| TargetScan Human | Moderate (predicted) | HEYL |
| TargetScan Human | Moderate (predicted) | HGF |
| TargetScan Human | Moderate (predicted) | HGH1 |
| TargetScan Human | Moderate (predicted) | HID1 |
| TargetScan Human | Moderate (predicted) | HIP1 |
| TargetScan Human | Moderate (predicted) | HLA-DQA1 |
| TargetScan Human | Moderate (predicted) | HMGCS1 |
| TargetScan Human | Moderate (predicted) | HMGN4 |
| TargetScan Human | Moderate (predicted) | HMMR |
| TargetScan Human | Moderate (predicted) | HNF1A |
| TargetScan Human | Moderate (predicted) | HNRNPUL1 |
| TargetScan Human | Moderate (predicted) | HPSE |
| TargetScan Human | Moderate (predicted) | HS1BP3 |
| TargetScan Human | Moderate (predicted) | HSPB6 |
| TargetScan Human | Moderate (predicted) | HSPBP1 |
| TargetScan Human | Moderate (predicted) | ICOSLG/LOC102723996 |
| TargetScan Human | Moderate (predicted) | IDH1 |
| TargetScan Human | Moderate (predicted) | IER5L |
| TargetScan Human | Moderate (predicted) | IFI35 |
| TargetScan Human | Moderate (predicted) | IGF2BP3 |
| TargetScan Human | Moderate (predicted) | IL10RB |
| TargetScan Human | Moderate (predicted) | IL1RN |
| TargetScan Human | Moderate (predicted) | IL21R |
| TargetScan Human | Moderate (predicted) | IL2RB |
| TargetScan Human | Moderate (predicted) | ILDR1 |
| TargetScan Human | Moderate (predicted) | ILDR2 |
| TargetScan Human | Moderate (predicted) | ING3 |
| TargetScan Human | Moderate (predicted) | INPP5B |
| TargetScan Human | Moderate (predicted) | INPP5D |
| TargetScan Human | Moderate (predicted) | INSM2 |
| TargetScan Human | Moderate (predicted) | IRAK2 |
| TargetScan Human | Moderate (predicted) | IRAK4 |
| TargetScan Human | Moderate (predicted) | IRF4 |
| TargetScan Human | Moderate (predicted) | IRS1 |
| TargetScan Human | Moderate (predicted) | ISY1-RAB43 |
| TargetScan Human | Moderate (predicted) | JAZF1 |
| TargetScan Human | Moderate (predicted) | JPH1 |
| TargetScan Human | Moderate (predicted) | JRK |
| TargetScan Human | Moderate (predicted) | KBTBD12 |
| TargetScan Human | Moderate (predicted) | KCNA2 |
| TargetScan Human | Moderate (predicted) | KCNC3 |
| TargetScan Human | Moderate (predicted) | KCNJ8 |
| TargetScan Human | Moderate (predicted) | KCNS2 |
| TargetScan Human | Moderate (predicted) | KCTD21 |
| TargetScan Human | Moderate (predicted) | KDELR3 |
| TargetScan Human | Moderate (predicted) | KDM8 |
| TargetScan Human | Moderate (predicted) | KIAA0825 |
| TargetScan Human | Moderate (predicted) | KIF13A |
| TargetScan Human | Moderate (predicted) | KLF17 |
| TargetScan Human | Moderate (predicted) | KLHDC7A |
| TargetScan Human | Moderate (predicted) | KLHDC8B |
| TargetScan Human | Moderate (predicted) | KLHL23/PHOSPHO2-KLHL23 |
| TargetScan Human | Moderate (predicted) | KLK13 |
| TargetScan Human | Moderate (predicted) | KRT5 |
| TargetScan Human | Moderate (predicted) | KRT74 |
| TargetScan Human | Moderate (predicted) | KRT82 |
| TargetScan Human | Moderate (predicted) | KRTAP10-7 |
| TargetScan Human | Moderate (predicted) | KSR1 |
| TargetScan Human | Moderate (predicted) | KSR2 |
| TargetScan Human | Moderate (predicted) | KY |
| TargetScan Human | Moderate (predicted) | LCLAT1 |
| TargetScan Human | Moderate (predicted) | LDAH |
| TargetScan Human | Moderate (predicted) | LDOC1L |
| TargetScan Human | Moderate (predicted) | LELP1 |
| TargetScan Human | Moderate (predicted) | LEPROTL1 |
| TargetScan Human | Moderate (predicted) | LETM1 |
| TargetScan Human | Moderate (predicted) | LGI3 |
| TargetScan Human | Moderate (predicted) | LIMA1 |
| TargetScan Human | Moderate (predicted) | LMNA |
| TargetScan Human | Moderate (predicted) | LOC100130705 |
| TargetScan Human | Moderate (predicted) | LPCAT1 |
| TargetScan Human | Moderate (predicted) | LRIG1 |
| TargetScan Human | Moderate (predicted) | LRP4 |
| TargetScan Human | Moderate (predicted) | LRRC46 |
| TargetScan Human | Moderate (predicted) | LSM14B |
| TargetScan Human | Moderate (predicted) | LTF |
| TargetScan Human | Moderate (predicted) | LUZP4 |
| TargetScan Human | Moderate (predicted) | LY6G5B |
| TargetScan Human | Moderate (predicted) | LYPD8 |
| TargetScan Human | Moderate (predicted) | LZTS2 |
| TargetScan Human | Moderate (predicted) | MAF1 |
| TargetScan Human | Moderate (predicted) | MAGEA12 |
| TargetScan Human | Moderate (predicted) | MAGEA2/MAGEA2B |
| TargetScan Human | Moderate (predicted) | MAGT1 |
| TargetScan Human | Moderate (predicted) | MAL |
| TargetScan Human | Moderate (predicted) | MAP3K14 |
| TargetScan Human | Moderate (predicted) | MAP3K15 |
| TargetScan Human | Moderate (predicted) | MAP6D1 |
| TargetScan Human | Moderate (predicted) | MAPRE2 |
| TargetScan Human | Moderate (predicted) | MARCKS |
| TargetScan Human | Moderate (predicted) | MARVELD3 |
| TargetScan Human | Moderate (predicted) | MAST3 |
| TargetScan Human | Moderate (predicted) | MC2R |
| TargetScan Human | Moderate (predicted) | MCMDC2 |
| TargetScan Human | Moderate (predicted) | MCTP1 |
| TargetScan Human | Moderate (predicted) | MDFIC |
| TargetScan Human | Moderate (predicted) | MECR |
| TargetScan Human | Moderate (predicted) | MED19 |
| TargetScan Human | Moderate (predicted) | MED27 |
| TargetScan Human | Moderate (predicted) | METAP1 |
| TargetScan Human | Moderate (predicted) | METTL1 |
| TargetScan Human | Moderate (predicted) | METTL4 |
| TargetScan Human | Moderate (predicted) | MFAP4 |
| TargetScan Human | Moderate (predicted) | MFN2 |
| TargetScan Human | Moderate (predicted) | MFSD8 |
| TargetScan Human | Moderate (predicted) | MID1 |
| TargetScan Human | Moderate (predicted) | MIER2 |
| TargetScan Human | Moderate (predicted) | MIER3 |
| TargetScan Human | Moderate (predicted) | MITF |
| TargetScan Human | Moderate (predicted) | MKX |
| TargetScan Human | Moderate (predicted) | MMP25 |
| TargetScan Human | Moderate (predicted) | MNT |
| TargetScan Human | Moderate (predicted) | MPV17L2 |
| TargetScan Human | Moderate (predicted) | MRPL30 |
| TargetScan Human | Moderate (predicted) | MRPL33 |
| TargetScan Human | Moderate (predicted) | MRPS14 |
| TargetScan Human | Moderate (predicted) | MRPS25 |
| TargetScan Human | Moderate (predicted) | MRTO4 |
| TargetScan Human | Moderate (predicted) | MRVI1 |
| TargetScan Human | Moderate (predicted) | MS4A14 |
| TargetScan Human | Moderate (predicted) | MS4A2 |
| TargetScan Human | Moderate (predicted) | MSANTD1 |
| TargetScan Human | Moderate (predicted) | MSANTD3-TMEFF1 |
| TargetScan Human | Moderate (predicted) | MSTN |
| TargetScan Human | Moderate (predicted) | MTMR10 |
| TargetScan Human | Moderate (predicted) | MTMR12 |
| TargetScan Human | Moderate (predicted) | MTURN |
| TargetScan Human | Moderate (predicted) | MUC2 |
| TargetScan Human | Moderate (predicted) | MXD4 |
| TargetScan Human | Moderate (predicted) | MYO15A |
| TargetScan Human | Moderate (predicted) | MYO1C |
| TargetScan Human | Moderate (predicted) | MYOCD |
| TargetScan Human | Moderate (predicted) | MYOZ3 |
| TargetScan Human | Moderate (predicted) | MYPN |
| TargetScan Human | Moderate (predicted) | MYRF |
| TargetScan Human | Moderate (predicted) | MYT1 |
| TargetScan Human | Moderate (predicted) | NAGPA |
| TargetScan Human | Moderate (predicted) | NAIP |
| TargetScan Human | Moderate (predicted) | NAP1L1 |
| TargetScan Human | Moderate (predicted) | NAT10 |
| TargetScan Human | Moderate (predicted) | NCBP2 |
| TargetScan Human | Moderate (predicted) | NDUFAF6 |
| TargetScan Human | Moderate (predicted) | NEDD4 |
| TargetScan Human | Moderate (predicted) | NEDD9 |
| TargetScan Human | Moderate (predicted) | NEK5 |
| TargetScan Human | Moderate (predicted) | NETO2 |
| TargetScan Human | Moderate (predicted) | NFAM1 |
| TargetScan Human | Moderate (predicted) | NFKBIA |
| TargetScan Human | Moderate (predicted) | NFKBIE |
| TargetScan Human | Moderate (predicted) | NGB |
| TargetScan Human | Moderate (predicted) | NHS |
| TargetScan Human | Moderate (predicted) | NLRC5 |
| TargetScan Human | Moderate (predicted) | NOL10 |
| TargetScan Human | Moderate (predicted) | NOTCH3 |
| TargetScan Human | Moderate (predicted) | NQO1 |
| TargetScan Human | Moderate (predicted) | NR1I2 |
| TargetScan Human | Moderate (predicted) | NRIP2 |
| TargetScan Human | Moderate (predicted) | NRP2 |
| TargetScan Human | Moderate (predicted) | NUDCD3 |
| TargetScan Human | Moderate (predicted) | NUP210 |
| TargetScan Human | Moderate (predicted) | NUP43 |
| TargetScan Human | Moderate (predicted) | OAZ2 |
| TargetScan Human | Moderate (predicted) | OGFOD1 |
| TargetScan Human | Moderate (predicted) | OPN4 |
| TargetScan Human | Moderate (predicted) | OPRM1 |
| TargetScan Human | Moderate (predicted) | ORAOV1 |
| TargetScan Human | Moderate (predicted) | OVOL1 |
| TargetScan Human | Moderate (predicted) | PADI1 |
| TargetScan Human | Moderate (predicted) | PADI2 |
| TargetScan Human | Moderate (predicted) | PAFAH1B2 |
| TargetScan Human | Moderate (predicted) | PAFAH2 |
| TargetScan Human | Moderate (predicted) | PALLD |
| TargetScan Human | Moderate (predicted) | PAPD5 |
| TargetScan Human | Moderate (predicted) | PAQR8 |
| TargetScan Human | Moderate (predicted) | PARVA |
| TargetScan Human | Moderate (predicted) | PAX7 |
| TargetScan Human | Moderate (predicted) | PCBP4 |
| TargetScan Human | Moderate (predicted) | PCSK7 |
| TargetScan Human | Moderate (predicted) | PDE11A |
| TargetScan Human | Moderate (predicted) | PDLIM7 |
| TargetScan Human | Moderate (predicted) | PER2 |
| TargetScan Human | Moderate (predicted) | PEX2 |
| TargetScan Human | Moderate (predicted) | PEX3 |
| TargetScan Human | Moderate (predicted) | PFKFB3 |
| TargetScan Human | Moderate (predicted) | PGD |
| TargetScan Human | Moderate (predicted) | PHKG2 |
| TargetScan Human | Moderate (predicted) | PI4KB |
| TargetScan Human | Moderate (predicted) | PIK3R2 |
| TargetScan Human | Moderate (predicted) | PLA2G4D |
| TargetScan Human | Moderate (predicted) | PLA2G6 |
| TargetScan Human | Moderate (predicted) | PLAGL1 |
| TargetScan Human | Moderate (predicted) | PLCB1 |
| TargetScan Human | Moderate (predicted) | PLCXD3 |
| TargetScan Human | Moderate (predicted) | PLEKHG2 |
| TargetScan Human | Moderate (predicted) | PLEKHM1 |
| TargetScan Human | Moderate (predicted) | PLPPR2 |
| TargetScan Human | Moderate (predicted) | PMF1/PMF1-BGLAP |
| TargetScan Human | Moderate (predicted) | PNMA3 |
| TargetScan Human | Moderate (predicted) | PNP |
| TargetScan Human | Moderate (predicted) | PNPLA8 |
| TargetScan Human | Moderate (predicted) | POLR2F |
| TargetScan Human | Moderate (predicted) | POLR2J2/POLR2J3 |
| TargetScan Human | Moderate (predicted) | POP4 |
| TargetScan Human | Moderate (predicted) | PPARG |
| TargetScan Human | Moderate (predicted) | PPIE |
| TargetScan Human | Moderate (predicted) | PPIL2 |
| TargetScan Human | Moderate (predicted) | PPM1M |
| TargetScan Human | Moderate (predicted) | PPP1R8 |
| TargetScan Human | Moderate (predicted) | PPP2R5A |
| TargetScan Human | Moderate (predicted) | PPP3R1 |
| TargetScan Human | Moderate (predicted) | PRELID2 |
| TargetScan Human | Moderate (predicted) | PRKAG1 |
| TargetScan Human | Moderate (predicted) | PRKCB |
| TargetScan Human | Moderate (predicted) | PRKCH |
| TargetScan Human | Moderate (predicted) | PRLR |
| TargetScan Human | Moderate (predicted) | PRMT2 |
| TargetScan Human | Moderate (predicted) | PROSC |
| TargetScan Human | Moderate (predicted) | PROZ |
| TargetScan Human | Moderate (predicted) | PRR11 |
| TargetScan Human | Moderate (predicted) | PRR23B |
| TargetScan Human | Moderate (predicted) | PRR3 |
| TargetScan Human | Moderate (predicted) | PRSS21 |
| TargetScan Human | Moderate (predicted) | PSMD5 |
| TargetScan Human | Moderate (predicted) | PSME1 |
| TargetScan Human | Moderate (predicted) | PSME3 |
| TargetScan Human | Moderate (predicted) | PSTK |
| TargetScan Human | Moderate (predicted) | PTPN14 |
| TargetScan Human | Moderate (predicted) | PTPN18 |
| TargetScan Human | Moderate (predicted) | PTPRB |
| TargetScan Human | Moderate (predicted) | PTPRR |
| TargetScan Human | Moderate (predicted) | PTRH1 |
| TargetScan Human | Moderate (predicted) | PUS7L |
| TargetScan Human | Moderate (predicted) | PVR |
| TargetScan Human | Moderate (predicted) | PVRL2 |
| TargetScan Human | Moderate (predicted) | PYDC1 |
| TargetScan Human | Moderate (predicted) | RAB21 |
| TargetScan Human | Moderate (predicted) | RAB29 |
| TargetScan Human | Moderate (predicted) | RAB3B |
| TargetScan Human | Moderate (predicted) | RAB43 |
| TargetScan Human | Moderate (predicted) | RAB4A |
| TargetScan Human | Moderate (predicted) | RAD23B |
| TargetScan Human | Moderate (predicted) | RAD9A |
| TargetScan Human | Moderate (predicted) | RAI14 |
| TargetScan Human | Moderate (predicted) | RASA4 |
| TargetScan Human | Moderate (predicted) | RBM28 |
| TargetScan Human | Moderate (predicted) | RBMS2 |
| TargetScan Human | Moderate (predicted) | RBMXL1 |
| TargetScan Human | Moderate (predicted) | RDH11 |
| TargetScan Human | Moderate (predicted) | RDH12 |
| TargetScan Human | Moderate (predicted) | RGS6 |
| TargetScan Human | Moderate (predicted) | RHOBTB3 |
| TargetScan Human | Moderate (predicted) | RHOJ |
| TargetScan Human | Moderate (predicted) | RHOQ |
| TargetScan Human | Moderate (predicted) | RIMBP2 |
| TargetScan Human | Moderate (predicted) | RIMKLA |
| TargetScan Human | Moderate (predicted) | RNASEL |
| TargetScan Human | Moderate (predicted) | RNF103-CHMP3 |
| TargetScan Human | Moderate (predicted) | RNF170 |
| TargetScan Human | Moderate (predicted) | RNF182 |
| TargetScan Human | Moderate (predicted) | RNF216 |
| TargetScan Human | Moderate (predicted) | RNPS1 |
| TargetScan Human | Moderate (predicted) | ROGDI |
| TargetScan Human | Moderate (predicted) | RPL13A |
| TargetScan Human | Moderate (predicted) | RPL32 |
| TargetScan Human | Moderate (predicted) | RPS24 |
| TargetScan Human | Moderate (predicted) | RRAGD |
| TargetScan Human | Moderate (predicted) | RSPO1 |
| TargetScan Human | Moderate (predicted) | S1PR2 |
| TargetScan Human | Moderate (predicted) | S1PR3 |
| TargetScan Human | Moderate (predicted) | SAMD4A |
| TargetScan Human | Moderate (predicted) | SCN4A |
| TargetScan Human | Moderate (predicted) | SCUBE2 |
| TargetScan Human | Moderate (predicted) | SCUBE3 |
| TargetScan Human | Moderate (predicted) | SEC16A |
| TargetScan Human | Moderate (predicted) | SEC61A1 |
| TargetScan Human | Moderate (predicted) | SELPLG |
| TargetScan Human | Moderate (predicted) | SEMA3E |
| TargetScan Human | Moderate (predicted) | SEMA6A |
| TargetScan Human | Moderate (predicted) | SENP3 |
| TargetScan Human | Moderate (predicted) | SEPT6 |
| TargetScan Human | Moderate (predicted) | SERINC1 |
| TargetScan Human | Moderate (predicted) | SERPINB2 |
| TargetScan Human | Moderate (predicted) | SERPINB8 |
| TargetScan Human | Moderate (predicted) | SESTD1 |
| TargetScan Human | Moderate (predicted) | SFT2D3 |
| TargetScan Human | Moderate (predicted) | SFXN2 |
| TargetScan Human | Moderate (predicted) | SH3BGRL2 |
| TargetScan Human | Moderate (predicted) | SH3PXD2B |
| TargetScan Human | Moderate (predicted) | SHMT1 |
| TargetScan Human | Moderate (predicted) | SHOC2 |
| TargetScan Human | Moderate (predicted) | SHOX |
| TargetScan Human | Moderate (predicted) | SLC10A2 |
| TargetScan Human | Moderate (predicted) | SLC15A5 |
| TargetScan Human | Moderate (predicted) | SLC16A1 |
| TargetScan Human | Moderate (predicted) | SLC16A14 |
| TargetScan Human | Moderate (predicted) | SLC22A12 |
| TargetScan Human | Moderate (predicted) | SLC22A15 |
| TargetScan Human | Moderate (predicted) | SLC25A53 |
| TargetScan Human | Moderate (predicted) | SLC26A9 |
| TargetScan Human | Moderate (predicted) | SLC2A8 |
| TargetScan Human | Moderate (predicted) | SLC30A6 |
| TargetScan Human | Moderate (predicted) | SLC35D2 |
| TargetScan Human | Moderate (predicted) | SLC35E2 |
| TargetScan Human | Moderate (predicted) | SLC35G2 |
| TargetScan Human | Moderate (predicted) | SLC38A1 |
| TargetScan Human | Moderate (predicted) | SLC39A9 |
| TargetScan Human | Moderate (predicted) | SLC6A14 |
| TargetScan Human | Moderate (predicted) | SLC6A3 |
| TargetScan Human | Moderate (predicted) | SLC7A10 |
| TargetScan Human | Moderate (predicted) | SLCO2A1 |
| TargetScan Human | Moderate (predicted) | SLITRK4 |
| TargetScan Human | Moderate (predicted) | SLX4 |
| TargetScan Human | Moderate (predicted) | SMIM13 |
| TargetScan Human | Moderate (predicted) | SMIM3 |
| TargetScan Human | Moderate (predicted) | SMPD1 |
| TargetScan Human | Moderate (predicted) | SMTNL2 |
| TargetScan Human | Moderate (predicted) | SNAPC1 |
| TargetScan Human | Moderate (predicted) | SNCA |
| TargetScan Human | Moderate (predicted) | SNED1 |
| TargetScan Human | Moderate (predicted) | SNX9 |
| TargetScan Human | Moderate (predicted) | SOCS7 |
| TargetScan Human | Moderate (predicted) | SPATA12 |
| TargetScan Human | Moderate (predicted) | SPEN |
| TargetScan Human | Moderate (predicted) | SPON1 |
| TargetScan Human | Moderate (predicted) | SPRR2G |
| TargetScan Human | Moderate (predicted) | SPSB4 |
| TargetScan Human | Moderate (predicted) | SRGAP2 |
| TargetScan Human | Moderate (predicted) | SRXN1 |
| TargetScan Human | Moderate (predicted) | STARD3 |
| TargetScan Human | Moderate (predicted) | STC1 |
| TargetScan Human | Moderate (predicted) | STK35 |
| TargetScan Human | Moderate (predicted) | STMN1 |
| TargetScan Human | Moderate (predicted) | STMN3 |
| TargetScan Human | Moderate (predicted) | STRAP |
| TargetScan Human | Moderate (predicted) | SUCO |
| TargetScan Human | Moderate (predicted) | SULT4A1 |
| TargetScan Human | Moderate (predicted) | SUPT6H |
| TargetScan Human | Moderate (predicted) | SURF6 |
| TargetScan Human | Moderate (predicted) | SVIL |
| TargetScan Human | Moderate (predicted) | SWAP70 |
| TargetScan Human | Moderate (predicted) | SWT1 |
| TargetScan Human | Moderate (predicted) | SYNPO |
| TargetScan Human | Moderate (predicted) | SYT15 |
| TargetScan Human | Moderate (predicted) | TAL2 |
| TargetScan Human | Moderate (predicted) | TATDN2 |
| TargetScan Human | Moderate (predicted) | TBC1D2 |
| TargetScan Human | Moderate (predicted) | TBC1D22A |
| TargetScan Human | Moderate (predicted) | TBRG1 |
| TargetScan Human | Moderate (predicted) | TCTEX1D1 |
| TargetScan Human | Moderate (predicted) | TEDDM1 |
| TargetScan Human | Moderate (predicted) | TESPA1 |
| TargetScan Human | Moderate (predicted) | TEX22 |
| TargetScan Human | Moderate (predicted) | TEX264 |
| TargetScan Human | Moderate (predicted) | TGFA |
| TargetScan Human | Moderate (predicted) | TGFBI |
| TargetScan Human | Moderate (predicted) | TGS1 |
| TargetScan Human | Moderate (predicted) | TH |
| TargetScan Human | Moderate (predicted) | THBD |
| TargetScan Human | Moderate (predicted) | THPO |
| TargetScan Human | Moderate (predicted) | TIGD6 |
| TargetScan Human | Moderate (predicted) | TKFC |
| TargetScan Human | Moderate (predicted) | TLX2 |
| TargetScan Human | Moderate (predicted) | TMBIM1 |
| TargetScan Human | Moderate (predicted) | TMEFF1 |
| TargetScan Human | Moderate (predicted) | TMEM136 |
| TargetScan Human | Moderate (predicted) | TMEM141 |
| TargetScan Human | Moderate (predicted) | TMEM167A |
| TargetScan Human | Moderate (predicted) | TMEM173 |
| TargetScan Human | Moderate (predicted) | TMEM234 |
| TargetScan Human | Moderate (predicted) | TMEM25 |
| TargetScan Human | Moderate (predicted) | TMEM59L |
| TargetScan Human | Moderate (predicted) | TMEM79 |
| TargetScan Human | Moderate (predicted) | TMPRSS4 |
| TargetScan Human | Moderate (predicted) | TMPRSS6 |
| TargetScan Human | Moderate (predicted) | TNFSF14 |
| TargetScan Human | Moderate (predicted) | TNS2 |
| TargetScan Human | Moderate (predicted) | TOR1A |
| TargetScan Human | Moderate (predicted) | TP53INP1 |
| TargetScan Human | Moderate (predicted) | TP53TG3B (includes others) |
| TargetScan Human | Moderate (predicted) | TPPP |
| TargetScan Human | Moderate (predicted) | TRAFD1 |
| TargetScan Human | Moderate (predicted) | TRAPPC6A |
| TargetScan Human | Moderate (predicted) | TREM1 |
| TargetScan Human | Moderate (predicted) | TRIM10 |
| TargetScan Human | Moderate (predicted) | TRIM21 |
| TargetScan Human | Moderate (predicted) | TRIM32 |
| TargetScan Human | Moderate (predicted) | TRIM62 |
| TargetScan Human | Moderate (predicted) | TRIQK |
| TargetScan Human | Moderate (predicted) | TRMT61A |
| TargetScan Human | Moderate (predicted) | TRNP1 |
| TargetScan Human | Moderate (predicted) | TSPAN33 |
| TargetScan Human | Moderate (predicted) | TSR1 |
| TargetScan Human | Moderate (predicted) | TUBA3C/TUBA3D |
| TargetScan Human | Moderate (predicted) | TUBB1 |
| TargetScan Human | Moderate (predicted) | TUSC3 |
| TargetScan Human | Moderate (predicted) | UACA |
| TargetScan Human | Moderate (predicted) | UBA1 |
| TargetScan Human | Moderate (predicted) | UBA5 |
| TargetScan Human | Moderate (predicted) | UBE2H |
| TargetScan Human | Moderate (predicted) | UBE2K |
| TargetScan Human | Moderate (predicted) | UBE2NL |
| TargetScan Human | Moderate (predicted) | UBL4A |
| TargetScan Human | Moderate (predicted) | ULBP2 |
| TargetScan Human | Moderate (predicted) | ULK4 |
| TargetScan Human | Moderate (predicted) | UNC119 |
| TargetScan Human | Moderate (predicted) | UNC5A |
| TargetScan Human | Moderate (predicted) | UQCRQ |
| TargetScan Human | Moderate (predicted) | USP54 |
| TargetScan Human | Moderate (predicted) | USP8 |
| TargetScan Human | Moderate (predicted) | VPS52 |
| TargetScan Human | Moderate (predicted) | VSIG1 |
| TargetScan Human | Moderate (predicted) | WDR33 |
| TargetScan Human | Moderate (predicted) | WDR66 |
| TargetScan Human | Moderate (predicted) | WDR77 |
| TargetScan Human | Moderate (predicted) | WFDC5 |
| TargetScan Human | Moderate (predicted) | WIPI2 |
| TargetScan Human | Moderate (predicted) | WNT2B |
| TargetScan Human | Moderate (predicted) | WNT9B |
| TargetScan Human | Moderate (predicted) | WWC3 |
| TargetScan Human | Moderate (predicted) | XBP1 |
| TargetScan Human | Moderate (predicted) | XPC |
| TargetScan Human | Moderate (predicted) | XPO4 |
| TargetScan Human | Moderate (predicted) | YDJC |
| TargetScan Human | Moderate (predicted) | ZBTB4 |
| TargetScan Human | Moderate (predicted) | ZBTB42 |
| TargetScan Human | Moderate (predicted) | ZDHHC15 |
| TargetScan Human | Moderate (predicted) | ZDHHC8 |
| TargetScan Human | Moderate (predicted) | ZEB1 |
| TargetScan Human | Moderate (predicted) | ZFP2 |
| TargetScan Human | Moderate (predicted) | ZMYND11 |
| TargetScan Human | Moderate (predicted) | ZNF169 |
| TargetScan Human | Moderate (predicted) | ZNF207 |
| TargetScan Human | Moderate (predicted) | ZNF324 |
| TargetScan Human | Moderate (predicted) | ZNF324B |
| TargetScan Human | Moderate (predicted) | ZNF502 |
| TargetScan Human | Moderate (predicted) | ZNF512B |
| TargetScan Human | Moderate (predicted) | ZNF530 |
| TargetScan Human | Moderate (predicted) | ZNF628 |
| TargetScan Human | Moderate (predicted) | ZNF672 |
| TargetScan Human | Moderate (predicted) | ZNF707 |
| TargetScan Human | Moderate (predicted) | ZNF766 |
| TargetScan Human | Moderate (predicted) | ZNF789 |
| TargetScan Human | Moderate (predicted) | ZNF800 |
| TargetScan Human | Moderate (predicted) | ZNF821 |
| TargetScan Human | Moderate (predicted) | ZSWIM4 |

S2B Table: Verified/predicted target genes of miR-351

| Source | Status | Gene Abbv |
| --- | --- | --- |
| Ingenuity Expert Findings | Experimentally Observed | ACSS1 |
| miRecords | Experimentally Observed | ADAMTS1 |
| TarBase | Experimentally Observed | AJUBA |
| Ingenuity Expert Findings | Experimentally Observed | ALOX5 |
| TarBase | Experimentally Observed | APLN |
| miRecords | Experimentally Observed | ATP6AP1L |
| miRecords | Experimentally Observed | B3GALT4 |
| miRecords | Experimentally Observed | CASP6 |
| miRecords | Experimentally Observed | CASP7 |
| Ingenuity Expert Findings | Experimentally Observed | CBFB |
| miRecords | Experimentally Observed | CBLN2 |
| miRecords | Experimentally Observed | CDC25A |
| miRecords | Experimentally Observed | CDK6 |
| miRecords | Experimentally Observed | CDKN2A |
| miRecords | Experimentally Observed | CEBPG |
| miRecords | Experimentally Observed | CYP1A1 |
| TarBase | Experimentally Observed | DDX19B |
| miRecords | Experimentally Observed | DIO3 |
| miRecords | Experimentally Observed | FAM19A1 |
| miRecords | Experimentally Observed | H3F3A/H3F3B |
| miRecords | Experimentally Observed | HIST1H4A |
| miRecords | Experimentally Observed | ID1 |
| miRecords | Experimentally Observed | ID2 |
| miRecords | Experimentally Observed | ID3 |
| miRecords | Experimentally Observed | IL1RN |
| miRecords | Experimentally Observed | JARID2 |
| miRecords | Experimentally Observed | KRT19 |
| miRecords | Experimentally Observed | MAN1A1 |
| Ingenuity Expert Findings | Experimentally Observed | MAZ |
| Ingenuity Expert Findings | Experimentally Observed | MYD88 |
| miRecords | Experimentally Observed | PCDHB10 |
| miRecords | Experimentally Observed | PERP |
| miRecords | Experimentally Observed | PIGR |
| TarBase | Experimentally Observed | PPT2 |
| miRecords | Experimentally Observed | RBM8A |
| TarBase | Experimentally Observed | RHEBL1 |
| miRecords | Experimentally Observed | SMO |
| miRecords | Experimentally Observed | TENM2 |
| miRecords | Experimentally Observed | TSPAN8 |
| miRecords | Experimentally Observed | UBE2I |
| miRecords | Experimentally Observed | UGT2B15 |
| miRecords | Experimentally Observed | UGT2B17 |
| miRecords | Experimentally Observed | UGT2B28 |
| TarBase,TargetScan Human | Experimentally Observed,High (predicted) | ABTB1 |
| TargetScan Human,miRecords | Experimentally Observed,High (predicted) | ANAPC16 |
| TarBase,TargetScan Human | Experimentally Observed,High (predicted) | ARID3A |
| TarBase,TargetScan Human,miRecords | Experimentally Observed,High (predicted) | ARID3B |
| Ingenuity Expert Findings,TargetScan Human,miRecords | Experimentally Observed,High (predicted) | BAK1 |
| TargetScan Human,miRecords | Experimentally Observed,High (predicted) | BMF |
| TargetScan Human,miRecords | Experimentally Observed,High (predicted) | BMPR1B |
| Ingenuity Expert Findings,TargetScan Human | Experimentally Observed,High (predicted) | C10orf54 |
| TargetScan Human,miRecords | Experimentally Observed,High (predicted) | CBX7 |
| Ingenuity Expert Findings,TargetScan Human | Experimentally Observed,High (predicted) | CCR5 |
| Ingenuity Expert Findings,TargetScan Human | Experimentally Observed,High (predicted) | CDH5 |
| TargetScan Human,miRecords | Experimentally Observed,High (predicted) | DICER1 |
| TarBase,TargetScan Human | Experimentally Observed,High (predicted) | DUS1L |
| Ingenuity Expert Findings,TargetScan Human | Experimentally Observed,High (predicted) | E2F3 |
| TargetScan Human,miRecords | Experimentally Observed,High (predicted) | ELAVL1 |
| TarBase,TargetScan Human | Experimentally Observed,High (predicted) | ENTPD4 |
| TargetScan Human,miRecords | Experimentally Observed,High (predicted) | ERBB3 |
| Ingenuity Expert Findings,TargetScan Human | Experimentally Observed,High (predicted) | HK2 |
| Ingenuity Expert Findings,TargetScan Human | Experimentally Observed,High (predicted) | IKZF4 |
| Ingenuity Expert Findings,TargetScan Human | Experimentally Observed,High (predicted) | KLF13 |
| TarBase,TargetScan Human,miRecords | Experimentally Observed,High (predicted) | LIN28A |
| Ingenuity Expert Findings,TargetScan Human | Experimentally Observed,High (predicted) | LIPA |
| TarBase,TargetScan Human | Experimentally Observed,High (predicted) | MAP2K7 |
| Ingenuity Expert Findings,TargetScan Human | Experimentally Observed,High (predicted) | PCTP |
| TarBase,TargetScan Human | Experimentally Observed,High (predicted) | RABL6 |
| Ingenuity Expert Findings,TargetScan Human | Experimentally Observed,High (predicted) | SCD |
| TargetScan Human,miRecords | Experimentally Observed,High (predicted) | SGPL1 |
| TargetScan Human,miRecords | Experimentally Observed,High (predicted) | ST18 |
| TarBase,TargetScan Human | Experimentally Observed,High (predicted) | TOR2A |
| Ingenuity Expert Findings,TargetScan Human | Experimentally Observed,High (predicted) | UVRAG |
| TarBase,TargetScan Human | Experimentally Observed,High (predicted) | ZNF385A |
| TargetScan Human,miRecords | Experimentally Observed,Moderate (predicted) | ERBB2 |
| TargetScan Human,miRecords | Experimentally Observed,Moderate (predicted) | GPR160 |
| Ingenuity Expert Findings,TargetScan Human | Experimentally Observed,Moderate (predicted) | GSS |
| TargetScan Human,miRecords | Experimentally Observed,Moderate (predicted) | IGFBP3 |
| Ingenuity Expert Findings,TargetScan Human,miRecords | Experimentally Observed,Moderate (predicted) | TP53 |
| TargetScan Human | High (predicted) | ABCC4 |
| TargetScan Human | High (predicted) | ABHD3 |
| TargetScan Human | High (predicted) | ABHD6 |
| TargetScan Human | High (predicted) | ABL1 |
| TargetScan Human | High (predicted) | ABL2 |
| TargetScan Human | High (predicted) | ABR |
| TargetScan Human | High (predicted) | ACACB |
| TargetScan Human | High (predicted) | ACER2 |
| TargetScan Human | High (predicted) | ACHE |
| TargetScan Human | High (predicted) | ACOT13 |
| TargetScan Human | High (predicted) | ACSL6 |
| TargetScan Human | High (predicted) | ACVR1C |
| TargetScan Human | High (predicted) | ADAM11 |
| TargetScan Human | High (predicted) | ADAM9 |
| TargetScan Human | High (predicted) | ADAMTS14 |
| TargetScan Human | High (predicted) | ADAMTS15 |
| TargetScan Human | High (predicted) | ADAMTS4 |
| TargetScan Human | High (predicted) | ADCY1 |
| TargetScan Human | High (predicted) | ADGRG5 |
| TargetScan Human | High (predicted) | ADPRH |
| TargetScan Human | High (predicted) | ADRBK1 |
| TargetScan Human | High (predicted) | AGGF1 |
| TargetScan Human | High (predicted) | AGO2 |
| TargetScan Human | High (predicted) | AGXT2 |
| TargetScan Human | High (predicted) | AHRR |
| TargetScan Human | High (predicted) | AKAP13 |
| TargetScan Human | High (predicted) | AKT1S1 |
| TargetScan Human | High (predicted) | ALDH1A3 |
| TargetScan Human | High (predicted) | ALPK3 |
| TargetScan Human | High (predicted) | AMER2 |
| TargetScan Human | High (predicted) | AMIGO2 |
| TargetScan Human | High (predicted) | ANGPT2 |
| TargetScan Human | High (predicted) | ANKH |
| TargetScan Human | High (predicted) | ANKRD13B |
| TargetScan Human | High (predicted) | ANKRD29 |
| TargetScan Human | High (predicted) | ANKRD33B |
| TargetScan Human | High (predicted) | ANKRD42 |
| TargetScan Human | High (predicted) | ANKRD44 |
| TargetScan Human | High (predicted) | ANKRD50 |
| TargetScan Human | High (predicted) | ANKZF1 |
| TargetScan Human | High (predicted) | ANO6 |
| TargetScan Human | High (predicted) | ANPEP |
| TargetScan Human | High (predicted) | ANTXR2 |
| TargetScan Human | High (predicted) | AP5Z1 |
| TargetScan Human | High (predicted) | APBA1 |
| TargetScan Human | High (predicted) | APC |
| TargetScan Human | High (predicted) | ARCN1 |
| TargetScan Human | High (predicted) | AREL1 |
| TargetScan Human | High (predicted) | ARF3 |
| TargetScan Human | High (predicted) | ARFGEF3 |
| TargetScan Human | High (predicted) | ARHGAP40 |
| TargetScan Human | High (predicted) | ARHGEF1 |
| TargetScan Human | High (predicted) | ARHGEF39 |
| TargetScan Human | High (predicted) | ARMC7 |
| TargetScan Human | High (predicted) | ARRB1 |
| TargetScan Human | High (predicted) | ARSI |
| TargetScan Human | High (predicted) | ASB13 |
| TargetScan Human | High (predicted) | ASB4 |
| TargetScan Human | High (predicted) | ASIC1 |
| TargetScan Human | High (predicted) | ASPH |
| TargetScan Human | High (predicted) | ASPHD1 |
| TargetScan Human | High (predicted) | ATG4D |
| TargetScan Human | High (predicted) | ATOH8 |
| TargetScan Human | High (predicted) | ATP10D |
| TargetScan Human | High (predicted) | ATP13A3 |
| TargetScan Human | High (predicted) | ATP1B4 |
| TargetScan Human | High (predicted) | ATP2B3 |
| TargetScan Human | High (predicted) | ATP5G2 |
| TargetScan Human | High (predicted) | ATXN1 |
| TargetScan Human | High (predicted) | ATXN1L |
| TargetScan Human | High (predicted) | ATXN3 |
| TargetScan Human | High (predicted) | ATXN7L3 |
| TargetScan Human | High (predicted) | B3GALNT2 |
| TargetScan Human | High (predicted) | B4GALT1 |
| TargetScan Human | High (predicted) | B4GALT6 |
| TargetScan Human | High (predicted) | BACH1 |
| TargetScan Human | High (predicted) | BAG4 |
| TargetScan Human | High (predicted) | BAP1 |
| TargetScan Human | High (predicted) | BAZ2A |
| TargetScan Human | High (predicted) | BBC3 |
| TargetScan Human | High (predicted) | BCAN |
| TargetScan Human | High (predicted) | BCAT1 |
| TargetScan Human | High (predicted) | BCL2 |
| TargetScan Human | High (predicted) | BCL2L12 |
| TargetScan Human | High (predicted) | BCL2L14 |
| TargetScan Human | High (predicted) | BCL2L2 |
| TargetScan Human | High (predicted) | BDH1 |
| TargetScan Human | High (predicted) | BIN2 |
| TargetScan Human | High (predicted) | BMPR2 |
| TargetScan Human | High (predicted) | BORCS6 |
| TargetScan Human | High (predicted) | BRPF1 |
| TargetScan Human | High (predicted) | BRSK2 |
| TargetScan Human | High (predicted) | BRWD1 |
| TargetScan Human | High (predicted) | BSN |
| TargetScan Human | High (predicted) | C11orf21 |
| TargetScan Human | High (predicted) | C11orf57 |
| TargetScan Human | High (predicted) | C15orf39 |
| TargetScan Human | High (predicted) | C15orf53 |
| TargetScan Human | High (predicted) | C17orf102 |
| TargetScan Human | High (predicted) | C17orf51 |
| TargetScan Human | High (predicted) | C19orf38 |
| TargetScan Human | High (predicted) | C19orf54 |
| TargetScan Human | High (predicted) | C1orf210 |
| TargetScan Human | High (predicted) | C2orf88 |
| TargetScan Human | High (predicted) | C4orf19 |
| TargetScan Human | High (predicted) | C6orf47 |
| TargetScan Human | High (predicted) | CABP5 |
| TargetScan Human | High (predicted) | CACNA1B |
| TargetScan Human | High (predicted) | CACNB1 |
| TargetScan Human | High (predicted) | CACNB2 |
| TargetScan Human | High (predicted) | CACNB3 |
| TargetScan Human | High (predicted) | CACNG2 |
| TargetScan Human | High (predicted) | CALCOCO2 |
| TargetScan Human | High (predicted) | CAMSAP3 |
| TargetScan Human | High (predicted) | CASP2 |
| TargetScan Human | High (predicted) | CBLL1 |
| TargetScan Human | High (predicted) | CCBE1 |
| TargetScan Human | High (predicted) | CCDC142 |
| TargetScan Human | High (predicted) | CCDC71L |
| TargetScan Human | High (predicted) | CCDC85C |
| TargetScan Human | High (predicted) | CCNJ |
| TargetScan Human | High (predicted) | CCNJL |
| TargetScan Human | High (predicted) | CCR2 |
| TargetScan Human | High (predicted) | CD248 |
| TargetScan Human | High (predicted) | CD300LB |
| TargetScan Human | High (predicted) | CD300LF |
| TargetScan Human | High (predicted) | CD5L |
| TargetScan Human | High (predicted) | CD69 |
| TargetScan Human | High (predicted) | CDC14B |
| TargetScan Human | High (predicted) | CDC42BPG |
| TargetScan Human | High (predicted) | CDC42SE1 |
| TargetScan Human | High (predicted) | CDK16 |
| TargetScan Human | High (predicted) | CDK19 |
| TargetScan Human | High (predicted) | CDKN2B |
| TargetScan Human | High (predicted) | CDR2L |
| TargetScan Human | High (predicted) | CDS2 |
| TargetScan Human | High (predicted) | CELSR2 |
| TargetScan Human | High (predicted) | CEP164 |
| TargetScan Human | High (predicted) | CEP85 |
| TargetScan Human | High (predicted) | CGN |
| TargetScan Human | High (predicted) | CGREF1 |
| TargetScan Human | High (predicted) | CHAMP1 |
| TargetScan Human | High (predicted) | CHTF8 |
| TargetScan Human | High (predicted) | CLDN12 |
| TargetScan Human | High (predicted) | CLEC16A |
| TargetScan Human | High (predicted) | CLIP2 |
| TargetScan Human | High (predicted) | CNNM1 |
| TargetScan Human | High (predicted) | COL11A2 |
| TargetScan Human | High (predicted) | COL4A3 |
| TargetScan Human | High (predicted) | COPS7B |
| TargetScan Human | High (predicted) | COPZ1 |
| TargetScan Human | High (predicted) | CORO1C |
| TargetScan Human | High (predicted) | CORO2A |
| TargetScan Human | High (predicted) | CORO2B |
| TargetScan Human | High (predicted) | CORO7/CORO7-PAM16 |
| TargetScan Human | High (predicted) | CPEB3 |
| TargetScan Human | High (predicted) | CPM |
| TargetScan Human | High (predicted) | CPSF6 |
| TargetScan Human | High (predicted) | CRAMP1 |
| TargetScan Human | High (predicted) | CRB2 |
| TargetScan Human | High (predicted) | CREB1 |
| TargetScan Human | High (predicted) | CRTC1 |
| TargetScan Human | High (predicted) | CSNK1G1 |
| TargetScan Human | High (predicted) | CSNK2A1 |
| TargetScan Human | High (predicted) | CSRNP3 |
| TargetScan Human | High (predicted) | CTDSP2 |
| TargetScan Human | High (predicted) | CTU1 |
| TargetScan Human | High (predicted) | CXCL13 |
| TargetScan Human | High (predicted) | CXorf23 |
| TargetScan Human | High (predicted) | CYB5D1 |
| TargetScan Human | High (predicted) | CYP24A1 |
| TargetScan Human | High (predicted) | CYTH1 |
| TargetScan Human | High (predicted) | CYTH2 |
| TargetScan Human | High (predicted) | DAAM2 |
| TargetScan Human | High (predicted) | DAGLA |
| TargetScan Human | High (predicted) | DAZAP2 |
| TargetScan Human | High (predicted) | DCP1A |
| TargetScan Human | High (predicted) | DCTN1 |
| TargetScan Human | High (predicted) | DDX42 |
| TargetScan Human | High (predicted) | DDX54 |
| TargetScan Human | High (predicted) | DENND6A |
| TargetScan Human | High (predicted) | DGAT1 |
| TargetScan Human | High (predicted) | DHX33 |
| TargetScan Human | High (predicted) | DIP2A |
| TargetScan Human | High (predicted) | DIRAS1 |
| TargetScan Human | High (predicted) | DIS3 |
| TargetScan Human | High (predicted) | DIS3L2 |
| TargetScan Human | High (predicted) | DLD |
| TargetScan Human | High (predicted) | DNAJB2 |
| TargetScan Human | High (predicted) | DNAJB5 |
| TargetScan Human | High (predicted) | DNAJC14 |
| TargetScan Human | High (predicted) | DNAL1 |
| TargetScan Human | High (predicted) | DNAL4 |
| TargetScan Human | High (predicted) | DOCK3 |
| TargetScan Human | High (predicted) | DPF2 |
| TargetScan Human | High (predicted) | DPH2 |
| TargetScan Human | High (predicted) | DPP9 |
| TargetScan Human | High (predicted) | DPYSL4 |
| TargetScan Human | High (predicted) | DRAM2 |
| TargetScan Human | High (predicted) | DTX4 |
| TargetScan Human | High (predicted) | DUSP3 |
| TargetScan Human | High (predicted) | DUSP6 |
| TargetScan Human | High (predicted) | DUSP7 |
| TargetScan Human | High (predicted) | DVL3 |
| TargetScan Human | High (predicted) | DYNLT3 |
| TargetScan Human | High (predicted) | E2F2 |
| TargetScan Human | High (predicted) | EAF1 |
| TargetScan Human | High (predicted) | EBF4 |
| TargetScan Human | High (predicted) | ECE1 |
| TargetScan Human | High (predicted) | EDC3 |
| TargetScan Human | High (predicted) | EDEM1 |
| TargetScan Human | High (predicted) | EIF1AD |
| TargetScan Human | High (predicted) | EIF2B5 |
| TargetScan Human | High (predicted) | EIF4EBP1 |
| TargetScan Human | High (predicted) | EIF5A2 |
| TargetScan Human | High (predicted) | ELAC2 |
| TargetScan Human | High (predicted) | ELL |
| TargetScan Human | High (predicted) | ELMSAN1 |
| TargetScan Human | High (predicted) | ELOVL1 |
| TargetScan Human | High (predicted) | ELOVL4 |
| TargetScan Human | High (predicted) | ELOVL6 |
| TargetScan Human | High (predicted) | EMID1 |
| TargetScan Human | High (predicted) | ENPEP |
| TargetScan Human | High (predicted) | ENPP1 |
| TargetScan Human | High (predicted) | ENTPD1 |
| TargetScan Human | High (predicted) | ENTPD3 |
| TargetScan Human | High (predicted) | EPHA10 |
| TargetScan Human | High (predicted) | EPO |
| TargetScan Human | High (predicted) | ERBB4 |
| TargetScan Human | High (predicted) | ERCC6 |
| TargetScan Human | High (predicted) | ESRRA |
| TargetScan Human | High (predicted) | ESRRG |
| TargetScan Human | High (predicted) | ESYT1 |
| TargetScan Human | High (predicted) | ETS1 |
| TargetScan Human | High (predicted) | ETV6 |
| TargetScan Human | High (predicted) | EVA1A |
| TargetScan Human | High (predicted) | EXO5 |
| TargetScan Human | High (predicted) | FAM107B |
| TargetScan Human | High (predicted) | FAM118A |
| TargetScan Human | High (predicted) | FAM126B |
| TargetScan Human | High (predicted) | FAM129B |
| TargetScan Human | High (predicted) | FAM131B |
| TargetScan Human | High (predicted) | FAM134A |
| TargetScan Human | High (predicted) | FAM134C |
| TargetScan Human | High (predicted) | FAM135B |
| TargetScan Human | High (predicted) | FAM169B |
| TargetScan Human | High (predicted) | FAM174B |
| TargetScan Human | High (predicted) | FAM210B |
| TargetScan Human | High (predicted) | FAM213A |
| TargetScan Human | High (predicted) | FAM234B |
| TargetScan Human | High (predicted) | FAM46A |
| TargetScan Human | High (predicted) | FAM53C |
| TargetScan Human | High (predicted) | FAM65B |
| TargetScan Human | High (predicted) | FAM71D |
| TargetScan Human | High (predicted) | FAM73A |
| TargetScan Human | High (predicted) | FAM78A |
| TargetScan Human | High (predicted) | FAM83F |
| TargetScan Human | High (predicted) | FAM83H |
| TargetScan Human | High (predicted) | FAM92B |
| TargetScan Human | High (predicted) | FAT4 |
| TargetScan Human | High (predicted) | FBXO10 |
| TargetScan Human | High (predicted) | FBXO45 |
| TargetScan Human | High (predicted) | FBXW4 |
| TargetScan Human | High (predicted) | FBXW8 |
| TargetScan Human | High (predicted) | FGF14 |
| TargetScan Human | High (predicted) | FGF9 |
| TargetScan Human | High (predicted) | FGFR1 |
| TargetScan Human | High (predicted) | FGFR2 |
| TargetScan Human | High (predicted) | FIBP |
| TargetScan Human | High (predicted) | FLOT2 |
| TargetScan Human | High (predicted) | FLVCR2 |
| TargetScan Human | High (predicted) | FNDC3B |
| TargetScan Human | High (predicted) | FOXD2 |
| TargetScan Human | High (predicted) | FOXK2 |
| TargetScan Human | High (predicted) | FOXN3 |
| TargetScan Human | High (predicted) | FOXQ1 |
| TargetScan Human | High (predicted) | FOXS1 |
| TargetScan Human | High (predicted) | FPR3 |
| TargetScan Human | High (predicted) | FRAS1 |
| TargetScan Human | High (predicted) | FREM1 |
| TargetScan Human | High (predicted) | FRMD1 |
| TargetScan Human | High (predicted) | FURIN |
| TargetScan Human | High (predicted) | FUT1 |
| TargetScan Human | High (predicted) | FUT4 |
| TargetScan Human | High (predicted) | GAB2 |
| TargetScan Human | High (predicted) | GAB4 |
| TargetScan Human | High (predicted) | GALNT14 |
| TargetScan Human | High (predicted) | GALNT7 |
| TargetScan Human | High (predicted) | GANC |
| TargetScan Human | High (predicted) | GAS2L1 |
| TargetScan Human | High (predicted) | GCNT1 |
| TargetScan Human | High (predicted) | GDF11 |
| TargetScan Human | High (predicted) | GDNF |
| TargetScan Human | High (predicted) | GEMIN2 |
| TargetScan Human | High (predicted) | GFOD2 |
| TargetScan Human | High (predicted) | GGA2 |
| TargetScan Human | High (predicted) | GGT7 |
| TargetScan Human | High (predicted) | GJC1 |
| TargetScan Human | High (predicted) | GK5 |
| TargetScan Human | High (predicted) | GLB1L2 |
| TargetScan Human | High (predicted) | GLIPR1 |
| TargetScan Human | High (predicted) | GLS |
| TargetScan Human | High (predicted) | GLTP |
| TargetScan Human | High (predicted) | GMIP |
| TargetScan Human | High (predicted) | GOPC |
| TargetScan Human | High (predicted) | GPATCH8 |
| TargetScan Human | High (predicted) | GPC4 |
| TargetScan Human | High (predicted) | GPC6 |
| TargetScan Human | High (predicted) | GPR107 |
| TargetScan Human | High (predicted) | GPR153 |
| TargetScan Human | High (predicted) | GPR173 |
| TargetScan Human | High (predicted) | GPR39 |
| TargetScan Human | High (predicted) | GRB10 |
| TargetScan Human | High (predicted) | GRHL1 |
| TargetScan Human | High (predicted) | GRIN2A |
| TargetScan Human | High (predicted) | GRIP2 |
| TargetScan Human | High (predicted) | GRSF1 |
| TargetScan Human | High (predicted) | GTPBP2 |
| TargetScan Human | High (predicted) | HAPLN1 |
| TargetScan Human | High (predicted) | HCN1 |
| TargetScan Human | High (predicted) | HCN3 |
| TargetScan Human | High (predicted) | HCN4 |
| TargetScan Human | High (predicted) | HDDC3 |
| TargetScan Human | High (predicted) | HIC2 |
| TargetScan Human | High (predicted) | HIF1AN |
| TargetScan Human | High (predicted) | HINFP |
| TargetScan Human | High (predicted) | HMGB3 |
| TargetScan Human | High (predicted) | HNRNPUL2 |
| TargetScan Human | High (predicted) | HOMEZ |
| TargetScan Human | High (predicted) | HOXB3 |
| TargetScan Human | High (predicted) | HOXD1 |
| TargetScan Human | High (predicted) | HOXD9 |
| TargetScan Human | High (predicted) | HTATIP2 |
| TargetScan Human | High (predicted) | IBA57 |
| TargetScan Human | High (predicted) | IER2 |
| TargetScan Human | High (predicted) | IER3IP1 |
| TargetScan Human | High (predicted) | IFFO1 |
| TargetScan Human | High (predicted) | IGSF11 |
| TargetScan Human | High (predicted) | IL16 |
| TargetScan Human | High (predicted) | IL31 |
| TargetScan Human | High (predicted) | IL6R |
| TargetScan Human | High (predicted) | IMPAD1 |
| TargetScan Human | High (predicted) | INO80D |
| TargetScan Human | High (predicted) | INTS7 |
| TargetScan Human | High (predicted) | IP6K1 |
| TargetScan Human | High (predicted) | IPMK |
| TargetScan Human | High (predicted) | IQSEC2 |
| TargetScan Human | High (predicted) | IRF4 |
| TargetScan Human | High (predicted) | IST1 |
| TargetScan Human | High (predicted) | ITGA9 |
| TargetScan Human | High (predicted) | ITGB3 |
| TargetScan Human | High (predicted) | JADE2 |
| TargetScan Human | High (predicted) | JMY |
| TargetScan Human | High (predicted) | KCNA1 |
| TargetScan Human | High (predicted) | KCNH1 |
| TargetScan Human | High (predicted) | KCNH3 |
| TargetScan Human | High (predicted) | KCNH4 |
| TargetScan Human | High (predicted) | KCNH7 |
| TargetScan Human | High (predicted) | KCNIP3 |
| TargetScan Human | High (predicted) | KCNJ12 |
| TargetScan Human | High (predicted) | KCNK10 |
| TargetScan Human | High (predicted) | KCNS3 |
| TargetScan Human | High (predicted) | KCTD15 |
| TargetScan Human | High (predicted) | KCTD21 |
| TargetScan Human | High (predicted) | KDM4B |
| TargetScan Human | High (predicted) | KHNYN |
| TargetScan Human | High (predicted) | KIAA0319L |
| TargetScan Human | High (predicted) | KIAA0556 |
| TargetScan Human | High (predicted) | KIAA1522 |
| TargetScan Human | High (predicted) | KIAA1644 |
| TargetScan Human | High (predicted) | KIAA1841 |
| TargetScan Human | High (predicted) | KLC2 |
| TargetScan Human | High (predicted) | KLF16 |
| TargetScan Human | High (predicted) | KLF3 |
| TargetScan Human | High (predicted) | KLHL24 |
| TargetScan Human | High (predicted) | KLHL31 |
| TargetScan Human | High (predicted) | KLHL6 |
| TargetScan Human | High (predicted) | KLRC2 |
| TargetScan Human | High (predicted) | KMT5C |
| TargetScan Human | High (predicted) | KNOP1 |
| TargetScan Human | High (predicted) | KPNA6 |
| TargetScan Human | High (predicted) | KRT85 |
| TargetScan Human | High (predicted) | KSR2 |
| TargetScan Human | High (predicted) | LACTB |
| TargetScan Human | High (predicted) | LAMA4 |
| TargetScan Human | High (predicted) | LBH |
| TargetScan Human | High (predicted) | LCLAT1 |
| TargetScan Human | High (predicted) | LCOR |
| TargetScan Human | High (predicted) | LCORL |
| TargetScan Human | High (predicted) | LDAH |
| TargetScan Human | High (predicted) | LFNG |
| TargetScan Human | High (predicted) | LGI2 |
| TargetScan Human | High (predicted) | LIF |
| TargetScan Human | High (predicted) | LIFR |
| TargetScan Human | High (predicted) | LIMD2 |
| TargetScan Human | High (predicted) | LIMK1 |
| TargetScan Human | High (predicted) | LIN28B |
| TargetScan Human | High (predicted) | LNPEP |
| TargetScan Human | High (predicted) | LONRF2 |
| TargetScan Human | High (predicted) | LOXL1 |
| TargetScan Human | High (predicted) | LPCAT4 |
| TargetScan Human | High (predicted) | LPP |
| TargetScan Human | High (predicted) | LRFN2 |
| TargetScan Human | High (predicted) | LRP4 |
| TargetScan Human | High (predicted) | LRRC10B |
| TargetScan Human | High (predicted) | LRRC25 |
| TargetScan Human | High (predicted) | LRRC8A |
| TargetScan Human | High (predicted) | LRRC8B |
| TargetScan Human | High (predicted) | LRTOMT |
| TargetScan Human | High (predicted) | LSM8 |
| TargetScan Human | High (predicted) | LURAP1L |
| TargetScan Human | High (predicted) | LUZP1 |
| TargetScan Human | High (predicted) | LVRN |
| TargetScan Human | High (predicted) | LYPD6 |
| TargetScan Human | High (predicted) | LYPLA2 |
| TargetScan Human | High (predicted) | LYZL6 |
| TargetScan Human | High (predicted) | M6PR |
| TargetScan Human | High (predicted) | MAF |
| TargetScan Human | High (predicted) | MAMDC2 |
| TargetScan Human | High (predicted) | MAN1B1 |
| TargetScan Human | High (predicted) | MAP3K1 |
| TargetScan Human | High (predicted) | MAP3K10 |
| TargetScan Human | High (predicted) | MAP3K11 |
| TargetScan Human | High (predicted) | MAP3K13 |
| TargetScan Human | High (predicted) | MAP3K3 |
| TargetScan Human | High (predicted) | MAPK12 |
| TargetScan Human | High (predicted) | MAPK14 |
| TargetScan Human | High (predicted) | MAPK1IP1L |
| TargetScan Human | High (predicted) | MAPKAPK2 |
| TargetScan Human | High (predicted) | MAPRE2 |
| TargetScan Human | High (predicted) | MARK2 |
| TargetScan Human | High (predicted) | MASP1 |
| TargetScan Human | High (predicted) | MAVS |
| TargetScan Human | High (predicted) | MBD1 |
| TargetScan Human | High (predicted) | MBNL3 |
| TargetScan Human | High (predicted) | MCL1 |
| TargetScan Human | High (predicted) | MED15 |
| TargetScan Human | High (predicted) | MEF2D |
| TargetScan Human | High (predicted) | MEGF8 |
| TargetScan Human | High (predicted) | MEGF9 |
| TargetScan Human | High (predicted) | MEMO1 |
| TargetScan Human | High (predicted) | MFHAS1 |
| TargetScan Human | High (predicted) | MFN1 |
| TargetScan Human | High (predicted) | MFSD13A |
| TargetScan Human | High (predicted) | MFSD14B |
| TargetScan Human | High (predicted) | MGAT4A |
| TargetScan Human | High (predicted) | MIB1 |
| TargetScan Human | High (predicted) | MICALL1 |
| TargetScan Human | High (predicted) | MKNK2 |
| TargetScan Human | High (predicted) | MLF2 |
| TargetScan Human | High (predicted) | MMP11 |
| TargetScan Human | High (predicted) | MOCS1 |
| TargetScan Human | High (predicted) | MRAP |
| TargetScan Human | High (predicted) | MS4A3 |
| TargetScan Human | High (predicted) | MSI1 |
| TargetScan Human | High (predicted) | MSL1 |
| TargetScan Human | High (predicted) | MSRB3 |
| TargetScan Human | High (predicted) | MTF1 |
| TargetScan Human | High (predicted) | MTFP1 |
| TargetScan Human | High (predicted) | MTMR3 |
| TargetScan Human | High (predicted) | MTUS1 |
| TargetScan Human | High (predicted) | MTUS2 |
| TargetScan Human | High (predicted) | MUL1 |
| TargetScan Human | High (predicted) | MXD4 |
| TargetScan Human | High (predicted) | MXRA8 |
| TargetScan Human | High (predicted) | MYO18A |
| TargetScan Human | High (predicted) | MYO7A |
| TargetScan Human | High (predicted) | MYT1 |
| TargetScan Human | High (predicted) | NAA25 |
| TargetScan Human | High (predicted) | NAA40 |
| TargetScan Human | High (predicted) | NAGA |
| TargetScan Human | High (predicted) | NAIF1 |
| TargetScan Human | High (predicted) | NATD1 |
| TargetScan Human | High (predicted) | NBEAL2 |
| TargetScan Human | High (predicted) | NCAN |
| TargetScan Human | High (predicted) | NCKAP5L |
| TargetScan Human | High (predicted) | NCLN |
| TargetScan Human | High (predicted) | NCOR2 |
| TargetScan Human | High (predicted) | NDRG3 |
| TargetScan Human | High (predicted) | NECAB3 |
| TargetScan Human | High (predicted) | NEDD9 |
| TargetScan Human | High (predicted) | NEMP1 |
| TargetScan Human | High (predicted) | NEO1 |
| TargetScan Human | High (predicted) | NEU1 |
| TargetScan Human | High (predicted) | NFIB |
| TargetScan Human | High (predicted) | NIN |
| TargetScan Human | High (predicted) | NIP7 |
| TargetScan Human | High (predicted) | NIPA1 |
| TargetScan Human | High (predicted) | NIPAL4 |
| TargetScan Human | High (predicted) | NKIRAS2 |
| TargetScan Human | High (predicted) | NKPD1 |
| TargetScan Human | High (predicted) | NLRP1 |
| TargetScan Human | High (predicted) | NOS1AP |
| TargetScan Human | High (predicted) | NPL |
| TargetScan Human | High (predicted) | NRG3 |
| TargetScan Human | High (predicted) | NRM |
| TargetScan Human | High (predicted) | NRXN1 |
| TargetScan Human | High (predicted) | NT5DC1 |
| TargetScan Human | High (predicted) | NUMBL |
| TargetScan Human | High (predicted) | NUP210 |
| TargetScan Human | High (predicted) | NUP50 |
| TargetScan Human | High (predicted) | NXF1 |
| TargetScan Human | High (predicted) | OAZ2 |
| TargetScan Human | High (predicted) | OGFR |
| TargetScan Human | High (predicted) | OLFML2A |
| TargetScan Human | High (predicted) | ONECUT2 |
| TargetScan Human | High (predicted) | OPALIN |
| TargetScan Human | High (predicted) | ORC2 |
| TargetScan Human | High (predicted) | OSBPL9 |
| TargetScan Human | High (predicted) | OTUB2 |
| TargetScan Human | High (predicted) | OVOL1 |
| TargetScan Human | High (predicted) | P2RY8 |
| TargetScan Human | High (predicted) | PACS2 |
| TargetScan Human | High (predicted) | PAFAH1B1 |
| TargetScan Human | High (predicted) | PAPOLA |
| TargetScan Human | High (predicted) | PAPOLB |
| TargetScan Human | High (predicted) | PARM1 |
| TargetScan Human | High (predicted) | PARP14 |
| TargetScan Human | High (predicted) | PARVG |
| TargetScan Human | High (predicted) | PCGF6 |
| TargetScan Human | High (predicted) | PCNX |
| TargetScan Human | High (predicted) | PCSK7 |
| TargetScan Human | High (predicted) | PDE7A |
| TargetScan Human | High (predicted) | PDK3 |
| TargetScan Human | High (predicted) | PDPR |
| TargetScan Human | High (predicted) | PDZD3 |
| TargetScan Human | High (predicted) | PEAK1 |
| TargetScan Human | High (predicted) | PELI2 |
| TargetScan Human | High (predicted) | PGAP3 |
| TargetScan Human | High (predicted) | PGP |
| TargetScan Human | High (predicted) | PHC2 |
| TargetScan Human | High (predicted) | PHF20 |
| TargetScan Human | High (predicted) | PHF23 |
| TargetScan Human | High (predicted) | PHOX2B |
| TargetScan Human | High (predicted) | PHYHIP |
| TargetScan Human | High (predicted) | PI4K2B |
| TargetScan Human | High (predicted) | PIK3C2B |
| TargetScan Human | High (predicted) | PIK3CD |
| TargetScan Human | High (predicted) | PIK3R5 |
| TargetScan Human | High (predicted) | PIP4K2B |
| TargetScan Human | High (predicted) | PIP5K1C |
| TargetScan Human | High (predicted) | PIP5KL1 |
| TargetScan Human | High (predicted) | PLAGL1 |
| TargetScan Human | High (predicted) | PLAGL2 |
| TargetScan Human | High (predicted) | PLEKHA8 |
| TargetScan Human | High (predicted) | PLEKHM3 |
| TargetScan Human | High (predicted) | PLXNA1 |
| TargetScan Human | High (predicted) | PMM2 |
| TargetScan Human | High (predicted) | PODXL |
| TargetScan Human | High (predicted) | POU2F1 |
| TargetScan Human | High (predicted) | POU2F2 |
| TargetScan Human | High (predicted) | PPARA |
| TargetScan Human | High (predicted) | PPAT |
| TargetScan Human | High (predicted) | PPM1A |
| TargetScan Human | High (predicted) | PPM1H |
| TargetScan Human | High (predicted) | PPME1 |
| TargetScan Human | High (predicted) | PPP1CA |
| TargetScan Human | High (predicted) | PPP1R37 |
| TargetScan Human | High (predicted) | PPP1R9B |
| TargetScan Human | High (predicted) | PPP2CA |
| TargetScan Human | High (predicted) | PPP2R4 |
| TargetScan Human | High (predicted) | PPP2R5C |
| TargetScan Human | High (predicted) | PPT1 |
| TargetScan Human | High (predicted) | PRDM1 |
| TargetScan Human | High (predicted) | PRELID2 |
| TargetScan Human | High (predicted) | PRKAA2 |
| TargetScan Human | High (predicted) | PROX1 |
| TargetScan Human | High (predicted) | PRRC1 |
| TargetScan Human | High (predicted) | PRSS35 |
| TargetScan Human | High (predicted) | PRTG |
| TargetScan Human | High (predicted) | PSMB8 |
| TargetScan Human | High (predicted) | PSMD7 |
| TargetScan Human | High (predicted) | PSMD9 |
| TargetScan Human | High (predicted) | PSTPIP2 |
| TargetScan Human | High (predicted) | PTAR1 |
| TargetScan Human | High (predicted) | PTH1R |
| TargetScan Human | High (predicted) | PTPN18 |
| TargetScan Human | High (predicted) | PVRL2 |
| TargetScan Human | High (predicted) | QKI |
| TargetScan Human | High (predicted) | QSOX2 |
| TargetScan Human | High (predicted) | RAB22A |
| TargetScan Human | High (predicted) | RAB3D |
| TargetScan Human | High (predicted) | RAB3IP |
| TargetScan Human | High (predicted) | RAB6B |
| TargetScan Human | High (predicted) | RAB8B |
| TargetScan Human | High (predicted) | RABEP2 |
| TargetScan Human | High (predicted) | RAF1 |
| TargetScan Human | High (predicted) | RALGPS2 |
| TargetScan Human | High (predicted) | RAP1GAP2 |
| TargetScan Human | High (predicted) | RAPGEF5 |
| TargetScan Human | High (predicted) | RAPGEFL1 |
| TargetScan Human | High (predicted) | RASAL2 |
| TargetScan Human | High (predicted) | RASGEF1A |
| TargetScan Human | High (predicted) | RASGRF1 |
| TargetScan Human | High (predicted) | RASGRF2 |
| TargetScan Human | High (predicted) | RASL10B |
| TargetScan Human | High (predicted) | RASSF3 |
| TargetScan Human | High (predicted) | RBAK |
| TargetScan Human | High (predicted) | RBCK1 |
| TargetScan Human | High (predicted) | RBFOX2 |
| TargetScan Human | High (predicted) | RBM20 |
| TargetScan Human | High (predicted) | RBM24 |
| TargetScan Human | High (predicted) | RBM38 |
| TargetScan Human | High (predicted) | RBM7 |
| TargetScan Human | High (predicted) | REEP3 |
| TargetScan Human | High (predicted) | REST |
| TargetScan Human | High (predicted) | RET |
| TargetScan Human | High (predicted) | RFX5 |
| TargetScan Human | High (predicted) | RFXANK |
| TargetScan Human | High (predicted) | RGS6 |
| TargetScan Human | High (predicted) | RHOBTB2 |
| TargetScan Human | High (predicted) | RHOQ |
| TargetScan Human | High (predicted) | RHOT2 |
| TargetScan Human | High (predicted) | RIMKLA |
| TargetScan Human | High (predicted) | RIN3 |
| TargetScan Human | High (predicted) | RIT1 |
| TargetScan Human | High (predicted) | RMND5A |
| TargetScan Human | High (predicted) | RND2 |
| TargetScan Human | High (predicted) | RNF121 |
| TargetScan Human | High (predicted) | RNF144A |
| TargetScan Human | High (predicted) | RNF144B |
| TargetScan Human | High (predicted) | RNF168 |
| TargetScan Human | High (predicted) | RNF222 |
| TargetScan Human | High (predicted) | RNF40 |
| TargetScan Human | High (predicted) | RNF44 |
| TargetScan Human | High (predicted) | RORA |
| TargetScan Human | High (predicted) | RPS6KA1 |
| TargetScan Human | High (predicted) | RREB1 |
| TargetScan Human | High (predicted) | RS1 |
| TargetScan Human | High (predicted) | RTP1 |
| TargetScan Human | High (predicted) | RUFY3 |
| TargetScan Human | High (predicted) | RUSC2 |
| TargetScan Human | High (predicted) | RYBP |
| TargetScan Human | High (predicted) | SAA2-SAA4 |
| TargetScan Human | High (predicted) | SAA4 |
| TargetScan Human | High (predicted) | SAMD10 |
| TargetScan Human | High (predicted) | SAR1B |
| TargetScan Human | High (predicted) | SARM1 |
| TargetScan Human | High (predicted) | SASH1 |
| TargetScan Human | High (predicted) | SBNO1 |
| TargetScan Human | High (predicted) | SCARA5 |
| TargetScan Human | High (predicted) | SCARB1 |
| TargetScan Human | High (predicted) | SCARB2 |
| TargetScan Human | High (predicted) | SCARF2 |
| TargetScan Human | High (predicted) | SCN2B |
| TargetScan Human | High (predicted) | SCN4A |
| TargetScan Human | High (predicted) | SCN4B |
| TargetScan Human | High (predicted) | SCN5A |
| TargetScan Human | High (predicted) | SCRT1 |
| TargetScan Human | High (predicted) | SCRT2 |
| TargetScan Human | High (predicted) | SEC61A2 |
| TargetScan Human | High (predicted) | SEL1L |
| TargetScan Human | High (predicted) | SEMA4B |
| TargetScan Human | High (predicted) | SEMA4C |
| TargetScan Human | High (predicted) | SEMA4D |
| TargetScan Human | High (predicted) | SEMA4F |
| TargetScan Human | High (predicted) | SEPN1 |
| TargetScan Human | High (predicted) | SEPT11 |
| TargetScan Human | High (predicted) | SERTAD3 |
| TargetScan Human | High (predicted) | SESTD1 |
| TargetScan Human | High (predicted) | SETD7 |
| TargetScan Human | High (predicted) | SFXN2 |
| TargetScan Human | High (predicted) | SGSM2 |
| TargetScan Human | High (predicted) | SH2B3 |
| TargetScan Human | High (predicted) | SH3BP4 |
| TargetScan Human | High (predicted) | SH3BP5L |
| TargetScan Human | High (predicted) | SH3TC2 |
| TargetScan Human | High (predicted) | SHISA6 |
| TargetScan Human | High (predicted) | SHTN1 |
| TargetScan Human | High (predicted) | SIRT7 |
| TargetScan Human | High (predicted) | SLA2 |
| TargetScan Human | High (predicted) | SLC16A6 |
| TargetScan Human | High (predicted) | SLC17A7 |
| TargetScan Human | High (predicted) | SLC18A3 |
| TargetScan Human | High (predicted) | SLC19A1 |
| TargetScan Human | High (predicted) | SLC23A2 |
| TargetScan Human | High (predicted) | SLC24A2 |
| TargetScan Human | High (predicted) | SLC25A15 |
| TargetScan Human | High (predicted) | SLC25A53 |
| TargetScan Human | High (predicted) | SLC26A6 |
| TargetScan Human | High (predicted) | SLC27A4 |
| TargetScan Human | High (predicted) | SLC31A1 |
| TargetScan Human | High (predicted) | SLC35A4 |
| TargetScan Human | High (predicted) | SLC35C1 |
| TargetScan Human | High (predicted) | SLC37A2 |
| TargetScan Human | High (predicted) | SLC38A9 |
| TargetScan Human | High (predicted) | SLC39A13 |
| TargetScan Human | High (predicted) | SLC39A9 |
| TargetScan Human | High (predicted) | SLC46A3 |
| TargetScan Human | High (predicted) | SLC4A10 |
| TargetScan Human | High (predicted) | SLC4A4 |
| TargetScan Human | High (predicted) | SLC4A8 |
| TargetScan Human | High (predicted) | SLC6A15 |
| TargetScan Human | High (predicted) | SLC6A17 |
| TargetScan Human | High (predicted) | SLC7A1 |
| TargetScan Human | High (predicted) | SLC7A6 |
| TargetScan Human | High (predicted) | SLC8A2 |
| TargetScan Human | High (predicted) | SLCO2A1 |
| TargetScan Human | High (predicted) | SLCO2B1 |
| TargetScan Human | High (predicted) | SLITRK6 |
| TargetScan Human | High (predicted) | SMAD2 |
| TargetScan Human | High (predicted) | SMAD4 |
| TargetScan Human | High (predicted) | SMARCD2 |
| TargetScan Human | High (predicted) | SMCR8 |
| TargetScan Human | High (predicted) | SMG1 |
| TargetScan Human | High (predicted) | SMG5 |
| TargetScan Human | High (predicted) | SMURF1 |
| TargetScan Human | High (predicted) | SNAP29 |
| TargetScan Human | High (predicted) | SNTB2 |
| TargetScan Human | High (predicted) | SNX18 |
| TargetScan Human | High (predicted) | SNX27 |
| TargetScan Human | High (predicted) | SOCS4 |
| TargetScan Human | High (predicted) | SORD |
| TargetScan Human | High (predicted) | SORT1 |
| TargetScan Human | High (predicted) | SOX11 |
| TargetScan Human | High (predicted) | SP1 |
| TargetScan Human | High (predicted) | SP7 |
| TargetScan Human | High (predicted) | SPATA31D1 (includes others) |
| TargetScan Human | High (predicted) | SPATA33 |
| TargetScan Human | High (predicted) | SPEG |
| TargetScan Human | High (predicted) | SPSB1 |
| TargetScan Human | High (predicted) | SPSB4 |
| TargetScan Human | High (predicted) | SPTB |
| TargetScan Human | High (predicted) | SRF |
| TargetScan Human | High (predicted) | SRGAP2 |
| TargetScan Human | High (predicted) | SRPRB |
| TargetScan Human | High (predicted) | SRRM3 |
| TargetScan Human | High (predicted) | SRSF6 |
| TargetScan Human | High (predicted) | SSTR3 |
| TargetScan Human | High (predicted) | ST6GAL1 |
| TargetScan Human | High (predicted) | ST6GALNAC6 |
| TargetScan Human | High (predicted) | ST8SIA4 |
| TargetScan Human | High (predicted) | STARD13 |
| TargetScan Human | High (predicted) | STAT3 |
| TargetScan Human | High (predicted) | STMN3 |
| TargetScan Human | High (predicted) | STX18 |
| TargetScan Human | High (predicted) | STX6 |
| TargetScan Human | High (predicted) | STXBP5L |
| TargetScan Human | High (predicted) | SULT4A1 |
| TargetScan Human | High (predicted) | SUN1 |
| TargetScan Human | High (predicted) | SUSD6 |
| TargetScan Human | High (predicted) | SUV39H1 |
| TargetScan Human | High (predicted) | SVIP |
| TargetScan Human | High (predicted) | SWSAP1 |
| TargetScan Human | High (predicted) | SYDE2 |
| TargetScan Human | High (predicted) | SYN2 |
| TargetScan Human | High (predicted) | SYT2 |
| TargetScan Human | High (predicted) | SYVN1 |
| TargetScan Human | High (predicted) | SZRD1 |
| TargetScan Human | High (predicted) | TACC2 |
| TargetScan Human | High (predicted) | TADA2B |
| TargetScan Human | High (predicted) | TAF5L |
| TargetScan Human | High (predicted) | TAF9B |
| TargetScan Human | High (predicted) | TAOK1 |
| TargetScan Human | High (predicted) | TAP2 |
| TargetScan Human | High (predicted) | TAT |
| TargetScan Human | High (predicted) | TAZ |
| TargetScan Human | High (predicted) | TBC1D1 |
| TargetScan Human | High (predicted) | TBC1D8B |
| TargetScan Human | High (predicted) | TBX4 |
| TargetScan Human | High (predicted) | TCTA |
| TargetScan Human | High (predicted) | TDG |
| TargetScan Human | High (predicted) | TEF |
| TargetScan Human | High (predicted) | TET2 |
| TargetScan Human | High (predicted) | TGOLN2 |
| TargetScan Human | High (predicted) | THEMIS2 |
| TargetScan Human | High (predicted) | THY1 |
| TargetScan Human | High (predicted) | TIMM17B |
| TargetScan Human | High (predicted) | TLE3 |
| TargetScan Human | High (predicted) | TLK2 |
| TargetScan Human | High (predicted) | TMBIM6 |
| TargetScan Human | High (predicted) | TMCC2 |
| TargetScan Human | High (predicted) | TMED9 |
| TargetScan Human | High (predicted) | TMEM101 |
| TargetScan Human | High (predicted) | TMEM120B |
| TargetScan Human | High (predicted) | TMEM123 |
| TargetScan Human | High (predicted) | TMEM135 |
| TargetScan Human | High (predicted) | TMEM136 |
| TargetScan Human | High (predicted) | TMEM161B |
| TargetScan Human | High (predicted) | TMEM168 |
| TargetScan Human | High (predicted) | TMEM170B |
| TargetScan Human | High (predicted) | TMEM174 |
| TargetScan Human | High (predicted) | TMEM177 |
| TargetScan Human | High (predicted) | TMEM19 |
| TargetScan Human | High (predicted) | TMEM198 |
| TargetScan Human | High (predicted) | TMEM201 |
| TargetScan Human | High (predicted) | TMEM229B |
| TargetScan Human | High (predicted) | TMEM26 |
| TargetScan Human | High (predicted) | TMEM63A |
| TargetScan Human | High (predicted) | TMEM86A |
| TargetScan Human | High (predicted) | TMEM87B |
| TargetScan Human | High (predicted) | TMLHE |
| TargetScan Human | High (predicted) | TMPRSS13 |
| TargetScan Human | High (predicted) | TNFAIP3 |
| TargetScan Human | High (predicted) | TNFRSF1B |
| TargetScan Human | High (predicted) | TNFSF4 |
| TargetScan Human | High (predicted) | TOMM40 |
| TargetScan Human | High (predicted) | TP53INP1 |
| TargetScan Human | High (predicted) | TRAF6 |
| TargetScan Human | High (predicted) | TRAPPC6B |
| TargetScan Human | High (predicted) | TRIAP1 |
| TargetScan Human | High (predicted) | TRIB2 |
| TargetScan Human | High (predicted) | TRIL |
| TargetScan Human | High (predicted) | TRIM7 |
| TargetScan Human | High (predicted) | TRIM71 |
| TargetScan Human | High (predicted) | TRMT5 |
| TargetScan Human | High (predicted) | TRPS1 |
| TargetScan Human | High (predicted) | TSEN54 |
| TargetScan Human | High (predicted) | TSHZ2 |
| TargetScan Human | High (predicted) | TSPAN12 |
| TargetScan Human | High (predicted) | TSPAN14 |
| TargetScan Human | High (predicted) | TSTA3 |
| TargetScan Human | High (predicted) | TSTD2 |
| TargetScan Human | High (predicted) | TTC7A |
| TargetScan Human | High (predicted) | TTPA |
| TargetScan Human | High (predicted) | TXNRD1 |
| TargetScan Human | High (predicted) | TXNRD3 |
| TargetScan Human | High (predicted) | TYSND1 |
| TargetScan Human | High (predicted) | UBASH3B |
| TargetScan Human | High (predicted) | UBE2G1 |
| TargetScan Human | High (predicted) | UBE2J1 |
| TargetScan Human | High (predicted) | UBE2L3 |
| TargetScan Human | High (predicted) | UBE2R2 |
| TargetScan Human | High (predicted) | UBE2W |
| TargetScan Human | High (predicted) | UBL4B |
| TargetScan Human | High (predicted) | UBN1 |
| TargetScan Human | High (predicted) | UBR2 |
| TargetScan Human | High (predicted) | UBR7 |
| TargetScan Human | High (predicted) | UBTD1 |
| TargetScan Human | High (predicted) | UCK2 |
| TargetScan Human | High (predicted) | ULK3 |
| TargetScan Human | High (predicted) | UNC5C |
| TargetScan Human | High (predicted) | USP12 |
| TargetScan Human | High (predicted) | USP2 |
| TargetScan Human | High (predicted) | USP37 |
| TargetScan Human | High (predicted) | USP38 |
| TargetScan Human | High (predicted) | USP45 |
| TargetScan Human | High (predicted) | USP46 |
| TargetScan Human | High (predicted) | USP8 |
| TargetScan Human | High (predicted) | VANGL1 |
| TargetScan Human | High (predicted) | VANGL2 |
| TargetScan Human | High (predicted) | VAV3 |
| TargetScan Human | High (predicted) | VAX1 |
| TargetScan Human | High (predicted) | VCPIP1 |
| TargetScan Human | High (predicted) | VDR |
| TargetScan Human | High (predicted) | VEGFA |
| TargetScan Human | High (predicted) | VPS36 |
| TargetScan Human | High (predicted) | VPS37B |
| TargetScan Human | High (predicted) | VPS37C |
| TargetScan Human | High (predicted) | VPS4B |
| TargetScan Human | High (predicted) | VTCN1 |
| TargetScan Human | High (predicted) | WARS |
| TargetScan Human | High (predicted) | WDR1 |
| TargetScan Human | High (predicted) | WIPF2 |
| TargetScan Human | High (predicted) | WIZ |
| TargetScan Human | High (predicted) | XKRX |
| TargetScan Human | High (predicted) | YES1 |
| TargetScan Human | High (predicted) | YIPF6 |
| TargetScan Human | High (predicted) | YOD1 |
| TargetScan Human | High (predicted) | YWHAG |
| TargetScan Human | High (predicted) | ZBTB33 |
| TargetScan Human | High (predicted) | ZBTB34 |
| TargetScan Human | High (predicted) | ZBTB37 |
| TargetScan Human | High (predicted) | ZBTB38 |
| TargetScan Human | High (predicted) | ZBTB43 |
| TargetScan Human | High (predicted) | ZBTB47 |
| TargetScan Human | High (predicted) | ZBTB7A |
| TargetScan Human | High (predicted) | ZC3H12B |
| TargetScan Human | High (predicted) | ZC3H7B |
| TargetScan Human | High (predicted) | ZCCHC8 |
| TargetScan Human | High (predicted) | ZDHHC7 |
| TargetScan Human | High (predicted) | ZDHHC9 |
| TargetScan Human | High (predicted) | ZFP62 |
| TargetScan Human | High (predicted) | ZFYVE1 |
| TargetScan Human | High (predicted) | ZKSCAN5 |
| TargetScan Human | High (predicted) | ZMYM2 |
| TargetScan Human | High (predicted) | ZNF12 |
| TargetScan Human | High (predicted) | ZNF148 |
| TargetScan Human | High (predicted) | ZNF236 |
| TargetScan Human | High (predicted) | ZNF281 |
| TargetScan Human | High (predicted) | ZNF304 |
| TargetScan Human | High (predicted) | ZNF343 |
| TargetScan Human | High (predicted) | ZNF395 |
| TargetScan Human | High (predicted) | ZNF460 |
| TargetScan Human | High (predicted) | ZNF512B |
| TargetScan Human | High (predicted) | ZNF518A |
| TargetScan Human | High (predicted) | ZNF543 |
| TargetScan Human | High (predicted) | ZNF546 |
| TargetScan Human | High (predicted) | ZNF618 |
| TargetScan Human | High (predicted) | ZNF624 |
| TargetScan Human | High (predicted) | ZNF652 |
| TargetScan Human | High (predicted) | ZNF664 |
| TargetScan Human | High (predicted) | ZNF691 |
| TargetScan Human | High (predicted) | ZNF704 |
| TargetScan Human | High (predicted) | ZNF707 |
| TargetScan Human | High (predicted) | ZNF76 |
| TargetScan Human | High (predicted) | ZNF792 |
| TargetScan Human | High (predicted) | ZNRF3 |
| TargetScan Human | High (predicted) | ZSCAN22 |
| TargetScan Human | High (predicted) | ZSCAN29 |
| TargetScan Human | High (predicted) | ZSWIM4 |
| TargetScan Human | High (predicted) | ZSWIM5 |
| TargetScan Human | High (predicted) | ZSWIM6 |
| TargetScan Human | High (predicted),Moderate (predicted) | METTL15 |
| TargetScan Human | High (predicted),Moderate (predicted) | NBPF15 (includes others) |
| TargetScan Human | High (predicted),Moderate (predicted) | POFUT2 |
| TargetScan Human | Moderate (predicted) | AARS2 |
| TargetScan Human | Moderate (predicted) | ABAT |
| TargetScan Human | Moderate (predicted) | ABCC5 |
| TargetScan Human | Moderate (predicted) | ABLIM3 |
| TargetScan Human | Moderate (predicted) | ACADS |
| TargetScan Human | Moderate (predicted) | ACCSL |
| TargetScan Human | Moderate (predicted) | ACPP |
| TargetScan Human | Moderate (predicted) | ACSBG2 |
| TargetScan Human | Moderate (predicted) | ACTR10 |
| TargetScan Human | Moderate (predicted) | ACTR8 |
| TargetScan Human | Moderate (predicted) | ADAT1 |
| TargetScan Human | Moderate (predicted) | ADCK1 |
| TargetScan Human | Moderate (predicted) | ADD2 |
| TargetScan Human | Moderate (predicted) | ADH1B |
| TargetScan Human | Moderate (predicted) | AEN |
| TargetScan Human | Moderate (predicted) | AGTRAP |
| TargetScan Human | Moderate (predicted) | AKAP6 |
| TargetScan Human | Moderate (predicted) | ALDH4A1 |
| TargetScan Human | Moderate (predicted) | ALG1 |
| TargetScan Human | Moderate (predicted) | ALG10B |
| TargetScan Human | Moderate (predicted) | ALG12 |
| TargetScan Human | Moderate (predicted) | ALG13 |
| TargetScan Human | Moderate (predicted) | AMBRA1 |
| TargetScan Human | Moderate (predicted) | ANAPC15 |
| TargetScan Human | Moderate (predicted) | ANKMY1 |
| TargetScan Human | Moderate (predicted) | ANKRD26 |
| TargetScan Human | Moderate (predicted) | ANKRD35 |
| TargetScan Human | Moderate (predicted) | ANKS6 |
| TargetScan Human | Moderate (predicted) | ANO3 |
| TargetScan Human | Moderate (predicted) | APOBEC3F |
| TargetScan Human | Moderate (predicted) | APOBEC4 |
| TargetScan Human | Moderate (predicted) | APOL4 |
| TargetScan Human | Moderate (predicted) | APOL6 |
| TargetScan Human | Moderate (predicted) | APPL1 |
| TargetScan Human | Moderate (predicted) | AQP6 |
| TargetScan Human | Moderate (predicted) | ARHGAP27 |
| TargetScan Human | Moderate (predicted) | ARHGEF18 |
| TargetScan Human | Moderate (predicted) | ARHGEF2 |
| TargetScan Human | Moderate (predicted) | ARMT1 |
| TargetScan Human | Moderate (predicted) | ARPIN/C15orf38-AP3S2 |
| TargetScan Human | Moderate (predicted) | ARSD |
| TargetScan Human | Moderate (predicted) | ASB11 |
| TargetScan Human | Moderate (predicted) | ASRGL1 |
| TargetScan Human | Moderate (predicted) | ASTN2 |
| TargetScan Human | Moderate (predicted) | ATHL1 |
| TargetScan Human | Moderate (predicted) | ATL2 |
| TargetScan Human | Moderate (predicted) | AZI2 |
| TargetScan Human | Moderate (predicted) | B3GLCT |
| TargetScan Human | Moderate (predicted) | B4GALNT1 |
| TargetScan Human | Moderate (predicted) | B4GAT1 |
| TargetScan Human | Moderate (predicted) | BAIAP2L1 |
| TargetScan Human | Moderate (predicted) | BATF2 |
| TargetScan Human | Moderate (predicted) | BCKDK |
| TargetScan Human | Moderate (predicted) | BCL2L13 |
| TargetScan Human | Moderate (predicted) | BFAR |
| TargetScan Human | Moderate (predicted) | BHLHB9 |
| TargetScan Human | Moderate (predicted) | BIN3 |
| TargetScan Human | Moderate (predicted) | BLOC1S3 |
| TargetScan Human | Moderate (predicted) | BRCC3 |
| TargetScan Human | Moderate (predicted) | BRMS1 |
| TargetScan Human | Moderate (predicted) | BRPF3 |
| TargetScan Human | Moderate (predicted) | BSG |
| TargetScan Human | Moderate (predicted) | BTBD9 |
| TargetScan Human | Moderate (predicted) | BTG2 |
| TargetScan Human | Moderate (predicted) | C16orf62 |
| TargetScan Human | Moderate (predicted) | C17orf50 |
| TargetScan Human | Moderate (predicted) | C17orf96 |
| TargetScan Human | Moderate (predicted) | C1orf109 |
| TargetScan Human | Moderate (predicted) | C1orf116 |
| TargetScan Human | Moderate (predicted) | C20orf203 |
| TargetScan Human | Moderate (predicted) | C3orf62 |
| TargetScan Human | Moderate (predicted) | C3orf80 |
| TargetScan Human | Moderate (predicted) | C4orf26 |
| TargetScan Human | Moderate (predicted) | C6orf223 |
| TargetScan Human | Moderate (predicted) | C7orf26 |
| TargetScan Human | Moderate (predicted) | C8orf33 |
| TargetScan Human | Moderate (predicted) | C9orf139 |
| TargetScan Human | Moderate (predicted) | C9orf170 |
| TargetScan Human | Moderate (predicted) | C9orf47 |
| TargetScan Human | Moderate (predicted) | C9orf9 |
| TargetScan Human | Moderate (predicted) | CA12 |
| TargetScan Human | Moderate (predicted) | CAB39L |
| TargetScan Human | Moderate (predicted) | CALCA |
| TargetScan Human | Moderate (predicted) | CARD8 |
| TargetScan Human | Moderate (predicted) | CARS2 |
| TargetScan Human | Moderate (predicted) | CASKIN2 |
| TargetScan Human | Moderate (predicted) | CCDC120 |
| TargetScan Human | Moderate (predicted) | CCDC126 |
| TargetScan Human | Moderate (predicted) | CCDC134 |
| TargetScan Human | Moderate (predicted) | CCDC88B |
| TargetScan Human | Moderate (predicted) | CCR7 |
| TargetScan Human | Moderate (predicted) | CD244 |
| TargetScan Human | Moderate (predicted) | CD59 |
| TargetScan Human | Moderate (predicted) | CD8A |
| TargetScan Human | Moderate (predicted) | CDC20B |
| TargetScan Human | Moderate (predicted) | CDH12 |
| TargetScan Human | Moderate (predicted) | CDIPT |
| TargetScan Human | Moderate (predicted) | CEMIP |
| TargetScan Human | Moderate (predicted) | CEMP1 |
| TargetScan Human | Moderate (predicted) | CEP68 |
| TargetScan Human | Moderate (predicted) | CERS3 |
| TargetScan Human | Moderate (predicted) | CES3 |
| TargetScan Human | Moderate (predicted) | CHIT1 |
| TargetScan Human | Moderate (predicted) | CHRM5 |
| TargetScan Human | Moderate (predicted) | CHRNA10 |
| TargetScan Human | Moderate (predicted) | CKLF |
| TargetScan Human | Moderate (predicted) | CLEC2B |
| TargetScan Human | Moderate (predicted) | CLN6 |
| TargetScan Human | Moderate (predicted) | CMTM4 |
| TargetScan Human | Moderate (predicted) | CNGA2 |
| TargetScan Human | Moderate (predicted) | CNTNAP1 |
| TargetScan Human | Moderate (predicted) | COX11 |
| TargetScan Human | Moderate (predicted) | COX7A2L |
| TargetScan Human | Moderate (predicted) | CRCP |
| TargetScan Human | Moderate (predicted) | CRTAM |
| TargetScan Human | Moderate (predicted) | CRYAA/LOC102724652 |
| TargetScan Human | Moderate (predicted) | CSF2RB |
| TargetScan Human | Moderate (predicted) | CSRNP1 |
| TargetScan Human | Moderate (predicted) | CST9 |
| TargetScan Human | Moderate (predicted) | CYB561 |
| TargetScan Human | Moderate (predicted) | CYB561D1 |
| TargetScan Human | Moderate (predicted) | CYFIP2 |
| TargetScan Human | Moderate (predicted) | CYP11B2 |
| TargetScan Human | Moderate (predicted) | CYYR1 |
| TargetScan Human | Moderate (predicted) | DCAF4L1 |
| TargetScan Human | Moderate (predicted) | DCLK1 |
| TargetScan Human | Moderate (predicted) | DCLRE1C |
| TargetScan Human | Moderate (predicted) | DCTPP1 |
| TargetScan Human | Moderate (predicted) | DDR1 |
| TargetScan Human | Moderate (predicted) | DGKQ |
| TargetScan Human | Moderate (predicted) | DIDO1 |
| TargetScan Human | Moderate (predicted) | DMC1 |
| TargetScan Human | Moderate (predicted) | DNAJA4 |
| TargetScan Human | Moderate (predicted) | DNAJC19 |
| TargetScan Human | Moderate (predicted) | DOK1 |
| TargetScan Human | Moderate (predicted) | DPM2 |
| TargetScan Human | Moderate (predicted) | DQX1 |
| TargetScan Human | Moderate (predicted) | DRD4 |
| TargetScan Human | Moderate (predicted) | DRP2 |
| TargetScan Human | Moderate (predicted) | DTNA |
| TargetScan Human | Moderate (predicted) | DTWD2 |
| TargetScan Human | Moderate (predicted) | DUOX1 |
| TargetScan Human | Moderate (predicted) | EFCAB2 |
| TargetScan Human | Moderate (predicted) | EFNA2 |
| TargetScan Human | Moderate (predicted) | EHD1 |
| TargetScan Human | Moderate (predicted) | ELK1 |
| TargetScan Human | Moderate (predicted) | EMILIN1 |
| TargetScan Human | Moderate (predicted) | ENDOD1 |
| TargetScan Human | Moderate (predicted) | ENKUR |
| TargetScan Human | Moderate (predicted) | ENTHD2 |
| TargetScan Human | Moderate (predicted) | EPB41 |
| TargetScan Human | Moderate (predicted) | EPHA2 |
| TargetScan Human | Moderate (predicted) | EPHA8 |
| TargetScan Human | Moderate (predicted) | EPM2A |
| TargetScan Human | Moderate (predicted) | ERMN |
| TargetScan Human | Moderate (predicted) | ERMP1 |
| TargetScan Human | Moderate (predicted) | FAAP100 |
| TargetScan Human | Moderate (predicted) | FAM131C |
| TargetScan Human | Moderate (predicted) | FAM155B |
| TargetScan Human | Moderate (predicted) | FAM160B2 |
| TargetScan Human | Moderate (predicted) | FAM178B |
| TargetScan Human | Moderate (predicted) | FAM185A |
| TargetScan Human | Moderate (predicted) | FAM20B |
| TargetScan Human | Moderate (predicted) | FAM221B |
| TargetScan Human | Moderate (predicted) | FBXL20 |
| TargetScan Human | Moderate (predicted) | FBXO48 |
| TargetScan Human | Moderate (predicted) | FEM1A |
| TargetScan Human | Moderate (predicted) | FFAR4 |
| TargetScan Human | Moderate (predicted) | FGB |
| TargetScan Human | Moderate (predicted) | FGF5 |
| TargetScan Human | Moderate (predicted) | FHAD1 |
| TargetScan Human | Moderate (predicted) | FIBIN |
| TargetScan Human | Moderate (predicted) | FLG |
| TargetScan Human | Moderate (predicted) | FLJ44635 |
| TargetScan Human | Moderate (predicted) | FMNL3 |
| TargetScan Human | Moderate (predicted) | FMO2 |
| TargetScan Human | Moderate (predicted) | FOXL2NB |
| TargetScan Human | Moderate (predicted) | FOXRED2 |
| TargetScan Human | Moderate (predicted) | FRMPD4 |
| TargetScan Human | Moderate (predicted) | FUT7 |
| TargetScan Human | Moderate (predicted) | GABRP |
| TargetScan Human | Moderate (predicted) | GALNS |
| TargetScan Human | Moderate (predicted) | GINS3 |
| TargetScan Human | Moderate (predicted) | GJB7 |
| TargetScan Human | Moderate (predicted) | GJC2 |
| TargetScan Human | Moderate (predicted) | GJC3 |
| TargetScan Human | Moderate (predicted) | GLYCTK |
| TargetScan Human | Moderate (predicted) | GOLGA5 |
| TargetScan Human | Moderate (predicted) | GOLGB1 |
| TargetScan Human | Moderate (predicted) | GORASP1 |
| TargetScan Human | Moderate (predicted) | GP2 |
| TargetScan Human | Moderate (predicted) | GPATCH3 |
| TargetScan Human | Moderate (predicted) | GPC1 |
| TargetScan Human | Moderate (predicted) | GPKOW |
| TargetScan Human | Moderate (predicted) | GPR65 |
| TargetScan Human | Moderate (predicted) | GPR78 |
| TargetScan Human | Moderate (predicted) | GREB1 |
| TargetScan Human | Moderate (predicted) | GRID1 |
| TargetScan Human | Moderate (predicted) | GTF3C3 |
| TargetScan Human | Moderate (predicted) | GUCD1 |
| TargetScan Human | Moderate (predicted) | H1FX |
| TargetScan Human | Moderate (predicted) | H6PD |
| TargetScan Human | Moderate (predicted) | HCAR2 |
| TargetScan Human | Moderate (predicted) | HCAR3 |
| TargetScan Human | Moderate (predicted) | HDAC5 |
| TargetScan Human | Moderate (predicted) | HERC6 |
| TargetScan Human | Moderate (predicted) | HES2 |
| TargetScan Human | Moderate (predicted) | HHAT |
| TargetScan Human | Moderate (predicted) | HHIPL1 |
| TargetScan Human | Moderate (predicted) | HIP1R |
| TargetScan Human | Moderate (predicted) | HIST1H4L |
| TargetScan Human | Moderate (predicted) | HIST2H2BF |
| TargetScan Human | Moderate (predicted) | HIVEP2 |
| TargetScan Human | Moderate (predicted) | HMGCR |
| TargetScan Human | Moderate (predicted) | HNRNPA2B1 |
| TargetScan Human | Moderate (predicted) | HPS6 |
| TargetScan Human | Moderate (predicted) | HSH2D |
| TargetScan Human | Moderate (predicted) | HSPB2 |
| TargetScan Human | Moderate (predicted) | IFNG |
| TargetScan Human | Moderate (predicted) | IGHMBP2 |
| TargetScan Human | Moderate (predicted) | IKBKG |
| TargetScan Human | Moderate (predicted) | IL10RA |
| TargetScan Human | Moderate (predicted) | IL1F10 |
| TargetScan Human | Moderate (predicted) | IL22RA1 |
| TargetScan Human | Moderate (predicted) | IL2RB |
| TargetScan Human | Moderate (predicted) | IQCJ |
| TargetScan Human | Moderate (predicted) | IQSEC3 |
| TargetScan Human | Moderate (predicted) | IRF1 |
| TargetScan Human | Moderate (predicted) | ITGA7 |
| TargetScan Human | Moderate (predicted) | ITIH6 |
| TargetScan Human | Moderate (predicted) | ITPKB |
| TargetScan Human | Moderate (predicted) | ITSN2 |
| TargetScan Human | Moderate (predicted) | IYD |
| TargetScan Human | Moderate (predicted) | KANSL2 |
| TargetScan Human | Moderate (predicted) | KBTBD11 |
| TargetScan Human | Moderate (predicted) | KBTBD13 |
| TargetScan Human | Moderate (predicted) | KCNA7 |
| TargetScan Human | Moderate (predicted) | KCNH8 |
| TargetScan Human | Moderate (predicted) | KCNJ13 |
| TargetScan Human | Moderate (predicted) | KCNJ9 |
| TargetScan Human | Moderate (predicted) | KCTD7 |
| TargetScan Human | Moderate (predicted) | KIAA0141 |
| TargetScan Human | Moderate (predicted) | KIAA0753 |
| TargetScan Human | Moderate (predicted) | KIAA1257 |
| TargetScan Human | Moderate (predicted) | KIF24 |
| TargetScan Human | Moderate (predicted) | KLHDC7B |
| TargetScan Human | Moderate (predicted) | KLHL40 |
| TargetScan Human | Moderate (predicted) | KLK5 |
| TargetScan Human | Moderate (predicted) | KMO |
| TargetScan Human | Moderate (predicted) | KNCN |
| TargetScan Human | Moderate (predicted) | KRT74 |
| TargetScan Human | Moderate (predicted) | KRTAP5-4 |
| TargetScan Human | Moderate (predicted) | KRTAP5-7 |
| TargetScan Human | Moderate (predicted) | KRTAP5-8 |
| TargetScan Human | Moderate (predicted) | LACC1 |
| TargetScan Human | Moderate (predicted) | LAMC2 |
| TargetScan Human | Moderate (predicted) | LAMTOR3 |
| TargetScan Human | Moderate (predicted) | LEPROT |
| TargetScan Human | Moderate (predicted) | LETM1 |
| TargetScan Human | Moderate (predicted) | LHX6 |
| TargetScan Human | Moderate (predicted) | LIG4 |
| TargetScan Human | Moderate (predicted) | LILRA2 |
| TargetScan Human | Moderate (predicted) | LILRA6 |
| TargetScan Human | Moderate (predicted) | LIMA1 |
| TargetScan Human | Moderate (predicted) | LIMCH1 |
| TargetScan Human | Moderate (predicted) | LIPH |
| TargetScan Human | Moderate (predicted) | LMLN |
| TargetScan Human | Moderate (predicted) | LMOD1 |
| TargetScan Human | Moderate (predicted) | LOC100506422 |
| TargetScan Human | Moderate (predicted) | LPIN2 |
| TargetScan Human | Moderate (predicted) | LRP3 |
| TargetScan Human | Moderate (predicted) | LRPAP1 |
| TargetScan Human | Moderate (predicted) | LRRC15 |
| TargetScan Human | Moderate (predicted) | LRRC37A3 (includes others) |
| TargetScan Human | Moderate (predicted) | LRRC46 |
| TargetScan Human | Moderate (predicted) | LSM4 |
| TargetScan Human | Moderate (predicted) | LYRM4 |
| TargetScan Human | Moderate (predicted) | LYRM9 |
| TargetScan Human | Moderate (predicted) | LZTS1 |
| TargetScan Human | Moderate (predicted) | LZTS2 |
| TargetScan Human | Moderate (predicted) | MADD |
| TargetScan Human | Moderate (predicted) | MAGOHB |
| TargetScan Human | Moderate (predicted) | MALL |
| TargetScan Human | Moderate (predicted) | MAP3K9 |
| TargetScan Human | Moderate (predicted) | MAP6 |
| TargetScan Human | Moderate (predicted) | MARCH4 |
| TargetScan Human | Moderate (predicted) | MAT1A |
| TargetScan Human | Moderate (predicted) | MBP |
| TargetScan Human | Moderate (predicted) | MCAM |
| TargetScan Human | Moderate (predicted) | MCTP2 |
| TargetScan Human | Moderate (predicted) | MDC1 |
| TargetScan Human | Moderate (predicted) | MED20 |
| TargetScan Human | Moderate (predicted) | METTL21A |
| TargetScan Human | Moderate (predicted) | MLX |
| TargetScan Human | Moderate (predicted) | MLYCD |
| TargetScan Human | Moderate (predicted) | MMP2 |
| TargetScan Human | Moderate (predicted) | MMP25 |
| TargetScan Human | Moderate (predicted) | MMP28 |
| TargetScan Human | Moderate (predicted) | MOB3A |
| TargetScan Human | Moderate (predicted) | MOBP |
| TargetScan Human | Moderate (predicted) | MPZL1 |
| TargetScan Human | Moderate (predicted) | MRPL10 |
| TargetScan Human | Moderate (predicted) | MRPL11 |
| TargetScan Human | Moderate (predicted) | MRPL15 |
| TargetScan Human | Moderate (predicted) | MRPL30 |
| TargetScan Human | Moderate (predicted) | MRPS7 |
| TargetScan Human | Moderate (predicted) | MS4A6A |
| TargetScan Human | Moderate (predicted) | MTMR6 |
| TargetScan Human | Moderate (predicted) | MTSS1L |
| TargetScan Human | Moderate (predicted) | MUC2 |
| TargetScan Human | Moderate (predicted) | MUC5B |
| TargetScan Human | Moderate (predicted) | MVK |
| TargetScan Human | Moderate (predicted) | MYEOV |
| TargetScan Human | Moderate (predicted) | MYL9 |
| TargetScan Human | Moderate (predicted) | MYO15A |
| TargetScan Human | Moderate (predicted) | MYO1D |
| TargetScan Human | Moderate (predicted) | NAA60 |
| TargetScan Human | Moderate (predicted) | NAT9 |
| TargetScan Human | Moderate (predicted) | NBPF3 |
| TargetScan Human | Moderate (predicted) | NCR3LG1 |
| TargetScan Human | Moderate (predicted) | NDUFA2 |
| TargetScan Human | Moderate (predicted) | NFAM1 |
| TargetScan Human | Moderate (predicted) | NFATC1 |
| TargetScan Human | Moderate (predicted) | NFATC4 |
| TargetScan Human | Moderate (predicted) | NICN1 |
| TargetScan Human | Moderate (predicted) | NKX2-3 |
| TargetScan Human | Moderate (predicted) | NLRC5 |
| TargetScan Human | Moderate (predicted) | NLRP2 |
| TargetScan Human | Moderate (predicted) | NME9 |
| TargetScan Human | Moderate (predicted) | NMRK2 |
| TargetScan Human | Moderate (predicted) | NOD1 |
| TargetScan Human | Moderate (predicted) | NOL12 |
| TargetScan Human | Moderate (predicted) | NOTCH2 |
| TargetScan Human | Moderate (predicted) | NR2C1 |
| TargetScan Human | Moderate (predicted) | NRSN2 |
| TargetScan Human | Moderate (predicted) | NSL1 |
| TargetScan Human | Moderate (predicted) | NT5E |
| TargetScan Human | Moderate (predicted) | NT5M |
| TargetScan Human | Moderate (predicted) | NTF4 |
| TargetScan Human | Moderate (predicted) | NUDT16 |
| TargetScan Human | Moderate (predicted) | OPHN1 |
| TargetScan Human | Moderate (predicted) | OR13A1 |
| TargetScan Human | Moderate (predicted) | ORC4 |
| TargetScan Human | Moderate (predicted) | OTUB1 |
| TargetScan Human | Moderate (predicted) | P2RX4 |
| TargetScan Human | Moderate (predicted) | P2RX6 |
| TargetScan Human | Moderate (predicted) | P2RX7 |
| TargetScan Human | Moderate (predicted) | PACSIN1 |
| TargetScan Human | Moderate (predicted) | PADI2 |
| TargetScan Human | Moderate (predicted) | PAGR1 |
| TargetScan Human | Moderate (predicted) | PAN2 |
| TargetScan Human | Moderate (predicted) | PANK1 |
| TargetScan Human | Moderate (predicted) | PAQR4 |
| TargetScan Human | Moderate (predicted) | PARP11 |
| TargetScan Human | Moderate (predicted) | PCDH15 |
| TargetScan Human | Moderate (predicted) | PDIA3 |
| TargetScan Human | Moderate (predicted) | PDK2 |
| TargetScan Human | Moderate (predicted) | PES1 |
| TargetScan Human | Moderate (predicted) | PFAS |
| TargetScan Human | Moderate (predicted) | PGC |
| TargetScan Human | Moderate (predicted) | PHKA1 |
| TargetScan Human | Moderate (predicted) | PKHD1 |
| TargetScan Human | Moderate (predicted) | PLA2G16 |
| TargetScan Human | Moderate (predicted) | PLA2G2D |
| TargetScan Human | Moderate (predicted) | PLA2G5 |
| TargetScan Human | Moderate (predicted) | PLCB3 |
| TargetScan Human | Moderate (predicted) | PLEKHG2 |
| TargetScan Human | Moderate (predicted) | PLIN3 |
| TargetScan Human | Moderate (predicted) | PLIN4 |
| TargetScan Human | Moderate (predicted) | PNPLA1 |
| TargetScan Human | Moderate (predicted) | PNPT1 |
| TargetScan Human | Moderate (predicted) | POLR1A |
| TargetScan Human | Moderate (predicted) | POM121/POM121C |
| TargetScan Human | Moderate (predicted) | PORCN |
| TargetScan Human | Moderate (predicted) | PPCDC |
| TargetScan Human | Moderate (predicted) | PPIF |
| TargetScan Human | Moderate (predicted) | PPIL2 |
| TargetScan Human | Moderate (predicted) | PPP1R12B |
| TargetScan Human | Moderate (predicted) | PPP2R1B |
| TargetScan Human | Moderate (predicted) | PPP3R2 |
| TargetScan Human | Moderate (predicted) | PPP6R3 |
| TargetScan Human | Moderate (predicted) | PRAMEF18/PRAMEF19 |
| TargetScan Human | Moderate (predicted) | PRCD |
| TargetScan Human | Moderate (predicted) | PRF1 |
| TargetScan Human | Moderate (predicted) | PROS1 |
| TargetScan Human | Moderate (predicted) | PROSER2 |
| TargetScan Human | Moderate (predicted) | PRR3 |
| TargetScan Human | Moderate (predicted) | PSAPL1 |
| TargetScan Human | Moderate (predicted) | PSG1 |
| TargetScan Human | Moderate (predicted) | PSG4 |
| TargetScan Human | Moderate (predicted) | PSG8 |
| TargetScan Human | Moderate (predicted) | PSMB9 |
| TargetScan Human | Moderate (predicted) | PSMD13 |
| TargetScan Human | Moderate (predicted) | PSMG3 |
| TargetScan Human | Moderate (predicted) | PTGDR2 |
| TargetScan Human | Moderate (predicted) | PTGES2 |
| TargetScan Human | Moderate (predicted) | PTPRB |
| TargetScan Human | Moderate (predicted) | PTPRO |
| TargetScan Human | Moderate (predicted) | PXMP4 |
| TargetScan Human | Moderate (predicted) | PYROXD1 |
| TargetScan Human | Moderate (predicted) | QRSL1 |
| TargetScan Human | Moderate (predicted) | RAB37 |
| TargetScan Human | Moderate (predicted) | RAB43 |
| TargetScan Human | Moderate (predicted) | RASA3 |
| TargetScan Human | Moderate (predicted) | RASAL1 |
| TargetScan Human | Moderate (predicted) | RAX |
| TargetScan Human | Moderate (predicted) | RBM14 |
| TargetScan Human | Moderate (predicted) | RBM4 |
| TargetScan Human | Moderate (predicted) | RCN3 |
| TargetScan Human | Moderate (predicted) | REL |
| TargetScan Human | Moderate (predicted) | RFC5 |
| TargetScan Human | Moderate (predicted) | RNF26 |
| TargetScan Human | Moderate (predicted) | ROBO4 |
| TargetScan Human | Moderate (predicted) | RP1 |
| TargetScan Human | Moderate (predicted) | RPL28 |
| TargetScan Human | Moderate (predicted) | RPL36A |
| TargetScan Human | Moderate (predicted) | RRM2 |
| TargetScan Human | Moderate (predicted) | RTCA |
| TargetScan Human | Moderate (predicted) | RTN4RL1 |
| TargetScan Human | Moderate (predicted) | RUFY4 |
| TargetScan Human | Moderate (predicted) | SAP30L |
| TargetScan Human | Moderate (predicted) | SARDH |
| TargetScan Human | Moderate (predicted) | SCLY |
| TargetScan Human | Moderate (predicted) | SEC14L1 |
| TargetScan Human | Moderate (predicted) | SEC14L2 |
| TargetScan Human | Moderate (predicted) | SEC23IP |
| TargetScan Human | Moderate (predicted) | SEC31B |
| TargetScan Human | Moderate (predicted) | SECISBP2 |
| TargetScan Human | Moderate (predicted) | SELV |
| TargetScan Human | Moderate (predicted) | SERPINB8 |
| TargetScan Human | Moderate (predicted) | SERTM1 |
| TargetScan Human | Moderate (predicted) | SETD6 |
| TargetScan Human | Moderate (predicted) | SFRP5 |
| TargetScan Human | Moderate (predicted) | SGOL1 |
| TargetScan Human | Moderate (predicted) | SIRT2 |
| TargetScan Human | Moderate (predicted) | SIRT5 |
| TargetScan Human | Moderate (predicted) | SIT1 |
| TargetScan Human | Moderate (predicted) | SIX5 |
| TargetScan Human | Moderate (predicted) | SLC12A3 |
| TargetScan Human | Moderate (predicted) | SLC1A5 |
| TargetScan Human | Moderate (predicted) | SLC22A13 |
| TargetScan Human | Moderate (predicted) | SLC25A10 |
| TargetScan Human | Moderate (predicted) | SLC26A9 |
| TargetScan Human | Moderate (predicted) | SLC28A1 |
| TargetScan Human | Moderate (predicted) | SLC28A2 |
| TargetScan Human | Moderate (predicted) | SLC35A5 |
| TargetScan Human | Moderate (predicted) | SLC39A11 |
| TargetScan Human | Moderate (predicted) | SLC39A8 |
| TargetScan Human | Moderate (predicted) | SLC44A2 |
| TargetScan Human | Moderate (predicted) | SLC45A3 |
| TargetScan Human | Moderate (predicted) | SLC46A1 |
| TargetScan Human | Moderate (predicted) | SLC46A2 |
| TargetScan Human | Moderate (predicted) | SMC2 |
| TargetScan Human | Moderate (predicted) | SNAI3 |
| TargetScan Human | Moderate (predicted) | SNX22 |
| TargetScan Human | Moderate (predicted) | SNX24 |
| TargetScan Human | Moderate (predicted) | SNX32 |
| TargetScan Human | Moderate (predicted) | SNX33 |
| TargetScan Human | Moderate (predicted) | SP3 |
| TargetScan Human | Moderate (predicted) | SP5 |
| TargetScan Human | Moderate (predicted) | SP6 |
| TargetScan Human | Moderate (predicted) | SPAG8 |
| TargetScan Human | Moderate (predicted) | SPDEF |
| TargetScan Human | Moderate (predicted) | SPRN |
| TargetScan Human | Moderate (predicted) | SPRR3 |
| TargetScan Human | Moderate (predicted) | SPRTN |
| TargetScan Human | Moderate (predicted) | SS18 |
| TargetScan Human | Moderate (predicted) | SSC4D |
| TargetScan Human | Moderate (predicted) | SSR2 |
| TargetScan Human | Moderate (predicted) | SSTR5 |
| TargetScan Human | Moderate (predicted) | SSX2/SSX2B |
| TargetScan Human | Moderate (predicted) | ST7L |
| TargetScan Human | Moderate (predicted) | ST8SIA3 |
| TargetScan Human | Moderate (predicted) | STC2 |
| TargetScan Human | Moderate (predicted) | STRA6 |
| TargetScan Human | Moderate (predicted) | SYNDIG1L |
| TargetScan Human | Moderate (predicted) | SYNPO |
| TargetScan Human | Moderate (predicted) | TACR1 |
| TargetScan Human | Moderate (predicted) | TAF8 |
| TargetScan Human | Moderate (predicted) | TAPBP |
| TargetScan Human | Moderate (predicted) | TCEB2 |
| TargetScan Human | Moderate (predicted) | TCF7 |
| TargetScan Human | Moderate (predicted) | TEX19 |
| TargetScan Human | Moderate (predicted) | THAP3 |
| TargetScan Human | Moderate (predicted) | THOC6 |
| TargetScan Human | Moderate (predicted) | THTPA |
| TargetScan Human | Moderate (predicted) | TIGD6 |
| TargetScan Human | Moderate (predicted) | TJAP1 |
| TargetScan Human | Moderate (predicted) | TK2 |
| TargetScan Human | Moderate (predicted) | TMEM151A |
| TargetScan Human | Moderate (predicted) | TMEM231 |
| TargetScan Human | Moderate (predicted) | TMEM266 |
| TargetScan Human | Moderate (predicted) | TMEM37 |
| TargetScan Human | Moderate (predicted) | TMEM50A |
| TargetScan Human | Moderate (predicted) | TMEM79 |
| TargetScan Human | Moderate (predicted) | TMEM8B |
| TargetScan Human | Moderate (predicted) | TMPRSS11F |
| TargetScan Human | Moderate (predicted) | TMPRSS4 |
| TargetScan Human | Moderate (predicted) | TNFAIP2 |
| TargetScan Human | Moderate (predicted) | TNFAIP8L3 |
| TargetScan Human | Moderate (predicted) | TNIP2 |
| TargetScan Human | Moderate (predicted) | TOR4A |
| TargetScan Human | Moderate (predicted) | TP73 |
| TargetScan Human | Moderate (predicted) | TREML2 |
| TargetScan Human | Moderate (predicted) | TRIM62 |
| TargetScan Human | Moderate (predicted) | TRIM65 |
| TargetScan Human | Moderate (predicted) | TRIM68 |
| TargetScan Human | Moderate (predicted) | TRIM9 |
| TargetScan Human | Moderate (predicted) | TRIOBP |
| TargetScan Human | Moderate (predicted) | TTC19 |
| TargetScan Human | Moderate (predicted) | TTC30B |
| TargetScan Human | Moderate (predicted) | TTLL10 |
| TargetScan Human | Moderate (predicted) | TUFT1 |
| TargetScan Human | Moderate (predicted) | UBA52 |
| TargetScan Human | Moderate (predicted) | UBAC2 |
| TargetScan Human | Moderate (predicted) | UBOX5 |
| TargetScan Human | Moderate (predicted) | UCK1 |
| TargetScan Human | Moderate (predicted) | ULBP1 |
| TargetScan Human | Moderate (predicted) | UNK |
| TargetScan Human | Moderate (predicted) | UNKL |
| TargetScan Human | Moderate (predicted) | VPS33A |
| TargetScan Human | Moderate (predicted) | VSTM4 |
| TargetScan Human | Moderate (predicted) | WDR5 |
| TargetScan Human | Moderate (predicted) | WDR7 |
| TargetScan Human | Moderate (predicted) | WSCD1 |
| TargetScan Human | Moderate (predicted) | WSCD2 |
| TargetScan Human | Moderate (predicted) | YTHDC1 |
| TargetScan Human | Moderate (predicted) | ZBED1 |
| TargetScan Human | Moderate (predicted) | ZC3H12D |
| TargetScan Human | Moderate (predicted) | ZC3H13 |
| TargetScan Human | Moderate (predicted) | ZDHHC18 |
| TargetScan Human | Moderate (predicted) | ZDHHC8 |
| TargetScan Human | Moderate (predicted) | ZMYM3 |
| TargetScan Human | Moderate (predicted) | ZNF254 |
| TargetScan Human | Moderate (predicted) | ZNF396 |
| TargetScan Human | Moderate (predicted) | ZNF473 |
| TargetScan Human | Moderate (predicted) | ZNF530 |
| TargetScan Human | Moderate (predicted) | ZNF559-ZNF177 |
| TargetScan Human | Moderate (predicted) | ZNF578 |
| TargetScan Human | Moderate (predicted) | ZNF592 |
| TargetScan Human | Moderate (predicted) | ZNF70 |
| TargetScan Human | Moderate (predicted) | ZNF737 |
| TargetScan Human | Moderate (predicted) | ZNF75D |
| TargetScan Human | Moderate (predicted) | ZNF780B |
| TargetScan Human | Moderate (predicted) | ZNF80 |
| TargetScan Human | Moderate (predicted) | ZNF831 |

S2C Table: Verified/predicted target genes of miR-490*

| Source | Status | Gene Abbv |
| --- | --- | --- |
| TargetScan Human | High (predicted) | ARHGAP26 |
| TargetScan Human | High (predicted) | C8orf37 |
| TargetScan Human | High (predicted) | CXorf56 |
| TargetScan Human | High (predicted) | DCAF8L1 |
| TargetScan Human | High (predicted) | DNAJB14 |
| TargetScan Human | High (predicted) | F8A1 (includes others) |
| TargetScan Human | High (predicted) | FOS |
| TargetScan Human | High (predicted) | GCH1 |
| TargetScan Human | High (predicted) | HYPM |
| TargetScan Human | High (predicted) | LILRA3 |
| TargetScan Human | High (predicted) | LMOD1 |
| TargetScan Human | High (predicted) | NBR1 |
| TargetScan Human | High (predicted) | PDE8B |
| TargetScan Human | High (predicted) | PLCXD3 |
| TargetScan Human | High (predicted) | RPS6KA3 |
| TargetScan Human | High (predicted) | SEL1L3 |
| TargetScan Human | High (predicted) | SLC16A7 |
| TargetScan Human | High (predicted) | TAOK1 |
| TargetScan Human | High (predicted) | TMEM123 |
| TargetScan Human | High (predicted) | ZNF562 |
| TargetScan Human | High (predicted) | ZNF770 |
| TargetScan Human | High (predicted) | ZXDB |
| TargetScan Human | High (predicted),Moderate (predicted) | ESR2 |
| TargetScan Human | Moderate (predicted) | ADCYAP1 |
| TargetScan Human | Moderate (predicted) | ADGRE3 |
| TargetScan Human | Moderate (predicted) | ADGRF3 |
| TargetScan Human | Moderate (predicted) | ADSS |
| TargetScan Human | Moderate (predicted) | AFF3 |
| TargetScan Human | Moderate (predicted) | AIMP1 |
| TargetScan Human | Moderate (predicted) | AKR1C4 |
| TargetScan Human | Moderate (predicted) | ALDH1A2 |
| TargetScan Human | Moderate (predicted) | ALDH1B1 |
| TargetScan Human | Moderate (predicted) | ALX4 |
| TargetScan Human | Moderate (predicted) | AMMECR1L |
| TargetScan Human | Moderate (predicted) | ANK2 |
| TargetScan Human | Moderate (predicted) | ANKLE2 |
| TargetScan Human | Moderate (predicted) | ANKRD33B |
| TargetScan Human | Moderate (predicted) | ANKRD34C |
| TargetScan Human | Moderate (predicted) | AP1S3 |
| TargetScan Human | Moderate (predicted) | APPL1 |
| TargetScan Human | Moderate (predicted) | ARHGAP42 |
| TargetScan Human | Moderate (predicted) | ARL4A |
| TargetScan Human | Moderate (predicted) | ARL4D |
| TargetScan Human | Moderate (predicted) | ATP1B1 |
| TargetScan Human | Moderate (predicted) | B4GALNT3 |
| TargetScan Human | Moderate (predicted) | BACH1 |
| TargetScan Human | Moderate (predicted) | BAHD1 |
| TargetScan Human | Moderate (predicted) | BEND7 |
| TargetScan Human | Moderate (predicted) | BMP2 |
| TargetScan Human | Moderate (predicted) | BORA |
| TargetScan Human | Moderate (predicted) | BRINP2 |
| TargetScan Human | Moderate (predicted) | BTAF1 |
| TargetScan Human | Moderate (predicted) | BTC |
| TargetScan Human | Moderate (predicted) | BTNL9 |
| TargetScan Human | Moderate (predicted) | C11orf58 |
| TargetScan Human | Moderate (predicted) | C12orf74 |
| TargetScan Human | Moderate (predicted) | C14orf79 |
| TargetScan Human | Moderate (predicted) | C16orf45 |
| TargetScan Human | Moderate (predicted) | C17orf80 |
| TargetScan Human | Moderate (predicted) | C4orf3 |
| TargetScan Human | Moderate (predicted) | C5orf64 |
| TargetScan Human | Moderate (predicted) | C8A |
| TargetScan Human | Moderate (predicted) | C9 |
| TargetScan Human | Moderate (predicted) | CA7 |
| TargetScan Human | Moderate (predicted) | CAGE1 |
| TargetScan Human | Moderate (predicted) | CAMTA1 |
| TargetScan Human | Moderate (predicted) | CBX5 |
| TargetScan Human | Moderate (predicted) | CCDC6 |
| TargetScan Human | Moderate (predicted) | CCDC88A |
| TargetScan Human | Moderate (predicted) | CCNG1 |
| TargetScan Human | Moderate (predicted) | CDC42EP3 |
| TargetScan Human | Moderate (predicted) | CDH19 |
| TargetScan Human | Moderate (predicted) | CDS2 |
| TargetScan Human | Moderate (predicted) | CEP162 |
| TargetScan Human | Moderate (predicted) | CEP19 |
| TargetScan Human | Moderate (predicted) | CHRDL1 |
| TargetScan Human | Moderate (predicted) | CHRNA2 |
| TargetScan Human | Moderate (predicted) | CLIP4 |
| TargetScan Human | Moderate (predicted) | CLN3 |
| TargetScan Human | Moderate (predicted) | CNDP2 |
| TargetScan Human | Moderate (predicted) | COA5 |
| TargetScan Human | Moderate (predicted) | COL4A3 |
| TargetScan Human | Moderate (predicted) | COL6A1 |
| TargetScan Human | Moderate (predicted) | CST8 |
| TargetScan Human | Moderate (predicted) | CXADR |
| TargetScan Human | Moderate (predicted) | CXorf40A/CXorf40B |
| TargetScan Human | Moderate (predicted) | DAAM1 |
| TargetScan Human | Moderate (predicted) | DACH1 |
| TargetScan Human | Moderate (predicted) | DDI1 |
| TargetScan Human | Moderate (predicted) | DHX37 |
| TargetScan Human | Moderate (predicted) | DIAPH3 |
| TargetScan Human | Moderate (predicted) | DOPEY2 |
| TargetScan Human | Moderate (predicted) | DSCC1 |
| TargetScan Human | Moderate (predicted) | DTL |
| TargetScan Human | Moderate (predicted) | DYNC1I2 |
| TargetScan Human | Moderate (predicted) | E2F6 |
| TargetScan Human | Moderate (predicted) | ECT2 |
| TargetScan Human | Moderate (predicted) | EFCAB1 |
| TargetScan Human | Moderate (predicted) | EFCAB13 |
| TargetScan Human | Moderate (predicted) | EFCAB14 |
| TargetScan Human | Moderate (predicted) | EFCAB2 |
| TargetScan Human | Moderate (predicted) | EIF4EBP2 |
| TargetScan Human | Moderate (predicted) | ELK4 |
| TargetScan Human | Moderate (predicted) | ELMO1 |
| TargetScan Human | Moderate (predicted) | EMC1 |
| TargetScan Human | Moderate (predicted) | EMCN |
| TargetScan Human | Moderate (predicted) | ENKUR |
| TargetScan Human | Moderate (predicted) | EPHA3 |
| TargetScan Human | Moderate (predicted) | EPHA4 |
| TargetScan Human | Moderate (predicted) | EPM2A |
| TargetScan Human | Moderate (predicted) | EPPIN-WFDC6 |
| TargetScan Human | Moderate (predicted) | ETF1 |
| TargetScan Human | Moderate (predicted) | ETS1 |
| TargetScan Human | Moderate (predicted) | EXOC6 |
| TargetScan Human | Moderate (predicted) | EXTL2 |
| TargetScan Human | Moderate (predicted) | EYA3 |
| TargetScan Human | Moderate (predicted) | FAM102A |
| TargetScan Human | Moderate (predicted) | FAM105A |
| TargetScan Human | Moderate (predicted) | FAM19A5 |
| TargetScan Human | Moderate (predicted) | FAM227B |
| TargetScan Human | Moderate (predicted) | FAM65B |
| TargetScan Human | Moderate (predicted) | FAM76B |
| TargetScan Human | Moderate (predicted) | FANCF |
| TargetScan Human | Moderate (predicted) | FBXL3 |
| TargetScan Human | Moderate (predicted) | FCRL1 |
| TargetScan Human | Moderate (predicted) | FECH |
| TargetScan Human | Moderate (predicted) | FERMT1 |
| TargetScan Human | Moderate (predicted) | FERMT2 |
| TargetScan Human | Moderate (predicted) | FGF14 |
| TargetScan Human | Moderate (predicted) | FGF5 |
| TargetScan Human | Moderate (predicted) | FGL1 |
| TargetScan Human | Moderate (predicted) | FNDC3A |
| TargetScan Human | Moderate (predicted) | FOXP2 |
| TargetScan Human | Moderate (predicted) | FOXRED2 |
| TargetScan Human | Moderate (predicted) | FRMD6 |
| TargetScan Human | Moderate (predicted) | G3BP2 |
| TargetScan Human | Moderate (predicted) | GANC |
| TargetScan Human | Moderate (predicted) | GCSH |
| TargetScan Human | Moderate (predicted) | GINS3 |
| TargetScan Human | Moderate (predicted) | GLTSCR1L |
| TargetScan Human | Moderate (predicted) | GOLT1B |
| TargetScan Human | Moderate (predicted) | GPX5 |
| TargetScan Human | Moderate (predicted) | GSTM3 |
| TargetScan Human | Moderate (predicted) | GTF2H5 |
| TargetScan Human | Moderate (predicted) | GUCY1A3 |
| TargetScan Human | Moderate (predicted) | GYS2 |
| TargetScan Human | Moderate (predicted) | HERC3 |
| TargetScan Human | Moderate (predicted) | HMG20A |
| TargetScan Human | Moderate (predicted) | HPS5 |
| TargetScan Human | Moderate (predicted) | HTR5A |
| TargetScan Human | Moderate (predicted) | HTR7 |
| TargetScan Human | Moderate (predicted) | IDS |
| TargetScan Human | Moderate (predicted) | IL20RB |
| TargetScan Human | Moderate (predicted) | IQCA1 |
| TargetScan Human | Moderate (predicted) | IRAK2 |
| TargetScan Human | Moderate (predicted) | KANK2 |
| TargetScan Human | Moderate (predicted) | KCNA1 |
| TargetScan Human | Moderate (predicted) | KCNA5 |
| TargetScan Human | Moderate (predicted) | KCNJ2 |
| TargetScan Human | Moderate (predicted) | KCNS3 |
| TargetScan Human | Moderate (predicted) | KIF11 |
| TargetScan Human | Moderate (predicted) | KIF6 |
| TargetScan Human | Moderate (predicted) | KLHDC7B |
| TargetScan Human | Moderate (predicted) | KLHL40 |
| TargetScan Human | Moderate (predicted) | KMT2A |
| TargetScan Human | Moderate (predicted) | KRT20 |
| TargetScan Human | Moderate (predicted) | KRT222 |
| TargetScan Human | Moderate (predicted) | KRT6A |
| TargetScan Human | Moderate (predicted) | KRTAP1-5 |
| TargetScan Human | Moderate (predicted) | LAMTOR3 |
| TargetScan Human | Moderate (predicted) | LCOR |
| TargetScan Human | Moderate (predicted) | LHFPL3 |
| TargetScan Human | Moderate (predicted) | LHX9 |
| TargetScan Human | Moderate (predicted) | LILRA1 |
| TargetScan Human | Moderate (predicted) | LIN54 |
| TargetScan Human | Moderate (predicted) | LMO4 |
| TargetScan Human | Moderate (predicted) | LONP2 |
| TargetScan Human | Moderate (predicted) | LRFN5 |
| TargetScan Human | Moderate (predicted) | LRIT2 |
| TargetScan Human | Moderate (predicted) | LRSAM1 |
| TargetScan Human | Moderate (predicted) | MAGOHB |
| TargetScan Human | Moderate (predicted) | MALT1 |
| TargetScan Human | Moderate (predicted) | MAP10 |
| TargetScan Human | Moderate (predicted) | MARCH8 |
| TargetScan Human | Moderate (predicted) | MARS2 |
| TargetScan Human | Moderate (predicted) | MAT2A |
| TargetScan Human | Moderate (predicted) | MBNL3 |
| TargetScan Human | Moderate (predicted) | MCHR2 |
| TargetScan Human | Moderate (predicted) | METAP2 |
| TargetScan Human | Moderate (predicted) | MGAT4A |
| TargetScan Human | Moderate (predicted) | MNT |
| TargetScan Human | Moderate (predicted) | MOB3A |
| TargetScan Human | Moderate (predicted) | MOBP |
| TargetScan Human | Moderate (predicted) | MRI1 |
| TargetScan Human | Moderate (predicted) | MRPL22 |
| TargetScan Human | Moderate (predicted) | MSN |
| TargetScan Human | Moderate (predicted) | MTRF1L |
| TargetScan Human | Moderate (predicted) | MTTP |
| TargetScan Human | Moderate (predicted) | MYCBP2 |
| TargetScan Human | Moderate (predicted) | MYEOV |
| TargetScan Human | Moderate (predicted) | MYLIP |
| TargetScan Human | Moderate (predicted) | MYOCD |
| TargetScan Human | Moderate (predicted) | MYOZ3 |
| TargetScan Human | Moderate (predicted) | NACC2 |
| TargetScan Human | Moderate (predicted) | NAMPT |
| TargetScan Human | Moderate (predicted) | NCAPG2 |
| TargetScan Human | Moderate (predicted) | NCAPH |
| TargetScan Human | Moderate (predicted) | NDUFAF6 |
| TargetScan Human | Moderate (predicted) | NFRKB |
| TargetScan Human | Moderate (predicted) | NIPAL3 |
| TargetScan Human | Moderate (predicted) | NLGN4Y |
| TargetScan Human | Moderate (predicted) | NLRP3 |
| TargetScan Human | Moderate (predicted) | NOTCH2 |
| TargetScan Human | Moderate (predicted) | NPHP3 |
| TargetScan Human | Moderate (predicted) | NPHS1 |
| TargetScan Human | Moderate (predicted) | NPHS2 |
| TargetScan Human | Moderate (predicted) | NR2F2 |
| TargetScan Human | Moderate (predicted) | NREP |
| TargetScan Human | Moderate (predicted) | NRXN1 |
| TargetScan Human | Moderate (predicted) | NT5C2 |
| TargetScan Human | Moderate (predicted) | NUP50 |
| TargetScan Human | Moderate (predicted) | OCLN |
| TargetScan Human | Moderate (predicted) | ONECUT1 |
| TargetScan Human | Moderate (predicted) | OPRM1 |
| TargetScan Human | Moderate (predicted) | PAICS |
| TargetScan Human | Moderate (predicted) | PARK2 |
| TargetScan Human | Moderate (predicted) | PARP8 |
| TargetScan Human | Moderate (predicted) | PARVA |
| TargetScan Human | Moderate (predicted) | PAX8 |
| TargetScan Human | Moderate (predicted) | PAXBP1 |
| TargetScan Human | Moderate (predicted) | PBX3 |
| TargetScan Human | Moderate (predicted) | PCDHA1 |
| TargetScan Human | Moderate (predicted) | PCDHA10 |
| TargetScan Human | Moderate (predicted) | PCDHA11 |
| TargetScan Human | Moderate (predicted) | PCDHA12 |
| TargetScan Human | Moderate (predicted) | PCDHA13 |
| TargetScan Human | Moderate (predicted) | PCDHA2 |
| TargetScan Human | Moderate (predicted) | PCDHA3 |
| TargetScan Human | Moderate (predicted) | PCDHA4 |
| TargetScan Human | Moderate (predicted) | PCDHA5 |
| TargetScan Human | Moderate (predicted) | PCDHA6 |
| TargetScan Human | Moderate (predicted) | PCDHA7 |
| TargetScan Human | Moderate (predicted) | PCDHA8 |
| TargetScan Human | Moderate (predicted) | PCDHA9 |
| TargetScan Human | Moderate (predicted) | PCDHAC1 |
| TargetScan Human | Moderate (predicted) | PCDHAC2 |
| TargetScan Human | Moderate (predicted) | PCDHB5 |
| TargetScan Human | Moderate (predicted) | PCNXL2 |
| TargetScan Human | Moderate (predicted) | PCTP |
| TargetScan Human | Moderate (predicted) | PDE3B |
| TargetScan Human | Moderate (predicted) | PEG3 |
| TargetScan Human | Moderate (predicted) | PER3 |
| TargetScan Human | Moderate (predicted) | PGAP1 |
| TargetScan Human | Moderate (predicted) | PGPEP1 |
| TargetScan Human | Moderate (predicted) | PHF19 |
| TargetScan Human | Moderate (predicted) | PIK3CA |
| TargetScan Human | Moderate (predicted) | PIKFYVE |
| TargetScan Human | Moderate (predicted) | PITPNM1 |
| TargetScan Human | Moderate (predicted) | PLCB1 |
| TargetScan Human | Moderate (predicted) | PLEK |
| TargetScan Human | Moderate (predicted) | PLG |
| TargetScan Human | Moderate (predicted) | POLD4 |
| TargetScan Human | Moderate (predicted) | POU2AF1 |
| TargetScan Human | Moderate (predicted) | PPM1E |
| TargetScan Human | Moderate (predicted) | PRDM15 |
| TargetScan Human | Moderate (predicted) | PRELID2 |
| TargetScan Human | Moderate (predicted) | PRELP |
| TargetScan Human | Moderate (predicted) | PRKAG2 |
| TargetScan Human | Moderate (predicted) | PRKAG3 |
| TargetScan Human | Moderate (predicted) | PROSC |
| TargetScan Human | Moderate (predicted) | PRR27 |
| TargetScan Human | Moderate (predicted) | PTPRD |
| TargetScan Human | Moderate (predicted) | PTPRK |
| TargetScan Human | Moderate (predicted) | QRSL1 |
| TargetScan Human | Moderate (predicted) | QTRTD1 |
| TargetScan Human | Moderate (predicted) | RAB30 |
| TargetScan Human | Moderate (predicted) | RAB5B |
| TargetScan Human | Moderate (predicted) | RASGEF1A |
| TargetScan Human | Moderate (predicted) | RASGRF2 |
| TargetScan Human | Moderate (predicted) | RILP |
| TargetScan Human | Moderate (predicted) | RIMBP2 |
| TargetScan Human | Moderate (predicted) | RNASE6 |
| TargetScan Human | Moderate (predicted) | RNF170 |
| TargetScan Human | Moderate (predicted) | RNF182 |
| TargetScan Human | Moderate (predicted) | RNF19B |
| TargetScan Human | Moderate (predicted) | RNF4 |
| TargetScan Human | Moderate (predicted) | ROBO1 |
| TargetScan Human | Moderate (predicted) | ROCK1 |
| TargetScan Human | Moderate (predicted) | RRAGD |
| TargetScan Human | Moderate (predicted) | RRN3 |
| TargetScan Human | Moderate (predicted) | RSBN1 |
| TargetScan Human | Moderate (predicted) | RUFY3 |
| TargetScan Human | Moderate (predicted) | RUFY4 |
| TargetScan Human | Moderate (predicted) | S100PBP |
| TargetScan Human | Moderate (predicted) | SAA2 |
| TargetScan Human | Moderate (predicted) | SARNP |
| TargetScan Human | Moderate (predicted) | SBNO1 |
| TargetScan Human | Moderate (predicted) | SCML2 |
| TargetScan Human | Moderate (predicted) | SCML4 |
| TargetScan Human | Moderate (predicted) | SCN1A |
| TargetScan Human | Moderate (predicted) | SEH1L |
| TargetScan Human | Moderate (predicted) | SEMA3A |
| TargetScan Human | Moderate (predicted) | SERPIND1 |
| TargetScan Human | Moderate (predicted) | SETD3 |
| TargetScan Human | Moderate (predicted) | SGPP1 |
| TargetScan Human | Moderate (predicted) | SHISA7 |
| TargetScan Human | Moderate (predicted) | SHISA9 |
| TargetScan Human | Moderate (predicted) | SIK3 |
| TargetScan Human | Moderate (predicted) | SLC25A25 |
| TargetScan Human | Moderate (predicted) | SLC26A4 |
| TargetScan Human | Moderate (predicted) | SLC28A3 |
| TargetScan Human | Moderate (predicted) | SLC31A1 |
| TargetScan Human | Moderate (predicted) | SLC35G1 |
| TargetScan Human | Moderate (predicted) | SLC6A1 |
| TargetScan Human | Moderate (predicted) | SLC9A2 |
| TargetScan Human | Moderate (predicted) | SLF2 |
| TargetScan Human | Moderate (predicted) | SMCO1 |
| TargetScan Human | Moderate (predicted) | SOSTDC1 |
| TargetScan Human | Moderate (predicted) | SOX2 |
| TargetScan Human | Moderate (predicted) | SPCS2 |
| TargetScan Human | Moderate (predicted) | SPOCK2 |
| TargetScan Human | Moderate (predicted) | SSUH2 |
| TargetScan Human | Moderate (predicted) | STC1 |
| TargetScan Human | Moderate (predicted) | SUCLG2 |
| TargetScan Human | Moderate (predicted) | SV2B |
| TargetScan Human | Moderate (predicted) | SYAP1 |
| TargetScan Human | Moderate (predicted) | TANC1 |
| TargetScan Human | Moderate (predicted) | TBCK |
| TargetScan Human | Moderate (predicted) | TBK1 |
| TargetScan Human | Moderate (predicted) | TCAF2 |
| TargetScan Human | Moderate (predicted) | TCAIM |
| TargetScan Human | Moderate (predicted) | TCFL5 |
| TargetScan Human | Moderate (predicted) | TET1 |
| TargetScan Human | Moderate (predicted) | TF |
| TargetScan Human | Moderate (predicted) | TFDP1 |
| TargetScan Human | Moderate (predicted) | TFDP3 |
| TargetScan Human | Moderate (predicted) | TFRC |
| TargetScan Human | Moderate (predicted) | TGIF2LY |
| TargetScan Human | Moderate (predicted) | TLR5 |
| TargetScan Human | Moderate (predicted) | TMEM33 |
| TargetScan Human | Moderate (predicted) | TNFRSF10B |
| TargetScan Human | Moderate (predicted) | TNFSF10 |
| TargetScan Human | Moderate (predicted) | TOX3 |
| TargetScan Human | Moderate (predicted) | TRAK1 |
| TargetScan Human | Moderate (predicted) | TRDMT1 |
| TargetScan Human | Moderate (predicted) | TRIM34 |
| TargetScan Human | Moderate (predicted) | TRIM35 |
| TargetScan Human | Moderate (predicted) | TRIM6-TRIM34 |
| TargetScan Human | Moderate (predicted) | TRIM9 |
| TargetScan Human | Moderate (predicted) | TTC33 |
| TargetScan Human | Moderate (predicted) | TUBB1 |
| TargetScan Human | Moderate (predicted) | TUFT1 |
| TargetScan Human | Moderate (predicted) | TXLNA |
| TargetScan Human | Moderate (predicted) | TXNL4A |
| TargetScan Human | Moderate (predicted) | TXNRD1 |
| TargetScan Human | Moderate (predicted) | UBE4A |
| TargetScan Human | Moderate (predicted) | UBR1 |
| TargetScan Human | Moderate (predicted) | UBTD2 |
| TargetScan Human | Moderate (predicted) | UBXN8 |
| TargetScan Human | Moderate (predicted) | UFL1 |
| TargetScan Human | Moderate (predicted) | UGDH |
| TargetScan Human | Moderate (predicted) | UNC5B |
| TargetScan Human | Moderate (predicted) | USH2A |
| TargetScan Human | Moderate (predicted) | USP13 |
| TargetScan Human | Moderate (predicted) | USP48 |
| TargetScan Human | Moderate (predicted) | WDR44 |
| TargetScan Human | Moderate (predicted) | WLS |
| TargetScan Human | Moderate (predicted) | XKR9 |
| TargetScan Human | Moderate (predicted) | YWHAB |
| TargetScan Human | Moderate (predicted) | ZCCHC5 |
| TargetScan Human | Moderate (predicted) | ZCCHC7 |
| TargetScan Human | Moderate (predicted) | ZDHHC15 |
| TargetScan Human | Moderate (predicted) | ZDHHC2 |
| TargetScan Human | Moderate (predicted) | ZDHHC23 |
| TargetScan Human | Moderate (predicted) | ZEB2 |
| TargetScan Human | Moderate (predicted) | ZFYVE27 |
| TargetScan Human | Moderate (predicted) | ZKSCAN4 |
| TargetScan Human | Moderate (predicted) | ZNF107 |
| TargetScan Human | Moderate (predicted) | ZNF112 |
| TargetScan Human | Moderate (predicted) | ZNF143 |
| TargetScan Human | Moderate (predicted) | ZNF230 |
| TargetScan Human | Moderate (predicted) | ZNF385B |
| TargetScan Human | Moderate (predicted) | ZNF714 |
| TargetScan Human | Moderate (predicted) | ZNF808 |

## S2D Table: KEGG_Pathways-miR34a

| KEGG_PATHWAY | Gene Count | p_value | Genes |
| --- | --- | --- | --- |
| rno05200:Pathways in cancer | 43 | 0.00216 | E2F3, GNAI2, PGF, ADCY5, MITF, PPARG, NFKBIA, KITLG, LPAR2, BDKRB2, KIT, CCNE2, WNT1, RASGRP4, BCL2, TGFA, RARB, AXIN2, PLCB1, MYC, CSF1R, PIK3R2, RET, ROCK1, MAP2K1, CYCS, MET, SMAD4, TP53, FGF23, LEF1, CDK6, HGF, RALGDS, WNT2B, PRKCB, CCND1, HDAC1, PLCG1, VEGFA, WNT9B, PDGFRA, PDGFRB |
| rno04144:Endocytosis | 32 | 0.00539 | CAV3, CHMP3, USP8, CHMP7, VPS37A, CYTH4, CXCR1, VPS37B, EPS15L1, EEA1, SNX4, VPS37D, PIP5K1A, KIT, SRC, VPS4A, NEDD4L, AGAP2, EHD4, CSF1R, PARD6B, IL2RB, RET, RAB4A, MET, PSD3, RAB11FIP4, NEDD4, PDGFRA, GRK6, EPN2, RNF41 |
| rno04151:PI3K-Akt signaling pathway | 32 | 0.04826 | PPP2R3A, PGF, PPP2R5A, ITGA10, KITLG, LPAR2, KIT, CCNE2, ITGB8, BCL2, CREB3L1, MYC, PIK3R2, CSF1R, IL2RB, MAP2K1, CREB1, MET, TP53, FGF23, CREB5, CDK6, HGF, IL6R, IRS1, COL5A2, CCND1, PRLR, VEGFA, PDGFRA, PDGFRB, RELN |
| rno04015:Rap1 signaling pathway | 30 | 0.00025 | GNAI2, RAP1GAP, PGF, DRD2, ADCY5, SIPA1, CTNND1, KITLG, LPAR2, KIT, SRC, RRAS, PLCB1, PIK3R2, CSF1R, PARD6B, GNAO1, MAP2K1, MET, FGF23, HGF, RALGDS, FARP2, PRKCB, PRKD1, PLCG1, MAPK13, VEGFA, PDGFRA, PDGFRB |
| rno05206:MicroRNAs in cancer | 29 | 0.00000 | E2F3, ZEB1, PDCD4, CCNE2, BCL2, BMF, MYC, RECK, ROCK1, MAP2K1, MET, TP53, CDK6, PRKCE, IRS1, SIRT1, CDC25A, PRKCB, NOTCH3, NOTCH2, CCND1, NOTCH1, PLCG1, VEGFA, PDGFRA, PDGFRB, MARCKS, MDM4, STMN1 |
| rno04010:MAPK signaling pathway | 27 | 0.02432 | PPM1A, PPP3R1, CACNB1, CACNB3, RASGRP4, MAPT, RRAS, MYC, MAP2K1, PTPRR, TP53, CACNG4, FGF23, CACNG2, CACNA2D2, PRKCB, DUSP4, MAP4K4, RPS6KA4, MAPK13, PDGFRA, PDGFRB, CACNA1E, STMN1, MAP3K14, DUSP8, PLA2G4D |
| rno05205:Proteoglycans in cancer | 24 | 0.00736 | CAV3, ROCK1, MAP2K1, MET, TP53, HGF, PDCD4, SRC, WNT2B, PRKCB, WNT1, CCND1, PLCG1, ANK2, MAPK13, HPSE, ANK3, VEGFA, GAB1, WNT9B, RRAS, MYC, TWIST2, PIK3R2 |
| rno04014:Ras signaling pathway | 24 | 0.03703 | MAP2K1, PGF, MET, KITLG, FGF23, HGF, KIT, RALGDS, PRKCB, KSR2, PLCG1, RASGRP4, VEGFA, GAB1, PDGFRA, PLA2G6, RRAS, PDGFRB, KSR1, RASA4, PLA2G4D, PIK3R2, CSF1R, PLA2G2F |
| rno04060:Cytokine-cytokine receptor interaction | 23 | 0.03138 | IL2RB, CCR1, IL21R, MET, KITLG, TNFSF14, CXCR1, EDAR, IL6R, HGF, KIT, CXCL11, CXCL10, ACVR2B, RELT, PRLR, IL10RB, CXCL16, VEGFA, PDGFRA, PDGFRB, THPO, CSF1R |
| rno04921:Oxytocin signaling pathway | 22 | 0.00232 | GNAO1, GNAI2, ROCK1, MAP2K1, ADCY5, PRKAG1, PPP3R1, CACNB1, CACNG4, CACNB3, RCAN1, CACNG2, CACNA2D2, SRC, PRKCB, CCND1, EEF2K, GUCY1A3, NFATC4, PLCB1, PLA2G4D, PIK3R2 |
| rno04022:cGMP-PKG signaling pathway | 21 | 0.00770 | FXYD2, GNAI2, ROCK1, MAP2K1, ADCY5, CREB1, MRVI1, PPP3R1, CREB5, BDKRB2, ATP1A2, PRKCE, IRS1, KCNJ8, PLN, CREB3L1, GUCY1A3, NFATC4, PLCB1, ADRA1D, PIK3R2 |
| rno04919:Thyroid hormone signaling pathway | 20 | 0.00021 | FXYD2, MAP2K1, TP53, RCAN1, ATP1A2, SRC, PRKCB, NOTCH3, NOTCH2, SLC16A2, NCOA1, NOTCH1, CCND1, HDAC1, PLCG1, PLN, MED27, PLCB1, MYC, PIK3R2 |
| rno04071:Sphingolipid signaling pathway | 20 | 0.00057 | PPP2R3A, GNAI2, ROCK1, MAP2K1, SGPP1, PPP2R5A, TP53, CERS6, CERS5, BDKRB2, PRKCE, PRKCB, S1PR2, S1PR3, MAPK13, BCL2, SMPD1, MS4A2, PLCB1, PIK3R2 |
| rno04360:Axon guidance | 20 | 0.00086 | ABLIM1, GNAI2, EFNB3, ROCK1, ABLIM3, EFNB1, MET, NTN4, PPP3R1, FES, EPHB2, EPHA4, SEMA6A, SEMA4F, UNC5A, SEMA3E, SEMA4C, SEMA4B, NFATC4, SRGAP2 |
| rno04261:Adrenergic signaling in cardiomyocytes | 20 | 0.00404 | FXYD2, PPP2R3A, GNAI2, PPP2R5A, ADCY5, CREB1, CACNB1, CACNG4, CACNB3, CREB5, ATP1A2, CACNG2, CACNA2D2, MAPK13, PLN, BCL2, CREB3L1, PLCB1, ADRA1D, PIK3R2 |
| rno05161:Hepatitis B | 18 | 0.01087 | E2F3, MAP2K1, CREB1, CYCS, TP53, SMAD4, NFKBIA, CDK6, CREB5, SRC, PRKCB, CCNE2, IKBKE, BCL2, CREB3L1, NFATC4, MYC, PIK3R2 |
| rno04152:AMPK signaling pathway | 17 | 0.00932 | PPP2R3A, PFKFB3, PPP2R5A, CREB1, PRKAG1, PPARG, ADIPOR2, CREB5, CFTR, PFKM, SIRT1, IRS1, CCND1, HNF4A, EEF2K, CREB3L1, PIK3R2 |
| rno04310:Wnt signaling pathway | 17 | 0.02536 | TBL1XR1, TP53, PPP3R1, SMAD4, LEF1, DAAM1, PRKCB, WNT2B, WNT1, CCND1, DKK1, WNT9B, NFATC4, AXIN2, PLCB1, FOSL1, MYC |
| rno04750:Inflammatory mediator regulation of TRP channels | 16 | 0.00909 | ADCY5, ASIC2, PRKCH, BDKRB2, PRKCE, SRC, PRKCB, PRKCQ, PLCG1, MAPK13, PLA2G6, PLCB1, HTR2C, PIK3R2, HTR2A, PLA2G4D |
| rno04728:Dopaminergic synapse | 16 | 0.02011 | SCN1A, PPP2R3A, GNAO1, GNAI2, DRD2, PPP2R5A, SLC6A3, ADCY5, MAOA, CREB1, TH, CREB5, PRKCB, MAPK13, PLCB1, CLOCK |
| rno05215:Prostate cancer | 15 | 0.00164 | E2F3, MAP2K1, CREB1, TP53, NFKBIA, LEF1, CREB5, CCNE2, CCND1, BCL2, PDGFRA, CREB3L1, PDGFRB, TGFA, PIK3R2 |
| rno04916:Melanogenesis | 15 | 0.00616 | GNAO1, MAP2K1, GNAI2, CREB1, ADCY5, MITF, LEF1, KITLG, KIT, PRKCB, WNT2B, WNT1, WNT9B, CREB3L1, PLCB1 |
| rno04070:Phosphatidylinositol signaling system | 14 | 0.01162 | SYNJ1, DGKI, PI4KB, PIP5K1A, TMEM55A, PRKCB, CDS2, DGKB, PLCG1, DGKZ, INPP5D, PLCB1, INPP5B, PIK3R2 |
| rno05231:Choline metabolism in cancer | 14 | 0.01732 | SLC44A2, MAP2K1, WASF1, DGKI, PIP5K1A, RALGDS, PRKCB, DGKB, PLCG1, PDGFRA, DGKZ, PDGFRB, PIK3R2, PLA2G4D |
| rno04270:Vascular smooth muscle contraction | 14 | 0.06558 | ROCK1, MAP2K1, ADCY5, MRVI1, PRKCH, PRKCE, PRKCB, PRKCQ, PLA2G6, GUCY1A3, PLCB1, ADRA1D, PLA2G2F, PLA2G4D |
| rno04722:Neurotrophin signaling pathway | 14 | 0.08025 | IRAK2, MAP2K1, NFKBIE, TP53, NFKBIA, IRS1, TP73, IRAK4, PLCG1, MAPK13, BCL2, GAB1, PIK3R2, ARHGDIB |
| rno04110:Cell cycle | 14 | 0.08422 | FZR1, E2F3, E2F5, TP53, SMAD4, CDC23, CDK6, CDC25A, CDKN1C, CCNE2, CCND1, HDAC1, TFDP2, MYC |
| rno04520:Adherens junction | 13 | 0.00221 | PTPRB, PTPRM, WASF1, MET, SMAD4, CTNND1, LEF1, SNAI1, SRC, FARP2, VCL, PVRL1, PVRL2 |
| rno04911:Insulin secretion | 13 | 0.01197 | FXYD2, STX1A, CREB1, ADCY5, CREB5, ATP1A2, PCLO, PRKCB, KCNN3, CREB3L1, VAMP2, PLCB1, GLP1R |
| rno04540:Gap junction | 13 | 0.01424 | MAP2K1, GNAI2, DRD2, ADCY5, SRC, PRKCB, PDGFRA, PDGFRB, GUCY1A3, TUBB1, PLCB1, HTR2C, HTR2A |
| rno05230:Central carbon metabolism in cancer | 12 | 0.00326 | RET, MAP2K1, MET, TP53, PDGFRA, HK1, PDGFRB, SIRT6, PFKM, KIT, MYC, PIK3R2 |
| rno05031:Amphetamine addiction | 12 | 0.00326 | STX1A, CREB1, ADCY5, MAOA, SLC6A3, TH, PPP3R1, CREB3L1, CREB5, FOSB, SIRT1, PRKCB |
| rno05218:Melanoma | 12 | 0.00591 | CCND1, E2F3, MAP2K1, MET, MITF, TP53, PDGFRA, FGF23, PDGFRB, CDK6, HGF, PIK3R2 |
| rno05032:Morphine addiction | 12 | 0.04372 | OPRM1, GABRG3, PDE7B, GNAO1, GNAI2, GABRA3, ADCY5, PDE4B, GRK6, PDE11A, GABBR2, PRKCB |
| rno04915:Estrogen signaling pathway | 12 | 0.05656 | OPRM1, GNAO1, GNAI2, MAP2K1, CREB1, ADCY5, CREB3L1, CREB5, GABBR2, PLCB1, SRC, PIK3R2 |
| rno05214:Glioma | 11 | 0.00876 | CCND1, E2F3, PLCG1, MAP2K1, TP53, PDGFRA, TGFA, PDGFRB, CDK6, PRKCB, PIK3R2 |
| rno05210:Colorectal cancer | 11 | 0.00977 | CCND1, MAP2K1, BCL2, CYCS, TP53, SMAD4, LEF1, AXIN2, MYC, RALGDS, PIK3R2 |
| rno04970:Salivary secretion | 11 | 0.02552 | FXYD2, LPO, NOS1, SLC12A2, ADCY5, GUCY1A3, VAMP2, ATP1A2, PLCB1, ADRA1D, PRKCB |
| rno04917:Prolactin signaling pathway | 11 | 0.02774 | CCND1, PRLR, MAP2K1, MAPK13, LHCGR, TH, IRF1, SOCS7, SOCS4, SRC, PIK3R2 |
| rno05410:Hypertrophic cardiomyopathy (HCM) | 11 | 0.04098 | ACE, ITGB8, PRKAG1, DAG1, LMNA, CACNB1, ITGA10, CACNG4, CACNB3, CACNG2, CACNA2D2 |
| rno04925:Aldosterone synthesis and secretion | 11 | 0.05428 | PRKD1, CREB1, ADCY5, MC2R, NR4A2, CREB3L1, CREB5, PLCB1, PRKCE, KCNK3, PRKCB |
| rno05414:Dilated cardiomyopathy | 11 | 0.05428 | ITGB8, PLN, ADCY5, DAG1, LMNA, CACNB1, ITGA10, CACNG4, CACNB3, CACNG2, CACNA2D2 |
| rno05222:Small cell lung cancer | 11 | 0.06594 | CCNE2, CCND1, E2F3, BCL2, CYCS, TP53, NFKBIA, CDK6, RARB, MYC, PIK3R2 |
| rno00564:Glycerophospholipid metabolism | 11 | 0.09898 | CDS2, GPD2, DGKB, PLA2G15, LPCAT1, LCLAT1, PLA2G6, DGKZ, DGKI, PLA2G2F, PLA2G4D |
| rno05030:Cocaine addiction | 10 | 0.00307 | GNAI2, DRD2, CREB1, ADCY5, MAOA, SLC6A3, TH, CREB3L1, CREB5, FOSB |
| rno05223:Non-small cell lung cancer | 10 | 0.01164 | CCND1, E2F3, PLCG1, MAP2K1, TP53, TGFA, CDK6, RARB, PRKCB, PIK3R2 |
| rno05212:Pancreatic cancer | 10 | 0.02403 | CCND1, E2F3, MAP2K1, VEGFA, TP53, SMAD4, TGFA, CDK6, RALGDS, PIK3R2 |
| rno04918:Thyroid hormone synthesis | 10 | 0.03729 | FXYD2, CREB1, ADCY5, GPX3, CREB3L1, CREB5, GPX8, ATP1A2, PLCB1, PRKCB |
| rno04115:p53 signaling pathway | 10 | 0.04044 | CCNE2, EI24, CCND1, SERPINE1, CYCS, TP53, CDK6, MDM4, IGFBP3, TP73 |
| rno05412:Arrhythmogenic right ventricular cardiomyopathy (ARVC) | 10 | 0.04728 | ITGB8, DAG1, LMNA, CACNB1, LEF1, ITGA10, CACNG4, CACNB3, CACNG2, CACNA2D2 |
| rno05220:Chronic myeloid leukemia | 10 | 0.06315 | CCND1, E2F3, MAP2K1, HDAC1, TP53, SMAD4, NFKBIA, CDK6, MYC, PIK3R2 |
| rno05100:Bacterial invasion of epithelial cells | 10 | 0.09266 | CAV3, SEPT3, WASF1, MET, GAB1, SEPT6, SRC, ELMO1, VCL, PIK3R2 |
| rno04330:Notch signaling pathway | 9 | 0.02167 | NOTCH3, NOTCH2, NOTCH1, APH1A, HDAC1, DLL1, JAG1, NCOR2, NUMBL |
| rno04370:VEGF signaling pathway | 9 | 0.04631 | PLCG1, MAP2K1, MAPK13, VEGFA, PPP3R1, SRC, PLA2G4D, PRKCB, PIK3R2 |
| rno04730:Long-term depression | 9 | 0.05453 | CRHR1, GNAO1, NOS1, GNAI2, MAP2K1, GUCY1A3, PLCB1, PLA2G4D, PRKCB |
| rno05216:Thyroid cancer | 7 | 0.01129 | CCND1, RET, MAP2K1, PPARG, TP53, LEF1, MYC |
| rno05219:Bladder cancer | 7 | 0.04411 | CCND1, E2F3, MAP2K1, VEGFA, TP53, MYC, SRC |
| rno04960:Aldosterone-regulated sodium reabsorption | 7 | 0.05436 | FXYD2, SCNN1G, NEDD4L, ATP1A2, IRS1, PRKCB, PIK3R2 |
| rno04320:Dorso-ventral axis formation | 6 | 0.02332 | NOTCH3, NOTCH2, NOTCH1, MAP2K1, CPEB2, CPEB3 |
| rno04710:Circadian rhythm | 6 | 0.04755 | CRY2, CREB1, PRKAG1, PER2, RORA, CLOCK |

## S2E Table: KEGG_Pathways-miR351

| KEGG_PATHWAY | Gene Count | p_value | Genes |
| --- | --- | --- | --- |
| rno01100:Metabolic pathways | 104 | 0.02131 | CYP24A1, CDIPT, IMPAD1, HMGCR, B3GALT4, ITPKB, PI4K2B, NMRK2, ACSS1, PGP, PIP5KL1, SCLY, MAT1A, DCTPP1, SGPL1, CYP1A1, TAT, LPCAT4, DLD, LCLAT1, ABAT, PLA2G2D, ENPP1, ACSBG2, PPT2, KMO, PPT1, CERS3, PPCDC, PPAT, TK2, QRSL1, THTPA, GALNS, ALDH4A1, B4GALT6, GCNT1, MOCS1, B4GALT1, MGAT4A, ST6GAL1, B4GAT1, ACER2, ACACB, GLYCTK, GGT7, MLYCD, GLS, AGXT2, ALG12, ALG13, COX11, PTGES2, ALG1, CYP11B2, MAN1B1, PIP5K1C, ANPEP, CDS2, GSS, ST6GALNAC6, PLCB3, NT5M, IPMK, NT5E, SARDH, ACADS, PIK3C2B, POLR1A, MAN1A1, LPIN2, PMM2, UGT2B17, DGAT1, H6PD, PANK1, RRM2, PLA2G5, BCAT1, SORD, GALNT7, FUT7, HK2, ATP5G2, PFAS, MTMR3, ALDH1A3, FUT4, FUT1, PAFAH1B1, UCK1, UCK2, TSTA3, MTMR6, BDH1, ACSL6, B4GALNT1, GALNT14, PLA2G16, NDUFA2, DGKQ, GANC, DPM2, ALOX5 |
| rno04010:MAPK signaling pathway | 37 | 0.00002 | FGFR2, FGF5, FGFR1, FGF14, FGF9, MKNK2, PPM1A, CACNB1, ELK1, PPP3R2, CACNB3, MAPKAPK2, SRF, MAP3K3, MAP3K1, TRAF6, MAP2K7, NFATC1, LAMTOR3, NTF4, TAOK1, TP53, RAF1, CACNG2, DUSP3, RPS6KA1, RASGRF2, MAPK12, RASGRF1, ARRB1, MAPK14, IKBKG, MAP3K13, DUSP7, CACNA1B, MAP3K11, DUSP6 |
| rno05200:Pathways in cancer | 37 | 0.03796 | FGFR2, FGF5, E2F2, FGFR1, ADCY1, E2F3, FGF14, FGF9, ERBB2, MMP2, PLCB3, CDKN2A, CDKN2B, BCL2, PIK3R5, TRAF6, APC, COL4A3, DVL3, TCF7, RET, ARHGEF1, PIK3CD, SMAD4, TP53, RAF1, SMAD2, CDK6, APPL1, STAT3, SMO, LAMA4, VEGFA, IKBKG, TCEB2, LAMC2, ABL1 |
| rno04151:PI3K-Akt signaling pathway | 34 | 0.01823 | FGFR2, FGFR1, FGF5, MCL1, FGF9, FGF14, EFNA2, PPP2R5C, ITGB3, EIF4EBP1, BCL2, PPP2CA, PIK3R5, PRKAA2, COL11A2, ANGPT2, EPO, PPP2R1B, COL4A3, IL2RB, CREB1, PIK3CD, TP53, RAF1, CDK6, IL6R, EPHA2, ITGA9, YWHAG, LAMA4, IKBKG, ITGA7, VEGFA, LAMC2 |
| rno04014:Ras signaling pathway | 30 | 0.00071 | FGFR2, FGF5, FGFR1, FGF9, FGF14, EFNA2, ELK1, RASAL2, RASAL1, REL, GAB2, RAPGEF5, PIK3R5, RASA3, ANGPT2, PLA2G16, PIK3CD, GRIN2A, RAF1, EPHA2, KSR2, RASGRF2, ETS1, RASGRF1, IKBKG, VEGFA, ABL1, ABL2, PLA2G2D, PLA2G5 |
| rno05206:MicroRNAs in cancer | 28 | 0.00000 | SLC45A3, CYP24A1, E2F2, E2F3, MCL1, ERBB3, ERBB2, DICER1, BMPR2, BCL2L2, ITGB3, TRIM71, BAK1, CDKN2A, BCL2, BMF, APC, TP53, RAF1, UBE2I, CDK6, CDC25A, STAT3, NOTCH2, SLC7A1, GLS, VEGFA, ABL1 |
| rno04144:Endocytosis | 28 | 0.04340 | FGFR2, USP8, ERBB4, CYTH1, ERBB3, PIP5K1C, VPS37B, ADRBK1, CYTH2, VPS37C, PIP5KL1, VPS4B, WIPF2, EHD1, TRAF6, IQSEC3, VPS36, IQSEC2, IL2RB, RET, SMAD2, CCR5, ARRB1, ARF3, IST1, RAB22A, SNX32, SMURF1 |
| rno04550:Signaling pathways regulating pluripotency of stem cells | 23 | 0.00014 | FGFR2, FGFR1, DVL3, JARID2, PIK3CD, BMPR2, SMAD4, LIFR, RAF1, SMAD2, REST, STAT3, ACVR1C, LIF, MAPK12, ID2, ID1, MAPK14, PCGF6, PIK3R5, ID3, BMPR1B, APC |
| rno04810:Regulation of actin cytoskeleton | 22 | 0.05711 | FGFR2, FGFR1, FGF5, VAV3, ARHGEF1, FGF9, FGF14, LIMK1, PPP1R12B, PIK3CD, RAF1, PIP5K1C, ITGB3, MYL9, ITGA9, PPP1CA, CHRM5, ITGA7, CYFIP2, PIK3R5, APC, PIP4K2B |
| rno05169:Epstein-Barr virus infection | 22 | 0.07728 | PIK3CD, TP53, STAT3, HDAC5, YWHAG, CSNK2A1, PSMD13, MAPK12, IL10RA, BCL2, MAPK14, IFNG, IKBKG, HSPB2, PIK3R5, ENTPD3, TNFAIP3, TRAF6, ENTPD1, MAP2K7, PSMD7, NCOR2 |
| rno04261:Adrenergic signaling in cardiomyocytes | 20 | 0.00379 | PPP2R1B, ADCY1, CREB1, PIK3CD, PPP2R5C, ATP1B4, CACNB1, CACNB2, CACNB3, CACNG2, PPP1CA, PLCB3, ATP2B3, MAPK12, BCL2, MAPK14, PPP2CA, SCN4B, PIK3R5, SCN5A |
| rno05205:Proteoglycans in cancer | 20 | 0.07283 | FGFR1, ARHGEF1, ERBB4, ERBB3, ERBB2, PIK3CD, TP53, RAF1, ELK1, ITGB3, MMP2, STAT3, SMO, PPP1CA, MAPK12, MAPK14, VEGFA, HSPB2, PIK3R5, GPC1 |
| rno04921:Oxytocin signaling pathway | 19 | 0.02024 | ADCY1, PPP1R12B, PIK3CD, CACNB1, RAF1, CACNB2, PPP3R2, ELK1, CACNB3, CACNG2, KCNJ12, MYL9, PPP1CA, PLCB3, KCNJ9, NFATC4, PIK3R5, PRKAA2, NFATC1 |
| rno04141:Protein processing in endoplasmic reticulum | 19 | 0.02819 | SEC31B, SYVN1, PDIA3, UBE2G1, MAN1B1, UBE2J1, MAN1A1, EDEM1, BAK1, ATXN3, BCL2, DNAJB2, SVIP, YOD1, SAR1B, MAP2K7, SSR2, SEL1L, SEC61A2 |
| rno04722:Neurotrophin signaling pathway | 18 | 0.00415 | NTF4, PIK3CD, TP53, RAF1, MAPKAPK2, TP73, MAPK12, MAP3K3, RPS6KA1, MAPK14, MAP3K1, BCL2, SORT1, SH2B3, PIK3R5, ABL1, TRAF6, MAP2K7 |
| rno05161:Hepatitis B | 18 | 0.01030 | MAVS, E2F2, E2F3, CREB1, PIK3CD, TP53, SMAD4, RAF1, ELK1, CDK6, STAT3, MYD88, MAP3K1, BCL2, IKBKG, NFATC4, PIK3R5, NFATC1 |
| rno04390:Hippo signaling pathway | 18 | 0.02485 | PPP2R1B, DVL3, TCF7, SMAD4, BMPR2, SMAD2, TP73, FRMD1, AJUBA, PPP1CA, YWHAG, ID2, BBC3, CRB2, ID1, PPP2CA, BMPR1B, APC |
| rno04020:Calcium signaling pathway | 18 | 0.09672 | ADCY1, SLC8A2, ERBB4, ERBB3, ERBB2, TACR1, PHKA1, GRIN2A, PPP3R2, ITPKB, PPIF, P2RX4, P2RX7, CHRM5, PLCB3, ATP2B3, P2RX6, CACNA1B |
| rno04022:cGMP-PKG signaling pathway | 17 | 0.08817 | ADCY1, SLC8A2, CREB1, PIK3CD, ATP1B4, RAF1, PPP3R2, SRF, MYL9, PPIF, MEF2D, PPP1CA, PLCB3, ATP2B3, NFATC4, PIK3R5, NFATC1 |
| rno04070:Phosphatidylinositol signaling system | 16 | 0.00164 | IMPAD1, CDIPT, DGKQ, PIK3C2B, PIK3CD, PIP5K1C, ITPKB, PI4K2B, CDS2, MTMR3, PLCB3, PIK3R5, IP6K1, MTMR6, IPMK, PIP4K2B |
| rno05145:Toxoplasmosis | 16 | 0.01677 | PIK3CD, STAT3, PPIF, LAMA4, MYD88, CCR5, MAPK12, IL10RA, MAPK14, BCL2, IFNG, IKBKG, LAMC2, PIK3R5, ALOX5, TRAF6 |
| rno05162:Measles | 16 | 0.03160 | MAVS, IL2RB, TACR1, PIK3CD, TP53, CDK6, STAT3, TP73, DOK1, MYD88, CSNK2A1, BBC3, IFNG, PIK3R5, TNFAIP3, TRAF6 |
| rno04310:Wnt signaling pathway | 16 | 0.04658 | DVL3, TCF7, VANGL1, VANGL2, TP53, SMAD4, PPP3R2, DAAM2, PORCN, GPC4, SFRP5, PLCB3, CSNK2A1, NFATC4, APC, NFATC1 |
| rno04071:Sphingolipid signaling pathway | 15 | 0.03673 | PPP2R1B, SGPL1, PLCB3, MAPK12, GAB2, BCL2, MAPK14, ACER2, PPP2CA, PPP2R5C, PIK3CD, TP53, RAF1, PIK3R5, CERS3 |
| rno05160:Hepatitis C | 15 | 0.04615 | MAVS, PPP2R1B, PPARA, PIK3CD, TP53, RAF1, STAT3, MAPK12, PPP2CA, MAPK14, IKBKG, IRF1, SCARB1, PIK3R5, TRAF6 |
| rno04360:Axon guidance | 15 | 0.04615 | PLXNA1, LIMK1, ABLIM3, EFNA2, PPP3R2, EPHA2, SEMA4F, EPHA8, SEMA4C, SEMA4B, NFATC4, SEMA4D, UNC5C, ABL1, SRGAP2 |
| rno05212:Pancreatic cancer | 14 | 0.00022 | E2F2, E2F3, ERBB2, PIK3CD, TP53, SMAD4, RAF1, CDK6, SMAD2, STAT3, CDKN2A, VEGFA, IKBKG, PIK3R5 |
| rno04350:TGF-beta signaling pathway | 14 | 0.00397 | PPP2R1B, SMAD4, BMPR2, SMAD2, ACVR1C, SP1, CDKN2B, ID2, ID1, PPP2CA, IFNG, ID3, SMURF1, BMPR1B |
| rno04066:HIF-1 signaling pathway | 14 | 0.02074 | ERBB2, PIK3CD, MKNK2, HK2, IL6R, STAT3, EIF4EBP1, BCL2, VEGFA, IFNG, TCEB2, PIK3R5, ANGPT2, EPO |
| rno00240:Pyrimidine metabolism | 14 | 0.02563 | PNPT1, POLR1A, TK2, NT5M, RRM2, TXNRD3, ENTPD3, UCK1, TXNRD1, UCK2, ENTPD4, ENTPD1, NT5E, DCTPP1 |
| rno04152:AMPK signaling pathway | 14 | 0.07374 | PPP2R1B, EIF4EBP1, AKT1S1, MLYCD, HMGCR, SCD, CREB1, PPP2CA, PPP2R5C, PIK3CD, ELAVL1, PIK3R5, PRKAA2, TBC1D1 |
| rno04110:Cell cycle | 14 | 0.08134 | E2F2, E2F3, CDC14B, SMAD4, TP53, CDK6, SMAD2, CDC25A, YWHAG, CDKN2A, CDKN2B, ORC4, ABL1, ORC2 |
| rno00562:Inositol phosphate metabolism | 13 | 0.00303 | IMPAD1, CDIPT, PIK3C2B, PIK3CD, PIP5K1C, ITPKB, PI4K2B, MTMR3, PLCB3, PIP5KL1, MTMR6, IPMK, PIP4K2B |
| rno05215:Prostate cancer | 13 | 0.01147 | FGFR2, E2F2, FGFR1, E2F3, TCF7, ERBB2, BCL2, CREB1, IKBKG, PIK3CD, TP53, RAF1, PIK3R5 |
| rno05222:Small cell lung cancer | 13 | 0.01252 | COL4A3, E2F2, E2F3, LAMA4, CDKN2B, BCL2, IKBKG, PIK3CD, TP53, PIK3R5, LAMC2, CDK6, TRAF6 |
| rno03015:mRNA surveillance pathway | 13 | 0.01896 | PPP2R1B, SMG5, PPP2R5C, SMG1, NXF1, PAPOLB, PPP1CA, PAPOLA, RBM8A, PPP2CA, MSI1, MAGOHB, CPSF6 |
| rno05142:Chagas disease (American trypanosomiasis) | 13 | 0.05287 | PPP2R1B, ADCY1, PIK3CD, SMAD2, PLCB3, MYD88, MAPK12, PPP2CA, MAPK14, IKBKG, IFNG, PIK3R5, TRAF6 |
| rno05218:Melanoma | 12 | 0.00566 | FGF5, E2F2, FGFR1, E2F3, CDKN2A, FGF9, FGF14, PIK3CD, TP53, RAF1, PIK3R5, CDK6 |
| rno05220:Chronic myeloid leukemia | 12 | 0.01060 | E2F2, E2F3, CDKN2A, GAB2, PIK3CD, IKBKG, TP53, SMAD4, RAF1, PIK3R5, CDK6, ABL1 |
| rno04012:ErbB signaling pathway | 12 | 0.03944 | EIF4EBP1, NRG3, ERBB4, ERBB3, ERBB2, PIK3CD, RAF1, ELK1, PIK3R5, ABL1, ABL2, MAP2K7 |
| rno00564:Glycerophospholipid metabolism | 12 | 0.04818 | CDS2, CDIPT, ACHE, PLA2G16, DGKQ, TAZ, LCLAT1, LYPLA2, LPIN2, PLA2G2D, LPCAT4, PLA2G5 |
| rno05230:Central carbon metabolism in cancer | 11 | 0.00940 | FGFR2, SLC1A5, FGFR1, RET, ERBB2, GLS, PIK3CD, TP53, HK2, RAF1, PIK3R5 |
| rno05210:Colorectal cancer | 10 | 0.02551 | TCF7, BCL2, PIK3CD, TP53, SMAD4, RAF1, PIK3R5, SMAD2, APPL1, APC |
| rno00510:N-Glycan biosynthesis | 9 | 0.01499 | B4GALT1, MGAT4A, ST6GAL1, ALG1, MAN1B1, DPM2, MAN1A1, ALG12, ALG13 |
| rno05223:Non-small cell lung cancer | 9 | 0.03147 | E2F2, E2F3, CDKN2A, ERBB2, PIK3CD, TP53, RAF1, PIK3R5, CDK6 |
| rno04520:Adherens junction | 9 | 0.09387 | PTPRB, FGFR1, TCF7, CSNK2A1, ERBB2, PVRL2, SMAD4, SMAD2, YES1 |
| rno00514:Other types of O-glycan biosynthesis | 8 | 0.00302 | B4GALT1, ST6GAL1, FUT7, B3GALT4, B3GLCT, FUT4, POFUT2, LFNG |
| rno05219:Bladder cancer | 8 | 0.01357 | E2F2, E2F3, CDKN2A, ERBB2, VEGFA, TP53, RAF1, MMP2 |
| rno05213:Endometrial cancer | 8 | 0.05627 | TCF7, ERBB2, PIK3CD, TP53, RAF1, ELK1, PIK3R5, APC |
| rno05014:Amyotrophic lateral sclerosis (ALS) | 8 | 0.07210 | TNFRSF1B, MAPK12, BCL2, MAPK14, TP53, GRIN2A, TOMM40, PPP3R2 |
| rno05340:Primary immunodeficiency | 6 | 0.08104 | DCLRE1C, CD8A, RFX5, TAP2, IKBKG, RFXANK |

## S2E Table: KEGG_Pathways-miR490*

| KEGG_PATHWAY | Gene Count | p_value | Genes |
| --- | --- | --- | --- |
| rno04921:Oxytocin signaling pathway | 8 | 0.01574 | PRKAG3, FOS, ROCK1, PRKAG2, PIK3CA, GUCY1A3, KCNJ2, PLCB1 |
| rno04014:Ras signaling pathway | 8 | 0.08415 | FGF5, RAB5B, RASGRF2, ETS1, TBK1, FGF14, HTR7, PIK3CA |
| rno00230:Purine metabolism | 7 | 0.07762 | POLD4, ADSS, NT5C2, PDE3B, GUCY1A3, PDE8B, PAICS |
| rno05146:Amoebiasis | 6 | 0.03628 | C8A, COL4A3, C9, RAB5B, PIK3CA, PLCB1 |
| rno04360:Axon guidance | 6 | 0.05843 | EPHA4, UNC5B, ROCK1, ROBO1, SEMA3A, EPHA3 |
| rno04924:Renin secretion | 5 | 0.02372 | PDE3B, GUCY1A3, KCNJ2, PLCB1, ADCYAP1 |
| rno04918:Thyroid hormone synthesis | 5 | 0.02489 | SLC26A4, ATP1B1, GPX5, PAX8, PLCB1 |
| rno05132:Salmonella infection | 5 | 0.04669 | RILP, FOS, ROCK1, TLR5, DYNC1I2 |
| rno04915:Estrogen signaling pathway | 5 | 0.07202 | OPRM1, FOS, PIK3CA, ESR2, PLCB1 |
| rno04922:Glucagon signaling pathway | 5 | 0.08098 | PRKAG3, PRKAG2, PDE3B, GYS2, PLCB1 |
| rno04710:Circadian rhythm | 4 | 0.01258 | PRKAG3, PRKAG2, PER3, FBXL3 |

## S2F Table: Comparison of KEGG_Pathways between all the miRs

| Name of KEGG_Pathway | miR-34a | miR-351 | miR-490* |
| --- | --- | --- | --- |
| rno04360:Axon guidance | x | x | x |
| rno04921:Oxytocin signaling pathway | x | x | x |
| rno04014:Ras signalling pathway | x | x | x |
| rno04918:Thyroid hormone synthesis | x |  | x |
| rno04710:Circadian rhythm | x |  | x |
| rno04915:Estrogen signaling pathway | x |  | x |
| rno05206:MicroRNAs in cancer | x | x |  |
| rno04071:Sphingolipid signaling pathway | x | x |  |
| rno05215:Prostate cancer | x | x |  |
| rno05200:Pathways in cancer | x | x |  |
| rno04520:Adherens junction | x | x |  |
| rno05230:Central carbon metabolism in cancer | x | x |  |
| rno04261:Adrenergic signaling in cardiomyocytes | x | x |  |
| rno04144:Endocytosis | x | x |  |
| rno05218:Melanoma | x | x |  |
| rno05205:Proteoglycans in cancer | x | x |  |
| rno04022:cGMP-PKG signaling pathway | x | x |  |
| rno04152:AMPK signaling pathway | x | x |  |
| rno05210:Colorectal cancer | x | x |  |
| rno05161:Hepatitis B | x | x |  |
| rno04070:Phosphatidylinositol signaling system | x | x |  |
| rno05223:Non-small cell lung cancer | x | x |  |
| rno05212:Pancreatic cancer | x | x |  |
| rno04010:MAPK signaling pathway | x | x |  |
| rno04310:Wnt signaling pathway | x | x |  |
| rno05219:Bladder cancer | x | x |  |
| rno04151:PI3K-Akt signaling pathway | x | x |  |
| rno05220:Chronic myeloid leukemia | x | x |  |
| rno05222:Small cell lung cancer | x | x |  |
| rno04722:Neurotrophin signaling pathway | x | x |  |
| rno04110:Cell cycle | x | x |  |
| rno00564:Glycerophospholipid metabolism | x | x |  |
| rno00230:Purine metabolism |  |  | x |
| rno04924:Renin secretion |  |  | x |
| rno05146:Amoebiasis |  |  | x |
| rno05132:Salmonella infection |  |  | x |
| rno04922:Glucagon signaling pathway |  |  | x |
| rno04550:Signaling pathways regulating pluripotency of stem cells |  | x |  |
| rno00514:Other types of O-glycan biosynthesis |  | x |  |
| rno00562:Inositol phosphate metabolism |  | x |  |
| rno04350:TGF-beta signaling pathway |  | x |  |
| rno00510:N-Glycan biosynthesis |  | x |  |
| rno05145:Toxoplasmosis |  | x |  |
| rno03015:mRNA surveillance pathway |  | x |  |
| rno04066:HIF-1 signaling pathway |  | x |  |
| rno01100:Metabolic pathways |  | x |  |
| rno04390:Hippo signaling pathway |  | x |  |
| rno00240:Pyrimidine metabolism |  | x |  |
| rno04141:Protein processing in endoplasmic reticulum |  | x |  |
| rno05162:Measles |  | x |  |
| rno04012:ErbB signaling pathway |  | x |  |
| rno05160:Hepatitis C |  | x |  |
| rno05142:Chagas disease (American trypanosomiasis) |  | x |  |
| rno05213:Endometrial cancer |  | x |  |
| rno04810:Regulation of actin cytoskeleton |  | x |  |
| rno05014:Amyotrophic lateral sclerosis (ALS) |  | x |  |
| rno05169:Epstein-Barr virus infection |  | x |  |
| rno05340:Primary immunodeficiency |  | x |  |
| rno04020:Calcium signaling pathway |  | x |  |
| rno04919:Thyroid hormone signaling pathway | x |  |  |
| rno04015:Rap1 signaling pathway | x |  |  |
| rno05030:Cocaine addiction | x |  |  |
| rno05031:Amphetamine addiction | x |  |  |
| rno04916:Melanogenesis | x |  |  |
| rno05214:Glioma | x |  |  |
| rno04750:Inflammatory mediator regulation of TRP channels | x |  |  |
| rno05216:Thyroid cancer | x |  |  |
| rno04911:Insulin secretion | x |  |  |
| rno04540:Gap junction | x |  |  |
| rno05231:Choline metabolism in cancer | x |  |  |
| rno04728:Dopaminergic synapse | x |  |  |
| rno04330:Notch signaling pathway | x |  |  |
| rno04320:Dorso-ventral axis formation | x |  |  |
| rno04970:Salivary secretion | x |  |  |
| rno04917:Prolactin signaling pathway | x |  |  |
| rno04060:Cytokine-cytokine receptor interaction | x |  |  |
| rno04115:p53 signaling pathway | x |  |  |
| rno05410:Hypertrophic cardiomyopathy (HCM) | x |  |  |
| rno05032:Morphine addiction | x |  |  |
| rno04370:VEGF signaling pathway | x |  |  |
| rno05412:Arrhythmogenic right ventricular cardiomyopathy (ARVC) | x |  |  |
| rno04925:Aldosterone synthesis and secretion | x |  |  |
| rno05414:Dilated cardiomyopathy | x |  |  |
| rno04960:Aldosterone-regulated sodium reabsorption | x |  |  |
| rno04730:Long-term depression | x |  |  |
| rno04270:Vascular smooth muscle contraction | x |  |  |
| rno05100:Bacterial invasion of epithelial cells | x |  |  |

## S2G Table: GOTERMS_BP-miR34a

| Term | Count | % | PValue | Genes | Fold Enrichment | Bonferroni | Benjamini | FDR |
| --- | --- | --- | --- | --- | --- | --- | --- | --- |
| GO:0023051~regulation of signaling | 302 | 22.14076246 | 3.83E-19 | SYT1, ADCY5, SNCA, LHCGR, SYT9, LPAR2, RORA, SIDT2, ITSN1, SHKBP1, S1PR2, DDX17, CRY2, GAB1, SERPINE1, CREB3L1, DAB2IP, STRN3, PTPRR, RAD9A, UBR1, RIC8B, PPARGC1B, FARP2, CRHR1, HNF4A, SERPINF2, VEGFA, MNT, PDGFRA, PDGFRB, ATPIF1, RALGPS2, PPP2R3A, RALGPS1, GCNT2, GNAI2, HOXA13, CRTC1, DAG1, NFKBIA, UBA5, BDKRB2, GPR143, MYT1, RIMS4, EPHB2, RIMS3, PEA15, TNKS, ASXL2, BMP3, GPR158, ARHGEF33, ABR, CAMLG, MAP2K1, IL1RN, SMAD4, MSTN, SKI, HGF, GAS1, EPHA4, NOTCH2, EI24, DLX1, NOTCH1, TRPS1, GRK6, TMOD2, RGS6, KLF4, BMP8B, OPRM1, CREBRF, LZTS2, GFAP, JPH3, HNF1A, CPEB3, PPARG, SHOC2, ZEB1, TKFC, MAPT, TGFA, GPHB5, MYC, AGAP2, ALS2CL, ARHGEF3, PGAP2, STX1A, FLOT2, TP53, CACNG4, CFTR, ARHGEF9, CACNG2, NFAM1, IL6R, MYADM, LRPAP1, INHBB, ZDHHC17, CCND1, MYRIP, MAP3K15, KSR2, SEMA4C, PLA2G6, MAP3K14, KSR1, CLOCK, ZMYND11, RAB3B, RAP1GAP, CCR1, FKBP1A, KIT, FKBP1B, NETO1, PLEKHG2, CCL22, XBP1, BCL2, LRRTM2, PER2, BCL6, BCL9L, RASA4, MLLT3, NOS1, AXL, LMNA, DGKI, MID1, TMPRSS6, SNAI1, TP73, CDH13, DUSP4, DKK1, NEUROD2, DGKZ, HTR2C, LRP4, TP53INP1, HTR2A, SLC44A2, KCNC3, SLC6A1, TNFSF14, ILDR1, JAG1, CXCL11, CALB1, CXCL10, NLRC5, WNT1, SLC16A1, PTGIS, MYOCD, ANK2, MDFIC, GATA3, RRAS, SPRED1, LGI1, RNF34, DISC1, PID1, RET, STMN3, PSD3, SIX3, FGF23, DLL1, SOCS4, IRS1, MAP4K4, UCN2, RELT, GPR55, TRIM32, AKAP6, RELN, VAMP2, GLP1R, CAV3, NETO2, SYVN1, DRD2, STRAP, SIPA1, ONECUT2, SOX4, SNX4, ULK4, GREM2, SRC, IRAK4, PEG10, AMER1, PPP1R16B, ECE1, TRIM67, OVOL2, RNF165, GRID2IP, AXIN2, BMF, FGD6, FGD3, CSF1R, DIXDC1, MYO1C, MET, ARPP19, NR4A2, CACNA2D2, TRIM62, FOXP1, CDKN1C, IKBKE, UACA, HDAC1, NEDD4, CDC42SE2, CACNA1E, NAT8L, BCAN, PDCD4, LGR4, SPRY3, PRMT2, RSPO1, HPSE, WWC3, LTF, PLCB1, CASP2, IRAK2, ANKS1A, CNTNAP4, PADI2, PRKCH, LEF1, PFKM, EDAR, DAPK2, PRKCE, ARHGAP26, PRKCB, PRKD1, MFN2, PRKCQ, ACVR2B, NCOA1, UBE2K, CNTN2, PSME3, FAIM2, KMT2D, CPLX2, RTN4RL1, CTNND2, CYTH4, PPM1A, CTNND1, KITLG, CALCA, SHISA7, MOAP1, TSPAN33, RASGRP4, MARVELD3, TOR1A, SYN2, NFATC4, INPP5D, THPO, PTPRD, DNM1L, NCDN, CREB1, TSPAN14, MAL, RCAN1, TMBIM1, SIRT1, WNT2B, RDH11, PPP1R9B, ATXN7, HEYL, SMPD1, IRF1, IRF4, IGFBP3, RNF41 | 1.609079284 | 2.92E-15 | 2.92E-15 | 7.61E-16 |
| GO:0009893~positive regulation of metabolic process | 354 | 25.95307918 | 2.13E-16 | PPP2R5A, SNCA, LHCGR, LPAR2, RORA, ITSN1, S1PR2, DDX17, GAB1, SERPINE1, CREB3L1, RARB, DAB2IP, STRN3, YY1, RAP1GDS1, HNF4G, MYH9, RIC8B, PPARGC1B, FARP2, CRHR1, MTF1, HNF4A, SERPINF2, MCIDAS, VEGFA, MC2R, PDGFRA, PDGFRB, ATPIF1, NEK5, SUPT6H, SUCO, RALGPS2, PPP2R3A, GCNT2, RALGPS1, HOXA13, GNAI2, CRTC1, NFKBIA, MYT1, RIMS3, PLAGL1, ACE, LPCAT1, SERINC1, TNKS, HIP1, ASXL2, PLAG1, KLF6, UNC119, BMP3, ARHGEF33, ESRRA, ABR, MAP2K1, IL1RN, SMAD4, MSTN, SKI, OXSR1, HGF, USF1, FAM131B, KCNK3, NOTCH3, EPHA4, NOTCH1, NR1I2, CSRNP3, DCP1A, TRPS1, PKP4, RGS6, RBMXL1, MARCKS, AREG, FOXI1, TCF12, KLF4, NCOR2, BMP8B, CREBRF, OPRM1, HNF1A, CRNKL1, CPEB3, PPARG, MITF, ZEB1, NMUR1, TGFA, GUCY1A3, MKX, GPIHBP1, MYC, AGAP2, ALS2CL, ARHGEF3, ANP32B, RAB4A, TP53, ERLIN1, ARHGEF9, SPEN, NFAM1, IL6R, FOSB, ZCCHC17, MYCN, INHBB, CCND1, KSR2, MAP3K15, DNAJC24, CLIC5, SLC26A9, SEMA4C, WNT9B, PLA2G6, KSR1, NSD1, CLOCK, SRGAP2, RAP1GAP, CCR1, UNG, ATG13, HK1, FKBP1A, KIT, GCH1, PLEKHG2, CCL22, ACSL1, MPV17L2, XBP1, BCL2, BCL11B, CHM, BCL6, BCL9L, RASA4, ELMOD1, TBC1D2, NOS1, LMNA, MID1, ATMIN, BRAT1, TMPRSS6, SNAI1, TP73, SGTA, TRIM21, PREB, CDH13, RPS6KA4, PHF19, PKNOX1, OBFC1, MEX3C, KDM8, NEUROD2, SVIP, HTR2C, TP53INP2, LRP4, HTR2A, TP53INP1, NCBP2, RNASEL, SLC6A1, SRCIN1, RASGEF1C, JAG1, CXCL11, CXCL10, NLRC5, WNT1, MYOCD, ATG5, ANK2, MDFIC, ANK3, GATA3, MS4A2, DISC1, SAMD4A, PID1, RET, SATB2, MTA2, SIX3, PSD3, FGF23, SOCS4, DLL1, GRHL2, IRS1, SGSM2, RELT, GPR55, TRIM32, CELF3, AKAP6, NFE2L1, RELN, NGB, CLIP3, GLP1R, CAMTA1, SNX9, FZR1, DRD2, SIPA1, ONECUT2, SOX4, LIN28A, SRC, NR2C2, OAZ2, SLX4, AMER1, PPP1R16B, ECE1, TRIM67, GMIP, OVOL2, ITGB8, TBC1D30, AXIN2, FGD6, FGD3, CSF1R, DIXDC1, MET, DOCK9, ARPP19, NR4A2, WHSC1, DOCK8, FOXP3, TRIM62, FOXP1, DOCK3, CORO1C, TBC1D25, CDKN1C, HSPBP1, UACA, AKTIP, HDAC1, NEDD4, GTF2F1, ZIC5, DNAJB2, DNAJB1, E2F3, ARID4A, PRKAG1, ARID4B, RHOQ, PEX3, PIP5K1A, CBFA2T3, LGR4, TMEM173, MAZ, PRMT2, RSPO1, PAX7, ARHGAP1, RTF1, LTF, LRRFIP1, PLCB1, FOSL1, IRAK2, TBL1XR1, KDM7A, CCDC88A, FOXJ2, NOS1AP, CYCS, CHST3, PADI2, PRKCH, LEF1, CDK6, EDAR, PRKCE, DDN, ARHGAP26, PRKCB, PRKD1, PRKCQ, ACVR2B, NCOA1, DGAT1, PSME1, UBE2K, ATG4B, CNTN2, MYRF, PSME3, TREM1, CUX1, CALCR, ABLIM1, KMT2D, APH1A, ABLIM3, CYTH4, PPP3R1, PPM1A, CTNND1, KITLG, CTCFL, CALCOCO2, CALCA, CALCB, RGMB, ARHGAP44, RASGRP4, POU2F1, CHD1, NFATC4, NEDD4L, PAFAH1B2, TNRC6B, EHD4, PIK3R2, THPO, GNAO1, CREB1, MAP1A, CREB5, SIRT1, ATXN7L3, RALGDS, PRLR, ATXN7, HEYL, SMPD1, IRF1, KDM4C, IRF4, EVI5L, IGFBP3, RNF41 | 1.47705134 | 1.69E-12 | 8.47E-13 | 4.44E-13 |
| GO:0051128~regulation of cellular component organization | 256 | 18.76832845 | 6.65E-15 | DYNC1LI1, SYT1, CHMP3, PPP2R5A, ADCY5, SNCA, CD47, RAB29, SERPINE1, VPS4A, LRRC7, RAB21, DBNL, DAB2IP, GBP5, ROCK1, MYH9, FARP2, OGFOD1, UHRF2, HNF4A, SERPINF2, VEGFA, SURF4, PDGFRB, ATPIF1, ADD2, SUPT6H, ALDOA, GCNT2, HOXA13, CRTC1, DAG1, GPR143, RIMS3, EPHB2, ACE, PVRL1, CDA, NAT10, TNKS, HIP1, ASXL2, UNC119, ABR, MAP2K1, SMAD4, MSTN, CDC23, HGF, GAS1, KCNK3, FAM131B, SEMA6A, EPHA4, EI24, NOTCH1, TRPS1, TMOD2, MARCKS, KLF4, ARSB, GFAP, LIMA1, HNF1A, CRNKL1, CPEB3, WASF1, PPARG, SYNJ1, MITF, VPS37B, XYLT1, NMUR1, MAPT, SEMA3E, ANP32A, EEF2K, TGFA, MYC, FMNL3, STX1A, ZDHHC8, RAB4A, TP53, MYADM, ZCCHC17, ANKRD23, SEMA4F, PLEKHH2, SEMA4C, WNT9B, PLA2G6, SEMA4B, NSD1, PARVA, RAP1GAP, ATG13, PPFIA1, HK1, KIT, FKBP1B, MPV17L2, FAT3, XBP1, BCL2, LRRTM2, BCL6, BCL9L, AGO4, LYPLAL1, ACSL4, TLX2, ELMOD1, TBC1D2, CAPN6, CNST, NOS1, SWAP70, AXL, LMNA, LRRC46, MID1, MARCH5, SNAI1, CDH13, RPS6KA4, PHF19, DKK1, OBFC1, MEX3C, NEUROD2, SVIP, ADGRL1, LRP4, SRCIN1, SLC6A1, TNFSF14, CXCL10, WNT1, ATG5, MYOCD, ANK3, GATA3, TBC1D13, LGI1, SAR1A, DISC1, PID1, RET, STMN3, DLL1, CDKL2, TBC1D22A, NUMBL, MAP4K4, SLITRK4, TRIM32, AKAP6, RELN, STMN1, CLIP3, MAP6D1, CAV3, FXYD2, SNX9, FZR1, DRD2, STRAP, ULK4, LMAN1, SRC, NR2C2, SLX4, TRIM67, OVOL2, GMIP, CNOT6L, AXIN2, TBC1D30, STK38L, BMF, CSF1R, DIXDC1, ZMYM4, MYO1C, PODXL, MET, FOXP3, TRIM62, CORO1C, TBC1D25, CDC42SE2, NEDD4, CXCL16, DNAJB2, DNAJB1, RHOJ, DPH6, RHOQ, PEX3, FES, SPICE1, MBP, WISP2, RSPO1, TRIM9, PAX7, CAMSAP1, RTF1, PLCB1, FOSL1, CCDC88A, ADIPOR2, CHST3, PRKCH, PADI2, LEF1, PRKCE, VAT1, PRKD1, PRKCQ, TPPP, CNTN2, TREM1, CUX1, EPN2, ABLIM3, PPM1A, CTCFL, CALCOCO2, CALCB, MOAP1, FAM107A, TOR1A, PALM2, NFATC4, NEDD4L, EHD4, SYNPO, PIK3R2, INF2, PTPRD, DNM1L, MAP1A, CREB1, ASIC2, TMBIM1, SIRT1, GMFB, PPP1R9B, RPL13A, KCNN3, KDM4C, EVI5L, IGFBP3 | 1.585599391 | 5.08E-11 | 1.69E-11 | 1.32E-11 |
| GO:0009966~regulation of signal transduction | 254 | 18.62170088 | 3.35E-14 | SNCA, LHCGR, LPAR2, RORA, ITSN1, SHKBP1, DDX17, CRY2, GAB1, SERPINE1, CREB3L1, DAB2IP, STRN3, PTPRR, RAD9A, UBR1, RIC8B, PPARGC1B, FARP2, HNF4A, SERPINF2, VEGFA, MNT, PDGFRA, PDGFRB, ATPIF1, RALGPS2, PPP2R3A, RALGPS1, GCNT2, GNAI2, HOXA13, DAG1, NFKBIA, UBA5, BDKRB2, GPR143, PEA15, TNKS, ASXL2, BMP3, GPR158, ARHGEF33, ABR, MAP2K1, IL1RN, SMAD4, MSTN, SKI, HGF, GAS1, EPHA4, NOTCH2, EI24, DLX1, NOTCH1, TRPS1, TMOD2, RGS6, GRK6, KLF4, BMP8B, OPRM1, CREBRF, LZTS2, HNF1A, PPARG, SHOC2, ZEB1, TKFC, TGFA, GPHB5, AGAP2, MYC, ALS2CL, ARHGEF3, PGAP2, FLOT2, TP53, CACNG4, ARHGEF9, NFAM1, IL6R, CACNG2, MYADM, LRPAP1, INHBB, ZDHHC17, CCND1, MAP3K15, KSR2, SEMA4C, PLA2G6, KSR1, MAP3K14, CLOCK, ZMYND11, RAP1GAP, CCR1, FKBP1A, KIT, CCL22, PLEKHG2, XBP1, BCL2, BCL6, BCL9L, RASA4, MLLT3, NOS1, AXL, LMNA, DGKI, MID1, TMPRSS6, SNAI1, TP73, CDH13, DUSP4, DKK1, NEUROD2, DGKZ, HTR2C, LRP4, TP53INP1, HTR2A, SLC44A2, TNFSF14, JAG1, CXCL11, CXCL10, NLRC5, WNT1, PTGIS, MYOCD, MDFIC, GATA3, RRAS, SPRED1, RNF34, DISC1, PID1, RET, STMN3, PSD3, SIX3, FGF23, DLL1, SOCS4, IRS1, MAP4K4, GPR55, RELT, TRIM32, AKAP6, RELN, NETO2, CAV3, SYVN1, DRD2, STRAP, SIPA1, ONECUT2, SOX4, ULK4, GREM2, SRC, IRAK4, AMER1, PEG10, PPP1R16B, ECE1, TRIM67, OVOL2, RNF165, AXIN2, BMF, FGD6, FGD3, CSF1R, DIXDC1, MYO1C, MET, ARPP19, NR4A2, TRIM62, FOXP1, CDKN1C, IKBKE, UACA, HDAC1, CDC42SE2, NEDD4, PDCD4, LGR4, SPRY3, PRMT2, RSPO1, HPSE, WWC3, LTF, PLCB1, CASP2, IRAK2, ANKS1A, PRKCH, PADI2, LEF1, EDAR, DAPK2, PRKCE, ARHGAP26, PRKCB, PRKD1, MFN2, PRKCQ, ACVR2B, NCOA1, UBE2K, CNTN2, PSME3, FAIM2, KMT2D, RTN4RL1, CTNND2, CYTH4, PPM1A, CTNND1, KITLG, CALCA, MOAP1, TSPAN33, RASGRP4, MARVELD3, NFATC4, INPP5D, THPO, PTPRD, DNM1L, TSPAN14, MAL, RCAN1, TMBIM1, SIRT1, WNT2B, RDH11, PPP1R9B, ATXN7, HEYL, SMPD1, IRF1, IRF4, IGFBP3, RNF41 | 1.567863109 | 2.56E-10 | 6.40E-11 | 6.66E-11 |
| GO:0051049~regulation of transport | 201 | 14.73607038 | 8.07E-13 | CTTNBP2NL, CALHM1, SYT1, CHMP3, VTCN1, ADCY5, LHCGR, SNCA, SYT9, SIDT2, CD47, CRY2, RAB29, SERPINE1, VPS4A, RAB21, DAB2IP, SCN2B, PCLO, CRHR1, HNF4A, VEGFA, PDGFRB, STC1, ATPIF1, SUPT6H, SCN1A, GNAI2, CACNB1, NFKBIA, CACNB3, GPR143, MYT1, RIMS4, RIMS3, PEA15, ACE, GOLPH3L, SLC31A2, HIP1, UNC119, ABR, MAP2K1, IL1RN, PTPN14, SMAD4, OXSR1, GAS1, NOTCH1, KCNJ8, GRK6, MARCKS, OPRM1, CREBRF, JPH3, GFAP, HNF1A, PPARG, SYNJ1, VPS37B, TMEM109, NMUR1, MAPT, EEF2K, MYC, KDELR3, STX1A, ANP32B, RAB4A, ZDHHC8, CACNG4, CFTR, CACNG2, INHBB, MYRIP, GRM7, CLIC5, PLA2G6, KCNH7, CLOCK, RAB3B, RAP1GAP, CCR1, ATG13, HK1, FKBP1A, FKBP1B, TPCN2, BEST1, XBP1, BCL2, LRRTM2, PER2, LYPLAL1, ACSL4, ELMOD1, NOS1, CNST, AXL, LRRC46, ATP1A2, CDH13, DKK1, PLN, SVIP, HTR2C, HTR2A, NCBP2, RNASEL, KCNC3, SRCIN1, SLC6A1, XPO4, TNFSF14, ILDR1, CXCL11, CXCL10, SLC16A1, ATG5, ANK2, ANK3, MDFIC, GATA3, PDE4B, MS4A2, LGI3, SLC25A27, SAR1A, PID1, FGF23, DLL1, PKIA, IRS1, CDKL2, MAP4K4, UCN2, AKAP6, RELN, VAMP2, CLIP3, GLP1R, NETO2, CAV3, FXYD2, DRD2, KCNA2, SOX4, SNX4, SRC, OAZ2, KCNS2, SLC30A3, AXIN2, HCN3, CSF1R, MYO1C, MET, FOXP3, CACNA2D2, FOXP1, UACA, PLCG1, HDAC1, NEDD4, CACNA1E, NAT8L, DPH6, RHOQ, FES, LGR4, TMEM173, KCNQ3, RSPO1, TRIM9, PLCB1, NOS1AP, ADIPOR2, PFKM, EDAR, PRKCE, PRKCB, PRKD1, PRKCQ, EPN2, ABLIM3, PPM1A, PPP3R1, CALCA, TOR1A, SYN2, NEDD4L, PIK3R2, EHD4, GNAO1, DNM1L, CREB1, ASIC2, SIRT6, SIRT1, KCNN3, SCN4A | 1.633683253 | 6.16E-09 | 1.23E-09 | 1.60E-09 |
| GO:0048468~cell development | 235 | 17.228739 | 1.10E-12 | SYT1, ADCY5, FGFRL1, SIDT2, HOOK3, S1PR2, S1PR3, BRINP1, RAB29, MED27, RARB, LRRC7, RAB21, DBNL, DAB2IP, PTPRM, ROCK1, EFNB3, EFNB1, ZHX2, MYH9, FARP2, VSIG1, FOXG1, VEGFA, PDGFRA, PDGFRB, TGIF2, STC1, GCNT2, HOXA13, TDRD6, CRTC1, TFCP2L1, DAG1, NRN1, EPHB2, CALU, ALCAM, ACE, PVRL1, PVRL2, PLAG1, BMP3, MAP2K1, SMAD4, MSTN, SKI, HGF, GAS1, NOTCH3, EPHA4, SEMA6A, DLX1, NOTCH1, TRPS1, TMOD2, RGS6, MARCKS, AREG, TCF12, KLF4, BMP8B, NRP2, OPRM1, ARSB, GFAP, CPEB3, PPARG, SYNJ1, ZEB1, XYLT1, MAPT, SEMA3E, ANP32A, EEF2K, MYC, TP53, CFTR, COL25A1, SPEN, MYADM, MYCN, INHBB, ANKRD23, SEMA4F, CLIC5, SEMA4C, WNT9B, SEMA4B, MAP3K14, CLOCK, PARVA, SRGAP2, CLDN18, RAP1GAP, TH, KIT, FKBP1B, FAT3, XBP1, FAT4, SPEG, BCL2, BCL11B, PER2, SLC4A7, BCL6, BCL9L, ACSL4, TLX2, NOS1, AXL, LMNA, AFF4, TNP1, SNAI1, RAPH1, TP73, PHF19, DKK1, MEX3C, NEUROD2, GFRA1, TMTC3, LRP4, SRCIN1, JAG1, VCL, GP5, ATG5, ANK2, MYOCD, UNC5A, ANK3, GATA3, LRRC55, LGI1, DISC1, FNDC3B, RBFOX2, RET, SATB2, STMN3, SIX3, DLL1, GRHL2, NUMBL, MAP4K4, SLITRK4, TRIM32, AKAP6, RELN, NGB, STMN1, CAV3, STRAP, DRD2, PEAK1, ONECUT2, SOX4, ULK4, LIN28A, SRC, PPP1R16B, TRIM67, OVOL2, RNF165, AXIN2, TMEM79, CSF1R, DIXDC1, MUC2, PODXL, MET, NTN4, NR4A2, CACNA2D2, TRIM62, FOXP1, CORO1C, CDKN1C, HDAC1, NEDD4, ADAM22, ARID4A, ARID4B, FES, UQCRQ, MBP, SPRY3, PRMT2, KCNQ3, RTF1, CAMSAP1, CNTNAP2, CNTNAP1, CASP2, CCDC88A, NOS1AP, CHST3, PRKCH, LEF1, CDK6, BBS1, PRKD1, NCOA1, XIRP1, CNTN2, MYRF, JMJD1C, CUX1, ABLIM1, KMT2D, CTNND2, PPP3R1, KITLG, NPTX1, TOR1A, NFATC4, NEDD4L, DCX, PTPRD, GNAO1, NCDN, AKIP1, CREB1, DPYSL4, SIRT6, RCAN1, SIRT1, PPP1R9B, MYPN, HEYL, KDM4C | 1.553581916 | 8.38E-09 | 1.40E-09 | 2.18E-09 |
| GO:0048699~generation of neurons | 173 | 12.68328446 | 2.36E-12 | SYT1, SRCIN1, ADCY5, RORA, JAG1, HOOK3, VCL, WNT1, GP5, BRINP1, OLIG3, UNC5A, ANK3, GATA3, RAB29, RARB, LRRC55, LGI1, DISC1, RAB21, LRRC7, DBNL, RBFOX2, SATB2, RET, DAB2IP, PTPRM, STMN3, EFNB3, EFNB1, PTPRR, ZHX2, SIX3, DLL1, FARP2, NUMBL, MAP4K4, SLITRK4, NAV1, TRIM32, FOXG1, VEGFA, TGIF2, RELN, NGB, STMN1, PPP2R3A, DRD2, CRTC1, ONECUT2, DAG1, SOX4, ULK4, LIN28A, NRN1, CALU, EPHB2, ALCAM, ACE, TRIM67, PVRL1, RNF165, CSF1R, PLAG1, DIXDC1, MAP2K1, MET, NR4A2, SMAD4, NTN4, SKI, HGF, GAS1, FOXP1, NOTCH3, CDKN1C, EPHA4, SEMA6A, DLX1, NOTCH1, HDAC1, NEDD4, RGS6, MARCKS, AREG, TCF12, KLF4, NRP2, ARSB, OPRM1, GFAP, CPEB3, PPARG, SYNJ1, ZEB1, FES, UQCRQ, MBP, SPRY3, XYLT1, PEX2, MAPT, PAX7, CAMSAP1, SEMA3E, ANP32A, EEF2K, CNTNAP2, CNTNAP1, MYC, CCDC88A, NOS1AP, SDK2, TP53, CHST3, PRKCH, LEF1, COL25A1, SPEN, MYCN, BBS1, PRKD1, PRKCQ, NCOA1, SEMA4F, CLIC5, CNTN2, WNT9B, SEMA4C, SEMA4B, CUX1, MAP3K14, FAIM2, SRGAP2, ABLIM1, RAP1GAP, TH, CTNND2, KIT, FKBP1B, NPTX1, FAT3, XBP1, FAT4, BCL2, BCL11B, TOR1A, PER2, SLC4A7, BCL6, NFATC4, NEDD4L, DCX, ACSL4, TLX2, PTPRD, NOS1, GNAO1, NCDN, CREB1, AXL, DPYSL4, RAPH1, SIRT1, TP73, WNT2B, PPP1R9B, DKK1, HEYL, NEUROD2, KDM4C, GFRA1, LRP4 | 1.693201148 | 1.80E-08 | 2.58E-09 | 4.70E-09 |
| GO:0022008~neurogenesis | 182 | 13.3431085 | 4.64E-12 | SYT1, SRCIN1, ADCY5, RORA, JAG1, HOOK3, VCL, WNT1, GP5, BRINP1, OLIG3, UNC5A, ANK3, RAB29, GATA3, RARB, LRRC55, LGI1, DISC1, RAB21, LRRC7, DBNL, RBFOX2, SATB2, RET, DAB2IP, PTPRM, STMN3, EFNB3, EFNB1, PTPRR, SIX3, ZHX2, SOCS7, DLL1, NUMBL, FARP2, MAP4K4, SLITRK4, NAV1, VEGFA, TRIM32, FOXG1, PDGFRA, TGIF2, RELN, NGB, STMN1, PPP2R3A, DRD2, CRTC1, ONECUT2, DAG1, SOX4, ULK4, LIN28A, NRN1, CALU, EPHB2, ALCAM, ACE, TRIM67, PVRL1, RNF165, CSF1R, PLAG1, DIXDC1, MAP2K1, MET, NR4A2, SMAD4, NTN4, ROGDI, SKI, HGF, GAS1, FOXP1, NOTCH3, CDKN1C, EPHA4, SEMA6A, DLX1, NOTCH1, HDAC1, NEDD4, RGS6, MARCKS, AREG, ADAM22, TCF12, KLF4, NRP2, ARSB, OPRM1, GFAP, CPEB3, PPARG, SYNJ1, NAP1L1, ZEB1, FES, UQCRQ, MBP, SPRY3, KCNQ3, XYLT1, PEX2, MAPT, PAX7, CAMSAP1, SEMA3E, ANP32A, EEF2K, CNTNAP2, CNTNAP1, MYC, CCDC88A, NOS1AP, SDK2, TP53, CHST3, PRKCH, LEF1, CDK6, COL25A1, SPEN, MYCN, BBS1, PRKD1, PRKCQ, NCOA1, SEMA4F, CLIC5, CNTN2, WNT9B, SEMA4C, MYRF, SEMA4B, CUX1, MAP3K14, FAIM2, SRGAP2, ABLIM1, RAP1GAP, TH, CTNND2, PPP3R1, KIT, FKBP1B, NPTX1, FAT3, XBP1, FAT4, BCL2, BCL11B, TOR1A, PER2, SLC4A7, BCL6, NFATC4, NEDD4L, DCX, ACSL4, TLX2, PTPRD, NOS1, GNAO1, NCDN, CREB1, AXL, DPYSL4, RAPH1, SIRT1, TP73, WNT2B, PPP1R9B, DKK1, HEYL, NEUROD2, KDM4C, GFRA1, LRP4 | 1.653176703 | 3.54E-08 | 4.43E-09 | 9.22E-09 |
| GO:0007399~nervous system development | 237 | 17.37536657 | 4.79E-12 | SYT1, LDHA, ADCY5, LHCGR, RORA, HOOK3, BRINP1, RAB29, RARB, LRRC7, RAB21, INA, DBNL, DAB2IP, PTPRM, SCN2B, EFNB3, EFNB1, PTPRR, ZHX2, BSN, PCLO, FARP2, CRHR1, NAV1, NAV2, FOXG1, VEGFA, PDGFRA, TGIF2, PPP2R3A, PFKFB3, CRTC1, HMGCS1, DAG1, NRN1, EPHB2, CALU, ALCAM, TAL2, ACE, PVRL1, PLAG1, ABR, MAP2K1, SMAD4, ROGDI, SKI, HGF, GAS1, KCNK3, NOTCH3, EPHA4, SEMA6A, DLX1, NOTCH1, RGS6, MARCKS, AREG, TCF12, KLF4, NCOR2, NRP2, OPRM1, ARSB, GFAP, CRNKL1, CPEB3, GLRA3, PPARG, SYNJ1, NAP1L1, ZEB1, XYLT1, MAPT, SEMA3E, ANP32A, EEF2K, MYC, ANP32B, SDK2, TP53, COL25A1, SPEN, MYCN, INHBB, SEMA4F, CLIC5, SEMA4C, WNT9B, SEMA4B, MAP3K14, SRGAP2, FUT9, RAP1GAP, CCR1, TH, KIT, FKBP1B, FAT3, XBP1, FAT4, BCL2, LRRTM2, BCL11B, PER2, SLC4A7, BCL6, ACSL4, TLX2, NOS1, AXL, RAPH1, TP73, DKK1, NEUROD2, GFRA1, ADGRL1, LRP4, SRCIN1, SLC6A3, JAG1, VCL, WNT1, GP5, ANK2, OLIG3, UNC5A, ANK3, GATA3, SMIM3, LRRC55, LGI1, DISC1, RBFOX2, RET, SATB2, STMN3, SIX3, SOCS7, DLL1, GRHL2, NUMBL, MAP4K4, SLITRK4, TRIM32, RELN, NGB, STMN1, DRD2, KCNA2, ONECUT2, SOX4, LRIG1, ULK4, LIN28A, NR2C2, SRC, TRIM67, OVOL2, SBK1, RNF165, CSF1R, DIXDC1, B4GALT2, MET, NTN4, NR4A2, AK3, AK4, SLC6A17, FOXP1, FOXP2, CDKN1C, HDAC1, NEDD4, ZIC5, ADAM22, SLC5A3, NDST1, BCAN, FES, UQCRQ, MBP, SPRY3, KCNQ3, PEX2, PAX7, CAMSAP1, DLG3, CNTNAP2, CNTNAP1, PLCB1, CASP2, CABLES1, CCDC88A, NRXN2, NOS1AP, CHST3, PRKCH, PADI2, LEF1, CDK6, BBS1, PRKD1, PRKCQ, NCOA1, CNTN2, MYRF, CNTN3, CUX1, FAIM2, ABLIM1, CTNND2, PPP3R1, CTNND1, NPTX1, SEC16A, TOR1A, NFATC4, PAFAH1B2, NEDD4L, DCX, PTPRD, GNAO1, NCDN, TRNP1, CREB1, ASIC2, DPYSL4, MAL, SIRT1, WNT2B, PPP1R9B, HEYL, KDM4C, POFUT1 | 1.528538254 | 3.65E-08 | 4.06E-09 | 9.51E-09 |
| GO:0045595~regulation of cell differentiation | 190 | 13.92961877 | 5.10E-12 | SYT1, SRCIN1, PDLIM7, ADCY5, TNFSF14, JAG1, RORA, PNP, HOOK3, CXCL10, S1PR2, WNT1, S1PR3, DDX17, BRINP1, MYOCD, RAB29, GATA3, MED27, RARB, DISC1, FNDC3B, TWIST2, LRRC7, RAB21, RBFOX2, RET, DAB2IP, ROCK1, SIX3, ZHX2, FGF23, DLL1, GRHL2, PPARGC1B, NUMBL, MAP4K4, GPR55, SERPINF2, VEGFA, TRIM32, FOXG1, PDGFRA, AKAP6, TGIF2, RELN, NEK5, ASB4, GLP1R, SUCO, SUPT6H, CAV3, GCNT2, DRD2, STRAP, CRTC1, DAG1, SOX4, NFKBIA, ULK4, LIN28A, NR2C2, EPHB2, ACE, TRIM67, OVOL2, AXIN2, CSF1R, PLAG1, ASXL2, DIXDC1, ESRRA, ZBTB46, MAP2K1, MET, SMAD4, MSTN, SKI, HGF, FOXP3, TRIM62, FOXP1, CORO1C, NOTCH3, NOTCH2, EPHA4, SEMA6A, DLX1, NOTCH1, HDAC1, NEDD4, TRPS1, RGS6, MARCKS, AREG, TCF12, KLF4, NCOR2, OPRM1, ARSB, GFAP, CPEB3, MITF, PPARG, SYNJ1, ZEB1, FES, MBP, XYLT1, MAPT, PAX7, RTF1, SEMA3E, ANP32A, EEF2K, LTF, MKX, PLCB1, MYC, SH3PXD2B, CCDC88A, ANP32B, FLOT2, TP53, PRKCH, LEF1, CDK6, SPEN, NFAM1, MYADM, MYCN, PRKD1, ACVR2B, NCOA1, CCND1, ANKRD23, SEMA4F, CNTN2, WNT9B, SEMA4C, SEMA4B, JMJD1C, CUX1, CLOCK, SRGAP2, CLDN18, RAP1GAP, CCR1, KITLG, KIT, FKBP1B, CALCA, FNDC5, FAT3, XBP1, FAT4, BCL2, BCL11B, PER2, BCL6, NFATC4, BCL9L, INPP5D, NEDD4L, TLX2, THPO, PTPRD, NOS1, CREB1, AXL, LMNA, RCAN1, SNAI1, COL5A2, SIRT1, TP73, PHF19, DKK1, PRLR, HEYL, IRF1, NEUROD2, MEX3C, KDM4C, IGFBP3, HTR2C, LRP4, TOB2, RNF41, HTR2A | 1.629058 | 3.89E-08 | 3.89E-09 | 1.01E-08 |
| GO:0010647~positive regulation of cell communication | 179 | 13.12316716 | 1.23E-11 | SYT1, SLC44A2, SLC6A1, SNCA, LHCGR, TNFSF14, LPAR2, JAG1, ILDR1, ITSN1, CXCL11, CXCL10, SHKBP1, NLRC5, WNT1, DDX17, PTGIS, ATG5, MYOCD, ANK3, MDFIC, GATA3, GAB1, SPRED1, LGI1, DISC1, RET, DAB2IP, FGF23, DLL1, RAD9A, IRS1, CRHR1, GPR55, RELT, HNF4A, SERPINF2, VEGFA, TRIM32, PDGFRA, PDGFRB, AKAP6, ATPIF1, RELN, VAMP2, GLP1R, NETO2, PPP2R3A, GCNT2, GNAI2, DRD2, CRTC1, SOX4, SNX4, SRC, NR2C2, RIMS3, IRAK4, PEA15, AMER1, ECE1, GMIP, RNF165, TNKS, BMF, CSF1R, ASXL2, DIXDC1, BMP3, MYO1C, MAP2K1, IL1RN, MET, ARPP19, SMAD4, MSTN, HGF, GAS1, TRIM62, KCNK3, FAM131B, CDKN1C, NOTCH2, EPHA4, IKBKE, EI24, NOTCH1, HDAC1, NEDD4, TRPS1, TMOD2, NAT8L, BMP8B, CREBRF, OPRM1, GFAP, CRNKL1, SHOC2, PEX3, LGR4, RSPO1, HPSE, LTF, TGFA, PLCB1, AGAP2, MYC, CASP2, IRAK2, STX1A, TP53, CHST3, PRKCH, CFTR, IL6R, PFKM, EDAR, NFAM1, PRKCE, ZCCHC17, PRKCB, PRKD1, INHBB, PRKCQ, ACVR2B, ZDHHC17, MYRIP, KSR2, MAP3K15, UBE2K, CNTN2, SEMA4C, PLA2G6, TREM1, MAP3K14, KSR1, KMT2D, RAB3B, CCR1, ATG13, PPM1A, HK1, KITLG, CALCOCO2, FKBP1A, KIT, NETO1, CALCB, MOAP1, CCL22, TSPAN33, XBP1, RASGRP4, LRRTM2, TOR1A, NFATC4, PAFAH1B2, MLLT3, THPO, NOS1, DNM1L, MAP1A, CREB1, AXL, TSPAN14, MAL, DGKI, MID1, SIRT1, TP73, WNT2B, CDH13, DKK1, NEUROD2, MEX3C, IGFBP3, HTR2C, TP53INP1, HTR2A | 1.643398567 | 9.37E-08 | 8.52E-09 | 2.44E-08 |
| GO:0030182~neuron differentiation | 156 | 11.43695015 | 3.83E-11 | SYT1, SRCIN1, ADCY5, RORA, JAG1, VCL, WNT1, GP5, BRINP1, OLIG3, UNC5A, ANK3, GATA3, RAB29, RARB, LRRC55, LGI1, DISC1, RAB21, LRRC7, DBNL, RBFOX2, SATB2, RET, DAB2IP, PTPRM, STMN3, EFNB3, EFNB1, PTPRR, ZHX2, SIX3, DLL1, FARP2, NUMBL, MAP4K4, SLITRK4, TRIM32, FOXG1, VEGFA, TGIF2, RELN, NGB, STMN1, PPP2R3A, DRD2, CRTC1, ONECUT2, DAG1, SOX4, ULK4, LIN28A, NRN1, CALU, EPHB2, ALCAM, TRIM67, PVRL1, RNF165, CSF1R, DIXDC1, MAP2K1, MET, NR4A2, NTN4, SMAD4, HGF, GAS1, FOXP1, NOTCH3, CDKN1C, EPHA4, SEMA6A, DLX1, NOTCH1, HDAC1, NEDD4, RGS6, MARCKS, AREG, TCF12, KLF4, NRP2, ARSB, GFAP, CPEB3, ZEB1, FES, UQCRQ, MBP, SPRY3, XYLT1, MAPT, PAX7, SEMA3E, CAMSAP1, ANP32A, EEF2K, CNTNAP2, CNTNAP1, CCDC88A, NOS1AP, SDK2, CHST3, LEF1, COL25A1, MYCN, BBS1, PRKD1, PRKCQ, NCOA1, SEMA4F, CLIC5, CNTN2, WNT9B, SEMA4C, SEMA4B, CUX1, MAP3K14, FAIM2, SRGAP2, ABLIM1, RAP1GAP, TH, CTNND2, FKBP1B, NPTX1, FAT3, XBP1, FAT4, BCL2, BCL11B, TOR1A, SLC4A7, BCL6, NFATC4, NEDD4L, DCX, ACSL4, TLX2, PTPRD, GNAO1, NCDN, CREB1, DPYSL4, SIRT1, RAPH1, TP73, WNT2B, PPP1R9B, DKK1, HEYL, NEUROD2, KDM4C, GFRA1, LRP4 | 1.692155904 | 2.92E-07 | 2.43E-08 | 7.61E-08 |
| GO:0031325~positive regulation of cellular metabolic process | 285 | 20.89442815 | 3.95E-11 | PPP2R5A, SNCA, LHCGR, LPAR2, RORA, ITSN1, S1PR2, DDX17, GAB1, CREB3L1, RARB, DAB2IP, STRN3, YY1, HNF4G, MYH9, PPARGC1B, CRHR1, MTF1, HNF4A, SERPINF2, MCIDAS, VEGFA, MC2R, PDGFRA, PDGFRB, ATPIF1, NEK5, SUPT6H, GCNT2, GNAI2, HOXA13, CRTC1, NFKBIA, MYT1, RIMS3, PLAGL1, ACE, TNKS, HIP1, PLAG1, ASXL2, KLF6, BMP3, UNC119, ESRRA, MAP2K1, IL1RN, SMAD4, MSTN, SKI, OXSR1, HGF, USF1, FAM131B, KCNK3, NOTCH3, EPHA4, NOTCH1, NR1I2, CSRNP3, TRPS1, RBMXL1, MARCKS, AREG, TCF12, FOXI1, KLF4, NCOR2, BMP8B, OPRM1, HNF1A, CRNKL1, CPEB3, PPARG, MITF, ZEB1, GUCY1A3, TGFA, MKX, MYC, AGAP2, ANP32B, TP53, ERLIN1, SPEN, NFAM1, IL6R, FOSB, ZCCHC17, MYCN, INHBB, CCND1, MAP3K15, KSR2, SEMA4C, PLA2G6, KSR1, NSD1, CLOCK, CCR1, UNG, ATG13, HK1, FKBP1A, KIT, CCL22, ACSL1, MPV17L2, XBP1, BCL2, BCL11B, BCL6, BCL9L, NOS1, LMNA, MID1, ATMIN, TMPRSS6, SNAI1, BRAT1, TP73, SGTA, TRIM21, PREB, CDH13, RPS6KA4, PHF19, PKNOX1, OBFC1, MEX3C, KDM8, NEUROD2, SVIP, HTR2C, TP53INP2, LRP4, HTR2A, TP53INP1, NCBP2, RNASEL, SLC6A1, SRCIN1, JAG1, CXCL11, CXCL10, NLRC5, WNT1, MYOCD, ATG5, MDFIC, GATA3, DISC1, SAMD4A, PID1, RET, SATB2, MTA2, SIX3, FGF23, DLL1, SOCS4, GRHL2, IRS1, RELT, GPR55, TRIM32, CELF3, AKAP6, NFE2L1, RELN, CLIP3, GLP1R, CAMTA1, SNX9, FZR1, DRD2, ONECUT2, SOX4, LIN28A, SRC, NR2C2, SLX4, AMER1, PPP1R16B, ECE1, TRIM67, GMIP, OVOL2, AXIN2, CSF1R, DIXDC1, MET, ARPP19, NR4A2, WHSC1, FOXP3, TRIM62, FOXP1, CDKN1C, HSPBP1, UACA, AKTIP, HDAC1, GTF2F1, ZIC5, DNAJB2, E2F3, ARID4A, ARID4B, RHOQ, PEX3, CBFA2T3, LGR4, TMEM173, MAZ, PRMT2, RSPO1, PAX7, RTF1, LTF, LRRFIP1, PLCB1, FOSL1, IRAK2, TBL1XR1, KDM7A, CCDC88A, FOXJ2, NOS1AP, CYCS, CHST3, PADI2, PRKCH, LEF1, DDN, PRKCE, PRKCB, PRKD1, PRKCQ, ACVR2B, NCOA1, DGAT1, PSME1, UBE2K, ATG4B, CNTN2, MYRF, PSME3, TREM1, CUX1, CALCR, ABLIM1, KMT2D, ABLIM3, PPP3R1, PPM1A, CTNND1, CTCFL, CALCOCO2, KITLG, CALCA, CALCB, RGMB, POU2F1, CHD1, NFATC4, PAFAH1B2, TNRC6B, EHD4, PIK3R2, THPO, CREB1, MAP1A, CREB5, SIRT1, ATXN7L3, PRLR, ATXN7, HEYL, SMPD1, IRF1, IRF4, IGFBP3, RNF41 | 1.428869996 | 3.02E-07 | 2.32E-08 | 7.86E-08 |
| GO:0007166~cell surface receptor signaling pathway | 222 | 16.27565982 | 4.62E-11 | ADCY5, SNCA, FGFRL1, AMOTL2, SHKBP1, CD47, S1PR3, GAB1, SERPINE1, GRID1, DAB2IP, EFNB3, EFNB1, PTPRR, FARP2, CRHR1, HNF4A, VEGFA, PDGFRA, PDGFRB, TGIF2, GCNT2, PPP2R3A, GNAI2, HOXA13, NFKBIA, ITGA10, CACNB3, EPHB2, PEA15, TNKS, KLF6, BMP3, CAMLG, IL1RN, SMAD4, MSTN, SKI, HGF, GAS1, NOTCH3, EPHA4, SEMA6A, NOTCH2, DLX1, NOTCH1, TRPS1, GRK6, AREG, KLF4, BMP8B, BTBD11, NRP2, OPRM1, CREBRF, LZTS2, HNF1A, PPARG, MITF, ZEB1, SEMA3E, TGFA, AGAP2, MYC, ADAM11, TP53, CACNG4, NFAM1, IL6R, CACNG2, ACKR4, INHBB, CCND1, SEMA4F, GRM7, WNT9B, SEMA4C, SEMA4B, ZMYND11, CLDN18, FUT8, CCR1, FKBP1A, KIT, CCL22, ACSL1, XBP1, FAT4, IL10RB, BCL2, BCL6, BCL9L, MLLT3, AXL, LMNA, TMPRSS6, SNAI1, CDH13, RPS6KA4, DKK1, P2RY14, PLN, ADGRL1, LRP4, JAG1, CXCL11, CXCL10, SLC2A8, NLRC5, WNT1, MYOCD, UNC5A, GATA3, PDE4B, MS4A2, RNF34, DISC1, PAG1, PID1, RET, SOGA1, CSNK1G1, SIX3, FGF23, SOCS7, DLL1, SOCS4, IRS1, NUMBL, RELT, TRIM32, RELN, GLP1R, NETO2, CAV3, STRAP, DRD2, ONECUT2, SOX4, EPHA10, GREM2, SRC, IRAK4, AMER1, PEG10, OVOL2, RNF165, ITGB8, AXIN2, CSF1R, DIXDC1, MYO1C, MET, FOXP3, CDKN1C, PLCG1, HDAC1, NEDD4, ADGRF2, NDST1, SGPP1, IL21R, RHOQ, CXCR1, FES, LGR4, JADE2, FAM83A, RSPO1, CDIP1, LTF, PLCB1, CASP2, IRAK2, TBL1XR1, ADIPOR2, PRKCH, PADI2, LEF1, CDK6, PRKCE, NRIP2, PRKCB, PRKD1, PRKCQ, ACVR2B, XIRP1, UBE2K, CNTN2, PSME3, EPN2, CALCR, FRK, RTN4RL1, CTNND2, PPM1A, KITLG, CTNND1, RGMB, MOAP1, TSPAN33, NFATC4, GIGYF1, THPO, PIK3R2, IL2RB, CR2, NCEH1, CREB1, TSPAN14, MAL, TMBIM1, TSPAN18, SIRT1, WNT2B, PRLR, ATXN7, HEYL, IRF1, IGFBP3, POFUT1, RNF41 | 1.521662612 | 3.53E-07 | 2.52E-08 | 9.19E-08 |
| GO:0010604~positive regulation of macromolecule metabolic process | 274 | 20.08797654 | 5.07E-11 | PPP2R5A, SNCA, LPAR2, RORA, S1PR2, DDX17, GAB1, SERPINE1, CREB3L1, RARB, DAB2IP, STRN3, YY1, HNF4G, MYH9, PPARGC1B, MTF1, HNF4A, SERPINF2, MCIDAS, VEGFA, PDGFRA, PDGFRB, ATPIF1, NEK5, SUPT6H, SUCO, PPP2R3A, GCNT2, GNAI2, HOXA13, CRTC1, NFKBIA, MYT1, PLAGL1, ACE, LPCAT1, TNKS, HIP1, PLAG1, ASXL2, KLF6, BMP3, UNC119, ESRRA, MAP2K1, IL1RN, SMAD4, MSTN, SKI, OXSR1, HGF, USF1, NOTCH3, EPHA4, NOTCH1, NR1I2, CSRNP3, TRPS1, RBMXL1, MARCKS, AREG, TCF12, FOXI1, KLF4, NCOR2, BMP8B, OPRM1, CREBRF, HNF1A, CPEB3, PPARG, MITF, ZEB1, TGFA, MKX, MYC, AGAP2, ANP32B, TP53, ERLIN1, SPEN, NFAM1, IL6R, FOSB, MYCN, INHBB, CCND1, MAP3K15, KSR2, SLC26A9, SEMA4C, PLA2G6, KSR1, NSD1, CLOCK, CCR1, UNG, ATG13, FKBP1A, KIT, CCL22, ACSL1, MPV17L2, XBP1, BCL2, BCL11B, BCL6, BCL9L, NOS1, LMNA, MID1, ATMIN, TMPRSS6, SNAI1, BRAT1, SGTA, TRIM21, TP73, PREB, CDH13, RPS6KA4, PHF19, PKNOX1, OBFC1, KDM8, NEUROD2, SVIP, HTR2C, TP53INP2, LRP4, TP53INP1, HTR2A, NCBP2, RNASEL, SRCIN1, JAG1, NLRC5, WNT1, MYOCD, ANK2, MDFIC, ANK3, GATA3, DISC1, SAMD4A, PID1, RET, SATB2, MTA2, SIX3, FGF23, DLL1, SOCS4, GRHL2, IRS1, RELT, GPR55, TRIM32, CELF3, AKAP6, NFE2L1, RELN, CLIP3, GLP1R, SNX9, CAMTA1, FZR1, DRD2, ONECUT2, SOX4, LIN28A, SRC, NR2C2, OAZ2, SLX4, AMER1, PPP1R16B, ECE1, TRIM67, OVOL2, ITGB8, AXIN2, CSF1R, DIXDC1, MET, NR4A2, WHSC1, FOXP3, TRIM62, FOXP1, CDKN1C, HSPBP1, UACA, AKTIP, HDAC1, NEDD4, GTF2F1, ZIC5, DNAJB2, E2F3, ARID4A, PRKAG1, ARID4B, RHOQ, CBFA2T3, LGR4, TMEM173, MAZ, PRMT2, RSPO1, PAX7, RTF1, LTF, LRRFIP1, PLCB1, FOSL1, IRAK2, TBL1XR1, KDM7A, CCDC88A, FOXJ2, NOS1AP, CYCS, PADI2, PRKCH, LEF1, CDK6, EDAR, DDN, PRKCE, PRKCB, PRKD1, PRKCQ, ACVR2B, NCOA1, PSME1, ATG4B, UBE2K, CNTN2, MYRF, PSME3, CUX1, ABLIM1, KMT2D, ABLIM3, PPP3R1, PPM1A, CTNND1, CTCFL, KITLG, CALCA, RGMB, POU2F1, CHD1, NFATC4, NEDD4L, TNRC6B, EHD4, PIK3R2, THPO, CREB1, CREB5, SIRT1, ATXN7L3, PRLR, ATXN7, HEYL, SMPD1, IRF1, KDM4C, IRF4, IGFBP3, RNF41 | 1.439822236 | 3.87E-07 | 2.58E-08 | 1.01E-07 |
| GO:0065009~regulation of molecular function | 256 | 18.76832845 | 7.01E-11 | CTTNBP2NL, DYNC1LI1, PPP2R5A, SNCA, LHCGR, LPAR2, ITSN1, CRY2, GAB1, SERPINE1, TWIST2, RECK, DAB2IP, SCN2B, ROCK1, RAP1GDS1, RIC8B, PPARGC1B, FARP2, CRHR1, HNF4A, SERPINF2, VEGFA, PDGFRA, PDGFRB, ATPIF1, WFDC5, NEK5, ADD2, RALGPS2, RALGPS1, GNAI2, CRTC1, CACNB1, NFKBIA, CACNB3, CALU, ACE, SERINC1, TNKS, HIP1, UNC119, ARHGEF33, ABR, MAP2K1, IL1RN, SMAD4, MSTN, SKI, OXSR1, HGF, EPHA4, NOTCH1, CSRNP3, DCP1A, PKP4, RGS6, GRK6, MARCKS, KLF4, OPRM1, JPH3, CPEB2, PPARG, ZYG11B, CCNE2, NMUR1, TGFA, GPIHBP1, MYC, AGAP2, ALS2CL, ARHGEF3, ANP32B, RAB4A, TP53, CACNG4, CFTR, ARHGEF9, NFAM1, IL6R, CACNG2, LRPAP1, MTMR12, CCND1, MAP3K15, DNAJC24, SERPINB8, GRM7, SERPINB2, WNT9B, PLA2G6, NSD1, ZER1, CLOCK, SRGAP2, RAP1GAP, ATG13, HK1, BCCIP, FKBP1A, KIT, FKBP1B, GCH1, CCL22, PLEKHG2, ACSL1, BCL2, CHM, PER2, MLLT1, BCL6, MTMR9, RASA4, ELMOD1, TBC1D2, CNST, NOS1, PPP1R11, PPP1R10, ATP1A2, DGKI, TRIM21, TP73, DUSP4, DKK1, PPP1R8, OBFC1, PLN, NEUROD2, DGKZ, HTR2A, SRCIN1, TNFSF14, RASGEF1C, NLRC5, WNT1, PTGIS, ANK2, MYOCD, MDFIC, ANK3, GATA3, PDE4B, MS4A2, SPRED1, NQO1, RNF34, RET, STMN3, PSD3, FGF23, SOCS4, GRHL2, PKIA, IRS1, SGSM2, GPR55, TRIM32, AKAP6, RELN, NGB, VAMP2, NETO2, CAV3, PACS1, FXYD2, SNX9, FZR1, DRD2, SIPA1, GREM2, SRC, OAZ2, SLX4, PPP1R16B, KCNS2, GMIP, LMTK3, AXIN2, TBC1D30, FGD6, FGD3, CSF1R, ARHGDIB, MET, DOCK9, ARPP19, NR4A2, DOCK8, FOXP3, TRIM62, DOCK3, CORO1C, CDKN1C, HSPBP1, TBC1D25, UACA, AKTIP, PLCG1, NEDD4, GTF2F1, DNAJB1, PRKAG1, PIP5K1A, PDCD4, TMEM173, PRMT2, PAX7, ARHGAP1, LTF, DLG3, LRRFIP1, PLCB1, FOSL1, IRAK2, CCDC88A, NOS1AP, CYCS, RIMBP2, PRKCH, LEF1, PRKCE, ARHGAP26, PRKCB, PRKD1, PRKCQ, ACVR2B, PSME1, PSME3, CALCR, RTN4RL1, APH1A, CYTH4, KITLG, CALCA, RASGRP4, ARHGAP44, NFATC4, NEDD4L, PIK3R2, GNAO1, FETUB, ASIC2, RCAN1, TMBIM1, SIRT1, RALGDS, PPP1R9B, PRLR, HEYL, SMPD1, IRF4, EVI5L, IGFBP3, RNF41 | 1.459847103 | 5.35E-07 | 3.34E-08 | 1.39E-07 |
| GO:0008104~protein localization | 222 | 16.27565982 | 7.71E-11 | CHMP3, VTCN1, PPP2R5A, PLEKHM1, ADCY5, CHMP7, LHCGR, VPS52, SYT9, SIDT2, HOOK3, KIF13A, CRY2, RAB29, AAGAB, VPS4A, RAB21, DAB2IP, GBP5, ROCK1, MYH9, PCLO, VTI1A, HNF4A, VEGFA, SURF4, UBL4A, ATPIF1, CACNB1, DAG1, NFKBIA, MYT1, STX17, GOLPH3L, TNKS, FCHO2, UNC119, GPR158, IL1RN, PTPN14, SMAD4, GAS8, NOTCH2, NOTCH1, DCP1A, MARCKS, OPRM1, CREBRF, LZTS2, HNF1A, AP1B1, GLRA3, PPARG, AP3S2, AP1S2, NMUR1, NEURL1B, AGAP2, GPIHBP1, ALS2CL, JAKMIP1, SH3PXD2B, KDELR3, PGAP2, STX1A, ANP32B, FLOT2, RAB4A, ZDHHC8, TP53, CFTR, ARHGEF9, MYADM, INHBB, ZDHHC15, ZDHHC17, MYRIP, CLIC5, PLA2G6, FKBP11, CLOCK, RAB3B, CLDN18, PPFIA1, ATG13, BET1, HK1, FKBP1B, NETO1, ZDHHC23, RAB43, XBP1, NUP210, PER2, CHM, BCL6, LYPLAL1, ELMOD1, NOS1, CNST, LMNA, LRRC46, MID1, MARCH5, PREB, COG3, COG6, RAB36, MCFD2, SVIP, TP53INP2, LRP4, HTR2A, SRCIN1, XPO5, LMAN2L, XPO4, TNFSF14, ILDR2, PNP, VCL, SLC16A1, ATG5, ANK2, MDFIC, ANK3, GATA3, MS4A2, SAR1A, DISC1, PID1, ATG9A, SIX3, PKIA, IRS1, CDKL2, MAP4K4, LYST, AKAP6, RELN, VAMP2, CLIP3, ERC1, GLP1R, CAV3, PACS1, SNX9, SYVN1, DRD2, PPIL2, COPZ1, SOX4, SNX4, LMAN1, SRC, OAZ2, AXIN2, SEC61A1, CSF1R, MYO1C, CRB3, WHSC1, WIPI2, FOXP3, FOXP1, CORO1C, UACA, PLCG1, HDAC1, AKTIP, NEDD4, CACNA1E, SPCS2, DPH6, RHOQ, PEX3, PIP5K1A, RHOV, LGR4, TMEM173, PEX2, DLG3, CNTNAP2, CNTNAP1, NRXN2, KIF17, SRPRA, PFKM, EDAR, PRKCE, BBS1, MFN2, PRKCQ, ACVR2B, DGAT1, ATG4B, CNTN2, TOM1, TREM1, CALCR, SNX15, ABLIM3, PPM1A, PPP3R1, MOAP1, TSPAN33, TMEM33, MARVELD3, TOR1A, NEDD4L, PIK3R2, SPRN, DNM1L, ASIC2, TSPAN14, MAL, TMBIM1, HID1, SIRT1, PPP1R9B, CDAN1, KCNN3 | 1.513093888 | 5.89E-07 | 3.46E-08 | 1.53E-07 |
| GO:0045184~establishment of protein localization | 184 | 13.48973607 | 1.09E-10 | CHMP3, SRCIN1, PLEKHM1, VTCN1, PPP2R5A, XPO5, LMAN2L, XPO4, ADCY5, CHMP7, LHCGR, VPS52, TNFSF14, SYT9, ILDR2, SIDT2, PNP, KIF13A, SLC16A1, CRY2, ANK2, ATG5, ANK3, MDFIC, RAB29, GATA3, AAGAB, MS4A2, VPS4A, SAR1A, RAB21, PID1, DAB2IP, ATG9A, GBP5, ROCK1, SIX3, MYH9, CDKL2, PCLO, IRS1, PKIA, VTI1A, MAP4K4, HNF4A, LYST, UBL4A, SURF4, AKAP6, ATPIF1, VAMP2, CLIP3, ERC1, GLP1R, CAV3, PACS1, SNX9, SYVN1, DRD2, COPZ1, CACNB1, DAG1, SOX4, NFKBIA, SNX4, MYT1, LMAN1, SRC, OAZ2, STX17, GOLPH3L, AXIN2, SEC61A1, CSF1R, UNC119, MYO1C, IL1RN, PTPN14, SMAD4, FOXP3, FOXP1, CORO1C, NOTCH2, NOTCH1, UACA, AKTIP, PLCG1, HDAC1, NEDD4, MARCKS, CACNA1E, SPCS2, OPRM1, CREBRF, HNF1A, AP1B1, DPH6, PPARG, AP3S2, RHOQ, PEX3, PIP5K1A, RHOV, LGR4, TMEM173, AP1S2, NMUR1, PEX2, NEURL1B, AGAP2, GPIHBP1, JAKMIP1, KDELR3, PGAP2, STX1A, ANP32B, FLOT2, ZDHHC8, RAB4A, TP53, CFTR, SRPRA, PFKM, EDAR, PRKCE, MYADM, BBS1, ZDHHC15, MFN2, INHBB, PRKCQ, ACVR2B, ZDHHC17, MYRIP, DGAT1, ATG4B, CNTN2, TOM1, PLA2G6, TREM1, FKBP11, CLOCK, CALCR, RAB3B, SNX15, ABLIM3, ATG13, PPFIA1, PPM1A, PPP3R1, BET1, HK1, FKBP1B, MOAP1, RAB43, TSPAN33, XBP1, NUP210, TOR1A, CHM, PER2, BCL6, LYPLAL1, ELMOD1, PIK3R2, SPRN, NOS1, CNST, DNM1L, LMNA, LRRC46, TSPAN14, MAL, TMBIM1, HID1, SIRT1, PREB, COG3, PPP1R9B, COG6, RAB36, KCNN3, MCFD2, SVIP | 1.587925267 | 8.35E-07 | 4.64E-08 | 2.18E-07 |
| GO:2000026~regulation of multicellular organismal development | 191 | 14.00293255 | 2.28E-10 | SYT1, SRCIN1, PGF, ADCY5, JAG1, PNP, HOOK3, CXCL10, S1PR2, WNT1, S1PR3, DDX17, BRINP1, PTGIS, MYOCD, RAB29, GATA3, SERPINE1, WDR77, RRAS, RARB, DISC1, TWIST2, LRRC7, RAB21, RBFOX2, RET, DAB2IP, PTPRM, ROCK1, SIX3, ZHX2, FGF23, DLL1, GRHL2, PPARGC1B, NUMBL, MAP4K4, SLITRK4, GPR55, HNF4A, VEGFA, TRIM32, FOXG1, PDGFRB, AKAP6, TGIF2, RELN, ATXN1L, ASB4, SUPT6H, CAV3, PPP2R3A, GCNT2, DRD2, STRAP, CRTC1, DAG1, NFKBIA, ULK4, LIN28A, NR2C2, EPHB2, PPP1R16B, ACE, TRIM67, PVRL1, OVOL2, AXIN2, TMEM79, ARHGDIB, CSF1R, PLAG1, ASXL2, DIXDC1, ESRRA, ZBTB46, MAP2K1, IL1RN, MET, SMAD4, NTN4, MSTN, SKI, WHSC1, HGF, FOXP3, TRIM62, FOXP1, FOXP2, NOTCH3, NOTCH2, EPHA4, SEMA6A, DLX1, NOTCH1, PLCG1, HDAC1, NEDD4, TRPS1, RGS6, MARCKS, TCF12, KLF4, OPRM1, ARSB, GFAP, CPEB3, MITF, PPARG, SYNJ1, ZEB1, FES, LGR4, MBP, XYLT1, HPSE, MAPT, SEMA3E, ANP32A, EEF2K, PLCB1, AGAP2, MYC, CCDC88A, TP53, PRKCH, LEF1, CDK6, SPEN, NFAM1, MYADM, MYCN, PRKCB, PRKD1, ACVR2B, NCOA1, CCND1, SEMA4F, CNTN2, WNT9B, SEMA4C, MYRF, SEMA4B, CUX1, CLOCK, SRGAP2, CLDN18, RAP1GAP, CCR1, UNG, KITLG, KIT, FKBP1B, CALCA, FAT3, XBP1, FAT4, BCL2, LRRTM2, BCL11B, PER2, BCL6, NFATC4, BCL9L, INPP5D, NEDD4L, TLX2, MLLT3, THPO, PTPRD, NOS1, CREB1, ASIC2, AXL, LMNA, SNAI1, COL5A2, SIRT1, TP73, WNT2B, DKK1, PRLR, HEYL, IRF1, NEUROD2, KDM4C, ADGRL1, LRP4, TOB2, RNF41 | 1.556761274 | 1.74E-06 | 9.17E-08 | 4.54E-07 |
| GO:0040011~locomotion | 165 | 12.09677419 | 2.39E-10 | PVR, SRCIN1, TNFSF14, CXCL11, AMOTL1, VCL, CXCL10, CD47, OLIG3, UNC5A, ANK3, GATA3, PDE4B, SERPINE1, GAB1, RRAS, LGI1, MTUS1, DISC1, FNDC3B, RECK, RBFOX2, SATB2, RET, DAB2IP, PTPRM, ROCK1, EFNB3, TMEM201, MTA2, EFNB1, PTPRR, SIX3, SOCS7, MYH9, IRS1, NAV1, LYST, TRIM32, FOXG1, VEGFA, PDGFRA, CELF3, PDGFRB, RELN, STC1, GCNT2, PPP2R3A, STRAP, DRD2, PEAK1, ONECUT2, DAG1, ULK4, SRC, EPHB2, ALCAM, IRAK4, ACE, OVOL2, PVRL1, RNF165, PVRL2, ARHGDIB, CSF1R, DIXDC1, MUC2, ABR, MYO1C, MAP2K1, PODXL, IL1RN, MET, NR4A2, SMAD4, HACE1, GAS1, HGF, DOCK8, GAS8, TRIM62, FOXP1, CORO1C, EPHA4, SEMA6A, NOTCH1, PLCG1, NEDD4, CXCL16, CACNA1E, KLF4, NRP2, ARSB, JPH3, MITF, CXCR1, PIP5K1A, FES, PEX2, MAPT, SEMA3E, CAP1, PLCB1, MYC, FMNL3, CCDC88A, TP53, LEF1, PADI2, ACKR4, DAPK2, PRKCE, MYADM, ELMO1, BBS1, PRKD1, PRKCQ, SEMA4F, CNTN2, SEMA4C, PLA2G6, SEMA4B, TREM1, USP24, SRGAP2, PARVA, ABLIM1, FUT8, CCR1, KITLG, KIT, TRIM10, CALCA, CCL22, FAT3, BCL2, BCL11B, MARVELD3, FAT2, TOR1A, SCNN1G, DCX, SELPLG, INPP5B, PLET1, SWAP70, CREB1, AXL, LMNA, TNP1, DPYSL4, RCAN1, ATP1A2, FUCA2, SNAI1, BRAT1, TRIM21, CDH13, PPP1R9B, RPL13A, GFRA1, IGFBP3, HTR2C, RNF41, TP53INP1 | 1.62365569 | 1.83E-06 | 9.13E-08 | 4.76E-07 |
| GO:0035556~intracellular signal transduction | 234 | 17.15542522 | 2.81E-10 | PLEKHM1, ADCY5, LHCGR, LPAR2, RORA, ITSN1, RAB29, GAB1, CREB3L1, RAB21, DBNL, DAB2IP, ROCK1, PTPRR, RAP1GDS1, RAD9A, UBR1, PCLO, FARP2, HNF4A, SERPINF2, VEGFA, PDGFRA, PDGFRB, RALGPS2, RALGPS1, GCNT2, GNAI2, DAG1, NFKBIA, BDKRB2, GPR143, BMP3, ARHGEF33, ABR, MAP2K1, IL1RN, SMAD4, MSTN, HGF, OXSR1, EPHA4, NOTCH2, EI24, NOTCH1, RGS6, KLF4, BMP8B, OPRM1, WASF1, SHOC2, TMEM109, STAC2, TKFC, NMUR1, MAPT, GUCY1A3, TGFA, AGAP2, MYC, ALS2CL, ARHGEF3, PGAP2, RAB4A, TP53, ARHGEF9, PI4KB, NFAM1, IL6R, MYADM, ELMO1, INHBB, MAST3, ZDHHC17, MAP3K15, KSR2, SEMA4C, PLA2G6, KSR1, MAP3K14, FKBP11, ZMYND11, RAB3B, USP8, RAP1GAP, CCR1, FKBP1A, KIT, FKBP1B, TPCN2, CCL22, PLEKHG2, RAB43, DGKB, XBP1, FAT4, BCL2, BCL6, RASA4, NOS1, AXL, SPSB4, ATP1A2, DGKI, MID1, SNAI1, TP73, CDH13, DUSP4, RPS6KA4, RAB36, MAPK13, PLN, NEUROD2, DGKZ, ADGRL1, HTR2C, HTR2A, SLC44A2, TUFT1, TNFSF14, RASGEF1C, CXCL11, CXCL10, WNT1, ANK2, MDFIC, GATA3, RRAS, SPRED1, RNF34, PAG1, DISC1, RET, STMN3, PSD3, FGF23, SOCS4, IRS1, MAP4K4, TNS2, GPR55, RELT, TRIM32, ASB1, AKAP6, RELN, STMN1, ERC1, ASB4, GLP1R, CAV3, SYVN1, DRD2, SIPA1, SOX4, ULK4, SRC, NR2C2, IRAK4, PPP1R16B, ECE1, TRIM67, GMIP, STK38L, FGD6, FGD3, RHOBTB3, CSF1R, DIXDC1, MET, ARPP19, DOCK9, DOCK8, TRIM62, DOCK3, IKBKE, PDE7B, UACA, PLCG1, HDAC1, NEDD4, RHOJ, NDST1, SGPP1, MRVI1, RHOQ, RHOV, PDCD4, HPSE, CDIP1, ARHGAP1, WWC3, LTF, PLCB1, CASP2, IRAK2, CCDC88A, PRKCH, EDAR, DAPK2, PRKCE, RASL12, ARHGAP26, PRKCB, PRKD1, MFN2, PRKCQ, UBE2K, MDM4, RTN4RL1, CYTH4, PPM1A, PPP3R1, KITLG, MOAP1, RASGRP4, MARVELD3, NFATC4, INPP5D, DCX, THPO, PIK3R2, PTPRD, DNM1L, RCAN1, SIRT1, RALGDS, PPP1R9B, PRLR, SMPD1, IRF1, IGFBP3, RNF41 | 1.471933795 | 2.14E-06 | 1.02E-07 | 5.58E-07 |
| GO:0045935~positive regulation of nucleobase-containing compound metabolic process | 182 | 13.3431085 | 7.58E-10 | NCBP2, RNASEL, LHCGR, RORA, JAG1, CXCL11, CXCL10, NLRC5, WNT1, DDX17, MYOCD, MDFIC, GATA3, CREB3L1, RARB, PID1, SATB2, RET, DAB2IP, MTA2, STRN3, YY1, SIX3, FGF23, DLL1, HNF4G, GRHL2, PPARGC1B, CRHR1, HNF4A, MTF1, SERPINF2, MCIDAS, VEGFA, MC2R, TRIM32, PDGFRA, CELF3, PDGFRB, NFE2L1, RELN, GLP1R, SUPT6H, CAMTA1, HOXA13, DRD2, CRTC1, ONECUT2, SOX4, NFKBIA, MYT1, SRC, NR2C2, PLAGL1, SLX4, ECE1, OVOL2, TNKS, PLAG1, ASXL2, KLF6, BMP3, ESRRA, MAP2K1, MET, NR4A2, SMAD4, MSTN, SKI, WHSC1, HGF, FOXP3, USF1, TRIM62, FOXP1, NOTCH3, CDKN1C, NOTCH1, NR1I2, CSRNP3, HDAC1, GTF2F1, TRPS1, ZIC5, RBMXL1, AREG, TCF12, FOXI1, KLF4, E2F3, HNF1A, ARID4A, CPEB3, MITF, PPARG, ARID4B, RHOQ, ZEB1, LGR4, TMEM173, MAZ, PRMT2, PAX7, RTF1, LTF, GUCY1A3, MKX, LRRFIP1, PLCB1, AGAP2, MYC, FOSL1, IRAK2, KDM7A, TBL1XR1, FOXJ2, TP53, PRKCH, LEF1, PADI2, ERLIN1, FOSB, IL6R, SPEN, NFAM1, DDN, MYCN, PRKCB, PRKD1, PRKCQ, NCOA1, MYRF, CUX1, NSD1, CLOCK, CALCR, ABLIM1, KMT2D, ABLIM3, UNG, PPM1A, PPP3R1, KITLG, CTCFL, KIT, CALCA, RGMB, XBP1, BCL11B, POU2F1, CHD1, NFATC4, BCL9L, TNRC6B, PIK3R2, NOS1, CREB1, LMNA, CREB5, SNAI1, TMPRSS6, ATMIN, SIRT1, TRIM21, ATXN7L3, TP73, PREB, CDH13, RPS6KA4, PKNOX1, OBFC1, ATXN7, HEYL, IRF1, NEUROD2, KDM8, IRF4, IGFBP3, TP53INP2, RNF41, TP53INP1, HTR2A | 1.554958557 | 5.79E-06 | 2.63E-07 | 1.51E-06 |
| GO:0015031~protein transport | 168 | 12.31671554 | 1.34E-09 | CHMP3, SRCIN1, PLEKHM1, VTCN1, XPO5, LMAN2L, XPO4, ADCY5, CHMP7, LHCGR, VPS52, TNFSF14, SYT9, ILDR2, SIDT2, PNP, KIF13A, SLC16A1, CRY2, ANK2, ATG5, ANK3, MDFIC, RAB29, GATA3, AAGAB, MS4A2, VPS4A, SAR1A, RAB21, DAB2IP, ATG9A, GBP5, SIX3, MYH9, CDKL2, PCLO, PKIA, IRS1, VTI1A, MAP4K4, HNF4A, LYST, SURF4, AKAP6, ATPIF1, VAMP2, CLIP3, ERC1, GLP1R, PACS1, SNX9, SYVN1, DRD2, COPZ1, CACNB1, DAG1, SOX4, NFKBIA, SNX4, MYT1, LMAN1, SRC, OAZ2, STX17, GOLPH3L, AXIN2, SEC61A1, CSF1R, UNC119, MYO1C, IL1RN, PTPN14, SMAD4, FOXP3, FOXP1, NOTCH2, NOTCH1, UACA, AKTIP, HDAC1, PLCG1, NEDD4, CACNA1E, MARCKS, SPCS2, OPRM1, CREBRF, HNF1A, AP1B1, DPH6, PPARG, AP3S2, PEX3, PIP5K1A, RHOV, LGR4, TMEM173, AP1S2, NMUR1, PEX2, NEURL1B, AGAP2, GPIHBP1, JAKMIP1, KDELR3, PGAP2, STX1A, ANP32B, ZDHHC8, RAB4A, TP53, CFTR, SRPRA, PFKM, EDAR, PRKCE, MYADM, BBS1, MFN2, INHBB, PRKCQ, ACVR2B, ZDHHC17, MYRIP, DGAT1, ATG4B, TOM1, PLA2G6, TREM1, FKBP11, CLOCK, CALCR, RAB3B, SNX15, ABLIM3, ATG13, PPM1A, PPP3R1, BET1, HK1, FKBP1B, RAB43, XBP1, NUP210, TOR1A, CHM, PER2, BCL6, LYPLAL1, ELMOD1, PIK3R2, SPRN, NOS1, CNST, DNM1L, LMNA, LRRC46, MAL, HID1, SIRT1, PREB, COG3, COG6, RAB36, KCNN3, MCFD2, SVIP | 1.578323941 | 1.03E-05 | 4.46E-07 | 2.67E-06 |
| GO:0033036~macromolecule localization | 240 | 17.59530792 | 1.40E-09 | CHMP3, VTCN1, PPP2R5A, PLEKHM1, ADCY5, CHMP7, LHCGR, VPS52, SYT9, SIDT1, SIDT2, HOOK3, KIF13A, CRY2, RAB29, AAGAB, VPS4A, RAB21, DAB2IP, GBP5, ROCK1, YY1, MYH9, PCLO, VTI1A, GLTP, CRHR1, HNF4A, VEGFA, SURF4, UBL4A, ATPIF1, SUPT6H, CACNB1, DAG1, NFKBIA, BDKRB2, MYT1, ACE, STX17, GOLPH3L, TNKS, FCHO2, UNC119, GPR158, IL1RN, SMAD4, PTPN14, GAS8, ABCG4, NOTCH2, NOTCH1, DCP1A, MARCKS, OPRM1, CREBRF, LZTS2, HNF1A, AP1B1, GLRA3, PPARG, AP3S2, AP1S2, NMUR1, NEURL1B, GPIHBP1, AGAP2, ALS2CL, JAKMIP1, KDELR3, SH3PXD2B, PGAP2, STX1A, ANP32B, FLOT2, RAB4A, ZDHHC8, TP53, CFTR, ARHGEF9, MYADM, INHBB, ZDHHC15, ZDHHC17, MYRIP, CLIC5, PLA2G6, FKBP11, CLOCK, SLC27A4, RAB3B, CLDN18, ABCD1, PPFIA1, ATG13, BET1, HK1, FKBP1B, NETO1, ZDHHC23, ACSL1, RAB43, XBP1, NUP210, CHM, PER2, BCL6, LYPLAL1, SLCO3A1, ACSL4, ELMOD1, NOS1, CNST, LMNA, LRRC46, MID1, MARCH5, PREB, COG3, COG6, RAB36, MCFD2, SVIP, TP53INP2, LRP4, HTR2A, NCBP2, SRCIN1, XPO5, LMAN2L, XPO4, TNFSF14, ILDR2, PNP, VCL, SLC16A1, ATG5, ANK2, MDFIC, ANK3, GATA3, MS4A2, SAR1A, DISC1, PID1, ATG9A, SIX3, CDKL2, PKIA, IRS1, MAP4K4, PNPLA8, LYST, AKAP6, RELN, VAMP2, CLIP3, ERC1, GLP1R, CAV3, PACS1, SNX9, SYVN1, DRD2, PPIL2, COPZ1, SOX4, SNX4, LMAN1, SRC, SLCO2A1, OAZ2, AXIN2, SEC61A1, CSF1R, MYO1C, CRB3, WHSC1, WIPI2, FOXP3, FOXP1, CORO1C, UACA, PLCG1, HDAC1, AKTIP, NEDD4, CACNA1E, SPCS2, DPH6, RHOQ, PEX3, PIP5K1A, RHOV, LGR4, TMEM173, PEX2, DLG3, CNTNAP2, CNTNAP1, LDAH, NRXN2, KIF17, SRPRA, PFKM, EDAR, PRKCE, BBS1, MFN2, PRKCQ, ACVR2B, DGAT1, ATG4B, CNTN2, TOM1, TREM1, CALCR, SNX15, ABLIM3, PPM1A, PPP3R1, MOAP1, TSPAN33, TMEM33, MARVELD3, TOR1A, NEDD4L, PIK3R2, SPRN, DNM1L, ASIC2, TSPAN14, MAL, TMBIM1, HID1, SLC10A2, SIRT1, PPP1R9B, CDAN1, KCNN3 | 1.438646786 | 1.07E-05 | 4.44E-07 | 2.78E-06 |
| GO:0060284~regulation of cell development | 118 | 8.651026393 | 1.53E-09 | SYT1, SRCIN1, ADCY5, JAG1, HOOK3, S1PR2, S1PR3, BRINP1, GATA3, RAB29, RARB, DISC1, RAB21, LRRC7, DAB2IP, RET, ROCK1, ZHX2, SIX3, DLL1, NUMBL, MAP4K4, TRIM32, FOXG1, VEGFA, AKAP6, TGIF2, RELN, CAV3, GCNT2, STRAP, DRD2, CRTC1, DAG1, ULK4, LIN28A, EPHB2, ACE, TRIM67, OVOL2, AXIN2, PLAG1, DIXDC1, MAP2K1, MET, SMAD4, SKI, HGF, TRIM62, CORO1C, NOTCH3, SEMA6A, EPHA4, NOTCH1, DLX1, HDAC1, NEDD4, RGS6, MARCKS, TCF12, KLF4, ARSB, OPRM1, GFAP, CPEB3, SYNJ1, PPARG, ZEB1, FES, MBP, XYLT1, MAPT, SEMA3E, EEF2K, ANP32A, MYC, CCDC88A, TP53, LEF1, PRKCH, SPEN, MYADM, MYCN, PRKD1, NCOA1, ANKRD23, SEMA4F, CNTN2, WNT9B, SEMA4C, SEMA4B, CUX1, CLOCK, SRGAP2, CLDN18, RAP1GAP, KIT, FKBP1B, FAT3, BCL2, BCL11B, PER2, BCL6, NFATC4, BCL9L, NEDD4L, TLX2, PTPRD, NOS1, CREB1, SNAI1, SIRT1, TP73, DKK1, HEYL, NEUROD2, KDM4C, LRP4 | 1.757817338 | 1.17E-05 | 4.68E-07 | 3.05E-06 |
| GO:0023056~positive regulation of signaling | 159 | 11.6568915 | 1.98E-09 | SYT1, SLC44A2, SNCA, LHCGR, TNFSF14, LPAR2, ILDR1, JAG1, ITSN1, CXCL11, CXCL10, SHKBP1, NLRC5, WNT1, DDX17, PTGIS, MYOCD, MDFIC, GATA3, GAB1, SPRED1, LGI1, DISC1, RET, DAB2IP, FGF23, DLL1, RAD9A, IRS1, CRHR1, GPR55, HNF4A, RELT, SERPINF2, TRIM32, VEGFA, PDGFRA, PDGFRB, AKAP6, ATPIF1, RELN, VAMP2, GLP1R, NETO2, GCNT2, PPP2R3A, GNAI2, DRD2, CRTC1, SOX4, SNX4, SRC, IRAK4, PEA15, AMER1, ECE1, RNF165, TNKS, BMF, CSF1R, ASXL2, DIXDC1, BMP3, MYO1C, MAP2K1, IL1RN, MET, ARPP19, SMAD4, MSTN, HGF, GAS1, TRIM62, CDKN1C, NOTCH2, EPHA4, IKBKE, EI24, NOTCH1, HDAC1, NEDD4, TRPS1, TMOD2, BMP8B, NAT8L, CREBRF, OPRM1, GFAP, SHOC2, LGR4, RSPO1, HPSE, LTF, TGFA, PLCB1, AGAP2, MYC, CASP2, IRAK2, STX1A, TP53, PRKCH, CFTR, IL6R, EDAR, NFAM1, PFKM, PRKCE, PRKCB, PRKD1, INHBB, PRKCQ, ACVR2B, ZDHHC17, MYRIP, KSR2, MAP3K15, UBE2K, CNTN2, SEMA4C, PLA2G6, KSR1, MAP3K14, KMT2D, RAB3B, CCR1, PPM1A, KITLG, FKBP1A, KIT, NETO1, CCL22, MOAP1, TSPAN33, XBP1, RASGRP4, LRRTM2, TOR1A, NFATC4, MLLT3, THPO, NOS1, DNM1L, CREB1, AXL, TSPAN14, MAL, DGKI, MID1, SIRT1, TP73, WNT2B, CDH13, DKK1, NEUROD2, IGFBP3, HTR2C, TP53INP1, HTR2A | 1.594868625 | 1.51E-05 | 5.80E-07 | 3.93E-06 |
| GO:0051254~positive regulation of RNA metabolic process | 162 | 11.87683284 | 2.03E-09 | NCBP2, RNASEL, RORA, JAG1, NLRC5, WNT1, DDX17, MYOCD, MDFIC, GATA3, CREB3L1, RARB, PID1, SATB2, RET, DAB2IP, MTA2, STRN3, YY1, SIX3, FGF23, DLL1, HNF4G, GRHL2, PPARGC1B, HNF4A, MTF1, SERPINF2, MCIDAS, TRIM32, VEGFA, CELF3, NFE2L1, RELN, GLP1R, SUPT6H, CAMTA1, HOXA13, DRD2, CRTC1, ONECUT2, SOX4, NFKBIA, MYT1, SRC, NR2C2, PLAGL1, OVOL2, TNKS, ASXL2, PLAG1, KLF6, BMP3, ESRRA, MAP2K1, MET, NR4A2, SMAD4, MSTN, SKI, FOXP3, USF1, TRIM62, FOXP1, NOTCH3, CDKN1C, NOTCH1, NR1I2, CSRNP3, HDAC1, GTF2F1, TRPS1, ZIC5, RBMXL1, TCF12, FOXI1, KLF4, E2F3, HNF1A, ARID4A, CPEB3, MITF, PPARG, ARID4B, RHOQ, ZEB1, LGR4, TMEM173, MAZ, PRMT2, PAX7, RTF1, LTF, MKX, LRRFIP1, PLCB1, AGAP2, MYC, FOSL1, IRAK2, KDM7A, TBL1XR1, FOXJ2, TP53, PRKCH, LEF1, PADI2, ERLIN1, FOSB, IL6R, SPEN, NFAM1, DDN, MYCN, PRKCB, PRKD1, PRKCQ, NCOA1, MYRF, CUX1, NSD1, CLOCK, ABLIM1, KMT2D, ABLIM3, PPP3R1, PPM1A, CTCFL, KIT, RGMB, XBP1, BCL11B, POU2F1, CHD1, NFATC4, BCL9L, TNRC6B, PIK3R2, NOS1, CREB1, LMNA, CREB5, SNAI1, TMPRSS6, SIRT1, ATMIN, TRIM21, ATXN7L3, TP73, PREB, CDH13, RPS6KA4, PKNOX1, ATXN7, HEYL, IRF1, NEUROD2, KDM8, IRF4, TP53INP2, RNF41, TP53INP1 | 1.58554096 | 1.55E-05 | 5.75E-07 | 4.04E-06 |
| GO:1902680~positive regulation of RNA biosynthetic process | 158 | 11.58357771 | 2.05E-09 | RNASEL, RORA, JAG1, NLRC5, WNT1, DDX17, MYOCD, MDFIC, GATA3, CREB3L1, RARB, PID1, SATB2, RET, DAB2IP, MTA2, STRN3, YY1, SIX3, FGF23, DLL1, HNF4G, GRHL2, PPARGC1B, HNF4A, MTF1, SERPINF2, MCIDAS, TRIM32, VEGFA, NFE2L1, RELN, GLP1R, SUPT6H, CAMTA1, HOXA13, DRD2, CRTC1, ONECUT2, SOX4, NFKBIA, MYT1, SRC, NR2C2, PLAGL1, OVOL2, TNKS, ASXL2, PLAG1, KLF6, BMP3, ESRRA, MAP2K1, MET, NR4A2, SMAD4, MSTN, SKI, FOXP3, USF1, TRIM62, FOXP1, NOTCH3, CDKN1C, NOTCH1, NR1I2, CSRNP3, HDAC1, GTF2F1, TRPS1, ZIC5, RBMXL1, TCF12, FOXI1, KLF4, E2F3, HNF1A, ARID4A, MITF, PPARG, ARID4B, RHOQ, ZEB1, LGR4, TMEM173, MAZ, PRMT2, PAX7, RTF1, LTF, MKX, LRRFIP1, PLCB1, AGAP2, MYC, FOSL1, IRAK2, KDM7A, TBL1XR1, FOXJ2, TP53, PRKCH, LEF1, PADI2, ERLIN1, FOSB, IL6R, SPEN, NFAM1, DDN, MYCN, PRKCB, PRKD1, PRKCQ, NCOA1, MYRF, CUX1, NSD1, CLOCK, ABLIM1, KMT2D, ABLIM3, PPP3R1, PPM1A, CTCFL, KIT, RGMB, XBP1, BCL11B, POU2F1, CHD1, NFATC4, BCL9L, PIK3R2, NOS1, CREB1, LMNA, CREB5, SNAI1, TMPRSS6, SIRT1, ATMIN, TRIM21, ATXN7L3, TP73, PREB, CDH13, RPS6KA4, PKNOX1, ATXN7, HEYL, IRF1, NEUROD2, KDM8, IRF4, TP53INP2, RNF41, TP53INP1 | 1.598081777 | 1.56E-05 | 5.58E-07 | 4.07E-06 |
| GO:0045893~positive regulation of transcription, DNA-templated | 157 | 11.51026393 | 3.13E-09 | RNASEL, RORA, JAG1, NLRC5, WNT1, DDX17, MYOCD, MDFIC, GATA3, CREB3L1, RARB, PID1, SATB2, RET, DAB2IP, MTA2, STRN3, YY1, SIX3, FGF23, DLL1, HNF4G, GRHL2, PPARGC1B, HNF4A, MTF1, SERPINF2, MCIDAS, TRIM32, VEGFA, NFE2L1, RELN, GLP1R, SUPT6H, CAMTA1, HOXA13, DRD2, CRTC1, ONECUT2, SOX4, NFKBIA, MYT1, SRC, NR2C2, PLAGL1, OVOL2, TNKS, ASXL2, PLAG1, KLF6, BMP3, ESRRA, MAP2K1, MET, NR4A2, SMAD4, MSTN, SKI, FOXP3, USF1, TRIM62, FOXP1, NOTCH3, CDKN1C, NOTCH1, NR1I2, CSRNP3, HDAC1, GTF2F1, TRPS1, ZIC5, RBMXL1, TCF12, FOXI1, KLF4, E2F3, HNF1A, ARID4A, MITF, PPARG, ARID4B, RHOQ, ZEB1, LGR4, TMEM173, MAZ, PRMT2, PAX7, RTF1, LTF, MKX, LRRFIP1, PLCB1, AGAP2, MYC, FOSL1, IRAK2, KDM7A, TBL1XR1, FOXJ2, TP53, PRKCH, LEF1, PADI2, ERLIN1, FOSB, IL6R, SPEN, NFAM1, DDN, MYCN, PRKCB, PRKD1, PRKCQ, NCOA1, MYRF, CUX1, NSD1, CLOCK, ABLIM1, KMT2D, ABLIM3, PPP3R1, PPM1A, CTCFL, KIT, RGMB, XBP1, BCL11B, POU2F1, NFATC4, BCL9L, PIK3R2, NOS1, CREB1, LMNA, CREB5, SNAI1, TMPRSS6, SIRT1, ATMIN, TRIM21, ATXN7L3, TP73, PREB, CDH13, RPS6KA4, PKNOX1, ATXN7, HEYL, IRF1, NEUROD2, KDM8, IRF4, TP53INP2, RNF41, TP53INP1 | 1.591291761 | 2.39E-05 | 8.24E-07 | 6.23E-06 |
| GO:1903508~positive regulation of nucleic acid-templated transcription | 157 | 11.51026393 | 3.13E-09 | RNASEL, RORA, JAG1, NLRC5, WNT1, DDX17, MYOCD, MDFIC, GATA3, CREB3L1, RARB, PID1, SATB2, RET, DAB2IP, MTA2, STRN3, YY1, SIX3, FGF23, DLL1, HNF4G, GRHL2, PPARGC1B, HNF4A, MTF1, SERPINF2, MCIDAS, TRIM32, VEGFA, NFE2L1, RELN, GLP1R, SUPT6H, CAMTA1, HOXA13, DRD2, CRTC1, ONECUT2, SOX4, NFKBIA, MYT1, SRC, NR2C2, PLAGL1, OVOL2, TNKS, ASXL2, PLAG1, KLF6, BMP3, ESRRA, MAP2K1, MET, NR4A2, SMAD4, MSTN, SKI, FOXP3, USF1, TRIM62, FOXP1, NOTCH3, CDKN1C, NOTCH1, NR1I2, CSRNP3, HDAC1, GTF2F1, TRPS1, ZIC5, RBMXL1, TCF12, FOXI1, KLF4, E2F3, HNF1A, ARID4A, MITF, PPARG, ARID4B, RHOQ, ZEB1, LGR4, TMEM173, MAZ, PRMT2, PAX7, RTF1, LTF, MKX, LRRFIP1, PLCB1, AGAP2, MYC, FOSL1, IRAK2, KDM7A, TBL1XR1, FOXJ2, TP53, PRKCH, LEF1, PADI2, ERLIN1, FOSB, IL6R, SPEN, NFAM1, DDN, MYCN, PRKCB, PRKD1, PRKCQ, NCOA1, MYRF, CUX1, NSD1, CLOCK, ABLIM1, KMT2D, ABLIM3, PPP3R1, PPM1A, CTCFL, KIT, RGMB, XBP1, BCL11B, POU2F1, NFATC4, BCL9L, PIK3R2, NOS1, CREB1, LMNA, CREB5, SNAI1, TMPRSS6, SIRT1, ATMIN, TRIM21, ATXN7L3, TP73, PREB, CDH13, RPS6KA4, PKNOX1, ATXN7, HEYL, IRF1, NEUROD2, KDM8, IRF4, TP53INP2, RNF41, TP53INP1 | 1.591291761 | 2.39E-05 | 8.24E-07 | 6.23E-06 |
| GO:0051173~positive regulation of nitrogen compound metabolic process | 187 | 13.70967742 | 4.64E-09 | NCBP2, RNASEL, LHCGR, RORA, JAG1, CXCL11, CXCL10, NLRC5, WNT1, DDX17, MYOCD, MDFIC, GATA3, CREB3L1, RARB, SAMD4A, PID1, SATB2, RET, DAB2IP, MTA2, STRN3, YY1, SIX3, FGF23, DLL1, HNF4G, GRHL2, PPARGC1B, CRHR1, MTF1, HNF4A, SERPINF2, MCIDAS, VEGFA, MC2R, TRIM32, PDGFRA, CELF3, PDGFRB, NFE2L1, RELN, GLP1R, SUPT6H, CAMTA1, HOXA13, DRD2, CRTC1, ONECUT2, SOX4, NFKBIA, MYT1, LIN28A, SRC, NR2C2, PLAGL1, SLX4, ECE1, OVOL2, TNKS, PLAG1, ASXL2, KLF6, BMP3, ESRRA, MAP2K1, MET, NR4A2, SMAD4, MSTN, SKI, WHSC1, HGF, FOXP3, USF1, TRIM62, FOXP1, NOTCH3, CDKN1C, NOTCH1, NR1I2, CSRNP3, HDAC1, TRPS1, GTF2F1, ZIC5, RBMXL1, AREG, TCF12, FOXI1, KLF4, OPRM1, E2F3, HNF1A, ARID4A, CPEB3, MITF, PPARG, ARID4B, RHOQ, ZEB1, LGR4, TMEM173, MAZ, PRMT2, PAX7, RTF1, LTF, GUCY1A3, MKX, LRRFIP1, PLCB1, AGAP2, MYC, FOSL1, IRAK2, KDM7A, TBL1XR1, FOXJ2, TP53, PRKCH, LEF1, PADI2, ERLIN1, FOSB, IL6R, SPEN, NFAM1, DDN, MYCN, PRKCB, PRKD1, PRKCQ, NCOA1, MYRF, PLA2G6, CUX1, NSD1, CLOCK, CALCR, ABLIM1, KMT2D, ABLIM3, UNG, PPM1A, PPP3R1, KITLG, CTCFL, KIT, CALCA, RGMB, MPV17L2, XBP1, BCL11B, POU2F1, CHD1, NFATC4, BCL9L, TNRC6B, PIK3R2, NOS1, CREB1, LMNA, CREB5, SNAI1, TMPRSS6, ATMIN, SIRT1, TRIM21, ATXN7L3, TP73, PREB, CDH13, RPS6KA4, PKNOX1, OBFC1, ATXN7, HEYL, IRF1, NEUROD2, KDM8, IRF4, IGFBP3, TP53INP2, RNF41, TP53INP1, HTR2A | 1.508917355 | 3.54E-05 | 1.18E-06 | 9.23E-06 |
| GO:0031328~positive regulation of cellular biosynthetic process | 184 | 13.48973607 | 5.08E-09 | RNASEL, SNCA, LHCGR, RORA, JAG1, NLRC5, WNT1, DDX17, MYOCD, MDFIC, GATA3, CREB3L1, RARB, SAMD4A, PID1, SATB2, RET, DAB2IP, MTA2, STRN3, YY1, SIX3, FGF23, DLL1, HNF4G, GRHL2, IRS1, PPARGC1B, CRHR1, MTF1, HNF4A, SERPINF2, MCIDAS, VEGFA, MC2R, TRIM32, PDGFRA, PDGFRB, NFE2L1, RELN, GLP1R, SUPT6H, CAMTA1, HOXA13, DRD2, CRTC1, ONECUT2, SOX4, NFKBIA, MYT1, LIN28A, SRC, NR2C2, PLAGL1, ECE1, OVOL2, TNKS, PLAG1, ASXL2, KLF6, BMP3, ESRRA, MAP2K1, MET, NR4A2, ARPP19, SMAD4, MSTN, SKI, HGF, FOXP3, USF1, TRIM62, FOXP1, NOTCH3, CDKN1C, NOTCH1, NR1I2, CSRNP3, HDAC1, GTF2F1, TRPS1, ZIC5, RBMXL1, AREG, TCF12, FOXI1, KLF4, OPRM1, E2F3, HNF1A, ARID4A, CPEB3, MITF, PPARG, ARID4B, RHOQ, ZEB1, LGR4, TMEM173, MAZ, PRMT2, PAX7, RTF1, LTF, GUCY1A3, MKX, LRRFIP1, PLCB1, AGAP2, MYC, FOSL1, IRAK2, KDM7A, TBL1XR1, FOXJ2, TP53, PRKCH, LEF1, PADI2, ERLIN1, FOSB, IL6R, SPEN, NFAM1, DDN, MYCN, PRKCB, PRKD1, PRKCQ, NCOA1, DGAT1, MYRF, PLA2G6, CUX1, NSD1, CLOCK, CALCR, ABLIM1, KMT2D, ABLIM3, PPM1A, PPP3R1, KITLG, CTCFL, KIT, CALCA, RGMB, MPV17L2, XBP1, BCL11B, POU2F1, CHD1, NFATC4, BCL9L, PIK3R2, NOS1, CREB1, LMNA, CREB5, SNAI1, TMPRSS6, ATMIN, SIRT1, TRIM21, ATXN7L3, TP73, PREB, CDH13, RPS6KA4, PKNOX1, OBFC1, ATXN7, HEYL, IRF1, NEUROD2, KDM8, SVIP, IRF4, HTR2C, TP53INP2, RNF41, TP53INP1, HTR2A | 1.513294577 | 3.88E-05 | 1.25E-06 | 1.01E-05 |
| GO:0007267~cell-cell signaling | 112 | 8.211143695 | 7.79E-09 | SYT1, KCNC3, SLC6A1, ADCY5, SLC6A3, SNCA, SYT9, ILDR2, ILDR1, SIDT2, CALB1, S1PR2, WNT1, SLC16A1, CRY2, ANK2, GATA3, LGI1, SIX3, FGF23, DLL1, IRS1, PCLO, CRHR1, MAP4K4, UCN2, HNF4A, RELN, VAMP2, GLP1R, NETO2, GNAI2, DRD2, CRTC1, CACNB1, SOX4, SNX4, CACNB3, MYT1, RIMS4, RIMS3, EPHB2, GRID2IP, GABRA3, MET, IL1RN, SMAD4, HGF, CACNA2D2, HDAC1, TMOD2, CACNA1E, ADRA1D, NAT8L, OPRM1, GFAP, JPH3, HNF1A, CPEB3, GLRA3, SYNJ1, PPARG, BCAN, LGR4, WISP2, TRIM9, MAPT, DLG3, STX1A, NOS1AP, NRXN2, CNTNAP4, CFTR, PFKM, PRKCE, INHBB, ACVR2B, MYRIP, DGAT1, GRM7, CNTN2, PLA2G6, CLOCK, CPLX2, RAB3B, CCR1, TH, CTNND2, KIT, FKBP1B, NETO1, HMMR, SHISA7, XBP1, LRRTM2, SYN2, TOR1A, POU2F1, PER2, NFATC4, NOS1, NCDN, CREB1, DGKI, SIRT1, WNT2B, DKK1, PNOC, NEUROD2, ADGRL1, HTR2C, HTR2A | 1.73981377 | 5.94E-05 | 1.86E-06 | 1.55E-05 |
| GO:0006357~regulation of transcription from RNA polymerase II promoter | 181 | 13.26979472 | 8.15E-09 | RNASEL, JAG1, RORA, NLRC5, WNT1, DDX17, CRY2, OLIG3, MYOCD, GATA3, WDR77, MED27, CREB3L1, RARB, TWIST2, PID1, SATB1, SATB2, DAB2IP, MTA2, STRN3, YY1, SIX3, ZHX2, DLL1, GRHL2, PKIA, PPARGC1B, MED19, MTF1, HNF4A, SERPINF2, MCIDAS, VEGFA, FOXG1, MNT, NFE2L1, TGIF2, ATXN1L, GLP1R, SUPT6H, CAMTA1, HOXA13, DRD2, STRAP, CRTC1, TFCP2L1, ONECUT2, SOX4, NFKBIA, MYT1, GREM2, NR2C2, PLAGL1, TAL2, FOXQ1, OVOL2, RNF165, OVOL1, TFDP2, TNKS, PLAG1, ASXL2, KLF6, BMP3, ESRRA, MAP2K1, MET, NR4A2, SMAD4, KLF17, SKI, WHSC1, FOXP3, UBP1, USF1, FOXP1, ZBTB42, FOXP2, NOTCH3, CDKN1C, NOTCH2, DLX1, NOTCH1, NR1I2, CSRNP3, HDAC1, NEDD4, TRPS1, GTF2F1, ATF7, JAZF1, RBMXL1, TCF12, FOXI1, KLF4, NCOR2, BMP8B, CREBRF, HNF1A, ARID4A, CPEB3, MITF, PPARG, ARID4B, RHOQ, ZEB1, CBFA2T3, TMEM173, MAZ, PEX2, PAX7, RTF1, WWC3, MKX, AGAP2, MYC, FOSL1, TBL1XR1, FOXJ2, TP53, LEF1, ERLIN1, FOSB, SPEN, DDN, NRIP2, PURB, MYCN, FOXR2, PRKCB, PRKD1, ACVR2B, NCOA1, CCND1, MDM4, CUX1, NSD1, CLOCK, ABLIM1, FRK, ZMYND11, KMT2D, ABLIM3, PPM1A, PPP3R1, CTCFL, RGMB, XBP1, BCL11B, POU2F1, PER2, GATAD2B, BCL6, NFATC4, BCL9L, ZSCAN29, PIK3R2, NOS1, CREB1, LMNA, CREB5, SNAI1, TMPRSS6, SIRT1, TP73, PREB, CDH13, RPS6KA4, PHF19, PKNOX1, DKK1, SP2, ATXN7, HEYL, IRF1, NEUROD2, ZBTB4, KDM4C, IRF4, LRP4 | 1.510865063 | 6.22E-05 | 1.88E-06 | 1.62E-05 |
| GO:0051094~positive regulation of developmental process | 141 | 10.3372434 | 1.15E-08 | SYT1, PDLIM7, PGF, SLC6A3, TNFSF14, JAG1, PNP, S1PR2, WNT1, BRINP1, PTGIS, MYOCD, GATA3, SERPINE1, RRAS, RARB, DISC1, RAB21, FNDC3B, LRRC7, RET, DAB2IP, DLL1, PPARGC1B, NUMBL, SLITRK4, SERPINF2, TRIM32, FOXG1, VEGFA, PDGFRB, AKAP6, TGIF2, RELN, ATXN1L, ASB4, NEK5, GLP1R, SUCO, CAV3, GCNT2, DRD2, CRTC1, DAG1, LIN28A, NR2C2, SRC, EPHB2, PPP1R16B, ACE, TRIM67, OVOL2, AXIN2, TMEM79, CSF1R, ASXL2, PLAG1, DIXDC1, ZBTB46, MAP2K1, MET, SMAD4, WHSC1, HGF, FOXP3, CACNA2D2, FOXP1, FOXP2, CORO1C, NOTCH2, EPHA4, SEMA6A, NOTCH1, HDAC1, PLCG1, RGS6, MARCKS, TCF12, ARSB, OPRM1, GFAP, CPEB3, PPARG, SYNJ1, ZEB1, FES, LGR4, HPSE, MAPT, EEF2K, LTF, PLCB1, AGAP2, MYC, SH3PXD2B, TP53, PRKCH, LEF1, SPEN, MYADM, PRKCB, PRKD1, ACVR2B, NCOA1, CCND1, MYRF, CUX1, UNG, CCR1, KITLG, KIT, FKBP1B, CALCA, FNDC5, XBP1, BCL2, LRRTM2, BCL6, BCL9L, INPP5D, NEDD4L, THPO, PTPRD, DNM1L, CREB1, ASIC2, AXL, LMNA, SNAI1, MARCH5, SIRT1, TP73, WNT2B, DKK1, HEYL, NEUROD2, KDM4C, ADGRL1, IGFBP3, HTR2C, HTR2A | 1.608744448 | 8.77E-05 | 2.58E-06 | 2.29E-05 |
| GO:0009891~positive regulation of biosynthetic process | 185 | 13.56304985 | 1.31E-08 | RNASEL, SNCA, LHCGR, RORA, JAG1, NLRC5, WNT1, DDX17, MYOCD, MDFIC, GATA3, CREB3L1, RARB, SAMD4A, PID1, SATB2, RET, DAB2IP, MTA2, STRN3, YY1, SIX3, FGF23, DLL1, HNF4G, GRHL2, IRS1, PPARGC1B, CRHR1, MTF1, HNF4A, SERPINF2, MCIDAS, VEGFA, MC2R, TRIM32, PDGFRA, PDGFRB, NFE2L1, RELN, GLP1R, SUCO, SUPT6H, CAMTA1, HOXA13, DRD2, CRTC1, ONECUT2, SOX4, NFKBIA, MYT1, LIN28A, SRC, NR2C2, PLAGL1, ECE1, OVOL2, TNKS, PLAG1, ASXL2, KLF6, BMP3, ESRRA, MAP2K1, MET, NR4A2, ARPP19, SMAD4, MSTN, SKI, HGF, FOXP3, USF1, TRIM62, FOXP1, NOTCH3, CDKN1C, NOTCH1, NR1I2, CSRNP3, HDAC1, GTF2F1, TRPS1, ZIC5, RBMXL1, AREG, TCF12, FOXI1, KLF4, OPRM1, E2F3, HNF1A, ARID4A, CPEB3, MITF, PPARG, ARID4B, RHOQ, ZEB1, LGR4, TMEM173, MAZ, PRMT2, PAX7, RTF1, LTF, GUCY1A3, MKX, LRRFIP1, PLCB1, AGAP2, MYC, FOSL1, IRAK2, KDM7A, TBL1XR1, FOXJ2, TP53, PRKCH, LEF1, PADI2, ERLIN1, FOSB, IL6R, SPEN, NFAM1, DDN, MYCN, PRKCB, PRKD1, PRKCQ, NCOA1, DGAT1, MYRF, PLA2G6, CUX1, NSD1, CLOCK, CALCR, ABLIM1, KMT2D, ABLIM3, PPM1A, PPP3R1, KITLG, CTCFL, KIT, CALCA, RGMB, MPV17L2, XBP1, BCL11B, POU2F1, CHD1, NFATC4, BCL9L, PIK3R2, NOS1, CREB1, LMNA, CREB5, SNAI1, TMPRSS6, ATMIN, SIRT1, TRIM21, ATXN7L3, TP73, PREB, CDH13, RPS6KA4, PKNOX1, OBFC1, ATXN7, HEYL, IRF1, NEUROD2, KDM8, SVIP, IRF4, HTR2C, TP53INP2, RNF41, TP53INP1, HTR2A | 1.49195034 | 9.99E-05 | 2.85E-06 | 2.60E-05 |
| GO:0006928~movement of cell or subcellular component | 167 | 12.24340176 | 1.59E-08 | PVR, DYNC1LI1, SRCIN1, TNFSF14, CXCL11, AMOTL1, VCL, CXCL10, KIF13A, CD47, ANK2, OLIG3, UNC5A, ANK3, GATA3, PDE4B, SERPINE1, GAB1, RRAS, LGI1, MTUS1, DISC1, RAB21, FNDC3B, RECK, RBFOX2, SATB2, RET, DAB2IP, PTPRM, ROCK1, SCN2B, EFNB3, TMEM201, MTA2, EFNB1, PTPRR, SIX3, SOCS7, MYH9, IRS1, NAV1, LYST, TRIM32, FOXG1, VEGFA, PDGFRA, CELF3, PDGFRB, AKAP6, RELN, STC1, STMN1, CAV3, GCNT2, PPP2R3A, STRAP, DRD2, PEAK1, ONECUT2, DAG1, ULK4, SRC, EPHB2, ALCAM, IRAK4, ACE, OVOL2, PVRL1, RNF165, ARHGDIB, CSF1R, DIXDC1, MUC2, ABR, MYO1C, MAP2K1, PODXL, IL1RN, MET, NR4A2, SMAD4, HACE1, HGF, DOCK8, GAS1, GAS8, FOXP1, CORO1C, EPHA4, SEMA6A, NOTCH1, PLCG1, CXCL16, CACNA1E, KLF4, NRP2, ARSB, MITF, AP3S2, PIP5K1A, FES, PEX2, MAPT, SEMA3E, CAP1, PLCB1, MYC, FMNL3, CCDC88A, KIF17, TP53, CHST3, LEF1, PADI2, DAPK2, PRKCE, MYADM, ELMO1, BBS1, PRKD1, PRKCQ, SEMA4F, CNTN2, SEMA4C, SEMA4B, TREM1, USP24, SRGAP2, PARVA, ABLIM1, FUT8, CCR1, KITLG, KLC2, KIT, CALCA, CCL22, FAT3, BCL2, BCL11B, MARVELD3, FAT2, TOR1A, BCL6, SCNN1G, NEDD4L, DCX, SELPLG, INPP5B, PLET1, SWAP70, AXL, LMNA, TNP1, DPYSL4, ATP1A2, SNAI1, BRAT1, CDH13, PPP1R9B, RPL13A, PLN, GFRA1, IGFBP3, RNF41, TP53INP1 | 1.528396014 | 1.21E-04 | 3.37E-06 | 3.16E-05 |
| GO:0060341~regulation of cellular localization | 141 | 10.3372434 | 1.71E-08 | NCBP2, SYT1, CHMP3, KCNC3, SRCIN1, VTCN1, PPP2R5A, ADCY5, XPO4, SNCA, LHCGR, TNFSF14, SYT9, ILDR1, CXCL11, SIDT2, CXCL10, SLC16A1, CRY2, ANK2, ATG5, ANK3, MDFIC, GATA3, RAB29, MS4A2, VPS4A, LGI3, SAR1A, RAB21, PID1, DAB2IP, FGF23, CDKL2, PKIA, IRS1, PCLO, CRHR1, MAP4K4, UCN2, HNF4A, VEGFA, AKAP6, ATPIF1, VAMP2, CLIP3, GLP1R, SUPT6H, DRD2, KCNA2, SOX4, NFKBIA, SNX4, MYT1, GPR143, SRC, RIMS4, RIMS3, OAZ2, GOLPH3L, AXIN2, CSF1R, ABR, MYO1C, MAP2K1, IL1RN, PTPN14, SMAD4, GAS1, FOXP3, FOXP1, NOTCH1, UACA, HDAC1, PLCG1, NEDD4, CACNA1E, CREBRF, OPRM1, LZTS2, HNF1A, DPH6, PPARG, RHOQ, FES, LGR4, TMEM173, NMUR1, TRIM9, MAPT, PLCB1, STX1A, ANP32B, ZDHHC8, CFTR, EDAR, PFKM, PRKCE, PRKCB, PRKD1, INHBB, PRKCQ, MYRIP, GRM7, PLA2G6, CLOCK, CLDN18, RAB3B, ABLIM3, PPFIA1, ATG13, PPP3R1, PPM1A, FKBP1A, FKBP1B, CALCA, XBP1, BCL2, SYN2, TOR1A, PER2, NEDD4L, LYPLAL1, ELMOD1, PIK3R2, NOS1, DNM1L, CNST, CREB1, LMNA, LRRC46, TMBIM1, SIRT1, PPP1R9B, KCNN3, PLN, SVIP, HTR2C, LRP4, TP53INP2, HTR2A | 1.598697644 | 1.31E-04 | 3.54E-06 | 3.41E-05 |
| GO:0006796~phosphate-containing compound metabolic process | 269 | 19.72140762 | 2.07E-08 | CTTNBP2NL, LDHA, FASTKD2, PPP2R5A, ADCY5, SNCA, PGD, LHCGR, PLPPR2, LPAR2, RORA, ITSN1, S1PR2, CRY2, GAB1, DAB2IP, PTPRM, ROCK1, PTPRR, PPARGC1B, CRHR1, NAPEPLD, HNF4A, SERPINF2, VEGFA, MC2R, PDGFRA, PDGFRB, ATPIF1, NEK5, PLA2G2F, ALDOA, GCNT2, GNAI2, PFKFB3, DAG1, BDKRB2, EPHB2, ACE, LPCAT1, SERINC1, IDH1, CDA, TNKS, GPD2, BMP3, UNC119, PTPN18, MAP2K1, IL1RN, SMAD4, PTPN14, MSTN, HGF, OXSR1, RPS6KL1, EPHA4, CSRNP3, GRK6, MARCKS, FUK, AREG, KLF4, BMP8B, PLA2G4D, OPRM1, SYNJ1, PDE11A, CCNE2, CDS2, TKFC, EEF2K, GUCY1A3, TGFA, MYC, AGAP2, PGAP2, PHKG2, TP53, PI4KB, IL6R, MYADM, INHBB, MAST3, CCND1, MAP3K15, KSR2, ADK, GRM7, SEMA4C, WNT9B, PLA2G6, MAP3K14, KSR1, NSD1, ZMYND11, FUT8, CCR1, ATG13, HK1, BCCIP, FKBP1A, KIT, FKBP1B, GCH1, CCL22, ACSL1, DGKB, XBP1, SPEG, BCL2, MLLT1, GK5, SHMT1, CNST, NOS1, PLA2G15, SWAP70, PDK3, AXL, PPP1R11, ATP1A2, DGKI, MID1, BRAT1, TP73, DUSP4, RPS6KA4, DKK1, MAPK13, SDHC, DGKZ, HTR2C, LRP4, HTR2A, RNASEL, PSTK, SRCIN1, CTDSPL, STK35, CXCL11, PNP, CXCL10, NLRC5, WNT1, MYOCD, MDFIC, PDE4B, RRAS, CTDSP2, SPRED1, SAR1A, PID1, RET, PDXK, CSNK1G1, FGF23, SOCS4, CDKL2, IRS1, PKIA, MAP4K4, PNPLA8, UCN2, RELT, GPR55, LYST, AKAP6, RELN, CLIP3, ERC1, CAV3, SNX9, DRD2, STRAP, PEAK1, EPHA10, ULK4, SRC, NR2C2, IRAK4, PPP1R16B, ECE1, SBK1, LMTK3, AXIN2, STK38L, CSF1R, DIXDC1, MET, ARPP19, AK3, AK4, LHPP, AK9, CORO1C, CDKN1C, IKBKE, AKTIP, PLCG1, HDAC1, NDST1, SGPP1, PRKAG1, RHOQ, PIP5K1A, CBFA2T3, FES, PDCD4, RSPO1, LTF, DLG3, ABHD12, PLCB1, IRAK2, TBL1XR1, PIGZ, CCDC88A, CYCS, RIMBP2, NDUFC2, PRKCH, CDK6, PFKM, DAPK2, PRKCE, TBCK, PRKCB, PRKD1, PRKCQ, ACVR2B, UBE2K, PPM1M, PLPP5, CALCR, FRK, RTN4RL1, SNX15, PPP3R1, PPM1A, CTNND1, KITLG, CALCA, MARVELD3, UCK2, INPP5D, DCX, INPP5B, EHD4, THPO, PIK3R2, PTPRB, PTPRD, NCEH1, DNM1L, CREB1, SIRT6, RCAN1, AMPD2, SIRT1, CDC25A, TMEM55A, PPP1R9B, PRLR, ATXN7, SMPD1, IRF1, IGFBP3, RNF41 | 1.363240394 | 1.58E-04 | 4.15E-06 | 4.11E-05 |
| GO:0050767~regulation of neurogenesis | 97 | 7.11143695 | 2.13E-08 | ARSB, OPRM1, SYT1, GFAP, SRCIN1, CPEB3, ADCY5, PPARG, SYNJ1, ZEB1, JAG1, FES, HOOK3, MBP, BRINP1, XYLT1, MAPT, RAB29, GATA3, SEMA3E, ANP32A, EEF2K, RARB, MYC, DISC1, RAB21, LRRC7, RET, DAB2IP, CCDC88A, TP53, SIX3, ZHX2, PRKCH, DLL1, SPEN, NUMBL, MYCN, PRKD1, MAP4K4, NCOA1, SEMA4F, VEGFA, TRIM32, FOXG1, CNTN2, SEMA4C, SEMA4B, TGIF2, RELN, CUX1, SRGAP2, RAP1GAP, DRD2, CRTC1, KIT, ULK4, LIN28A, FKBP1B, EPHB2, ACE, TRIM67, FAT3, BCL2, BCL11B, PER2, BCL6, NFATC4, NEDD4L, TLX2, PLAG1, DIXDC1, PTPRD, NOS1, MAP2K1, CREB1, MET, SKI, HGF, SIRT1, TP73, NOTCH3, EPHA4, SEMA6A, DLX1, NOTCH1, DKK1, HDAC1, NEDD4, HEYL, RGS6, NEUROD2, KDM4C, MARCKS, TCF12, LRP4, KLF4 | 1.792443769 | 1.63E-04 | 4.17E-06 | 4.24E-05 |
| GO:0006366~transcription from RNA polymerase II promoter | 172 | 12.60997067 | 2.44E-08 | RNASEL, JAG1, RORA, NLRC5, WNT1, DDX17, CRY2, OLIG3, MYOCD, GATA3, CREB3L1, RARB, TWIST2, PID1, SATB1, SATB2, DAB2IP, MTA2, STRN3, YY1, SIX3, ZHX2, DLL1, GRHL2, PKIA, PPARGC1B, ELL2, HNF4A, MTF1, SERPINF2, MCIDAS, VEGFA, FOXG1, MNT, NFE2L1, TGIF2, ATXN1L, GLP1R, SUPT6H, CAMTA1, HOXA13, DRD2, STRAP, CRTC1, TFCP2L1, ONECUT2, SOX4, NFKBIA, MYT1, GREM2, NR2C2, PLAGL1, OVOL2, RNF165, OVOL1, TNKS, PLAG1, ASXL2, KLF6, BMP3, ESRRA, MAP2K1, TAF5, MET, NR4A2, SMAD4, KLF17, SKI, WHSC1, FOXP3, USF1, FOXP1, ZBTB42, FOXP2, NOTCH3, CDKN1C, NOTCH2, DLX1, NOTCH1, NR1I2, CSRNP3, HDAC1, NEDD4, TRPS1, GTF2F1, ATF7, JAZF1, RBMXL1, TCF12, FOXI1, KLF4, NCOR2, BMP8B, CREBRF, HNF1A, ARID4A, CPEB3, MITF, PPARG, ARID4B, RHOQ, ZEB1, CBFA2T3, TMEM173, MAZ, PEX2, PAX7, RTF1, WWC3, MKX, AGAP2, MYC, FOSL1, TBL1XR1, FOXJ2, TP53, LEF1, ERLIN1, FOSB, SPEN, DDN, NRIP2, PURB, MYCN, PRKD1, ACVR2B, NCOA1, CCND1, MDM4, CUX1, NSD1, CLOCK, ABLIM1, FRK, ZMYND11, KMT2D, POLR2F, ABLIM3, PPM1A, PPP3R1, CTCFL, RGMB, XBP1, BCL11B, POU2F1, PER2, GATAD2B, BCL6, NFATC4, BCL9L, PIK3R2, NOS1, CREB1, LMNA, CREB5, SNAI1, TMPRSS6, SIRT1, TP73, PREB, CDH13, RPS6KA4, PHF19, PKNOX1, DKK1, ATXN7, HEYL, IRF1, NEUROD2, ZBTB4, IRF4, LRP4 | 1.507656105 | 1.86E-04 | 4.65E-06 | 4.85E-05 |
| GO:0000904~cell morphogenesis involved in differentiation | 96 | 7.038123167 | 2.54E-08 | NRP2, SRCIN1, FGFRL1, MBP, VCL, GP5, ANK3, UNC5A, MAPT, GATA3, SEMA3E, EEF2K, LRRC55, LGI1, DISC1, RAB21, FNDC3B, RBFOX2, DAB2IP, RET, PTPRM, ROCK1, EFNB3, EFNB1, LEF1, COL25A1, MYH9, MYADM, GRHL2, NUMBL, BBS1, SLITRK4, VSIG1, SEMA4F, CLIC5, FOXG1, VEGFA, CNTN2, WNT9B, SEMA4C, SEMA4B, STC1, RELN, STMN1, JMJD1C, CUX1, SRGAP2, PARVA, ABLIM1, GCNT2, HOXA13, STRAP, DRD2, PEAK1, CTNND2, PPP3R1, DAG1, SRC, EPHB2, ALCAM, NPTX1, FAT3, OVOL2, PVRL1, RNF165, BCL2, BCL11B, NFATC4, BCL9L, DCX, AXIN2, TLX2, CSF1R, DIXDC1, PTPRD, AKIP1, MAP2K1, CREB1, MET, NR4A2, SMAD4, AXL, GAS1, TRIM62, SNAI1, RAPH1, FOXP1, CORO1C, EPHA4, SEMA6A, NOTCH1, DKK1, NEDD4, HEYL, MARCKS, LRP4 | 1.792206253 | 1.94E-04 | 4.73E-06 | 5.05E-05 |
| GO:0031175~neuron projection development | 107 | 7.84457478 | 2.62E-08 | SYT1, SRCIN1, ADCY5, VCL, GP5, ANK3, UNC5A, GATA3, RAB29, LRRC55, LGI1, RAB21, LRRC7, DISC1, RBFOX2, DBNL, DAB2IP, RET, PTPRM, STMN3, EFNB3, EFNB1, NUMBL, MAP4K4, SLITRK4, FOXG1, TRIM32, VEGFA, RELN, STMN1, NGB, DRD2, CRTC1, DAG1, NRN1, EPHB2, CALU, ALCAM, TRIM67, PVRL1, RNF165, CSF1R, DIXDC1, MAP2K1, MET, SMAD4, NR4A2, GAS1, HGF, FOXP1, SEMA6A, EPHA4, NOTCH1, NEDD4, MARCKS, AREG, KLF4, NRP2, ARSB, GFAP, CPEB3, FES, MBP, SPRY3, XYLT1, MAPT, SEMA3E, CAMSAP1, EEF2K, ANP32A, CNTNAP2, CNTNAP1, NOS1AP, CHST3, COL25A1, BBS1, PRKD1, SEMA4F, CNTN2, SEMA4C, SEMA4B, CUX1, MAP3K14, SRGAP2, ABLIM1, CTNND2, FKBP1B, NPTX1, FAT3, BCL2, BCL11B, TOR1A, NFATC4, NEDD4L, ACSL4, DCX, TLX2, PTPRD, GNAO1, NCDN, CREB1, DPYSL4, SIRT1, RAPH1, PPP1R9B, GFRA1, LRP4 | 1.726782427 | 2.00E-04 | 4.76E-06 | 5.20E-05 |
| GO:0010557~positive regulation of macromolecule biosynthetic process | 171 | 12.53665689 | 2.76E-08 | RNASEL, RORA, JAG1, NLRC5, WNT1, DDX17, MYOCD, MDFIC, GATA3, CREB3L1, RARB, SAMD4A, PID1, SATB2, RET, DAB2IP, MTA2, STRN3, YY1, SIX3, FGF23, DLL1, HNF4G, GRHL2, IRS1, PPARGC1B, HNF4A, MTF1, SERPINF2, MCIDAS, VEGFA, TRIM32, PDGFRA, PDGFRB, NFE2L1, RELN, GLP1R, SUCO, SUPT6H, CAMTA1, HOXA13, DRD2, CRTC1, ONECUT2, SOX4, NFKBIA, MYT1, LIN28A, SRC, NR2C2, PLAGL1, OVOL2, TNKS, PLAG1, ASXL2, KLF6, BMP3, ESRRA, MAP2K1, MET, NR4A2, SMAD4, MSTN, SKI, HGF, FOXP3, USF1, TRIM62, FOXP1, NOTCH3, CDKN1C, NOTCH1, NR1I2, CSRNP3, HDAC1, GTF2F1, TRPS1, ZIC5, RBMXL1, AREG, TCF12, FOXI1, KLF4, E2F3, HNF1A, ARID4A, CPEB3, MITF, PPARG, ARID4B, RHOQ, ZEB1, LGR4, TMEM173, MAZ, PRMT2, PAX7, RTF1, LTF, MKX, LRRFIP1, PLCB1, AGAP2, MYC, FOSL1, IRAK2, KDM7A, TBL1XR1, FOXJ2, TP53, PRKCH, LEF1, PADI2, ERLIN1, FOSB, IL6R, SPEN, NFAM1, DDN, MYCN, PRKCB, PRKD1, PRKCQ, NCOA1, MYRF, CUX1, NSD1, CLOCK, ABLIM1, KMT2D, ABLIM3, PPP3R1, PPM1A, KITLG, CTCFL, KIT, RGMB, MPV17L2, XBP1, BCL11B, POU2F1, CHD1, NFATC4, BCL9L, PIK3R2, NOS1, CREB1, LMNA, CREB5, SNAI1, TMPRSS6, ATMIN, SIRT1, TRIM21, ATXN7L3, TP73, PREB, CDH13, RPS6KA4, PKNOX1, OBFC1, ATXN7, HEYL, IRF1, NEUROD2, KDM8, SVIP, IRF4, TP53INP2, RNF41, TP53INP1 | 1.507076352 | 2.11E-04 | 4.90E-06 | 5.49E-05 |
| GO:0043269~regulation of ion transport | 82 | 6.011730205 | 2.85E-08 | OPRM1, CALHM1, JPH3, KCNC3, SLC6A1, LHCGR, SNCA, CXCL11, CXCL10, TMEM109, CRY2, KCNQ3, ATG5, ANK2, ANK3, PDE4B, SERPINE1, MS4A2, SCN2B, NOS1AP, FGF23, CACNG4, CFTR, CACNG2, PRKCE, PRKCB, PRKD1, CRHR1, GRM7, CLIC5, PDGFRB, AKAP6, PLA2G6, KCNH7, STC1, RELN, VAMP2, GLP1R, CAV3, NETO2, FXYD2, SCN1A, RAB3B, GNAI2, DRD2, CCR1, KCNA2, CACNB1, HK1, SNX4, FKBP1A, CACNB3, FKBP1B, TPCN2, CALCA, ACE, KCNS2, BEST1, BCL2, TOR1A, PER2, NEDD4L, SLC31A2, ACSL4, HCN3, NOS1, GNAO1, IL1RN, ASIC2, OXSR1, ATP1A2, CACNA2D2, PLCG1, KCNJ8, NEDD4, PLN, GRK6, CACNA1E, HTR2C, SCN4A, NAT8L, HTR2A | 1.893474929 | 2.18E-04 | 4.95E-06 | 5.67E-05 |
| GO:0048584~positive regulation of response to stimulus | 189 | 13.85630499 | 3.07E-08 | PVR, RNASEL, SLC44A2, SLC6A1, SNCA, LHCGR, TNFSF14, LPAR2, JAG1, ITSN1, CXCL11, CXCL10, SHKBP1, NLRC5, WNT1, CD47, DDX17, PTGIS, ATG5, MYOCD, MDFIC, GATA3, PDE4B, SERPINE1, GAB1, MS4A2, SPRED1, DISC1, RET, DAB2IP, GBP5, FGF23, DLL1, RAD9A, IRS1, CRHR1, GPR55, RELT, HNF4A, SERPINF2, VEGFA, TRIM32, PDGFRA, PDGFRB, AKAP6, ATPIF1, RELN, PPP2R3A, GCNT2, GNAI2, DRD2, SOX4, NFKBIA, CACNB3, SNX4, SRC, NR2C2, RIMS3, IRAK4, PEA15, ACE, AMER1, ECE1, GMIP, RNF165, PVRL2, TNKS, BMF, CSF1R, ASXL2, DIXDC1, BMP3, ESRRA, MYO1C, MAP2K1, IL1RN, MET, ARPP19, SMAD4, MSTN, WHSC1, HGF, GAS1, FOXP3, TRIM62, KCNK3, FAM131B, CDKN1C, NOTCH2, EPHA4, IKBKE, EI24, NOTCH1, PLCG1, HDAC1, NEDD4, TRPS1, TMOD2, MARCKS, BMP8B, OPRM1, CREBRF, CRNKL1, SHOC2, PEX3, LGR4, TMEM173, RSPO1, HPSE, TKFC, LTF, TGFA, PLCB1, AGAP2, MYC, CASP2, IRAK2, FLOT2, TP53, CHST3, PRKCH, CFTR, IL6R, EDAR, NFAM1, DAPK2, PRKCE, ZCCHC17, PRKCB, PRKD1, INHBB, PRKCQ, ACVR2B, ZDHHC17, KSR2, MAP3K15, UBE2K, CNTN2, SEMA4C, PLA2G6, TREM1, MAP3K14, KSR1, CLOCK, KMT2D, CCR1, UNG, ATG13, PPM1A, HK1, KITLG, CALCOCO2, FKBP1A, KIT, FKBP1B, CALCB, MOAP1, CCL22, TSPAN33, XBP1, RASGRP4, BCL2, NFATC4, PAFAH1B2, MLLT3, THPO, NOS1, CR2, DNM1L, SWAP70, MAP1A, AXL, TSPAN14, MAL, DGKI, MID1, SIRT1, SGTA, TP73, WNT2B, CDH13, IRF1, NEUROD2, MEX3C, IRF4, IGFBP3, HTR2C, TP53INP1, HTR2A | 1.467183279 | 2.34E-04 | 5.20E-06 | 6.10E-05 |
| GO:0045596~negative regulation of cell differentiation | 93 | 6.818181818 | 3.46E-08 | GFAP, ADCY5, PPARG, ZEB1, JAG1, RORA, CXCL10, MBP, HOOK3, WNT1, S1PR3, MYOCD, XYLT1, GATA3, RAB29, SEMA3E, MED27, RTF1, ANP32A, MKX, RARB, MYC, TWIST2, FNDC3B, DAB2IP, ANP32B, ZHX2, TP53, SIX3, FGF23, CDK6, DLL1, GRHL2, MYCN, MAP4K4, CCND1, GPR55, SEMA4F, FOXG1, VEGFA, CNTN2, WNT9B, SEMA4C, SEMA4B, JMJD1C, SRGAP2, CAV3, CLDN18, RAP1GAP, STRAP, NFKBIA, SOX4, KIT, LIN28A, EPHB2, CALCA, FAT3, OVOL2, XBP1, BCL2, BCL6, NFATC4, BCL9L, INPP5D, AXIN2, TLX2, DIXDC1, ZBTB46, MSTN, SKI, RCAN1, FOXP3, TRIM62, SNAI1, SIRT1, COL5A2, TP73, FOXP1, CORO1C, NOTCH3, EPHA4, SEMA6A, NOTCH1, DLX1, DKK1, PHF19, HDAC1, IRF1, AREG, LRP4, KLF4, NCOR2, TOB2 | 1.801017933 | 2.64E-04 | 5.74E-06 | 6.88E-05 |
| GO:0044093~positive regulation of molecular function | 159 | 11.6568915 | 4.24E-08 | SRCIN1, SNCA, LHCGR, LPAR2, RASGEF1C, ITSN1, WNT1, ANK2, MYOCD, ANK3, MDFIC, GATA3, GAB1, MS4A2, RET, DAB2IP, PSD3, RAP1GDS1, FGF23, RIC8B, IRS1, GRHL2, PPARGC1B, FARP2, CRHR1, GPR55, SGSM2, HNF4A, TRIM32, VEGFA, PDGFRA, PDGFRB, AKAP6, RELN, NGB, NEK5, ADD2, PACS1, SNX9, FZR1, RALGPS2, RALGPS1, GNAI2, DRD2, CRTC1, SIPA1, SRC, SLX4, ACE, GMIP, SERINC1, TNKS, AXIN2, TBC1D30, FGD6, FGD3, CSF1R, HIP1, UNC119, ARHGEF33, ABR, MAP2K1, IL1RN, MET, NR4A2, DOCK9, SKI, HGF, DOCK8, OXSR1, TRIM62, DOCK3, CORO1C, TBC1D25, EPHA4, UACA, AKTIP, DCP1A, GTF2F1, PKP4, RGS6, MARCKS, DNAJB1, KLF4, PPARG, PIP5K1A, TMEM173, NMUR1, ARHGAP1, LTF, TGFA, LRRFIP1, PLCB1, AGAP2, MYC, GPIHBP1, FOSL1, ALS2CL, IRAK2, ARHGEF3, CCDC88A, ANP32B, RAB4A, CYCS, PRKCH, CFTR, IL6R, ARHGEF9, NFAM1, PRKCE, ARHGAP26, PRKCB, PRKD1, PRKCQ, ACVR2B, CCND1, MAP3K15, PSME1, DNAJC24, WNT9B, PSME3, CLOCK, SRGAP2, CALCR, APH1A, RAP1GAP, ATG13, CYTH4, KITLG, FKBP1A, KIT, GCH1, CALCA, PLEKHG2, CCL22, ACSL1, RASGRP4, ARHGAP44, BCL2, CHM, NEDD4L, RASA4, ELMOD1, TBC1D2, NOS1, GNAO1, ASIC2, SIRT1, TRIM21, RALGDS, TP73, PRLR, OBFC1, NEUROD2, IRF4, EVI5L, IGFBP3, RNF41, HTR2A | 1.525343309 | 3.24E-04 | 6.89E-06 | 8.43E-05 |
| GO:0016477~cell migration | 126 | 9.237536657 | 4.54E-08 | PVR, TNFSF14, AMOTL1, CXCL11, CXCL10, CD47, OLIG3, GATA3, PDE4B, SERPINE1, RRAS, MTUS1, FNDC3B, DISC1, RECK, RBFOX2, SATB2, DAB2IP, RET, PTPRM, ROCK1, TMEM201, MTA2, EFNB1, SIX3, PTPRR, SOCS7, MYH9, IRS1, NAV1, LYST, FOXG1, TRIM32, VEGFA, PDGFRA, PDGFRB, STC1, RELN, GCNT2, STRAP, DRD2, ONECUT2, PEAK1, DAG1, ULK4, SRC, IRAK4, ACE, OVOL2, ARHGDIB, CSF1R, MUC2, DIXDC1, ABR, MYO1C, MAP2K1, PODXL, MET, IL1RN, NR4A2, DOCK8, HGF, FOXP1, CORO1C, SEMA6A, EPHA4, NOTCH1, PLCG1, CXCL16, KLF4, NRP2, ARSB, MITF, PIP5K1A, PEX2, MAPT, SEMA3E, CAP1, PLCB1, MYC, FMNL3, CCDC88A, TP53, LEF1, PADI2, DAPK2, PRKCE, MYADM, ELMO1, BBS1, PRKD1, PRKCQ, SEMA4F, CNTN2, SEMA4C, SEMA4B, TREM1, USP24, PARVA, SRGAP2, FUT8, CCR1, KITLG, KIT, CALCA, CCL22, FAT3, BCL2, MARVELD3, FAT2, TOR1A, SCNN1G, DCX, SELPLG, PLET1, SWAP70, AXL, SNAI1, BRAT1, CDH13, PPP1R9B, RPL13A, GFRA1, IGFBP3, TP53INP1, RNF41 | 1.625281181 | 3.47E-04 | 7.22E-06 | 9.03E-05 |
| GO:0045597~positive regulation of cell differentiation | 108 | 7.917888563 | 5.14E-08 | SYT1, PDLIM7, TNFSF14, JAG1, PNP, S1PR2, BRINP1, MYOCD, GATA3, RARB, RAB21, LRRC7, FNDC3B, DISC1, DAB2IP, RET, PPARGC1B, NUMBL, SERPINF2, TRIM32, FOXG1, VEGFA, AKAP6, RELN, TGIF2, ASB4, NEK5, GLP1R, SUCO, CAV3, GCNT2, DRD2, CRTC1, LIN28A, NR2C2, EPHB2, ACE, TRIM67, OVOL2, AXIN2, CSF1R, ASXL2, PLAG1, DIXDC1, ZBTB46, MAP2K1, MET, SMAD4, HGF, FOXP3, FOXP1, SEMA6A, EPHA4, NOTCH2, NOTCH1, HDAC1, RGS6, MARCKS, TCF12, ARSB, OPRM1, GFAP, CPEB3, SYNJ1, PPARG, ZEB1, FES, MAPT, EEF2K, LTF, PLCB1, MYC, SH3PXD2B, LEF1, PRKCH, SPEN, MYADM, PRKD1, NCOA1, ACVR2B, CUX1, CCR1, KITLG, KIT, FKBP1B, CALCA, FNDC5, XBP1, BCL2, BCL6, BCL9L, NEDD4L, INPP5D, THPO, PTPRD, CREB1, LMNA, AXL, SNAI1, SIRT1, TP73, DKK1, HEYL, NEUROD2, KDM4C, HTR2C, IGFBP3, HTR2A | 1.69948919 | 3.92E-04 | 8.00E-06 | 1.02E-04 |
| GO:0051130~positive regulation of cellular component organization | 141 | 10.3372434 | 5.36E-08 | SYT1, SLC6A1, SNCA, TNFSF14, WNT1, CD47, ATG5, ANK3, GATA3, SERPINE1, VPS4A, DISC1, RAB21, LRRC7, RET, DAB2IP, GBP5, ROCK1, DLL1, CDKL2, NUMBL, SLITRK4, SERPINF2, VEGFA, SURF4, PDGFRB, ATPIF1, RELN, CLIP3, CAV3, SNX9, GCNT2, HOXA13, DRD2, CRTC1, DAG1, LMAN1, NR2C2, SRC, EPHB2, RIMS3, SLX4, TRIM67, GMIP, CNOT6L, TNKS, AXIN2, BMF, HIP1, ASXL2, DIXDC1, ABR, MYO1C, MAP2K1, MET, SMAD4, MSTN, HGF, FOXP3, FAM131B, KCNK3, CORO1C, EPHA4, NOTCH1, MARCKS, ARSB, HNF1A, CRNKL1, CPEB3, WASF1, DPH6, MITF, PPARG, SYNJ1, RHOQ, VPS37B, PEX3, FES, NMUR1, MAPT, PAX7, RTF1, EEF2K, TGFA, PLCB1, FOSL1, ZDHHC8, TP53, CHST3, LEF1, PRKCE, ZCCHC17, MYADM, PRKD1, PRKCQ, TPPP, PLA2G6, TREM1, CUX1, RAP1GAP, ABLIM3, ATG13, HK1, CALCOCO2, KIT, FKBP1B, CALCB, MOAP1, MPV17L2, XBP1, LRRTM2, TOR1A, BCL6, BCL9L, NEDD4L, ELMOD1, SYNPO, PTPRD, NOS1, DNM1L, CNST, SWAP70, CREB1, MAP1A, ASIC2, LRRC46, AXL, SNAI1, MARCH5, SIRT1, GMFB, PPP1R9B, RPS6KA4, PHF19, OBFC1, KCNN3, NEUROD2, MEX3C, ADGRL1, IGFBP3, LRP4 | 1.569296308 | 4.09E-04 | 8.18E-06 | 1.06E-04 |
| GO:0048870~cell motility | 137 | 10.04398827 | 6.11E-08 | PVR, SRCIN1, TNFSF14, CXCL11, AMOTL1, CXCL10, VCL, CD47, OLIG3, GATA3, PDE4B, SERPINE1, GAB1, RRAS, MTUS1, DISC1, FNDC3B, RECK, RBFOX2, SATB2, DAB2IP, RET, PTPRM, ROCK1, TMEM201, MTA2, EFNB1, SIX3, PTPRR, SOCS7, MYH9, IRS1, NAV1, LYST, FOXG1, TRIM32, VEGFA, CELF3, PDGFRA, PDGFRB, STC1, RELN, GCNT2, PPP2R3A, STRAP, DRD2, PEAK1, ONECUT2, DAG1, ULK4, SRC, IRAK4, ACE, OVOL2, ARHGDIB, CSF1R, DIXDC1, MUC2, ABR, MYO1C, MAP2K1, PODXL, MET, IL1RN, NR4A2, HACE1, DOCK8, HGF, GAS8, FOXP1, CORO1C, EPHA4, SEMA6A, NOTCH1, PLCG1, CXCL16, CACNA1E, KLF4, NRP2, ARSB, MITF, PIP5K1A, PEX2, MAPT, SEMA3E, CAP1, PLCB1, MYC, FMNL3, CCDC88A, TP53, LEF1, PADI2, DAPK2, PRKCE, MYADM, ELMO1, BBS1, PRKD1, PRKCQ, SEMA4F, CNTN2, SEMA4C, SEMA4B, TREM1, USP24, PARVA, SRGAP2, FUT8, CCR1, KITLG, KIT, CALCA, CCL22, FAT3, BCL2, MARVELD3, FAT2, TOR1A, SCNN1G, DCX, SELPLG, INPP5B, PLET1, SWAP70, LMNA, AXL, TNP1, SNAI1, BRAT1, CDH13, PPP1R9B, RPL13A, GFRA1, IGFBP3, RNF41, TP53INP1 | 1.579233595 | 4.66E-04 | 9.14E-06 | 1.21E-04 |
| GO:0009967~positive regulation of signal transduction | 137 | 10.04398827 | 6.11E-08 | SLC44A2, LHCGR, TNFSF14, LPAR2, JAG1, ITSN1, CXCL11, CXCL10, SHKBP1, NLRC5, WNT1, DDX17, PTGIS, MYOCD, MDFIC, GATA3, GAB1, SPRED1, DISC1, DAB2IP, RET, FGF23, RAD9A, DLL1, IRS1, GPR55, HNF4A, RELT, SERPINF2, TRIM32, VEGFA, PDGFRA, PDGFRB, AKAP6, RELN, ATPIF1, GCNT2, PPP2R3A, GNAI2, DRD2, SOX4, SRC, IRAK4, PEA15, AMER1, ECE1, RNF165, TNKS, BMF, CSF1R, ASXL2, DIXDC1, BMP3, MYO1C, MAP2K1, MET, IL1RN, ARPP19, SMAD4, MSTN, GAS1, HGF, TRIM62, CDKN1C, NOTCH2, EPHA4, IKBKE, EI24, NOTCH1, HDAC1, NEDD4, TRPS1, TMOD2, BMP8B, CREBRF, OPRM1, SHOC2, LGR4, RSPO1, HPSE, LTF, TGFA, PLCB1, AGAP2, MYC, CASP2, IRAK2, TP53, PRKCH, IL6R, EDAR, NFAM1, PRKCE, PRKCB, PRKD1, INHBB, PRKCQ, ACVR2B, ZDHHC17, KSR2, MAP3K15, UBE2K, CNTN2, SEMA4C, PLA2G6, KSR1, MAP3K14, KMT2D, CCR1, PPM1A, KITLG, FKBP1A, KIT, CCL22, MOAP1, TSPAN33, XBP1, RASGRP4, NFATC4, MLLT3, THPO, NOS1, DNM1L, TSPAN14, AXL, MAL, DGKI, MID1, SIRT1, TP73, WNT2B, CDH13, NEUROD2, IGFBP3, HTR2C, HTR2A, TP53INP1 | 1.579233595 | 4.66E-04 | 9.14E-06 | 1.21E-04 |
| GO:0051674~localization of cell | 137 | 10.04398827 | 6.11E-08 | PVR, SRCIN1, TNFSF14, CXCL11, AMOTL1, CXCL10, VCL, CD47, OLIG3, GATA3, PDE4B, SERPINE1, GAB1, RRAS, MTUS1, DISC1, FNDC3B, RECK, RBFOX2, SATB2, DAB2IP, RET, PTPRM, ROCK1, TMEM201, MTA2, EFNB1, SIX3, PTPRR, SOCS7, MYH9, IRS1, NAV1, LYST, FOXG1, TRIM32, VEGFA, CELF3, PDGFRA, PDGFRB, STC1, RELN, GCNT2, PPP2R3A, STRAP, DRD2, PEAK1, ONECUT2, DAG1, ULK4, SRC, IRAK4, ACE, OVOL2, ARHGDIB, CSF1R, DIXDC1, MUC2, ABR, MYO1C, MAP2K1, PODXL, MET, IL1RN, NR4A2, HACE1, DOCK8, HGF, GAS8, FOXP1, CORO1C, EPHA4, SEMA6A, NOTCH1, PLCG1, CXCL16, CACNA1E, KLF4, NRP2, ARSB, MITF, PIP5K1A, PEX2, MAPT, SEMA3E, CAP1, PLCB1, MYC, FMNL3, CCDC88A, TP53, LEF1, PADI2, DAPK2, PRKCE, MYADM, ELMO1, BBS1, PRKD1, PRKCQ, SEMA4F, CNTN2, SEMA4C, SEMA4B, TREM1, USP24, PARVA, SRGAP2, FUT8, CCR1, KITLG, KIT, CALCA, CCL22, FAT3, BCL2, MARVELD3, FAT2, TOR1A, SCNN1G, DCX, SELPLG, INPP5B, PLET1, SWAP70, LMNA, AXL, TNP1, SNAI1, BRAT1, CDH13, PPP1R9B, RPL13A, GFRA1, IGFBP3, RNF41, TP53INP1 | 1.579233595 | 4.66E-04 | 9.14E-06 | 1.21E-04 |
| GO:0010628~positive regulation of gene expression | 177 | 12.97653959 | 6.43E-08 | NCBP2, RNASEL, RORA, JAG1, NLRC5, WNT1, DDX17, ANK2, MYOCD, ANK3, MDFIC, GATA3, SERPINE1, CREB3L1, RARB, SAMD4A, PID1, SATB2, RET, DAB2IP, MTA2, STRN3, YY1, SIX3, FGF23, DLL1, HNF4G, MYH9, GRHL2, PPARGC1B, HNF4A, MTF1, SERPINF2, MCIDAS, VEGFA, TRIM32, CELF3, NFE2L1, RELN, GLP1R, SUPT6H, CAMTA1, HOXA13, DRD2, CRTC1, ONECUT2, SOX4, NFKBIA, MYT1, LIN28A, SRC, NR2C2, PLAGL1, OVOL2, ITGB8, TNKS, PLAG1, ASXL2, KLF6, BMP3, ESRRA, MAP2K1, MET, NR4A2, SMAD4, MSTN, SKI, FOXP3, USF1, TRIM62, FOXP1, NOTCH3, CDKN1C, NOTCH1, NR1I2, CSRNP3, HDAC1, GTF2F1, TRPS1, ZIC5, RBMXL1, TCF12, FOXI1, KLF4, E2F3, HNF1A, ARID4A, CPEB3, PRKAG1, MITF, PPARG, ARID4B, RHOQ, ZEB1, LGR4, TMEM173, MAZ, PRMT2, PAX7, RTF1, LTF, MKX, LRRFIP1, PLCB1, AGAP2, MYC, FOSL1, IRAK2, KDM7A, TBL1XR1, FOXJ2, TP53, PRKCH, LEF1, PADI2, CDK6, ERLIN1, FOSB, IL6R, SPEN, EDAR, NFAM1, DDN, MYCN, PRKCB, PRKD1, PRKCQ, NCOA1, SLC26A9, CNTN2, MYRF, CUX1, NSD1, CLOCK, ABLIM1, KMT2D, ABLIM3, PPM1A, PPP3R1, CTNND1, CTCFL, KIT, RGMB, MPV17L2, XBP1, BCL11B, POU2F1, CHD1, NFATC4, BCL9L, TNRC6B, PIK3R2, NOS1, CREB1, LMNA, CREB5, SNAI1, TMPRSS6, ATMIN, SIRT1, TRIM21, ATXN7L3, TP73, PREB, CDH13, RPS6KA4, PKNOX1, ATXN7, HEYL, IRF1, NEUROD2, KDM8, KDM4C, IRF4, TP53INP2, RNF41, TP53INP1 | 1.475779481 | 4.91E-04 | 9.44E-06 | 1.28E-04 |
| GO:0048666~neuron development | 119 | 8.724340176 | 7.51E-08 | SYT1, SRCIN1, ADCY5, VCL, GP5, ANK3, UNC5A, GATA3, RAB29, LRRC55, LGI1, DISC1, RAB21, LRRC7, RBFOX2, DBNL, DAB2IP, RET, PTPRM, STMN3, EFNB3, EFNB1, FARP2, NUMBL, MAP4K4, SLITRK4, TRIM32, FOXG1, VEGFA, RELN, STMN1, NGB, DRD2, CRTC1, ONECUT2, DAG1, NRN1, EPHB2, CALU, ALCAM, TRIM67, PVRL1, RNF165, CSF1R, DIXDC1, MAP2K1, MET, NR4A2, NTN4, SMAD4, GAS1, HGF, FOXP1, CDKN1C, EPHA4, SEMA6A, NOTCH1, NEDD4, MARCKS, AREG, KLF4, NRP2, ARSB, GFAP, CPEB3, FES, UQCRQ, MBP, SPRY3, XYLT1, MAPT, SEMA3E, CAMSAP1, EEF2K, ANP32A, CNTNAP2, CNTNAP1, NOS1AP, CHST3, COL25A1, BBS1, PRKD1, SEMA4F, CLIC5, CNTN2, SEMA4C, SEMA4B, CUX1, MAP3K14, SRGAP2, ABLIM1, TH, CTNND2, FKBP1B, NPTX1, FAT3, XBP1, FAT4, BCL2, BCL11B, TOR1A, SLC4A7, NFATC4, NEDD4L, ACSL4, DCX, TLX2, PTPRD, GNAO1, NCDN, CREB1, DPYSL4, SIRT1, RAPH1, TP73, PPP1R9B, NEUROD2, GFRA1, LRP4 | 1.638290277 | 5.73E-04 | 1.08E-05 | 1.49E-04 |
| GO:0007420~brain development | 97 | 7.11143695 | 8.19E-08 | SYT1, LDHA, SLC6A3, RORA, HOOK3, WNT1, RARB, DISC1, INA, RBFOX2, DAB2IP, SATB2, SIX3, SOCS7, DLL1, GRHL2, NUMBL, CRHR1, FOXG1, RELN, STMN1, PFKFB3, DRD2, HMGCS1, ULK4, NR2C2, SRC, EPHB2, TAL2, ACE, SBK1, CSF1R, DIXDC1, B4GALT2, ABR, MAP2K1, MET, AK3, NR4A2, ROGDI, SKI, GAS1, AK4, SLC6A17, KCNK3, FOXP2, NOTCH3, NOTCH1, DLX1, HDAC1, ZIC5, MARCKS, NCOR2, NRP2, NDST1, CRNKL1, SYNJ1, BCAN, ZEB1, UQCRQ, MBP, MAPT, CNTNAP2, PLCB1, CASP2, MYC, ANP32B, TP53, PADI2, LEF1, BBS1, INHBB, NCOA1, SEMA4C, CNTN2, FAIM2, SRGAP2, CCR1, TH, CTNND1, FAT4, BCL11B, BCL2, SEC16A, SLC4A7, PAFAH1B2, DCX, GNAO1, TRNP1, CREB1, AXL, TP73, WNT2B, PPP1R9B, DKK1, NEUROD2, ADGRL1 | 1.743639607 | 6.25E-04 | 1.16E-05 | 1.63E-04 |
| GO:0045944~positive regulation of transcription from RNA polymerase II promoter | 118 | 8.651026393 | 8.57E-08 | RNASEL, JAG1, RORA, NLRC5, WNT1, DDX17, MYOCD, GATA3, CREB3L1, RARB, PID1, SATB2, DAB2IP, MTA2, YY1, STRN3, SIX3, DLL1, GRHL2, PPARGC1B, HNF4A, MTF1, SERPINF2, MCIDAS, VEGFA, NFE2L1, GLP1R, SUPT6H, CAMTA1, HOXA13, DRD2, CRTC1, ONECUT2, NFKBIA, SOX4, MYT1, NR2C2, PLAGL1, OVOL2, TNKS, ASXL2, PLAG1, KLF6, BMP3, ESRRA, MAP2K1, MET, NR4A2, SMAD4, SKI, FOXP3, USF1, FOXP1, NOTCH3, NOTCH1, NR1I2, CSRNP3, HDAC1, GTF2F1, TRPS1, RBMXL1, TCF12, FOXI1, KLF4, HNF1A, ARID4A, MITF, ARID4B, PPARG, RHOQ, ZEB1, TMEM173, MAZ, PAX7, RTF1, MKX, FOSL1, MYC, AGAP2, TBL1XR1, FOXJ2, TP53, LEF1, ERLIN1, FOSB, DDN, MYCN, PRKD1, NCOA1, CUX1, CLOCK, ABLIM1, KMT2D, ABLIM3, PPP3R1, CTCFL, XBP1, BCL11B, POU2F1, NFATC4, BCL9L, PIK3R2, NOS1, CREB1, LMNA, CREB5, SIRT1, TMPRSS6, TP73, PREB, CDH13, RPS6KA4, PKNOX1, ATXN7, HEYL, IRF1, NEUROD2, IRF4 | 1.638500865 | 6.54E-04 | 1.19E-05 | 1.70E-04 |
| GO:0034097~response to cytokine | 87 | 6.37829912 | 9.85E-08 | CREBRF, CRNKL1, SNCA, SYNJ1, IL21R, PPARG, CXCR1, RORA, CXCL11, CXCL10, MBP, NLRC5, TMEM173, PTGIS, XYLT1, CDIP1, GATA3, SERPINE1, GAB1, PLCB1, FOSL1, MYC, PID1, IRAK2, DAB2IP, GBP5, HYAL3, YY1, TP53, ADIPOR2, FGF23, LEF1, PADI2, CFTR, SOCS4, IL6R, ACKR4, NUMBL, INHBB, RELT, UBE2K, TRIM32, PDGFRA, AKAP6, CPD, CLDN18, RTN4RL1, CCR1, NFKBIA, CALCOCO2, FKBP1A, KIT, GREM2, SRC, GCH1, CALCA, IRAK4, CCL22, ACSL1, XBP1, IL10RB, BCL2, ACSL4, SELPLG, SEC61A1, CSF1R, THPO, KLF6, MUC2, IL2RB, GNAO1, MET, IL1RN, SMAD4, AXL, AFF3, SIRT1, TRIM21, IKBKE, RPS6KA4, PRLR, HDAC1, RPL13A, CXCL16, IRF1, TRAFD1, KLF4 | 1.802592612 | 7.51E-04 | 1.34E-05 | 1.96E-04 |
| GO:0007167~enzyme linked receptor protein signaling pathway | 94 | 6.891495601 | 1.12E-07 | NRP2, HNF1A, NDST1, SNCA, FGFRL1, RHOQ, ZEB1, FES, SLC2A8, SHKBP1, WNT1, JADE2, FAM83A, MYOCD, GATA3, GAB1, TGFA, PLCB1, PAG1, PID1, DAB2IP, RET, SOGA1, EFNB3, EFNB1, TP53, PTPRR, FGF23, SOCS7, LEF1, SOCS4, IRS1, PRKCB, PRKD1, INHBB, PRKCQ, ACVR2B, HNF4A, VEGFA, PDGFRA, PDGFRB, TGIF2, CAV3, FRK, GCNT2, HOXA13, FUT8, STRAP, ONECUT2, PPM1A, EPHA10, FKBP1A, KIT, GREM2, SRC, EPHB2, RGMB, PEG10, XBP1, RNF165, FAT4, BCL9L, GIGYF1, CSF1R, PIK3R2, BMP3, NCEH1, CAMLG, MYO1C, CREB1, MET, SMAD4, AXL, MSTN, SKI, HGF, SIRT1, TMPRSS6, CDKN1C, NOTCH2, CDH13, EPHA4, NOTCH1, DKK1, PLCG1, PRLR, NEDD4, ATXN7, TRPS1, AREG, IGFBP3, LRP4, BMP8B, BTBD11 | 1.750368959 | 8.52E-04 | 1.49E-05 | 2.22E-04 |
| GO:0051960~regulation of nervous system development | 103 | 7.551319648 | 1.14E-07 | SYT1, SRCIN1, ADCY5, JAG1, HOOK3, BRINP1, GATA3, RAB29, RARB, LRRC7, RAB21, DISC1, DAB2IP, RET, ZHX2, SIX3, DLL1, NUMBL, MAP4K4, SLITRK4, FOXG1, TRIM32, VEGFA, RELN, TGIF2, DRD2, CRTC1, ULK4, LIN28A, EPHB2, ACE, TRIM67, PVRL1, PLAG1, DIXDC1, MAP2K1, MET, SKI, HGF, NOTCH3, SEMA6A, EPHA4, NOTCH1, DLX1, HDAC1, NEDD4, RGS6, MARCKS, TCF12, KLF4, ARSB, OPRM1, GFAP, CPEB3, SYNJ1, PPARG, ZEB1, FES, MBP, XYLT1, MAPT, SEMA3E, EEF2K, ANP32A, MYC, CCDC88A, TP53, PRKCH, SPEN, MYCN, PRKD1, NCOA1, SEMA4F, CNTN2, MYRF, SEMA4C, SEMA4B, CUX1, SRGAP2, RAP1GAP, KIT, FKBP1B, FAT3, BCL11B, LRRTM2, BCL2, PER2, BCL6, NFATC4, NEDD4L, TLX2, PTPRD, NOS1, CREB1, ASIC2, SIRT1, TP73, DKK1, HEYL, NEUROD2, KDM4C, ADGRL1, LRP4 | 1.698078129 | 8.73E-04 | 1.51E-05 | 2.28E-04 |
| GO:0060322~head development | 100 | 7.331378299 | 1.26E-07 | SYT1, LDHA, SLC6A3, RORA, HOOK3, WNT1, RRAS, RARB, TWIST2, DISC1, INA, RBFOX2, DAB2IP, SATB2, SIX3, SOCS7, DLL1, GRHL2, NUMBL, CRHR1, FOXG1, PDGFRA, RELN, STMN1, PFKFB3, DRD2, HMGCS1, ULK4, NR2C2, SRC, EPHB2, TAL2, ACE, SBK1, CSF1R, DIXDC1, ABR, B4GALT2, MAP2K1, MET, AK3, NR4A2, ROGDI, SKI, GAS1, AK4, SLC6A17, KCNK3, FOXP2, NOTCH3, NOTCH1, DLX1, HDAC1, ZIC5, MARCKS, NCOR2, NRP2, NDST1, CRNKL1, SYNJ1, BCAN, ZEB1, UQCRQ, MBP, MAPT, CNTNAP2, PLCB1, CASP2, MYC, ANP32B, TP53, LEF1, PADI2, BBS1, INHBB, NCOA1, CNTN2, SEMA4C, FAIM2, SRGAP2, CCR1, TH, CTNND1, FAT4, BCL11B, BCL2, SEC16A, SLC4A7, PAFAH1B2, DCX, GNAO1, TRNP1, CREB1, AXL, TP73, WNT2B, PPP1R9B, DKK1, NEUROD2, ADGRL1 | 1.710758324 | 9.64E-04 | 1.64E-05 | 2.51E-04 |
| GO:0050790~regulation of catalytic activity | 199 | 14.58944282 | 1.57E-07 | DYNC1LI1, PPP2R5A, LHCGR, SNCA, LPAR2, ITSN1, CRY2, SERPINE1, GAB1, RECK, DAB2IP, ROCK1, RAP1GDS1, RIC8B, PPARGC1B, FARP2, CRHR1, HNF4A, SERPINF2, VEGFA, PDGFRA, PDGFRB, ATPIF1, WFDC5, NEK5, RALGPS2, RALGPS1, GNAI2, CALU, ACE, SERINC1, TNKS, HIP1, UNC119, ARHGEF33, ABR, MAP2K1, IL1RN, MSTN, HGF, OXSR1, EPHA4, NOTCH1, CSRNP3, DCP1A, PKP4, RGS6, MARCKS, KLF4, OPRM1, CPEB2, PPARG, CCNE2, ZYG11B, NMUR1, TGFA, AGAP2, MYC, GPIHBP1, ALS2CL, ARHGEF3, ANP32B, RAB4A, TP53, IL6R, ARHGEF9, MTMR12, CCND1, MAP3K15, SERPINB8, GRM7, DNAJC24, WNT9B, SERPINB2, ZER1, SRGAP2, RAP1GAP, ATG13, FKBP1A, BCCIP, KIT, FKBP1B, GCH1, CCL22, PLEKHG2, ACSL1, BCL2, CHM, MLLT1, BCL6, MTMR9, RASA4, TBC1D2, ELMOD1, NOS1, CNST, PPP1R11, PPP1R10, DGKI, TP73, DUSP4, PPP1R8, OBFC1, PLN, DGKZ, HTR2A, SRCIN1, TNFSF14, RASGEF1C, NLRC5, MYOCD, MDFIC, MS4A2, SPRED1, RNF34, NQO1, RET, STMN3, PSD3, FGF23, SOCS4, PKIA, IRS1, GRHL2, GPR55, SGSM2, AKAP6, RELN, NGB, CAV3, SNX9, FZR1, DRD2, SIPA1, SRC, OAZ2, SLX4, PPP1R16B, GMIP, LMTK3, AXIN2, TBC1D30, FGD6, FGD3, CSF1R, ARHGDIB, MET, NR4A2, ARPP19, DOCK9, DOCK8, DOCK3, CORO1C, HSPBP1, CDKN1C, TBC1D25, UACA, GTF2F1, DNAJB1, PRKAG1, PIP5K1A, PDCD4, ARHGAP1, DLG3, LTF, PLCB1, IRAK2, CCDC88A, NOS1AP, CYCS, RIMBP2, LEF1, PRKCE, ARHGAP26, PRKD1, PRKCQ, ACVR2B, PSME1, PSME3, CALCR, APH1A, RTN4RL1, CYTH4, KITLG, CALCA, RASGRP4, ARHGAP44, PIK3R2, GNAO1, FETUB, RCAN1, TMBIM1, SIRT1, RALGDS, PPP1R9B, PRLR, SMPD1, EVI5L, IGFBP3 | 1.418921599 | 0.001194311 | 1.99E-05 | 3.11E-04 |
| GO:0050804~modulation of synaptic transmission | 51 | 3.739002933 | 1.87E-07 | SYT1, GFAP, JPH3, KCNC3, SLC6A1, CPEB3, SNCA, BCAN, CALB1, S1PR2, MAPT, LGI1, STX1A, CNTNAP4, PRKCE, CRHR1, CNTN2, PLA2G6, RELN, VAMP2, NETO2, RAB3B, CPLX2, GNAI2, DRD2, CRTC1, CTNND2, KIT, RIMS4, NETO1, RIMS3, EPHB2, SHISA7, LRRTM2, TOR1A, SYN2, PER2, GRID2IP, NFATC4, NOS1, NCDN, CREB1, MET, DGKI, CACNA2D2, PPP1R9B, DKK1, NEUROD2, HTR2C, NAT8L, HTR2A | 2.204587044 | 0.00142791 | 2.34E-05 | 3.72E-04 |
| GO:0043085~positive regulation of catalytic activity | 132 | 9.677419355 | 1.88E-07 | SRCIN1, SNCA, LHCGR, LPAR2, RASGEF1C, ITSN1, MDFIC, GAB1, MS4A2, RET, DAB2IP, PSD3, RAP1GDS1, FGF23, RIC8B, IRS1, GRHL2, PPARGC1B, FARP2, CRHR1, GPR55, HNF4A, SGSM2, VEGFA, PDGFRA, PDGFRB, AKAP6, RELN, NGB, NEK5, SNX9, FZR1, RALGPS2, RALGPS1, GNAI2, DRD2, SIPA1, SRC, SLX4, ACE, GMIP, SERINC1, TNKS, TBC1D30, AXIN2, FGD6, FGD3, CSF1R, HIP1, UNC119, ARHGEF33, ABR, MAP2K1, MET, IL1RN, NR4A2, DOCK9, OXSR1, DOCK8, HGF, DOCK3, CORO1C, TBC1D25, EPHA4, UACA, DCP1A, GTF2F1, PKP4, RGS6, MARCKS, DNAJB1, KLF4, PPARG, PIP5K1A, NMUR1, ARHGAP1, LTF, TGFA, PLCB1, AGAP2, GPIHBP1, MYC, ALS2CL, IRAK2, ARHGEF3, CCDC88A, ANP32B, RAB4A, CYCS, IL6R, ARHGEF9, PRKCE, ARHGAP26, PRKD1, PRKCQ, ACVR2B, CCND1, MAP3K15, PSME1, DNAJC24, WNT9B, PSME3, SRGAP2, CALCR, RAP1GAP, APH1A, ATG13, CYTH4, KITLG, KIT, GCH1, CALCA, PLEKHG2, CCL22, ACSL1, RASGRP4, ARHGAP44, BCL2, CHM, RASA4, ELMOD1, TBC1D2, NOS1, GNAO1, SIRT1, RALGDS, TP73, PRLR, OBFC1, EVI5L, IGFBP3, HTR2A | 1.565071542 | 0.001431519 | 2.31E-05 | 3.73E-04 |
| GO:0051240~positive regulation of multicellular organismal process | 158 | 11.58357771 | 2.08E-07 | SYT1, PDLIM7, PGF, VTCN1, SLC6A3, RORA, JAG1, AMOTL1, PNP, WNT1, BRINP1, PTGIS, ATG5, MYOCD, GATA3, PDE4B, SERPINE1, RRAS, RARB, DISC1, RAB21, LRRC7, RET, DAB2IP, PTPRM, GBP5, DLL1, PPARGC1B, NUMBL, SLITRK4, SERPINF2, TRIM32, FOXG1, VEGFA, PDGFRB, AKAP6, TGIF2, RELN, ATXN1L, ASB4, GLP1R, SUCO, GCNT2, DRD2, CRTC1, DAG1, SNX4, LIN28A, SRC, NR2C2, EPHB2, PPP1R16B, ACE, TRIM67, ECE1, OVOL2, AXIN2, TMEM79, CSF1R, ASXL2, PLAG1, DIXDC1, ZBTB46, MAP2K1, MET, SMAD4, WHSC1, HGF, FOXP3, CACNA2D2, FOXP1, FOXP2, NOTCH2, EPHA4, SEMA6A, NOTCH1, HDAC1, PLCG1, RGS6, MARCKS, TCF12, ADRA1D, BMP8B, ARSB, OPRM1, GFAP, CPEB3, PPARG, SYNJ1, ZEB1, FES, LGR4, TMEM173, HPSE, TKFC, MAPT, EEF2K, LTF, MKX, PLCB1, AGAP2, MYC, GPIHBP1, PRKCH, LEF1, CFTR, IL6R, SPEN, PRKCE, PRKCB, PRKD1, INHBB, PRKCQ, ACVR2B, NCOA1, CCND1, ADK, CLIC5, MYRF, PLA2G6, CUX1, UNG, CCR1, KITLG, KIT, FKBP1B, CALCA, XBP1, BCL2, LRRTM2, BCL6, NFATC4, BCL9L, INPP5D, NEDD4L, THPO, PTPRD, NOS1, CREB1, ASIC2, AXL, LMNA, TMBIM1, SNAI1, SIRT1, TP73, WNT2B, DKK1, MAPK13, HEYL, IRF1, NEUROD2, KDM4C, ADGRL1, IRF4, HTR2C, TOB2, HTR2A | 1.491127636 | 0.001589432 | 2.52E-05 | 4.14E-04 |
| GO:0034762~regulation of transmembrane transport | 56 | 4.105571848 | 2.18E-07 | CTTNBP2NL, CALHM1, OPRM1, JPH3, KCNC3, TMEM109, KCNQ3, ANK2, ANK3, PDE4B, SERPINE1, SCN2B, NOS1AP, CACNG4, CFTR, CACNG2, CRHR1, CLIC5, PDGFRB, PLA2G6, AKAP6, KCNH7, STC1, RELN, VAMP2, NETO2, CAV3, FXYD2, SCN1A, DRD2, KCNA2, CACNB1, HK1, FKBP1A, CACNB3, FKBP1B, TPCN2, OAZ2, ACE, KCNS2, BCL2, NEDD4L, SLC31A2, HCN3, NOS1, ASIC2, ATP1A2, OXSR1, CACNA2D2, PLCG1, KCNJ8, NEDD4, PLN, GRK6, CACNA1E, SCN4A | 2.101713017 | 0.001664416 | 2.60E-05 | 4.34E-04 |
| GO:0051093~negative regulation of developmental process | 109 | 7.991202346 | 2.35E-07 | ADCY5, RORA, JAG1, CXCL10, HOOK3, S1PR3, WNT1, MYOCD, GATA3, RAB29, SERPINE1, WDR77, MED27, RARB, TWIST2, FNDC3B, DAB2IP, PTPRM, ROCK1, ZHX2, SIX3, FGF23, DLL1, GRHL2, MAP4K4, GPR55, FOXG1, VEGFA, CAV3, FZR1, STRAP, NFKBIA, SOX4, LIN28A, EPHB2, OVOL2, TBC1D30, AXIN2, ARHGDIB, DIXDC1, ZBTB46, ABR, MSTN, SKI, FOXP3, TRIM62, FOXP1, CORO1C, NOTCH3, SEMA6A, EPHA4, NOTCH1, DLX1, HDAC1, AREG, NCOR2, KLF4, GFAP, PPARG, ZEB1, MBP, XYLT1, WWC3, SEMA3E, RTF1, ANP32A, MKX, MYC, ANP32B, TP53, LEF1, CDK6, VAT1, MYCN, CCND1, SEMA4F, CNTN2, WNT9B, SEMA4C, SEMA4B, JMJD1C, SRGAP2, CLDN18, RAP1GAP, CCR1, KIT, CALCA, FAT3, XBP1, BCL2, BCL6, NFATC4, BCL9L, INPP5D, TLX2, LMNA, RCAN1, SIRT1, COL5A2, MARCH5, SNAI1, TP73, DKK1, PHF19, IRF1, NEUROD2, EVI5L, LRP4, TOB2 | 1.643980125 | 0.001788111 | 2.75E-05 | 4.66E-04 |
| GO:0009605~response to external stimulus | 226 | 16.56891496 | 2.41E-07 | LDHA, VTCN1, SNCA, RNF216, RORA, CD47, CRY2, SERPINE1, DAB2IP, PTPRM, GBP5, EFNB3, EFNB1, SERPINF2, FOXG1, VEGFA, PDGFRA, PDGFRB, STC1, ATPIF1, ALDOA, SCN1A, CRTC1, HMGCS1, DAG1, NFKBIA, RIMS3, EPHB2, ALCAM, ACE, PVRL1, STX17, CDA, ABR, MAP2K1, IL1RN, SMAD4, MSTN, HGF, GAS1, USF1, KCNK3, FAM131B, NOTCH2, EPHA4, SEMA6A, NOTCH1, KCNJ8, KLF4, BMP8B, NRP2, OPRM1, ARSB, CRNKL1, PPARG, ZEB1, XYLT1, TKFC, MAPT, SEMA3E, GPX3, GUCY1A3, MYC, HYAL3, ZDHHC8, TP53, IL6R, FOSB, ACKR4, ZCCHC17, INHBB, CCND1, ANKRD23, SEMA4F, CLIC5, SEMA4C, WNT9B, PLA2G6, SEMA4B, MAP3K14, CLOCK, SLC27A4, PARVA, CCR1, TH, ATG13, HK1, KIT, FAM46A, FKBP1B, GCH1, TPCN2, CCL22, ACSL1, BEST1, XBP1, BCL2, BCL11B, BCL6, ACSL4, NOS1, SWAP70, AXL, LMNA, STAB2, ATP1A2, TMPRSS6, CDH13, HNRNPUL1, MEX3C, DPYD, HTR2C, TP53INP2, TP53INP1, HTR2A, OPN4, RNASEL, SLC6A1, SLC6A3, TNFSF14, CXCL11, CXCL10, NLRC5, GP5, SLC16A1, PTGIS, ATG5, MYOCD, UNC5A, ANK3, GATA3, PDE4B, LGI1, NQO1, MTUS1, SATB1, PDXK, ATG9A, FGF23, THBD, UCN2, RELT, LYST, RELN, VAMP2, STMN1, CAV3, DRD2, SIPA1, SNX4, RRAGD, NR2C2, SRC, CSMD1, ECE1, GMIP, RNF165, CSF1R, MUC2, LPO, MET, NR4A2, AK3, WIPI2, FOXP3, FOXP1, FOXP2, TBC1D25, IKBKE, UACA, UCP3, HDAC1, SLC7A2, CXCL16, GTF2F1, CXCR1, PEX3, PIP5K1A, FES, TMEM173, HPSE, LTF, CNTNAP2, ABHD12, CASP2, FOSL1, IRAK2, TBL1XR1, NRXN2, ADIPOR2, CHST3, PADI2, LEF1, CDK6, DAPK2, PRKCE, PRKCB, PRKD1, PRKCQ, NCOA1, XPC, ATG4B, CNTN2, TREM1, ABLIM1, CALCOCO2, CALCA, CALCB, RASGRP4, PAFAH1B2, THPO, DNM1L, SLC12A2, MAP1A, CREB1, ASIC2, DPYSL4, SIRT6, RCAN1, FUCA2, SIRT1, WNT2B, RDH11, RPL13A, IRF1, IRF4 | 1.373431141 | 0.001838004 | 2.79E-05 | 4.79E-04 |
| GO:0030030~cell projection organization | 142 | 10.41055718 | 2.50E-07 | SYT1, SRCIN1, ADCY5, VCL, S1PR2, WNT1, GP5, ATG5, UNC5A, ANK3, GATA3, RAB29, TBC1D13, LRRC55, LGI1, DISC1, RAB21, LRRC7, RBFOX2, DBNL, RET, DAB2IP, PTPRM, STMN3, ROCK1, EFNB3, EFNB1, MYH9, TBC1D22A, NUMBL, MAP4K4, SLITRK4, HNF4A, MCIDAS, TRIM32, FOXG1, VEGFA, RELN, NGB, STMN1, DRD2, CRTC1, ONECUT2, DAG1, ULK4, NRN1, SRC, CALU, EPHB2, ALCAM, TRIM67, PVRL1, RNF165, PVRL2, TBC1D30, FGD3, CSF1R, DIXDC1, MAP2K1, PODXL, MET, NR4A2, SMAD4, GAS1, HGF, FOXP1, CORO1C, EPHA4, SEMA6A, NOTCH1, NEDD4, MARCKS, AREG, KLF4, NRP2, ARSB, LIMA1, GFAP, E2F5, CPEB3, WASF1, RHOQ, PIP5K1A, FES, MBP, SPRY3, XYLT1, MAPT, SEMA3E, CAMSAP1, ANP32A, EEF2K, CNTNAP2, CNTNAP1, CCDC88A, NOS1AP, KIF17, CHST3, COL25A1, BBS1, PRKD1, PRKCQ, SEMA4F, CLIC5, CNTN2, SEMA4C, SEMA4B, CUX1, MAP3K14, SRGAP2, PARVA, ABLIM1, RAP1GAP, ABLIM3, CTNND2, KIT, FKBP1B, NPTX1, FAT3, FAT4, BCL2, BCL11B, TOR1A, NFATC4, NEDD4L, ACSL4, DCX, TLX2, TBC1D2, PTPRD, GNAO1, NCDN, CREB1, DPYSL4, SIRT1, RAPH1, ATMIN, CDH13, PPP1R9B, GFRA1, EVI5L, LRP4 | 1.526614375 | 0.001906621 | 2.85E-05 | 4.97E-04 |
| GO:0032409~regulation of transporter activity | 36 | 2.639296188 | 2.76E-07 | CAV3, OPRM1, CTTNBP2NL, NETO2, FXYD2, JPH3, DRD2, SNCA, CACNB1, HK1, CACNB3, FKBP1A, FKBP1B, KCNS2, ANK2, ANK3, BCL2, PDE4B, NEDD4L, SCN2B, NOS1AP, ASIC2, CACNG4, CFTR, CACNG2, ATP1A2, OXSR1, CRHR1, PLCG1, NEDD4, PLN, GRK6, PLA2G6, AKAP6, RELN, VAMP2 | 2.614380871 | 0.002105909 | 3.10E-05 | 5.49E-04 |
| GO:0051649~establishment of localization in cell | 216 | 15.83577713 | 3.25E-07 | SYT1, CHMP3, VTCN1, ADCY5, SNCA, CHMP7, LHCGR, VPS52, SYT9, ITSN1, SIDT2, HOOK3, KIF13A, CRY2, RAB29, VPS4A, CREB3L1, RAB21, DAB2IP, GBP5, MYH9, ERGIC1, PCLO, VTI1A, CRHR1, HNF4A, TBRG1, SURF4, ATPIF1, SUPT6H, CACNB1, DAG1, NFKBIA, GPR143, MYT1, RIMS4, RIMS3, STX17, PVRL2, GOLPH3L, ABR, MAP2K1, IL1RN, PTPN14, SMAD4, CDC23, GAS1, NOTCH2, NOTCH1, MARCKS, OPRM1, CREBRF, LZTS2, JPH3, HNF1A, AP1B1, PPARG, SYNJ1, AP3S2, AP1S2, NMUR1, MAPT, ANP32A, GPIHBP1, STX1A, ANP32B, RAB4A, ZDHHC8, TP53, CFTR, MYADM, INHBB, MYRIP, GRM7, PLA2G6, CLOCK, RAB3B, CCR1, TH, ATG13, BET1, FKBP1A, KLC2, KIT, FKBP1B, TPCN2, RAB43, XBP1, BCL2, PER2, CHM, BCL6, LYPLAL1, ELMOD1, NOS1, CNST, AXL, LMNA, LRRC46, DGKI, COG3, COG6, PLN, SVIP, ADGRL1, HTR2C, HTR2A, NCBP2, KCNC3, SRCIN1, XPO5, XPO4, TNFSF14, ILDR2, ILDR1, CXCL11, PNP, CXCL10, SLC16A1, ATG5, ANK2, ANK3, MDFIC, GATA3, MS4A2, LGI3, SAR1A, TMEM201, SIX3, FGF23, PKIA, IRS1, CDKL2, MAP4K4, UCN2, SGSM2, LYST, AKAP6, VAMP2, ERC1, GLP1R, PACS1, SNX9, SYVN1, DRD2, KCNA2, COPZ1, SOX4, SNX4, LMAN1, SRC, OAZ2, AXIN2, SLC30A6, SEC61A1, TMEM79, RHOBTB3, CSF1R, MYO1C, FOXP3, FOXP1, UACA, UCP3, PLCG1, HDAC1, AKTIP, NEDD4, CACNA1E, SPCS2, DPH6, PEX3, PIP5K1A, FES, SPICE1, LGR4, TMEM173, PEX2, TRIM9, PLCB1, NRXN2, NOS1AP, SRPRA, PFKM, EDAR, PRKCE, PRKCB, BBS1, PRKD1, MFN2, PRKCQ, ACVR2B, DGAT1, ATG4B, TOM1, TREM1, CUX1, CPLX2, ABLIM3, PPM1A, PPP3R1, CALCA, MOAP1, NPTX1, ARHGAP44, TOR1A, SEC16A, SYN2, PIK3R2, EHD4, SPRN, DNM1L, CREB1, MAL, HID1, SIRT1, KCNN3 | 1.380834967 | 0.002479043 | 3.60E-05 | 6.47E-04 |
| GO:0033043~regulation of organelle organization | 129 | 9.457478006 | 3.35E-07 | SYT1, DYNC1LI1, CHMP3, SLC6A1, SNCA, ATG5, MYOCD, GATA3, TBC1D13, VPS4A, SAR1A, PID1, DBNL, STMN3, ROCK1, MYH9, CDKL2, TBC1D22A, UHRF2, SERPINF2, VEGFA, SURF4, PDGFRB, ATPIF1, STMN1, CLIP3, ADD2, SUPT6H, MAP6D1, CAV3, FZR1, HOXA13, DRD2, LMAN1, GPR143, NR2C2, SRC, RIMS3, SLX4, GMIP, CNOT6L, NAT10, TNKS, TBC1D30, AXIN2, BMF, CSF1R, ASXL2, DIXDC1, MAP2K1, MET, SMAD4, CDC23, MSTN, HGF, FOXP3, FAM131B, KCNK3, TBC1D25, TRPS1, TMOD2, LIMA1, CRNKL1, WASF1, DPH6, VPS37B, PEX3, FES, SPICE1, NMUR1, TRIM9, MAPT, PAX7, SEMA3E, RTF1, ANP32A, TGFA, PLCB1, MYC, CCDC88A, ZDHHC8, TP53, CHST3, PADI2, PRKCE, ZCCHC17, MYADM, VAT1, PRKD1, PRKCQ, ANKRD23, PLEKHH2, PLA2G6, TREM1, NSD1, ABLIM3, PPFIA1, ATG13, CALCOCO2, HK1, CTCFL, CALCB, MOAP1, MPV17L2, XBP1, BCL6, ELMOD1, TBC1D2, PIK3R2, SYNPO, INF2, CAPN6, NOS1, DNM1L, SWAP70, MAP1A, LRRC46, LMNA, MID1, SIRT1, MARCH5, GMFB, RPS6KA4, PHF19, OBFC1, KCNN3, MEX3C, KDM4C, EVI5L | 1.558768406 | 0.002553166 | 3.65E-05 | 6.66E-04 |
| GO:0023057~negative regulation of signaling | 123 | 9.017595308 | 3.54E-07 | SLC6A1, SNCA, RORA, S1PR2, NLRC5, WNT1, CRY2, GATA3, SERPINE1, CREB3L1, SPRED1, RNF34, PID1, DAB2IP, STMN3, STRN3, SIX3, PTPRR, FGF23, SOCS4, UBR1, IRS1, CRHR1, MAP4K4, UCN2, HNF4A, TRIM32, VEGFA, MNT, CAV3, SYVN1, PPP2R3A, GNAI2, STRAP, DRD2, ONECUT2, DAG1, NFKBIA, BDKRB2, GREM2, SRC, PEA15, PEG10, AMER1, TRIM67, OVOL2, GRID2IP, AXIN2, BMF, IL1RN, NR4A2, MSTN, SKI, GAS1, HGF, FOXP1, NOTCH1, DLX1, UACA, HDAC1, NEDD4, RGS6, GRK6, KLF4, CREBRF, OPRM1, LZTS2, PPARG, PDCD4, LGR4, TKFC, MAPT, WWC3, LTF, AGAP2, PGAP2, TP53, LEF1, PADI2, MYADM, PRKCB, LRPAP1, INHBB, MFN2, PRKCQ, CCND1, PLA2G6, PSME3, CLOCK, FAIM2, ZMYND11, RTN4RL1, PPM1A, CTNND1, FKBP1B, CALCA, XBP1, BCL2, MARVELD3, BCL6, NFATC4, BCL9L, INPP5D, RASA4, MLLT3, PTPRD, NOS1, LMNA, TMBIM1, SNAI1, SIRT1, TMPRSS6, TP73, DUSP4, DKK1, ATXN7, HEYL, IRF1, SMPD1, DGKZ, IRF4, LRP4, HTR2A | 1.576781637 | 0.002697876 | 3.80E-05 | 7.04E-04 |
| GO:0051174~regulation of phosphorus metabolic process | 164 | 12.02346041 | 3.56E-07 | SRCIN1, CTDSPL, PPP2R5A, SNCA, LHCGR, LPAR2, ITSN1, CXCL11, CXCL10, S1PR2, NLRC5, WNT1, CRY2, MYOCD, MDFIC, GAB1, RRAS, CTDSP2, SPRED1, PID1, RET, DAB2IP, ROCK1, PTPRR, FGF23, SOCS4, PKIA, IRS1, PPARGC1B, CRHR1, MAP4K4, GPR55, HNF4A, RELT, SERPINF2, MC2R, VEGFA, PDGFRA, PDGFRB, AKAP6, ATPIF1, RELN, CLIP3, CAV3, SNX9, GCNT2, GNAI2, STRAP, PFKFB3, DRD2, DAG1, BDKRB2, ULK4, SRC, PPP1R16B, ACE, ECE1, LPCAT1, IDH1, CDA, LMTK3, AXIN2, CSF1R, DIXDC1, BMP3, UNC119, MAP2K1, IL1RN, MET, ARPP19, SMAD4, MSTN, HGF, OXSR1, CORO1C, CDKN1C, EPHA4, CSRNP3, AKTIP, HDAC1, MARCKS, AREG, KLF4, BMP8B, OPRM1, CBFA2T3, PDCD4, CCNE2, RSPO1, EEF2K, LTF, DLG3, TGFA, GUCY1A3, PLCB1, AGAP2, MYC, IRAK2, TBL1XR1, CCDC88A, RIMBP2, TP53, IL6R, PRKCE, MYADM, PRKD1, INHBB, ACVR2B, CCND1, KSR2, MAP3K15, UBE2K, GRM7, WNT9B, SEMA4C, PLA2G6, KSR1, NSD1, CALCR, ZMYND11, RTN4RL1, CCR1, ATG13, CTNND1, KITLG, BCCIP, FKBP1A, KIT, FKBP1B, CALCA, CCL22, ACSL1, XBP1, BCL2, MARVELD3, MLLT1, EHD4, THPO, PIK3R2, NOS1, DNM1L, CNST, SWAP70, PDK3, PPP1R11, RCAN1, SIRT6, MID1, SIRT1, BRAT1, TP73, PPP1R9B, DUSP4, RPS6KA4, DKK1, PRLR, ATXN7, IRF1, SMPD1, IGFBP3, HTR2C, LRP4, RNF41, HTR2A | 1.465840899 | 0.002713742 | 3.77E-05 | 7.08E-04 |
| GO:0031399~regulation of protein modification process | 168 | 12.31671554 | 3.57E-07 | DYNC1LI1, SRCIN1, CTDSPL, PPP2R5A, SNCA, LPAR2, S1PR2, WNT1, CRY2, ATG5, MYOCD, MDFIC, GATA3, GAB1, RRAS, CTDSP2, SPRED1, DISC1, PID1, RET, DAB2IP, ROCK1, PTPRR, FGF23, SOCS4, PKIA, PPARGC1B, MAP4K4, GPR55, HNF4A, RELT, SERPINF2, VEGFA, PDGFRA, PDGFRB, AKAP6, RELN, CLIP3, SUPT6H, CAV3, SNX9, FZR1, GCNT2, GNAI2, STRAP, DRD2, DAG1, SOX4, BDKRB2, ULK4, SRC, PPP1R16B, AMER1, ACE, TRIM67, ECE1, LMTK3, AXIN2, CSF1R, ASXL2, DIXDC1, BMP3, UNC119, MAP2K1, IL1RN, MET, ARPP19, SMAD4, MSTN, HGF, OXSR1, FOXP3, CORO1C, HSPBP1, CDKN1C, EPHA4, CSRNP3, AKTIP, HDAC1, TRPS1, DNAJB2, MARCKS, KLF4, BMP8B, NCOR2, OPRM1, PDCD4, ZYG11B, CCNE2, RSPO1, PAX7, RTF1, ANP32A, EEF2K, LTF, DLG3, TGFA, PLCB1, AGAP2, IRAK2, ANKS1A, CCDC88A, NOS1AP, RIMBP2, TP53, IL6R, PRKCE, MYADM, PRKD1, INHBB, ACVR2B, CCND1, KSR2, MAP3K15, UBE2K, WNT9B, SEMA4C, PLA2G6, KSR1, NSD1, ZER1, ZMYND11, RTN4RL1, CCR1, ATG13, CTNND1, KITLG, CTCFL, BCCIP, FKBP1A, KIT, FKBP1B, CALCA, CCL22, ACSL1, XBP1, BCL2, MARVELD3, PER2, MLLT1, BCL6, EHD4, THPO, NOS1, CNST, SWAP70, PPP1R11, RCAN1, MID1, BRAT1, SIRT1, TRIM21, TP73, PPP1R9B, DUSP4, RPS6KA4, PHF19, DKK1, PRLR, IRF1, SMPD1, KDM4C, SVIP, IGFBP3, HTR2C, LRP4, RNF41, HTR2A | 1.456769441 | 0.002723849 | 3.74E-05 | 7.10E-04 |
| GO:0032989~cellular component morphogenesis | 139 | 10.19061584 | 4.13E-07 | SYT1, SRCIN1, FGFRL1, SIDT2, VCL, GP5, ANK2, ATG5, UNC5A, ANK3, GATA3, TBC1D13, LRRC55, LGI1, DISC1, RAB21, FNDC3B, PID1, DBNL, RBFOX2, RET, DAB2IP, PTPRM, ROCK1, EFNB3, EFNB1, MYH9, GRHL2, TBC1D22A, NUMBL, MAP4K4, SLITRK4, VSIG1, MCIDAS, FOXG1, VEGFA, PDGFRA, PDGFRB, RELN, STC1, STMN1, CAV3, ALDOA, GCNT2, HOXA13, STRAP, DRD2, TFCP2L1, PEAK1, ONECUT2, DAG1, ULK4, NRN1, SRC, EPHB2, ALCAM, OVOL2, PVRL1, RNF165, PVRL2, TBC1D30, AXIN2, CSF1R, DIXDC1, MAP2K1, MET, NR4A2, SMAD4, GAS1, HGF, TRIM62, FOXP1, CORO1C, EPHA4, SEMA6A, NOTCH1, CDC42SE2, NEDD4, TMOD2, MARCKS, NRP2, RHOJ, WASF1, RHOQ, FES, MBP, MAPT, SEMA3E, EEF2K, CNTNAP1, CAP1, FMNL3, LEF1, COL25A1, MYADM, BBS1, XIRP1, SEMA4F, CLIC5, CNTN2, WNT9B, SEMA4C, SEMA4B, JMJD1C, CUX1, MAP3K14, SRGAP2, PARVA, ABLIM1, ABLIM3, CTNND2, PPP3R1, KIT, NPTX1, FAT3, BCL2, BCL11B, PALM2, BCL6, NFATC4, BCL9L, NEDD4L, DCX, TLX2, TBC1D2, PTPRD, DNM1L, AKIP1, CREB1, AXL, DPYSL4, SNAI1, RAPH1, ATMIN, DKK1, MYPN, HEYL, EVI5L, LRP4 | 1.521388852 | 0.003143405 | 4.25E-05 | 8.20E-04 |
| GO:0019220~regulation of phosphate metabolic process | 163 | 11.95014663 | 4.74E-07 | SRCIN1, CTDSPL, PPP2R5A, SNCA, LHCGR, LPAR2, ITSN1, CXCL11, CXCL10, S1PR2, NLRC5, WNT1, CRY2, MYOCD, MDFIC, GAB1, RRAS, CTDSP2, SPRED1, PID1, RET, DAB2IP, ROCK1, PTPRR, FGF23, SOCS4, PKIA, IRS1, PPARGC1B, CRHR1, MAP4K4, GPR55, HNF4A, RELT, SERPINF2, MC2R, VEGFA, PDGFRA, PDGFRB, AKAP6, ATPIF1, RELN, CLIP3, CAV3, SNX9, GCNT2, GNAI2, STRAP, PFKFB3, DRD2, DAG1, BDKRB2, ULK4, SRC, PPP1R16B, ACE, ECE1, LPCAT1, IDH1, CDA, LMTK3, AXIN2, CSF1R, DIXDC1, BMP3, UNC119, MAP2K1, IL1RN, MET, ARPP19, SMAD4, MSTN, HGF, OXSR1, CORO1C, CDKN1C, EPHA4, CSRNP3, AKTIP, HDAC1, MARCKS, AREG, KLF4, BMP8B, OPRM1, CBFA2T3, PDCD4, CCNE2, RSPO1, EEF2K, LTF, DLG3, TGFA, GUCY1A3, PLCB1, AGAP2, MYC, IRAK2, TBL1XR1, CCDC88A, RIMBP2, TP53, IL6R, PRKCE, MYADM, PRKD1, INHBB, ACVR2B, CCND1, KSR2, MAP3K15, UBE2K, GRM7, WNT9B, SEMA4C, PLA2G6, KSR1, NSD1, CALCR, ZMYND11, RTN4RL1, CCR1, ATG13, CTNND1, KITLG, BCCIP, FKBP1A, KIT, FKBP1B, CALCA, CCL22, ACSL1, XBP1, BCL2, MARVELD3, MLLT1, EHD4, THPO, PIK3R2, NOS1, DNM1L, CNST, SWAP70, PPP1R11, RCAN1, SIRT6, MID1, SIRT1, BRAT1, TP73, PPP1R9B, DUSP4, RPS6KA4, DKK1, PRLR, ATXN7, IRF1, SMPD1, IGFBP3, HTR2C, LRP4, RNF41, HTR2A | 1.461399458 | 0.00361171 | 4.82E-05 | 9.42E-04 |
| GO:0022898~regulation of transmembrane transporter activity | 34 | 2.492668622 | 4.93E-07 | CAV3, OPRM1, NETO2, FXYD2, JPH3, DRD2, CACNB1, HK1, CACNB3, FKBP1A, FKBP1B, KCNS2, ANK2, ANK3, BCL2, PDE4B, NEDD4L, SCN2B, NOS1AP, ASIC2, CACNG4, CFTR, ATP1A2, OXSR1, CACNG2, CRHR1, PLCG1, NEDD4, PLN, GRK6, PLA2G6, AKAP6, RELN, VAMP2 | 2.640788759 | 0.003755612 | 4.95E-05 | 9.80E-04 |
| GO:0071310~cellular response to organic substance | 194 | 14.2228739 | 5.12E-07 | PGF, ADCY5, FGFRL1, LHCGR, SNCA, RORA, BRINP1, PLOD1, RAE1, SERPINE1, GAB1, CREB3L1, RARB, DAB2IP, GBP5, YY1, HNF4G, UBR1, CRHR1, HNF4A, VEGFA, PDGFRA, PDGFRB, STC1, GCNT2, HOXA13, HMGCS1, NFKBIA, ACE, BMP3, KLF6, ESRRA, IL1RN, SMAD4, MSTN, SKI, HGF, GAS1, USF1, NOTCH2, NOTCH1, NR1I2, MARCKS, BMP8B, KLF4, OPRM1, CREBRF, NRP2, CPEB2, CRNKL1, CPEB3, GLRA3, PPARG, NAP1L1, ZEB1, EEF2K, MYC, HYAL3, TP53, CFTR, IL6R, FOSB, ACKR4, INHBB, CCND1, PLA2G6, CLDN18, RAP1GAP, FUT8, CCR1, TH, FKBP1A, KIT, FKBP1B, CCL22, ACSL1, RPL32, IL10RB, FAT4, XBP1, BCL2, BCL9L, AGO4, SHMT1, NOS1, PDK3, AXL, ATP1A2, TMPRSS6, CDH13, DKK1, RPS6KA4, LRP4, TP53INP1, RNASEL, CBX3, CXCL11, CALB1, CXCL10, SLC2A8, NLRC5, WNT1, SLC16A1, PTGIS, MYOCD, GATA3, PDE4B, MTUS1, PID1, RET, SATB2, SOGA1, FGF23, SOCS7, DLL1, SOCS4, IRS1, NUMBL, MAP4K4, UCN2, RELT, TRIM32, AKAP6, RELN, CPD, VAMP2, GLP1R, CAV3, SYVN1, STRAP, ONECUT2, SOX4, PAQR7, PAQR8, RRAGD, LIN28A, GREM2, NR2C2, SRC, IRAK4, PEG10, GPR22, RNF165, AXIN2, HCN3, CSF1R, MUC2, MYO1C, MET, NR4A2, CDKN1C, UCP3, PLCG1, HDAC1, NEDD4, CXCL16, NDST1, IL21R, CXCR1, RHOQ, TMEM173, CDIP1, LTF, PLCB1, IRAK2, ADIPOR2, PADI2, LEF1, PRKCE, PRKCB, PRKD1, PRKCQ, NCOA1, ACVR2B, UBE2K, RTN4RL1, PPM1A, CALCA, NPTX1, RGMB, TOR1A, SELPLG, THPO, PIK3R2, EHD4, IL2RB, CREB1, COL5A2, SIRT1, PPP1R9B, PRLR, RPL13A, HEYL, IRF1 | 1.403946988 | 0.003901367 | 5.08E-05 | 0.001018216 |
| GO:0034765~regulation of ion transmembrane transport | 53 | 3.885630499 | 5.57E-07 | CALHM1, OPRM1, JPH3, KCNC3, TMEM109, KCNQ3, ANK2, ANK3, PDE4B, SERPINE1, SCN2B, NOS1AP, CACNG4, CFTR, CACNG2, CRHR1, CLIC5, PLA2G6, AKAP6, KCNH7, PDGFRB, RELN, STC1, VAMP2, NETO2, CAV3, FXYD2, SCN1A, DRD2, KCNA2, CACNB1, HK1, CACNB3, FKBP1A, FKBP1B, TPCN2, ACE, KCNS2, NEDD4L, SLC31A2, HCN3, NOS1, ASIC2, ATP1A2, OXSR1, CACNA2D2, PLCG1, KCNJ8, NEDD4, PLN, GRK6, CACNA1E, SCN4A | 2.091820443 | 0.004239874 | 5.45E-05 | 0.001106751 |
| GO:0010648~negative regulation of cell communication | 123 | 9.017595308 | 6.68E-07 | SLC6A1, SNCA, RORA, S1PR2, NLRC5, WNT1, CRY2, GATA3, SERPINE1, CREB3L1, SPRED1, RNF34, PID1, DAB2IP, STMN3, STRN3, SIX3, PTPRR, FGF23, SOCS4, UBR1, IRS1, CRHR1, MAP4K4, UCN2, HNF4A, TRIM32, VEGFA, MNT, CAV3, SYVN1, PPP2R3A, GNAI2, STRAP, DRD2, ONECUT2, DAG1, NFKBIA, BDKRB2, GREM2, SRC, PEA15, PEG10, AMER1, TRIM67, OVOL2, GRID2IP, AXIN2, BMF, IL1RN, NR4A2, MSTN, SKI, GAS1, HGF, FOXP1, NOTCH1, DLX1, UACA, HDAC1, NEDD4, RGS6, GRK6, KLF4, CREBRF, OPRM1, LZTS2, PPARG, PDCD4, LGR4, TKFC, MAPT, WWC3, LTF, AGAP2, PGAP2, TP53, LEF1, PADI2, MYADM, PRKCB, LRPAP1, INHBB, MFN2, PRKCQ, CCND1, PLA2G6, PSME3, CLOCK, FAIM2, ZMYND11, RTN4RL1, PPM1A, CTNND1, FKBP1B, CALCA, XBP1, BCL2, MARVELD3, BCL6, NFATC4, BCL9L, INPP5D, RASA4, MLLT3, PTPRD, NOS1, LMNA, TMBIM1, SNAI1, SIRT1, TMPRSS6, TP73, DUSP4, DKK1, ATXN7, HEYL, IRF1, SMPD1, DGKZ, IRF4, LRP4, HTR2A | 1.557535828 | 0.005083964 | 6.45E-05 | 0.001327648 |
| GO:0016310~phosphorylation | 196 | 14.36950147 | 6.98E-07 | FASTKD2, PPP2R5A, SNCA, LPAR2, ITSN1, S1PR2, GAB1, DAB2IP, ROCK1, PTPRR, PPARGC1B, HNF4A, SERPINF2, VEGFA, PDGFRA, PDGFRB, NEK5, ALDOA, GCNT2, GNAI2, PFKFB3, DAG1, BDKRB2, EPHB2, ACE, TNKS, BMP3, UNC119, MAP2K1, IL1RN, SMAD4, MSTN, HGF, OXSR1, RPS6KL1, EPHA4, GRK6, MARCKS, AREG, FUK, BMP8B, KLF4, OPRM1, CCNE2, TKFC, EEF2K, TGFA, AGAP2, MYC, PHKG2, TP53, IL6R, PI4KB, MYADM, MAST3, INHBB, CCND1, MAP3K15, KSR2, ADK, WNT9B, SEMA4C, PLA2G6, KSR1, MAP3K14, NSD1, ZMYND11, CCR1, ATG13, HK1, FKBP1A, BCCIP, KIT, CCL22, ACSL1, DGKB, SPEG, XBP1, BCL2, MLLT1, GK5, NOS1, PDK3, AXL, DGKI, MID1, BRAT1, TP73, DUSP4, DKK1, RPS6KA4, MAPK13, SDHC, DGKZ, HTR2C, LRP4, HTR2A, RNASEL, SRCIN1, PSTK, CTDSPL, STK35, NLRC5, WNT1, MYOCD, MDFIC, RRAS, CTDSP2, SPRED1, PID1, RET, PDXK, CSNK1G1, FGF23, SOCS4, PKIA, IRS1, CDKL2, MAP4K4, GPR55, RELT, AKAP6, RELN, CLIP3, ERC1, CAV3, SNX9, STRAP, DRD2, PEAK1, EPHA10, ULK4, NR2C2, SRC, IRAK4, ECE1, SBK1, AXIN2, STK38L, CSF1R, DIXDC1, MET, AK3, AK4, AK9, CORO1C, CDKN1C, IKBKE, HDAC1, AKTIP, NDST1, PRKAG1, PIP5K1A, CBFA2T3, FES, PDCD4, RSPO1, LTF, PLCB1, IRAK2, CCDC88A, CYCS, NDUFC2, PRKCH, CDK6, PFKM, DAPK2, PRKCE, TBCK, PRKCB, PRKD1, PRKCQ, ACVR2B, UBE2K, FRK, SNX15, RTN4RL1, KITLG, CTNND1, CALCA, MARVELD3, UCK2, DCX, THPO, PIK3R2, EHD4, CREB1, SIRT6, SIRT1, PPP1R9B, PRLR, ATXN7, IRF1, SMPD1, IGFBP3, RNF41 | 1.394108855 | 0.005309829 | 6.65E-05 | 0.001386788 |
| GO:0007268~synaptic transmission | 68 | 4.985337243 | 6.98E-07 | OPRM1, SYT1, GFAP, JPH3, KCNC3, SLC6A1, CPEB3, SLC6A3, GLRA3, SNCA, SYNJ1, BCAN, CALB1, S1PR2, TRIM9, MAPT, DLG3, LGI1, STX1A, NOS1AP, CNTNAP4, NRXN2, PRKCE, PCLO, CRHR1, GRM7, CNTN2, PLA2G6, RELN, VAMP2, NETO2, RAB3B, CPLX2, GNAI2, DRD2, CRTC1, TH, CACNB1, CTNND2, CACNB3, KIT, RIMS4, EPHB2, NETO1, RIMS3, SHISA7, XBP1, LRRTM2, TOR1A, SYN2, PER2, GRID2IP, NFATC4, NOS1, NCDN, GABRA3, CREB1, MET, DGKI, CACNA2D2, DKK1, PNOC, NEUROD2, TMOD2, ADGRL1, HTR2C, NAT8L, HTR2A | 1.877671095 | 0.005311632 | 6.57E-05 | 0.00138726 |
| GO:1902531~regulation of intracellular signal transduction | 149 | 10.92375367 | 7.73E-07 | SLC44A2, LHCGR, TNFSF14, LPAR2, RORA, ITSN1, CXCL11, CXCL10, MDFIC, GATA3, GAB1, RRAS, CREB3L1, SPRED1, RNF34, RET, DAB2IP, STMN3, PTPRR, PSD3, FGF23, RAD9A, SOCS4, UBR1, FARP2, MAP4K4, GPR55, HNF4A, RELT, SERPINF2, TRIM32, VEGFA, PDGFRA, PDGFRB, AKAP6, RELN, CAV3, RALGPS2, SYVN1, GCNT2, RALGPS1, GNAI2, DRD2, SIPA1, DAG1, NFKBIA, BDKRB2, ULK4, GPR143, SRC, IRAK4, PPP1R16B, TRIM67, ECE1, FGD6, FGD3, CSF1R, DIXDC1, BMP3, ARHGEF33, ABR, MAP2K1, IL1RN, MET, ARPP19, MSTN, HGF, TRIM62, NOTCH2, EPHA4, IKBKE, EI24, NOTCH1, UACA, HDAC1, NEDD4, KLF4, BMP8B, OPRM1, SHOC2, PDCD4, TKFC, HPSE, WWC3, LTF, TGFA, PLCB1, AGAP2, ALS2CL, IRAK2, PGAP2, ARHGEF3, TP53, IL6R, ARHGEF9, EDAR, DAPK2, PRKCE, MYADM, ARHGAP26, PRKCB, PRKD1, INHBB, MFN2, PRKCQ, ZDHHC17, KSR2, MAP3K15, SEMA4C, PLA2G6, KSR1, MAP3K14, ZMYND11, RTN4RL1, RAP1GAP, CCR1, CYTH4, PPM1A, KITLG, FKBP1A, KIT, PLEKHG2, CCL22, XBP1, RASGRP4, BCL2, MARVELD3, BCL6, RASA4, THPO, PTPRD, DNM1L, AXL, RCAN1, DGKI, MID1, SNAI1, SIRT1, TP73, CDH13, DUSP4, IRF1, SMPD1, NEUROD2, DGKZ, IGFBP3, HTR2C, RNF41, HTR2A | 1.479238816 | 0.005883322 | 7.20E-05 | 0.001537011 |
| GO:0032412~regulation of ion transmembrane transporter activity | 33 | 2.419354839 | 7.96E-07 | CAV3, OPRM1, NETO2, FXYD2, JPH3, DRD2, CACNB1, HK1, CACNB3, FKBP1A, FKBP1B, KCNS2, ANK2, ANK3, PDE4B, NEDD4L, SCN2B, NOS1AP, ASIC2, CACNG4, CFTR, ATP1A2, OXSR1, CACNG2, CRHR1, PLCG1, NEDD4, PLN, GRK6, PLA2G6, AKAP6, RELN, VAMP2 | 2.633533844 | 0.006054591 | 7.32E-05 | 0.00158189 |
| GO:0051270~regulation of cellular component movement | 93 | 6.818181818 | 8.32E-07 | ARSB, SRCIN1, TNFSF14, FES, CXCL11, AMOTL1, CXCL10, VCL, ANK2, MAPT, GATA3, PDE4B, SERPINE1, SEMA3E, GAB1, RRAS, PLCB1, MYC, MTUS1, RECK, DAB2IP, RET, PTPRM, SCN2B, MTA2, TP53, PTPRR, CHST3, PADI2, LEF1, DAPK2, PRKCE, MYADM, IRS1, BBS1, PRKD1, SEMA4F, TRIM32, VEGFA, PDGFRA, SEMA4C, PDGFRB, AKAP6, SEMA4B, STC1, RELN, STMN1, SRGAP2, CAV3, GCNT2, PPP2R3A, STRAP, DRD2, CCR1, ONECUT2, DAG1, KITLG, ULK4, KIT, SRC, ACE, BCL2, MARVELD3, BCL6, NEDD4L, ARHGDIB, CSF1R, MUC2, PLET1, ABR, MYO1C, MAP2K1, SWAP70, PODXL, MET, IL1RN, LMNA, HACE1, HGF, ATP1A2, SNAI1, FOXP1, CORO1C, CDH13, SEMA6A, NOTCH1, PLCG1, PLN, CXCL16, IGFBP3, KLF4, RNF41, TP53INP1 | 1.680054042 | 0.00632722 | 7.56E-05 | 0.001653346 |
| GO:0048812~neuron projection morphogenesis | 71 | 5.205278592 | 1.02E-06 | NRP2, SYT1, SRCIN1, MBP, VCL, GP5, ANK3, UNC5A, MAPT, GATA3, SEMA3E, EEF2K, CNTNAP1, LRRC55, LGI1, DISC1, RAB21, RBFOX2, DBNL, DAB2IP, PTPRM, EFNB3, EFNB1, COL25A1, NUMBL, MAP4K4, SLITRK4, SEMA4F, FOXG1, VEGFA, CNTN2, SEMA4C, SEMA4B, RELN, STMN1, CUX1, MAP3K14, SRGAP2, ABLIM1, DRD2, DAG1, CTNND2, NRN1, EPHB2, ALCAM, NPTX1, PVRL1, RNF165, BCL2, BCL11B, NFATC4, NEDD4L, DCX, TLX2, CSF1R, DIXDC1, PTPRD, MAP2K1, CREB1, MET, NR4A2, SMAD4, DPYSL4, GAS1, RAPH1, FOXP1, SEMA6A, EPHA4, NOTCH1, MARCKS, LRP4 | 1.828418458 | 0.007786036 | 9.20E-05 | 0.002036034 |
| GO:0018193~peptidyl-amino acid modification | 112 | 8.211143695 | 1.03E-06 | TUSC3, SRCIN1, SNCA, S1PR2, ATG5, BRPF3, MYOCD, PLOD1, GATA3, SPRED1, RET, CSNK1G1, SOCS4, NKTR, OGFOD1, EGFLAM, HNF4A, ASPHD2, VEGFA, PDGFRA, PDGFRB, RELN, CLIP3, SUPT6H, MAP6D1, SYVN1, PPIL2, PEAK1, SOX4, BDKRB2, SRC, EPHB2, ACE, SBK1, NAA50, TNKS, STK38L, CSF1R, ASXL2, UNC119, MAP2K1, TAF5, MET, SMAD4, WHSC1, OXSR1, HGF, FOXP3, NMT2, MSL2, PPIE, EPHA4, HDAC1, HNF1A, ARID4A, DPH6, ARID4B, FES, JADE2, PRMT2, RTF1, ANP32A, TGFA, KMT5B, NOS1AP, TP53, LEF1, PADI2, PRKCH, IL6R, FAM76A, PRKCE, PADI1, PRKCB, PRKD1, MAST3, PRKCQ, SENP3, NCOA1, UBE2K, FKBP11, NSD1, CLOCK, FKBP9, KMT2D, FRK, ING3, RAB3B, FUT8, KITLG, HK1, CTNND1, FKBP1A, KIT, FKBP1B, BCL2, DCX, BAZ2A, EHD4, NOS1, PDK3, AXL, SIRT1, DKK1, PHF19, RPS6KA4, MAPK13, IRF1, KDM4C, IRF4, LRP4, HTR2A | 1.583958983 | 0.007823384 | 9.13E-05 | 0.002045839 |
| GO:0016192~vesicle-mediated transport | 121 | 8.870967742 | 1.12E-06 | SYT1, CHMP3, SRCIN1, SNCA, VPS52, SYT9, ILDR1, ITSN1, HOOK3, KIF13A, CD47, ATG5, ANK3, RAB29, SERPINE1, MS4A2, VPS4A, LGI3, SAR1A, RAB21, DBNL, DLL1, MYH9, ERGIC1, PCLO, VTI1A, CRHR1, SGSM2, LYST, VEGFA, SURF4, VAMP2, CLIP3, ERC1, CAV3, PACS1, SNX9, DRD2, COPZ1, SNX4, LMAN1, RIMS4, SRC, RIMS3, STX17, GOLPH3L, FCHO2, TMEM79, SLC30A6, RHOBTB3, HIP1, UNC119, ABR, MAP2K1, GAS1, NOTCH1, AKTIP, NEDD4, CDC42SE2, CXCL16, AP1B1, SYNJ1, PPARG, AP3S2, VPS37B, CXCR1, FES, AP1S2, RSPO1, TRIM9, EEF2K, NEURL1B, CAP1, PLCB1, GPIHBP1, STX1A, KIF17, RAB4A, CFTR, ACKR4, ELMO1, BBS1, PRKD1, CNTN2, PLA2G6, CUX1, EPN2, CALCR, CPLX2, RAB3B, RAP1GAP, CCR1, TH, BET1, EEA1, KIT, CALCA, RAB43, ARHGAP44, LRRTM2, SEC16A, TOR1A, NEDD4L, LYPLAL1, GPR107, EHD4, DNM1L, CNST, AXL, STAB2, SFT2D3, TMPRSS4, COG3, PREB, CDH13, DKK1, COG6, MCFD2, ADGRL1, SNX30, LRP4 | 1.54840962 | 0.008535948 | 9.85E-05 | 0.002232976 |
| GO:0006935~chemotaxis | 63 | 4.618768328 | 1.13E-06 | NRP2, TNFSF14, CXCR1, PIP5K1A, FES, CXCL11, CXCL10, UNC5A, ANK3, GATA3, PDE4B, SERPINE1, SEMA3E, LGI1, MTUS1, PTPRM, EFNB3, EFNB1, LEF1, PADI2, ACKR4, DAPK2, PRKD1, PRKCQ, SEMA4F, LYST, FOXG1, VEGFA, CNTN2, SEMA4C, PDGFRA, PDGFRB, SEMA4B, PLA2G6, RELN, TREM1, PARVA, ABLIM1, CCR1, DAG1, KIT, EPHB2, ALCAM, CALCA, CCL22, PVRL1, RNF165, BCL11B, CSF1R, SWAP70, CREB1, MET, SMAD4, DPYSL4, HGF, GAS1, FOXP1, SEMA6A, EPHA4, CDH13, NOTCH1, RPL13A, CXCL16 | 1.906319385 | 0.008610147 | 9.83E-05 | 0.00225247 |
| GO:0051051~negative regulation of transport | 65 | 4.765395894 | 1.28E-06 | CTTNBP2NL, OPRM1, SRCIN1, SNCA, PPARG, LGR4, CRY2, ATG5, MDFIC, ANK3, TRIM9, MAPT, SAR1A, MYC, KDELR3, PID1, DAB2IP, FGF23, PRKCE, IRS1, PKIA, PRKCB, CRHR1, INHBB, MAP4K4, UCN2, HNF4A, GRM7, STC1, CAV3, DRD2, PPM1A, NFKBIA, HK1, SOX4, FKBP1A, FKBP1B, CALCA, OAZ2, PEA15, ACE, BCL2, LRRTM2, LYPLAL1, NEDD4L, ACSL4, AXIN2, UNC119, GNAO1, ABR, NOS1, IL1RN, SIRT6, OXSR1, ATP1A2, FOXP3, SIRT1, NOTCH1, UACA, HDAC1, NEDD4, PLN, GRK6, SVIP, HTR2A | 1.876902547 | 0.00970993 | 1.10E-04 | 0.002541584 |
| GO:0042330~taxis | 63 | 4.618768328 | 1.30E-06 | NRP2, TNFSF14, CXCR1, PIP5K1A, FES, CXCL11, CXCL10, UNC5A, ANK3, GATA3, PDE4B, SERPINE1, SEMA3E, LGI1, MTUS1, PTPRM, EFNB3, EFNB1, LEF1, PADI2, ACKR4, DAPK2, PRKD1, PRKCQ, SEMA4F, LYST, FOXG1, VEGFA, CNTN2, SEMA4C, PDGFRA, PDGFRB, SEMA4B, PLA2G6, RELN, TREM1, PARVA, ABLIM1, CCR1, DAG1, KIT, EPHB2, ALCAM, CALCA, CCL22, PVRL1, RNF165, BCL11B, CSF1R, SWAP70, CREB1, MET, SMAD4, DPYSL4, HGF, GAS1, FOXP1, SEMA6A, EPHA4, CDH13, NOTCH1, RPL13A, CXCL16 | 1.898409346 | 0.00985683 | 1.10E-04 | 0.002580226 |
| GO:0010033~response to organic substance | 262 | 19.20821114 | 1.34E-06 | LDHA, PGF, ADCY5, SNCA, FGFRL1, LHCGR, RORA, SIDT2, BRINP1, CRY2, PLOD1, RAE1, GAB1, SERPINE1, CREB3L1, RARB, LRRC7, DAB2IP, GJD3, GBP5, STRN3, YY1, UBR1, HNF4G, PPARGC1B, CRHR1, HNF4A, SERPINF2, VEGFA, PDGFRA, PDGFRB, STC1, ALDOA, GCNT2, HOXA13, DAG1, HMGCS1, NFKBIA, CALU, PEA15, ACE, IDH1, CDA, KLF6, BMP3, ESRRA, ABR, MAP2K1, IL1RN, SMAD4, MSTN, SKI, HGF, GAS1, USF1, NOTCH2, NOTCH1, NR1I2, KCNJ8, MARCKS, AREG, TRAFD1, KLF4, NCOR2, BMP8B, NRP2, OPRM1, ARSB, CREBRF, HNF1A, CPEB2, CRNKL1, CPEB3, GLRA3, SYNJ1, PPARG, NAP1L1, ZEB1, XYLT1, MAPT, GPX3, EEF2K, GUCY1A3, GPIHBP1, MYC, HYAL3, TP53, CFTR, ERLIN1, ACKR4, IL6R, FOSB, INHBB, CCND1, PLA2G6, CLDN18, FUT8, RAP1GAP, CCR1, TH, FKBP1A, KIT, FKBP1B, GCH1, TPCN2, CCL22, ACSL1, RPL32, XBP1, FAT4, IL10RB, BCL2, BCL9L, AGO4, ACSL4, SHMT1, NOS1, PDK3, AXL, AFF3, ATP1A2, TMPRSS6, SGTA, TRIM21, TP73, CDH13, ERP44, RPS6KA4, DKK1, PLN, SVIP, DPYD, HTR2C, LRP4, TP53INP1, HTR2A, RNASEL, SLC6A1, SLC6A3, CBX3, ILDR2, JAG1, CXCL11, CALB1, CXCL10, SLC2A8, NLRC5, WNT1, SLC16A1, PTGIS, ANK2, MYOCD, PAPPA, GATA3, PDE4B, NQO1, MTUS1, PID1, RET, SATB2, PDXK, SOGA1, FGF23, SOCS7, DLL1, SOCS4, IRS1, NUMBL, MAP4K4, THBD, UCN2, RELT, GPR55, TRIM32, AKAP6, RELN, VAMP2, CPD, GLP1R, CAV3, SYVN1, DRD2, STRAP, ONECUT2, PAQR7, SOX4, PAQR8, LIN28A, RRAGD, GREM2, SRC, NR2C2, IRAK4, GPR22, PEG10, RNF165, AXIN2, HCN3, SEC61A1, CSF1R, MUC2, MYO1C, MET, NR4A2, FOXP1, CDKN1C, IKBKE, PLCG1, HDAC1, UCP3, NEDD4, CXCL16, DNAJB2, NDST1, IL21R, RHOQ, CXCR1, MBP, TMEM173, CDIP1, LTF, PLCB1, FOSL1, IRAK2, ADIPOR2, PADI2, LEF1, PRKCE, PRKCB, PRKD1, MFN2, PRKCQ, ACVR2B, NCOA1, DGAT1, UBE2K, CALCR, KMT2D, RTN4RL1, PPM1A, CALCOCO2, CALCA, RGMB, NPTX1, TOR1A, SELPLG, EHD4, THPO, PIK3R2, IL2RB, GNAO1, CREB1, RCAN1, HID1, COL5A2, SIRT1, PPP1R9B, PRLR, RPL13A, HEYL, SMPD1, IRF1 | 1.30724033 | 0.010174155 | 1.12E-04 | 0.002663718 |
| GO:0023061~signal release | 57 | 4.17888563 | 1.38E-06 | OPRM1, SYT1, HNF1A, ADCY5, SNCA, SYNJ1, PPARG, SYT9, ILDR2, ILDR1, SIDT2, SLC16A1, CRY2, TRIM9, GATA3, STX1A, NOS1AP, NRXN2, FGF23, CFTR, PFKM, PRKCE, PCLO, IRS1, CRHR1, INHBB, MAP4K4, ACVR2B, MYRIP, DGAT1, UCN2, HNF4A, PLA2G6, VAMP2, GLP1R, CLOCK, CPLX2, DRD2, SOX4, SNX4, MYT1, FKBP1B, RIMS4, RIMS3, XBP1, SYN2, PER2, NOS1, CREB1, IL1RN, SMAD4, DGKI, SIRT1, HDAC1, CACNA1E, ADGRL1, HTR2C | 1.971160181 | 0.010440434 | 1.14E-04 | 0.002733799 |
| GO:0009968~negative regulation of signal transduction | 109 | 7.991202346 | 1.53E-06 | SNCA, RORA, NLRC5, WNT1, CRY2, GATA3, SERPINE1, CREB3L1, SPRED1, RNF34, PID1, DAB2IP, STMN3, STRN3, SIX3, PTPRR, SOCS4, UBR1, IRS1, HNF4A, TRIM32, VEGFA, MNT, CAV3, PPP2R3A, SYVN1, STRAP, DRD2, ONECUT2, DAG1, NFKBIA, BDKRB2, GREM2, SRC, PEA15, PEG10, AMER1, TRIM67, OVOL2, AXIN2, BMF, IL1RN, NR4A2, MSTN, SKI, GAS1, HGF, FOXP1, NOTCH1, DLX1, UACA, HDAC1, NEDD4, RGS6, GRK6, KLF4, CREBRF, OPRM1, LZTS2, PPARG, PDCD4, LGR4, TKFC, WWC3, LTF, AGAP2, PGAP2, TP53, LEF1, PADI2, MYADM, PRKCB, LRPAP1, MFN2, PRKCQ, CCND1, PSME3, CLOCK, FAIM2, ZMYND11, RTN4RL1, PPM1A, CTNND1, CALCA, XBP1, BCL2, MARVELD3, BCL6, BCL9L, NFATC4, INPP5D, RASA4, MLLT3, PTPRD, LMNA, TMBIM1, SIRT1, TMPRSS6, SNAI1, TP73, DUSP4, DKK1, ATXN7, HEYL, IRF1, SMPD1, DGKZ, IRF4, LRP4 | 1.583152861 | 0.011579777 | 1.25E-04 | 0.003033872 |
| GO:0006351~transcription, DNA-templated | 241 | 17.6686217 | 1.58E-06 | RORA, DDX17, CRY2, WDR77, MED27, CREB3L1, RARB, TWIST2, DAB2IP, STRN3, TAF4B, YY1, ZHX2, HNF4G, PPARGC1B, MED19, UHRF2, HNF4A, MTF1, SERPINF2, MCIDAS, FOXG1, VEGFA, MNT, TGIF2, ATXN1L, SUPT6H, TNRC18, HOXA13, CRTC1, TFCP2L1, NFKBIA, MYT1, PLAGL1, TAL2, TNKS, PLAG1, ASXL2, KLF6, BMP3, ESRRA, MAP2K1, SMAD4, MSTN, KLF17, SKI, UBP1, USF1, ZBTB42, NOTCH3, NOTCH2, DLX1, NOTCH1, NR1I2, CSRNP3, TRPS1, JAZF1, RBMXL1, TCF12, FOXI1, KLF4, NCOR2, BMP8B, CREBRF, HNF1A, CPEB3, PPARG, MITF, ZEB1, ANP32A, MKX, AGAP2, MYC, TP53, ERLIN1, SPEN, NFAM1, IL6R, FOSB, MYCN, FOXR2, MXD4, CCND1, NSD1, CLOCK, ZMYND11, POLR2F, KIT, XBP1, BCL11B, PER2, GATAD2B, BCL6, BCL9L, ZSCAN29, BAZ2A, NOS1, LMNA, TNP1, PPP1R10, TMPRSS6, SNAI1, ATMIN, TRIM21, TP73, PREB, CDH13, RPS6KA4, PHF19, PKNOX1, DKK1, MAPK13, KDM8, ZBTB4, NEUROD2, TP53INP2, LRP4, TP53INP1, RNASEL, CBX3, JAG1, MAF1, NLRC5, WNT1, PTGIS, MYOCD, OLIG3, MDFIC, GATA3, TGS1, PID1, SATB1, RBFOX2, RET, SATB2, MTA2, SIX3, FGF23, DLL1, PKIA, GRHL2, FOXN3, ELL2, TRIM32, NFE2L1, RELN, GLP1R, CAMTA1, STRAP, DRD2, ONECUT2, SOX4, GREM2, SRC, NR2C2, FOXQ1, OVOL2, RNF165, OVOL1, TFDP2, TAF5, MET, NR4A2, HACE1, WHSC1, FOXP3, TRIM62, FOXP1, FOXP2, CDKN1C, HDAC1, NEDD4, GTF2F1, ZIC5, ATF7, POU6F1, E2F3, ARID4A, E2F5, ARID4B, RHOQ, CBFA2T3, PDCD4, LGR4, TMEM173, MAZ, PRMT2, PEX2, PAX7, RTF1, WWC3, LTF, LRRFIP1, PLCB1, FOSL1, KMT5B, IRAK2, TBL1XR1, KDM7A, FOXJ2, PRKCH, PADI2, LEF1, DDN, PURB, NRIP2, PRKCB, PRKD1, PRKCQ, ACVR2B, NCOA1, MYRF, MDM4, JMJD1C, CUX1, ABLIM1, FRK, KMT2D, ING3, ABLIM3, CTNND2, PPM1A, PPP3R1, CTCFL, CALCA, RGMB, POU2F1, NFATC4, PIK3R2, CREB1, CREB5, SIRT6, SIRT1, ATXN7L3, SP2, ATXN7, HEYL, IRF1, KDM4C, IRF4, RNF41 | 1.324892316 | 0.011951689 | 1.28E-04 | 0.003131898 |
| GO:0000902~cell morphogenesis | 129 | 9.457478006 | 1.60E-06 | SYT1, SRCIN1, FGFRL1, SIDT2, VCL, GP5, ATG5, UNC5A, ANK3, GATA3, TBC1D13, LRRC55, LGI1, DISC1, RAB21, FNDC3B, RBFOX2, DBNL, DAB2IP, RET, PTPRM, ROCK1, EFNB3, EFNB1, MYH9, GRHL2, TBC1D22A, NUMBL, MAP4K4, SLITRK4, VSIG1, MCIDAS, FOXG1, VEGFA, RELN, STC1, STMN1, ALDOA, GCNT2, HOXA13, STRAP, DRD2, TFCP2L1, PEAK1, ONECUT2, DAG1, ULK4, NRN1, SRC, EPHB2, ALCAM, OVOL2, PVRL1, RNF165, TBC1D30, AXIN2, CSF1R, DIXDC1, MAP2K1, MET, NR4A2, SMAD4, GAS1, HGF, TRIM62, FOXP1, CORO1C, EPHA4, SEMA6A, NOTCH1, NEDD4, CDC42SE2, MARCKS, NRP2, RHOJ, WASF1, RHOQ, FES, MBP, MAPT, SEMA3E, EEF2K, CNTNAP1, CAP1, FMNL3, LEF1, COL25A1, MYADM, BBS1, SEMA4F, CLIC5, CNTN2, WNT9B, SEMA4C, SEMA4B, JMJD1C, CUX1, MAP3K14, PARVA, SRGAP2, ABLIM1, ABLIM3, CTNND2, PPP3R1, KIT, NPTX1, FAT3, BCL2, BCL11B, PALM2, BCL6, NFATC4, BCL9L, NEDD4L, DCX, TLX2, TBC1D2, PTPRD, AKIP1, CREB1, AXL, DPYSL4, SNAI1, RAPH1, ATMIN, DKK1, HEYL, EVI5L, LRP4 | 1.512219229 | 0.01213373 | 1.28E-04 | 0.003179893 |
| GO:0022603~regulation of anatomical structure morphogenesis | 107 | 7.84457478 | 1.63E-06 | SYT1, SRCIN1, PGF, TNFSF14, CXCL10, PTGIS, ATG5, GATA3, TBC1D13, SERPINE1, RRAS, RAB21, DISC1, DAB2IP, RET, PTPRM, ROCK1, DLL1, MYH9, TBC1D22A, NUMBL, HNF4A, VEGFA, RELN, TGIF2, ASB4, CAV3, ALDOA, GCNT2, PPP2R3A, STRAP, DAG1, SRC, EPHB2, PPP1R16B, OVOL2, TBC1D30, AXIN2, CSF1R, DIXDC1, ABR, ZMYM4, MAP2K1, MET, IL1RN, NTN4, SMAD4, HGF, TRIM62, FOXP1, FOXP2, CORO1C, SEMA6A, EPHA4, NOTCH1, PLCG1, NEDD4, CDC42SE2, MARCKS, KLF4, RHOJ, RHOQ, FES, LGR4, MBP, MAPT, SEMA3E, CAMSAP1, EEF2K, MYC, FMNL3, LEF1, MYADM, VAT1, PRKCB, PRKD1, ANKRD23, SEMA4F, CNTN2, WNT9B, SEMA4C, SEMA4B, CUX1, PARVA, KIT, XBP1, BCL2, PALM2, NFATC4, BCL9L, NEDD4L, AGO4, TLX2, TBC1D2, MLLT3, INF2, PTPRD, DNM1L, TMBIM1, SIRT1, COL5A2, MARCH5, SNAI1, WNT2B, DKK1, EVI5L, LRP4 | 1.589063583 | 0.012391257 | 1.30E-04 | 0.003247805 |
| GO:0051056~regulation of small GTPase mediated signal transduction | 35 | 2.565982405 | 1.64E-06 | RALGPS2, RALGPS1, RAP1GAP, SIPA1, CYTH4, SHOC2, KITLG, LPAR2, ITSN1, PLEKHG2, TRIM67, RASGRP4, BCL6, RASA4, FGD6, FGD3, ALS2CL, ARHGEF3, DAB2IP, ARHGEF33, ABR, STMN3, MAP2K1, ARPP19, PSD3, ARHGEF9, DGKI, ARHGAP26, FARP2, MFN2, NOTCH2, GPR55, PDGFRB, DGKZ, RELN | 2.467727359 | 0.012425169 | 1.29E-04 | 0.003256749 |
| GO:0045664~regulation of neuron differentiation | 78 | 5.718475073 | 1.83E-06 | ARSB, SYT1, GFAP, SRCIN1, CPEB3, ADCY5, ZEB1, JAG1, FES, MBP, BRINP1, XYLT1, MAPT, RAB29, GATA3, SEMA3E, EEF2K, ANP32A, RARB, DISC1, LRRC7, RAB21, DAB2IP, RET, CCDC88A, SIX3, ZHX2, DLL1, NUMBL, MYCN, PRKD1, MAP4K4, NCOA1, SEMA4F, FOXG1, VEGFA, TRIM32, CNTN2, SEMA4C, SEMA4B, TGIF2, RELN, CUX1, RAP1GAP, CRTC1, ULK4, LIN28A, FKBP1B, EPHB2, TRIM67, FAT3, BCL2, BCL11B, BCL6, NFATC4, NEDD4L, TLX2, DIXDC1, PTPRD, MAP2K1, MET, HGF, SIRT1, TP73, NOTCH3, SEMA6A, EPHA4, NOTCH1, DKK1, NEDD4, HEYL, RGS6, NEUROD2, KDM4C, MARCKS, TCF12, LRP4, KLF4 | 1.742920581 | 0.013882334 | 1.43E-04 | 0.00364136 |
| GO:0071495~cellular response to endogenous stimulus | 123 | 9.017595308 | 1.97E-06 | RNASEL, PGF, SNCA, LHCGR, FGFRL1, CBX3, RORA, SLC2A8, WNT1, MYOCD, PLOD1, GATA3, PDE4B, SERPINE1, RARB, MTUS1, PID1, DAB2IP, SOGA1, FGF23, SOCS7, HNF4G, UBR1, IRS1, CRHR1, UCN2, HNF4A, VEGFA, PDGFRA, PDGFRB, AKAP6, RELN, STC1, VAMP2, GLP1R, CAV3, GCNT2, STRAP, ONECUT2, HMGCS1, PAQR7, PAQR8, RRAGD, GREM2, NR2C2, SRC, SLX4, GPR22, PEG10, RNF165, HCN3, KLF6, BMP3, ESRRA, EME1, MET, IL1RN, NR4A2, SMAD4, MSTN, SKI, USF1, CDKN1C, NOTCH2, NOTCH1, NR1I2, UCP3, PLCG1, MARCKS, BMP8B, KLF4, CREBRF, OPRM1, NDST1, CRNKL1, CPEB2, CPEB3, GLRA3, PPARG, NAP1L1, RHOQ, ZEB1, EEF2K, MYC, ADIPOR2, TP53, LEF1, CFTR, FOSB, PRKCE, PRKCB, INHBB, PRKCQ, ACVR2B, NCOA1, RAP1GAP, FUT8, TH, PPM1A, KIT, CALCA, RGMB, ACSL1, RPL32, XBP1, FAT4, BCL9L, PIK3R2, SHMT1, NOS1, CREB1, ATP1A2, SIRT1, COL5A2, TMPRSS6, CDH13, PPP1R9B, DKK1, PRLR, RPL13A, HEYL, IRF1, LRP4 | 1.524311941 | 0.014919051 | 1.52E-04 | 0.00391534 |
| GO:1902580~single-organism cellular localization | 112 | 8.211143695 | 2.15E-06 | SYT1, CHMP3, PPP2R5A, SNCA, CHMP7, LHCGR, TNFSF14, KIF13A, CRY2, ANK2, MDFIC, ANK3, RAB29, VPS4A, MS4A2, SAR1A, PID1, DAB2IP, ROCK1, SIX3, MYH9, PKIA, PCLO, CDKL2, CRHR1, HNF4A, UBL4A, AKAP6, RELN, VAMP2, CLIP3, CAV3, PACS1, PPIL2, DAG1, CACNB1, NFKBIA, SNX4, GPR143, RIMS4, RIMS3, AXIN2, FCHO2, SEC61A1, GPR158, MYO1C, MAP2K1, SMAD4, CDC23, CRB3, UACA, NEDD4, SPCS2, DPH6, GLRA3, SYNJ1, AP3S2, RHOQ, PIP5K1A, PEX3, TMEM173, AP1S2, TRIM9, PEX2, NMUR1, MAPT, DLG3, SH3PXD2B, STX1A, NRXN2, ZDHHC8, FLOT2, TP53, SRPRA, EDAR, ARHGEF9, MYADM, BBS1, MFN2, PRKCQ, ATG4B, CALCR, CPLX2, ABLIM3, PPFIA1, TH, ATG13, PPP3R1, PPM1A, KIT, ZDHHC23, MOAP1, TSPAN33, XBP1, SEC16A, TOR1A, CHM, BCL6, LYPLAL1, ELMOD1, PIK3R2, SPRN, CNST, TSPAN14, LRRC46, LMNA, TMBIM1, SIRT1, PPP1R9B, KCNN3, ADGRL1, LRP4 | 1.56115727 | 0.016256473 | 1.64E-04 | 0.004269216 |
| GO:0009892~negative regulation of metabolic process | 236 | 17.30205279 | 2.27E-06 | DYNC1LI1, PPP2R5A, SNCA, CRY2, SERPINE1, RARB, TWIST2, RECK, DAB2IP, ROCK1, STRN3, YY1, PTPRR, ZHX2, PPARGC1B, UHRF2, HNF4A, SERPINF2, FOXG1, VEGFA, MNT, TGIF2, ATPIF1, ATXN1L, WFDC5, SUPT6H, TNRC18, TFCP2L1, DAG1, NFKBIA, BDKRB2, MYT1, CALU, ACE, LPCAT1, CDA, NAT10, TNKS, PLAG1, ASXL2, ESRRA, MAP2K1, SMAD4, MSTN, KLF17, SKI, HGF, GAS1, SFMBT2, ZBTB42, NOTCH3, NOTCH2, DLX1, NOTCH1, NR1I2, CSRNP3, TRPS1, JAZF1, KLF4, NCOR2, OPRM1, CREBRF, HNF1A, CPEB2, CPEB3, MITF, PPARG, ZEB1, ANP32A, MKX, AGAP2, MYC, FLOT2, TP53, ERLIN1, SPEN, IL6R, MYADM, MYCN, MXD4, INHBB, CCND1, SERPINB8, GRM7, SERPINB2, NSD1, CLOCK, ZMYND11, CCR1, FKBP1A, FKBP1B, SF3B3, XBP1, BCL2, LRRTM2, PER2, GATAD2B, MLLT1, BCL6, AGO4, BAZ2A, RASA4, NOS1, CNST, SWAP70, PPP1R11, TNP1, PPP1R10, MARCH8, DGKI, TMPRSS6, SNAI1, TRIM21, TP73, DUSP4, PHF19, DKK1, OBFC1, PLN, NEUROD2, ZBTB4, DGKZ, SVIP, HTR2C, NCBP2, SRCIN1, CTDSPL, XPO5, TNFSF14, CBX3, MAF1, NLRC5, WNT1, PTGIS, ATG5, MYOCD, OLIG3, MDFIC, GATA3, CTDSP2, SPRED1, RNF34, NQO1, SAMD4A, PID1, SATB1, RBFOX2, SATB2, SOGA1, MTA2, SIX3, SOCS4, PKIA, FOXN3, UCN2, TRIM32, AKAP6, CAV3, STRAP, DRD2, SOX4, LIN28A, SRC, OAZ2, PPP1R16B, OVOL2, GMIP, CNOT6L, ITGB8, OVOL1, TFDP2, RNF169, LMTK3, MET, NR4A2, ARPP19, WHSC1, RNPS1, FOXP3, TRIM62, FOXP1, FOXP2, CORO1C, CDKN1C, HSPBP1, HDAC1, NEDD4, ATF7, DNAJB2, ARID4A, CBFA2T3, PDCD4, LGR4, PRMT2, PEX2, PCBP4, RTF1, WWC3, LTF, DLG3, PLCB1, IRAK2, TBL1XR1, ANKS1A, CYCS, RIMBP2, LEF1, PRKCE, NRIP2, PURB, ACVR2B, MDM4, CUX1, FRK, KMT2D, RTN4RL1, CALCA, MARVELD3, POU2F1, NFATC4, INPP5D, TNRC6B, FETUB, CREB1, SIRT6, TMBIM1, SIRT1, PPP1R9B, RPL13A, CDAN1, ATXN7, HEYL, SMPD1, IRF1, KDM4C, IGFBP3 | 1.322942419 | 0.017140653 | 1.71E-04 | 0.00450343 |
| GO:0007417~central nervous system development | 111 | 8.137829912 | 2.46E-06 | SYT1, LDHA, SLC6A3, LHCGR, RORA, HOOK3, WNT1, OLIG3, RARB, DISC1, INA, RBFOX2, SATB2, DAB2IP, SIX3, SOCS7, DLL1, GRHL2, NUMBL, CRHR1, NAV2, FOXG1, RELN, STMN1, PFKFB3, DRD2, HMGCS1, SOX4, ULK4, NR2C2, SRC, EPHB2, TAL2, ACE, SBK1, CSF1R, DIXDC1, ABR, B4GALT2, MAP2K1, MET, AK3, NR4A2, ROGDI, SKI, SLC6A17, AK4, GAS1, FOXP1, KCNK3, FOXP2, NOTCH3, EPHA4, NOTCH1, DLX1, HDAC1, ZIC5, MARCKS, AREG, NCOR2, NRP2, ARSB, GFAP, NDST1, CRNKL1, SYNJ1, PPARG, BCAN, ZEB1, UQCRQ, MBP, KCNQ3, MAPT, PAX7, CNTNAP2, PLCB1, CASP2, MYC, ANP32B, TP53, LEF1, PADI2, MYCN, BBS1, INHBB, NCOA1, CNTN2, MYRF, SEMA4C, FAIM2, SRGAP2, CCR1, TH, CTNND1, FAT4, BCL2, BCL11B, SEC16A, SLC4A7, PAFAH1B2, DCX, GNAO1, TRNP1, CREB1, AXL, TP73, WNT2B, PPP1R9B, DKK1, NEUROD2, ADGRL1 | 1.559189107 | 0.018578202 | 1.84E-04 | 0.004884676 |
| GO:0007612~learning | 29 | 2.126099707 | 2.52E-06 | JPH3, SLC6A1, DRD2, SYNJ1, TH, CTNND2, KIT, EPHB2, NETO1, AP1S2, CHST10, CNTNAP2, PLCB1, FOSL1, B4GALT2, NRXN2, CREB1, ATP1A2, DGKI, FOXP2, GMFB, CRHR1, PPP1R9B, GRM7, CNTN2, NEUROD2, CACNA1E, RELN, GLP1R | 2.700037224 | 0.019032263 | 1.87E-04 | 0.005005211 |
| GO:0009628~response to abiotic stimulus | 129 | 9.457478006 | 2.61E-06 | LDHA, PGF, TNFSF14, RORA, CXCL10, SLC2A8, PTGIS, CRY2, MYOCD, PLOD1, GATA3, SERPINE1, RNF34, DISC1, PDXK, YY1, RAD9B, RAD9A, IRS1, CRHR1, UCN2, THBD, HNF4A, TRIM32, PGM1, VEGFA, PDGFRA, PDGFRB, RELN, STC1, NGB, MFAP4, CAV3, ALDOA, DRD2, CRTC1, SIPA1, HMGCS1, SOX4, NFKBIA, BDKRB2, SRC, ACE, ECE1, B4GALT2, MET, NR4A2, SMAD4, MSTN, OXSR1, USF1, KCNK3, FOXP2, EI24, NOTCH1, UACA, UCP3, HDAC1, PLCG1, NEDD4, KCNJ8, CACNA1E, ARSB, OPRM1, FAM175B, CPEB2, PPARG, CBFA2T3, TMEM109, AP1S2, PRMT2, XYLT1, EEF2K, CNTNAP2, ABHD12, MYC, CASP2, FOSL1, STX1A, HYAL3, NRXN2, TP53, FOSB, IL6R, PRKCE, PRKCB, INHBB, PRKCQ, CCND1, XPC, ANKRD23, MDM4, MAP3K14, CLOCK, TH, KIT, NETO1, CALCA, BEST1, XBP1, BCL2, MARVELD3, NFATC4, NEDD4L, UCK2, SCNN1G, DCX, NOS1, SLC12A2, CREB1, PDK3, ASIC2, LMNA, RCAN1, ATP1A2, SIRT1, BRAT1, TP73, CDC25A, AGTRAP, PPP1R9B, RDH11, MAPK13, RPL13A, IRF1, NEUROD2, HTR2A, TP53INP1, OPN4 | 1.498911699 | 0.019715856 | 1.91E-04 | 0.005186783 |
| GO:0007610~behavior | 94 | 6.891495601 | 2.72E-06 | CREBRF, OPRM1, JPH3, SLC6A1, CPEB3, SLC6A3, ADCY5, SNCA, SYNJ1, TNFSF14, CXCL11, CALB1, CXCL10, SLC16A1, AP1S2, CHST10, MAPT, SERPINE1, SEMA3E, CNTNAP2, ABHD12, PLCB1, FOSL1, MTUS1, GRID1, CNTNAP4, NRXN2, EFNB3, ZDHHC8, TP53, SIX3, PADI2, DAPK2, PRKCE, BBS1, PRKD1, CRHR1, NCOA1, NAPEPLD, DGAT1, NAV2, CELF6, SEMA4F, GRM7, VEGFA, CNTN2, PDGFRA, SEMA4C, PDGFRB, SEMA4B, PLA2G6, RELN, GLP1R, SCN1A, DRD2, CCR1, KCNA2, CRTC1, TH, CTNND2, KIT, NR2C2, EPHB2, NETO1, CALCA, ACE, BCL2, MYO15A, UCK2, NOS1, B4GALT2, GNAO1, SWAP70, CREB1, MET, IL1RN, NR4A2, AFF2, RCAN1, ATP1A2, DGKI, FOXP2, GMFB, CDH13, PPP1R9B, EPHA4, SEMA6A, NOTCH1, NEUROD2, TMOD2, CACNA1E, ADAM22, HTR2C, HTR2A | 1.629221704 | 0.020527632 | 1.98E-04 | 0.005402567 |
| GO:1903649~regulation of cytoplasmic transport | 58 | 4.252199413 | 2.97E-06 | NCBP2, DPH6, XPO4, LHCGR, SNCA, TNFSF14, CXCL11, CXCL10, TMEM173, ANK2, ATG5, MDFIC, NMUR1, MS4A2, SAR1A, RAB21, DAB2IP, ANP32B, ZDHHC8, EDAR, PRKCE, CDKL2, PKIA, PRKD1, PRKCQ, HNF4A, AKAP6, ATPIF1, SUPT6H, ABLIM3, ATG13, PPM1A, PPP3R1, NFKBIA, SOX4, FKBP1A, FKBP1B, SRC, CALCA, XBP1, BCL2, LYPLAL1, AXIN2, PIK3R2, ELMOD1, CNST, MAP2K1, SMAD4, LRRC46, PTPN14, GAS1, SIRT1, UACA, PLCG1, NEDD4, PLN, KCNN3, SVIP | 1.910230417 | 0.022429107 | 2.14E-04 | 0.005908709 |
| GO:0070887~cellular response to chemical stimulus | 223 | 16.34897361 | 3.10E-06 | SYT1, PGF, ADCY5, SNCA, FGFRL1, LHCGR, RORA, BRINP1, PLOD1, RAE1, GAB1, SERPINE1, CREB3L1, RARB, DAB2IP, GBP5, YY1, HNF4G, UBR1, PPARGC1B, CRHR1, HNF4A, VEGFA, PDGFRA, PDGFRB, STC1, GCNT2, HOXA13, HMGCS1, NFKBIA, BDKRB2, ACE, KLF6, BMP3, ESRRA, IL1RN, SMAD4, MSTN, SKI, HGF, GAS1, USF1, KCNK3, NOTCH2, NOTCH1, NR1I2, MARCKS, KLF4, BMP8B, NRP2, OPRM1, CREBRF, CPEB2, CRNKL1, CPEB3, GLRA3, PPARG, NAP1L1, ZEB1, MAPT, EEF2K, MYC, HYAL3, TP53, CFTR, IL6R, FOSB, ACKR4, INHBB, CCND1, PLA2G6, PARVA, CLDN18, RAP1GAP, FUT8, CCR1, TH, FKBP1A, KIT, FKBP1B, GCH1, CCL22, ACSL1, RPL32, XBP1, FAT4, IL10RB, BCL2, BCL9L, AGO4, RASA4, SHMT1, NOS1, SWAP70, PDK3, AXL, LMNA, ATP1A2, TMPRSS6, CDH13, RPS6KA4, DKK1, NEUROD2, LRP4, TP53INP1, RNASEL, TNFSF14, CBX3, CXCL11, CALB1, CXCL10, SLC2A8, NLRC5, WNT1, SLC16A1, PTGIS, MYOCD, ANK3, GATA3, PDE4B, NQO1, MTUS1, PID1, RET, SATB2, SOGA1, FGF23, SOCS7, DLL1, SOCS4, IRS1, NUMBL, MAP4K4, UCN2, RELT, LYST, TRIM32, AKAP6, NFE2L1, RELN, VAMP2, CPD, NGB, GLP1R, CAV3, SYVN1, STRAP, SIPA1, ONECUT2, SOX4, PAQR7, PAQR8, RRAGD, LIN28A, GREM2, NR2C2, SRC, IRAK4, PEG10, GPR22, RNF165, AXIN2, HCN3, CSF1R, MUC2, LPO, MYO1C, MET, NR4A2, CDKN1C, UACA, UCP3, PLCG1, HDAC1, NEDD4, CXCL16, NDST1, IL21R, RHOQ, CXCR1, PIP5K1A, TMEM173, KCNQ3, CDIP1, LTF, PLCB1, IRAK2, ADIPOR2, PADI2, LEF1, DAPK2, PRKCE, PRKCB, PRKD1, PRKCQ, ACVR2B, NCOA1, UBE2K, MDM4, TREM1, RTN4RL1, PPM1A, CALCA, RGMB, NPTX1, TOR1A, NFATC4, UCK2, SELPLG, THPO, PIK3R2, EHD4, IL2RB, NCEH1, CREB1, ASIC2, COL5A2, SIRT1, PPP1R9B, PRLR, RPL13A, HEYL, IRF1 | 1.331248423 | 0.023382933 | 2.21E-04 | 0.006162972 |
| GO:0048585~negative regulation of response to stimulus | 135 | 9.897360704 | 3.75E-06 | SLC6A3, SNCA, RORA, NLRC5, WNT1, GP5, PTGIS, CRY2, GATA3, SERPINE1, CREB3L1, SPRED1, RNF34, PID1, DAB2IP, STMN3, STRN3, PTPRR, SIX3, SOCS4, UBR1, IRS1, CRHR1, MAP4K4, NAPEPLD, THBD, HNF4A, SERPINF2, TRIM32, VEGFA, PDGFRA, MNT, NGB, CAV3, SYVN1, PPP2R3A, STRAP, DRD2, ONECUT2, DAG1, NFKBIA, BDKRB2, GREM2, SRC, PEA15, PEG10, AMER1, TRIM67, OVOL2, RNF169, AXIN2, BMF, ABR, MET, IL1RN, NR4A2, MSTN, SKI, GAS1, HGF, FOXP3, FOXP1, EPHA4, SEMA6A, NOTCH1, DLX1, UACA, HDAC1, NEDD4, RGS6, GRK6, TRAFD1, KLF4, CREBRF, OPRM1, LZTS2, PPARG, PDCD4, LGR4, XYLT1, TKFC, WWC3, SEMA3E, LTF, AGAP2, PGAP2, TP53, LEF1, PADI2, CDK6, MYADM, PRKCB, LRPAP1, MFN2, PRKCQ, CCND1, SEMA4F, SEMA4C, SEMA4B, PSME3, CLOCK, FAIM2, ZMYND11, RTN4RL1, CCR1, PPM1A, CTNND1, FKBP1B, CALCA, XBP1, BCL2, MARVELD3, BCL6, NFATC4, BCL9L, INPP5D, RASA4, MLLT3, PTPRD, LMNA, TMBIM1, SNAI1, TMPRSS6, SIRT1, TP73, DUSP4, DKK1, ATXN7, HEYL, IRF1, SMPD1, DGKZ, SVIP, IRF4, LRP4 | 1.472061301 | 0.028206932 | 2.65E-04 | 0.007452721 |
| GO:0051241~negative regulation of multicellular organismal process | 113 | 8.284457478 | 3.89E-06 | ADCY5, JAG1, CXCL10, HOOK3, GP5, CRY2, MYOCD, GATA3, RAB29, PDE4B, SERPINE1, WDR77, RARB, FNDC3B, TWIST2, DAB2IP, PTPRM, ATG9A, ROCK1, ZHX2, SIX3, PTPRR, FGF23, DLL1, GRHL2, MAP4K4, UCN2, THBD, GPR55, SERPINF2, FOXG1, VEGFA, PDGFRA, STC1, CAV3, STRAP, DRD2, NFKBIA, LIN28A, EPHB2, OVOL2, AXIN2, ARHGDIB, DIXDC1, ZBTB46, ABR, MET, MSTN, SKI, HGF, FOXP3, TRIM62, FOXP1, CORO1C, NOTCH3, SEMA6A, EPHA4, NOTCH1, DLX1, AREG, ADRA1D, KLF4, GFAP, MRVI1, PPARG, LGR4, MBP, XYLT1, WWC3, SEMA3E, ANP32A, GUCY1A3, MYC, TP53, LEF1, CDK6, IL6R, MYCN, LRPAP1, INHBB, SEMA4F, CNTN2, SEMA4C, SEMA4B, SRGAP2, CALCR, CLDN18, RAP1GAP, CCR1, FKBP1B, CALCA, FAT3, BCL2, MARVELD3, BCL6, NFATC4, INPP5D, TLX2, NOS1, LMNA, AXL, ATP1A2, SNAI1, SIRT1, COL5A2, TMPRSS6, TP73, DKK1, PLN, IRF1, NEUROD2, LRP4, TOB2 | 1.536751136 | 0.029245933 | 2.72E-04 | 0.007731345 |
| GO:0016570~histone modification | 55 | 4.032258065 | 4.12E-06 | KMT2D, ING3, HNF1A, ARID4A, SNCA, ARID4B, CTCFL, JADE2, PRMT2, ATG5, MYOCD, BRPF3, NAA50, XBP1, PAX7, GATA3, RTF1, ANP32A, PER2, BCL6, BAZ2A, KMT5B, KDM5D, ASXL2, SATB1, KDM7A, TBL1XR1, NOS1, TAF5, MTA2, TP53, SMAD4, PADI2, LEF1, WHSC1, SIRT6, FOXP3, SIRT1, ATXN7L3, PRKCB, PRKD1, MSL2, NCOA1, RPS6KA4, PHF19, HDAC1, ATXN7, TRPS1, VEGFA, KDM8, KDM4C, IRF4, NSD1, CLOCK, SUPT6H | 1.929561835 | 0.030967412 | 2.86E-04 | 0.008193641 |
| GO:0051580~regulation of neurotransmitter uptake | 8 | 0.586510264 | 4.17E-06 | GFAP, RAB3B, NOS1, DRD2, TOR1A, SNCA, PER2, NAT8L | 9.682892115 | 0.031340425 | 2.87E-04 | 0.00829392 |
| GO:0034613~cellular protein localization | 139 | 10.19061584 | 4.37E-06 | XPO5, PPP2R5A, XPO4, LHCGR, TNFSF14, VCL, HOOK3, KIF13A, CRY2, ANK2, ANK3, MDFIC, RAB29, VPS4A, SAR1A, DISC1, PID1, DAB2IP, ATG9A, ROCK1, SIX3, CDKL2, PKIA, IRS1, VTI1A, HNF4A, VEGFA, UBL4A, SURF4, AKAP6, ATPIF1, RELN, VAMP2, CLIP3, CAV3, PACS1, SNX9, SYVN1, COPZ1, PPIL2, CACNB1, DAG1, SOX4, NFKBIA, LMAN1, SRC, OAZ2, STX17, TNKS, AXIN2, FCHO2, SEC61A1, GPR158, MYO1C, PTPN14, SMAD4, CRB3, WIPI2, GAS8, UACA, NEDD4, DCP1A, MARCKS, SPCS2, LZTS2, AP1B1, DPH6, GLRA3, AP3S2, RHOQ, PEX3, PIP5K1A, TMEM173, AP1S2, NMUR1, PEX2, DLG3, CNTNAP2, CNTNAP1, GPIHBP1, KDELR3, SH3PXD2B, STX1A, ANP32B, NRXN2, FLOT2, RAB4A, ZDHHC8, TP53, SRPRA, ARHGEF9, EDAR, MYADM, BBS1, MFN2, PRKCQ, MYRIP, ATG4B, CNTN2, TOM1, CALCR, CLDN18, ABLIM3, PPFIA1, ATG13, PPP3R1, PPM1A, HK1, ZDHHC23, MOAP1, TSPAN33, XBP1, TMEM33, TOR1A, CHM, BCL6, NEDD4L, LYPLAL1, ELMOD1, PIK3R2, SPRN, DNM1L, CNST, TSPAN14, LRRC46, LMNA, MAL, TMBIM1, MID1, HID1, MARCH5, SIRT1, COG3, PPP1R9B, KCNN3, SVIP, LRP4, TP53INP2, HTR2A | 1.457677261 | 0.032794155 | 2.98E-04 | 0.008685102 |
| GO:0016482~cytoplasmic transport | 103 | 7.551319648 | 5.09E-06 | NCBP2, CHMP3, XPO5, XPO4, LHCGR, CHMP7, SNCA, TNFSF14, CXCL11, CXCL10, HOOK3, KIF13A, CRY2, ATG5, ANK2, MDFIC, ANK3, RAB29, MS4A2, SAR1A, RAB21, DAB2IP, SIX3, ERGIC1, PKIA, CDKL2, VTI1A, HNF4A, SGSM2, LYST, SURF4, AKAP6, ATPIF1, VAMP2, GLP1R, SUPT6H, PACS1, SYVN1, DRD2, DAG1, NFKBIA, SOX4, LMAN1, SRC, STX17, AXIN2, SEC61A1, SLC30A6, MAP2K1, SMAD4, PTPN14, GAS1, UACA, PLCG1, AKTIP, NEDD4, SPCS2, JPH3, LZTS2, DPH6, PEX3, TMEM173, PEX2, NMUR1, ANP32A, ANP32B, RAB4A, ZDHHC8, TP53, SRPRA, EDAR, PRKCE, BBS1, PRKD1, MFN2, PRKCQ, ABLIM3, ATG13, BET1, PPP3R1, PPM1A, FKBP1A, FKBP1B, TPCN2, CALCA, XBP1, BCL2, SEC16A, BCL6, LYPLAL1, PIK3R2, ELMOD1, SPRN, CNST, LRRC46, LMNA, SIRT1, COG3, KCNN3, PLN, SVIP, HTR2C, HTR2A | 1.564860703 | 0.038066909 | 3.43E-04 | 0.010108876 |
| GO:0046903~secretion | 112 | 8.211143695 | 5.50E-06 | SYT1, KCNC3, CHMP3, SLC6A1, SRCIN1, VTCN1, ADCY5, SLC6A3, SNCA, LHCGR, SYT9, ILDR2, ILDR1, ITSN1, SIDT2, PNP, SLC16A1, CRY2, ATG5, GATA3, VPS4A, MS4A2, CREB3L1, LGI3, RAB21, GBP5, FGF23, IRS1, PCLO, CRHR1, MAP4K4, PNPLA8, UCN2, HNF4A, LYST, VEGFA, STC1, VAMP2, GLP1R, DRD2, KCNA2, SOX4, SNX4, BDKRB2, MYT1, RIMS4, SRC, RIMS3, ACE, GOLPH3L, TMEM79, CSF1R, ABR, MET, IL1RN, SMAD4, FOXP3, FOXP1, NOTCH2, NOTCH1, HDAC1, PLCG1, CACNA1E, NCOR2, OPRM1, HNF1A, SYNJ1, PPARG, FES, LGR4, TRIM9, PLCB1, MYC, STX1A, NOS1AP, NRXN2, CFTR, PFKM, PRKCE, PRKCB, INHBB, PRKCQ, ACVR2B, MYRIP, NCOA1, CCND1, DGAT1, GRM7, PLA2G6, TREM1, CLOCK, CPLX2, RAB3B, CCR1, KIT, FKBP1B, XBP1, ARHGAP44, SYN2, PER2, ACSL4, DNM1L, NOS1, CREB1, AXL, DGKI, SIRT1, TP73, PRLR, ADGRL1, HTR2C, HTR2A | 1.528877702 | 0.041135207 | 3.68E-04 | 0.010940979 |
| GO:0008283~cell proliferation | 169 | 12.39002933 | 5.62E-06 | PGF, VTCN1, FGFRL1, RORA, TPD52, SIDT2, PNP, HOOK3, CXCL10, WNT1, CD47, MYOCD, GATA3, SERPINE1, WDR77, SLC25A27, RARB, DISC1, FNDC3B, TWIST2, PID1, SATB1, DAB2IP, PTPRM, PDXK, TAF4B, EFNB1, SIX3, DLL1, IRS1, GRHL2, NUMBL, OGFOD1, TNS2, UHRF2, UCN2, HNF4A, SERPINF2, TBRG1, VEGFA, TRIM32, FOXG1, EIF2S2, PDGFRA, PDGFRB, ATPIF1, STC1, ATXN1L, GLP1R, PLA2G2F, CAV3, FZR1, GCNT2, GNAI2, DRD2, STRAP, SOX4, BDKRB2, LIN28A, SRC, IRAK4, PPP1R16B, ACE, OVOL2, CNOT6L, OVOL1, FBXW4, AXIN2, CSF1R, PLAG1, DIXDC1, MUC2, MAP2K1, MET, PTPN14, SMAD4, ROGDI, MSTN, SKI, HGF, DOCK8, GAS1, FOXP3, FOXP1, FOXP2, NOTCH3, CDKN1C, NOTCH2, NOTCH1, HDAC1, DNAJB2, AREG, KLF4, NCOR2, LZTS2, GFAP, E2F3, MARCKSL1, PPARG, NAP1L1, ZEB1, CBFA2T3, LGR4, WISP2, FAM83A, RSPO1, HPSE, PEX2, PAX7, LTF, TGFA, AGAP2, MYC, FOSL1, ANP32B, TP53, PRKCH, LEF1, CDK6, IL6R, PURB, MYCN, BBS1, PRKD1, MFN2, PRKCQ, CCND1, XIRP1, ADK, MDM4, SRGAP2, KMT2D, RAP1GAP, KITLG, FKBP1A, KIT, FKBP1B, CALCA, XBP1, FAT4, RASGRP4, BCL2, BCL11B, MARVELD3, PER2, GMNC, BCL6, INPP5D, THPO, NOS1, CR2, TRNP1, CREB1, AXL, LMNA, SIRT6, BRAT1, SIRT1, TP73, WNT2B, CDH13, PPP1R8, SP2, IRF1, KDM4C, IGFBP3, RNF41, TP53INP1, HTR2A | 1.392293336 | 0.04196394 | 3.73E-04 | 0.011166182 |
| GO:0070727~cellular macromolecule localization | 139 | 10.19061584 | 5.69E-06 | XPO5, PPP2R5A, XPO4, LHCGR, TNFSF14, VCL, HOOK3, KIF13A, CRY2, ANK2, ANK3, MDFIC, RAB29, VPS4A, SAR1A, DISC1, PID1, DAB2IP, ATG9A, ROCK1, SIX3, CDKL2, PKIA, IRS1, VTI1A, HNF4A, VEGFA, UBL4A, SURF4, AKAP6, ATPIF1, RELN, VAMP2, CLIP3, CAV3, PACS1, SNX9, SYVN1, COPZ1, PPIL2, CACNB1, DAG1, SOX4, NFKBIA, LMAN1, SRC, OAZ2, STX17, TNKS, AXIN2, FCHO2, SEC61A1, GPR158, MYO1C, PTPN14, SMAD4, CRB3, WIPI2, GAS8, UACA, NEDD4, DCP1A, MARCKS, SPCS2, LZTS2, AP1B1, DPH6, GLRA3, AP3S2, RHOQ, PEX3, PIP5K1A, TMEM173, AP1S2, NMUR1, PEX2, DLG3, CNTNAP2, CNTNAP1, GPIHBP1, KDELR3, SH3PXD2B, STX1A, ANP32B, NRXN2, FLOT2, RAB4A, ZDHHC8, TP53, SRPRA, ARHGEF9, EDAR, MYADM, BBS1, MFN2, PRKCQ, MYRIP, ATG4B, CNTN2, TOM1, CALCR, CLDN18, ABLIM3, PPFIA1, ATG13, PPP3R1, PPM1A, HK1, ZDHHC23, MOAP1, TSPAN33, XBP1, TMEM33, TOR1A, CHM, BCL6, NEDD4L, LYPLAL1, ELMOD1, PIK3R2, SPRN, DNM1L, CNST, TSPAN14, LRRC46, LMNA, MAL, TMBIM1, MID1, HID1, MARCH5, SIRT1, COG3, PPP1R9B, KCNN3, SVIP, LRP4, TP53INP2, HTR2A | 1.449305819 | 0.04247331 | 3.74E-04 | 0.011304697 |
| GO:0051129~negative regulation of cellular component organization | 74 | 5.425219941 | 5.94E-06 | DYNC1LI1, LIMA1, GFAP, PPP2R5A, ADCY5, SNCA, RHOQ, MBP, CXCL10, ATG5, TRIM9, XYLT1, RAB29, SEMA3E, ANP32A, SAR1A, PID1, DBNL, DAB2IP, ROCK1, TP53, MYH9, MYADM, VAT1, MAP4K4, PLEKHH2, SEMA4F, SEMA4C, SEMA4B, CLIP3, STMN1, ADD2, SUPT6H, MAP6D1, CAV3, RAP1GAP, STRAP, PPFIA1, PPM1A, SRC, EPHB2, ACE, FAT3, OVOL2, LRRTM2, TNKS, NAT10, NFATC4, LYPLAL1, TBC1D30, TLX2, UNC119, SWAP70, MAP1A, LMNA, HGF, TMBIM1, MID1, FOXP3, TRIM62, SIRT1, GMFB, CORO1C, SEMA6A, EPHA4, RPL13A, NEUROD2, TMOD2, KDM4C, DNAJB2, SVIP, EVI5L, DNAJB1, LRP4 | 1.716934544 | 0.044350297 | 3.88E-04 | 0.011815746 |
| GO:0050890~cognition | 43 | 3.152492669 | 6.00E-06 | JPH3, TUSC3, SLC6A1, CPEB3, DRD2, CRTC1, LHCGR, SYNJ1, TH, CTNND2, KIT, CALB1, EPHB2, NETO1, AP1S2, CHST10, MAPT, CNTNAP2, PLCB1, FOSL1, JAKMIP1, B4GALT2, NRXN2, CREB1, IL1RN, RCAN1, AFF2, ATP1A2, DGKI, FOXP2, GMFB, CRHR1, PPP1R9B, MAGT1, GRM7, CNTN2, TMOD2, NEUROD2, PLA2G6, CACNA1E, RELN, GLP1R, HTR2A | 2.109954532 | 0.044787124 | 3.88E-04 | 0.011934826 |
| GO:0006836~neurotransmitter transport | 30 | 2.19941349 | 6.09E-06 | SYT1, GFAP, RAB3B, CPLX2, KCNC3, SLC6A1, DRD2, SLC6A3, SYNJ1, SNCA, RIMS4, RIMS3, XBP1, TRIM9, SYN2, TOR1A, PER2, STX1A, NOS1, NOS1AP, NRXN2, SLC6A14, SLC6A17, ATP1A2, DGKI, PCLO, ADGRL1, VAMP2, HTR2C, NAT8L | 2.533314798 | 0.045438191 | 3.91E-04 | 0.012112407 |
| GO:0000122~negative regulation of transcription from RNA polymerase II promoter | 84 | 6.158357771 | 6.28E-06 | CREBRF, HNF1A, CPEB3, MITF, PPARG, ZEB1, CBFA2T3, CRY2, MYOCD, OLIG3, PEX2, GATA3, WWC3, RTF1, MKX, RARB, MYC, TWIST2, SATB1, TBL1XR1, SATB2, DAB2IP, MTA2, YY1, STRN3, ZHX2, TP53, LEF1, SPEN, PKIA, PURB, NRIP2, ACVR2B, CCND1, FOXG1, VEGFA, MNT, TGIF2, MDM4, ATXN1L, CUX1, NSD1, ZMYND11, FRK, STRAP, TFCP2L1, XBP1, OVOL1, PER2, GATAD2B, BCL6, NFATC4, ASXL2, ESRRA, MET, NR4A2, SMAD4, KLF17, SKI, WHSC1, FOXP3, SNAI1, SIRT1, TMPRSS6, TP73, FOXP1, FOXP2, ZBTB42, NOTCH3, CDKN1C, NOTCH2, NOTCH1, DLX1, DKK1, PHF19, HDAC1, NEDD4, TRPS1, ATF7, HEYL, ZBTB4, JAZF1, KLF4, NCOR2 | 1.648708657 | 0.046798733 | 3.99E-04 | 0.012483892 |
| GO:0006468~protein phosphorylation | 164 | 12.02346041 | 6.29E-06 | RNASEL, FASTKD2, SRCIN1, CTDSPL, SNCA, STK35, LPAR2, S1PR2, WNT1, MYOCD, MDFIC, GAB1, RRAS, CTDSP2, SPRED1, PID1, RET, DAB2IP, ROCK1, CSNK1G1, PTPRR, FGF23, SOCS4, CDKL2, PKIA, MAP4K4, GPR55, HNF4A, RELT, SERPINF2, VEGFA, PDGFRA, PDGFRB, AKAP6, RELN, CLIP3, ERC1, NEK5, CAV3, SNX9, GCNT2, GNAI2, STRAP, DRD2, PEAK1, DAG1, EPHA10, BDKRB2, ULK4, SRC, NR2C2, EPHB2, IRAK4, ACE, ECE1, SBK1, TNKS, AXIN2, STK38L, CSF1R, DIXDC1, BMP3, UNC119, MAP2K1, IL1RN, MET, SMAD4, MSTN, HGF, OXSR1, RPS6KL1, CORO1C, CDKN1C, EPHA4, IKBKE, AKTIP, HDAC1, GRK6, MARCKS, KLF4, BMP8B, OPRM1, NDST1, PRKAG1, FES, PDCD4, CCNE2, RSPO1, EEF2K, LTF, TGFA, PLCB1, AGAP2, MYC, IRAK2, CCDC88A, PHKG2, TP53, PRKCH, CDK6, IL6R, DAPK2, PRKCE, MYADM, TBCK, PRKCB, PRKD1, MAST3, INHBB, PRKCQ, ACVR2B, CCND1, KSR2, MAP3K15, UBE2K, SEMA4C, PLA2G6, KSR1, MAP3K14, NSD1, FRK, ZMYND11, RTN4RL1, SNX15, CCR1, ATG13, CTNND1, HK1, KITLG, BCCIP, FKBP1A, KIT, CALCA, CCL22, ACSL1, XBP1, SPEG, BCL2, MARVELD3, MLLT1, DCX, EHD4, THPO, NOS1, PDK3, CREB1, AXL, MID1, SIRT1, BRAT1, TP73, PPP1R9B, DUSP4, RPS6KA4, DKK1, PRLR, MAPK13, IRF1, SMPD1, IGFBP3, HTR2C, LRP4, RNF41, HTR2A | 1.396243529 | 0.046880935 | 3.97E-04 | 0.012506353 |
| GO:0072091~regulation of stem cell proliferation | 24 | 1.759530792 | 6.29E-06 | DRD2, TAF4B, TP53, LMNA, SIX3, KITLG, SIRT6, ZEB1, GAS1, IRS1, FOXP1, FOXP2, MYCN, NOTCH1, ACE, OVOL2, FOXG1, FBXW4, OVOL1, VEGFA, ATXN1L, MYC, DISC1, THPO | 2.904867635 | 0.046884855 | 3.94E-04 | 0.012507425 |
| GO:0050769~positive regulation of neurogenesis | 59 | 4.325513196 | 6.51E-06 | OPRM1, ARSB, SYT1, GFAP, CPEB3, SYNJ1, PPARG, ZEB1, FES, BRINP1, MAPT, EEF2K, RARB, MYC, LRRC7, RAB21, DISC1, RET, DAB2IP, PRKCH, SPEN, NUMBL, PRKD1, NCOA1, FOXG1, TRIM32, VEGFA, RELN, TGIF2, CUX1, DRD2, CRTC1, KIT, LIN28A, FKBP1B, EPHB2, ACE, TRIM67, BCL2, BCL6, NEDD4L, PLAG1, DIXDC1, PTPRD, MAP2K1, MET, HGF, SIRT1, TP73, SEMA6A, EPHA4, NOTCH1, HDAC1, HEYL, RGS6, NEUROD2, KDM4C, MARCKS, TCF12 | 1.85083359 | 0.048474424 | 4.04E-04 | 0.012942153 |
| GO:0072089~stem cell proliferation | 29 | 2.126099707 | 6.85E-06 | DRD2, KITLG, ZEB1, WNT1, ACE, OVOL2, FAT4, OVOL1, FBXW4, MYC, DISC1, THPO, TAF4B, TP53, SIX3, LMNA, LEF1, SIRT6, GAS1, IRS1, FOXP1, WNT2B, MYCN, NUMBL, FOXP2, NOTCH1, FOXG1, VEGFA, ATXN1L | 2.568328091 | 0.050956697 | 4.22E-04 | 0.013622476 |
| GO:0061024~membrane organization | 88 | 6.451612903 | 7.01E-06 | SYT1, CHMP3, AP1B1, PPP2R5A, GLRA3, SNCA, PPARG, VPS37B, RHOQ, PIP5K1A, PEX3, MBP, KIF13A, ANK2, ANK3, TRIM9, FAM73B, RAB29, VPS4A, DLG3, CNTNAP2, SAR1A, MYC, PID1, SH3PXD2B, STX1A, ATG9A, CCDC88A, ROCK1, NRXN2, FLOT2, TP53, SRPRA, ARHGEF9, MYH9, MYADM, VTI1A, VAT1, ELMO1, BBS1, MFN2, LETM1, ATG4B, CNTN2, MYRF, UBL4A, RELN, ATPIF1, CLIP3, VAMP2, PACS1, CALCR, CAV3, SNX9, PPIL2, PPFIA1, BET1, CACNB1, CALCOCO2, EEA1, ZDHHC23, MOAP1, TSPAN33, FAT4, BCL2, STX17, TOR1A, SEC16A, PVRL2, CHM, LYPLAL1, FCHO2, SEC61A1, PIK3R2, HIP1, GPR158, PTPRD, DNM1L, CNST, MYO1C, LMNA, TSPAN14, HACE1, CRB3, TMBIM1, WIPI2, PPP1R9B, LRP4 | 1.622007309 | 0.052113438 | 4.28E-04 | 0.013940114 |
| GO:1902679~negative regulation of RNA biosynthetic process | 121 | 8.870967742 | 7.13E-06 | CBX3, MAF1, NLRC5, PTGIS, CRY2, MYOCD, OLIG3, MDFIC, GATA3, RARB, TWIST2, RBFOX2, SATB1, SATB2, DAB2IP, MTA2, YY1, STRN3, ZHX2, SIX3, PKIA, PPARGC1B, FOXN3, UHRF2, HNF4A, TRIM32, FOXG1, VEGFA, MNT, TGIF2, ATXN1L, TNRC18, STRAP, TFCP2L1, NFKBIA, SRC, OVOL2, OVOL1, TFDP2, ASXL2, ESRRA, MET, NR4A2, SMAD4, KLF17, WHSC1, SKI, FOXP3, TRIM62, FOXP1, ZBTB42, FOXP2, CDKN1C, NOTCH3, NOTCH2, NOTCH1, DLX1, NR1I2, HDAC1, NEDD4, TRPS1, ATF7, JAZF1, NCOR2, KLF4, CREBRF, HNF1A, ARID4A, CPEB3, MITF, PPARG, ZEB1, CBFA2T3, PDCD4, LGR4, PRMT2, PEX2, WWC3, RTF1, MKX, PLCB1, MYC, IRAK2, TBL1XR1, TP53, LEF1, SPEN, PURB, NRIP2, MXD4, ACVR2B, CCND1, MDM4, CUX1, NSD1, CLOCK, KMT2D, FRK, ZMYND11, CALCA, XBP1, POU2F1, PER2, GATAD2B, BCL6, NFATC4, BAZ2A, CREB1, TNP1, SIRT6, SNAI1, SIRT1, TMPRSS6, TRIM21, TP73, PHF19, DKK1, HEYL, IRF1, NEUROD2, ZBTB4 | 1.491888726 | 0.052941681 | 4.32E-04 | 0.014167785 |
| GO:0016569~covalent chromatin modification | 55 | 4.032258065 | 7.28E-06 | KMT2D, ING3, HNF1A, ARID4A, SNCA, ARID4B, CTCFL, JADE2, PRMT2, ATG5, MYOCD, BRPF3, NAA50, XBP1, PAX7, GATA3, RTF1, ANP32A, PER2, BCL6, BAZ2A, KMT5B, KDM5D, ASXL2, SATB1, KDM7A, TBL1XR1, NOS1, TAF5, MTA2, TP53, SMAD4, PADI2, LEF1, WHSC1, SIRT6, FOXP3, SIRT1, ATXN7L3, PRKCB, PRKD1, MSL2, NCOA1, RPS6KA4, PHF19, HDAC1, ATXN7, TRPS1, VEGFA, KDM8, KDM4C, IRF4, NSD1, CLOCK, SUPT6H | 1.892982463 | 0.054057478 | 4.37E-04 | 0.014474813 |
| GO:0001505~regulation of neurotransmitter levels | 28 | 2.052785924 | 7.66E-06 | SYT1, RAB3B, CPLX2, DRD2, SLC6A3, SYNJ1, SNCA, TH, RIMS4, RIMS3, XBP1, TRIM9, SYN2, TOR1A, PER2, STX1A, NOS1, NOS1AP, NRXN2, MAOA, ATP1A2, DGKI, PCLO, CELF6, ADGRL1, VAMP2, HTR2C, NAT8L | 2.606932493 | 0.056808422 | 4.57E-04 | 0.015233324 |
| GO:0045892~negative regulation of transcription, DNA-templated | 118 | 8.651026393 | 8.65E-06 | CBX3, MAF1, NLRC5, PTGIS, CRY2, MYOCD, OLIG3, MDFIC, GATA3, RARB, TWIST2, RBFOX2, SATB1, SATB2, DAB2IP, MTA2, YY1, STRN3, ZHX2, SIX3, PKIA, PPARGC1B, FOXN3, UHRF2, HNF4A, FOXG1, VEGFA, MNT, TGIF2, ATXN1L, TNRC18, STRAP, TFCP2L1, NFKBIA, SRC, OVOL2, OVOL1, TFDP2, ASXL2, ESRRA, MET, NR4A2, SMAD4, KLF17, WHSC1, SKI, FOXP3, FOXP1, ZBTB42, FOXP2, CDKN1C, NOTCH3, NOTCH2, NOTCH1, DLX1, NR1I2, HDAC1, NEDD4, TRPS1, ATF7, JAZF1, NCOR2, KLF4, CREBRF, HNF1A, ARID4A, CPEB3, MITF, PPARG, ZEB1, CBFA2T3, PDCD4, LGR4, PRMT2, PEX2, WWC3, RTF1, MKX, PLCB1, MYC, IRAK2, TBL1XR1, TP53, LEF1, SPEN, PURB, NRIP2, MXD4, ACVR2B, CCND1, MDM4, CUX1, NSD1, CLOCK, KMT2D, FRK, ZMYND11, CALCA, XBP1, POU2F1, PER2, GATAD2B, BCL6, NFATC4, BAZ2A, CREB1, TNP1, SIRT6, SNAI1, SIRT1, TMPRSS6, TRIM21, TP73, DKK1, PHF19, HEYL, IRF1, ZBTB4 | 1.494221364 | 0.063887137 | 5.12E-04 | 0.01719531 |
| GO:0051924~regulation of calcium ion transport | 36 | 2.639296188 | 9.27E-06 | CAV3, JPH3, GNAI2, DRD2, CCR1, LHCGR, SNCA, CACNB1, CACNB3, FKBP1A, CXCL11, FKBP1B, CXCL10, CALCA, ACE, BEST1, ATG5, ANK2, BCL2, PDE4B, SERPINE1, MS4A2, GNAO1, NOS1, NOS1AP, ATP1A2, PRKCE, PRKD1, CRHR1, PLCG1, PLN, PLA2G6, PDGFRB, AKAP6, STC1, GLP1R | 2.253776613 | 0.068291241 | 5.44E-04 | 0.018423463 |
| GO:0060429~epithelium development | 116 | 8.504398827 | 9.29E-06 | PGF, JAG1, SIDT2, CALB1, VCL, HS2ST1, S1PR2, WNT1, S1PR3, PLOD1, GATA3, WDR77, GAB1, RARB, FNDC3B, SATB1, RET, ROCK1, SIX3, DLL1, GRHL2, CRHR1, VSIG1, TAGLN, MCIDAS, VEGFA, STC1, FZR1, PPP2R3A, HOXA13, TFCP2L1, ONECUT2, DAG1, SOX4, SRC, PPP1R16B, FOXQ1, OVOL2, OVOL1, AXIN2, TMEM79, MUC2, MAP2K1, PODXL, MET, NTN4, SMAD4, SKI, GAS1, HGF, FOXP1, FOXP2, NOTCH2, EPHA4, NOTCH1, HDAC1, TRPS1, ZIC5, AREG, NCOR2, KLF4, ARID4A, MITF, ARID4B, PPARG, ZEB1, LGR4, HPSE, PAX7, SEMA3E, DLG3, MYC, AGAP2, TP53, LEF1, PRKCH, CDK6, EDAR, MYADM, MYCN, ACVR2B, CCND1, CLIC5, WNT9B, SEMA4C, JMJD1C, CUX1, CLOCK, PPP3R1, KITLG, CTNND1, BCCIP, KIT, XBP1, FAT4, BCL2, BCL11B, TOR1A, POU2F1, SLC4A7, NFATC4, SCNN1G, MLLT3, CAPN5, PLET1, SLC12A2, CREB1, GRSF1, SNAI1, WNT2B, DKK1, PRLR, HEYL, TMTC3, POFUT1, LRP4 | 1.497620647 | 0.068416639 | 5.41E-04 | 0.018458517 |
| GO:0010720~positive regulation of cell development | 68 | 4.985337243 | 9.32E-06 | OPRM1, ARSB, SYT1, GFAP, CPEB3, SYNJ1, PPARG, ZEB1, FES, S1PR2, BRINP1, MAPT, EEF2K, RARB, MYC, LRRC7, RAB21, DISC1, DAB2IP, RET, LEF1, PRKCH, SPEN, MYADM, NUMBL, PRKD1, NCOA1, FOXG1, VEGFA, TRIM32, AKAP6, TGIF2, RELN, CUX1, GCNT2, DRD2, CRTC1, KIT, LIN28A, FKBP1B, EPHB2, ACE, TRIM67, BCL2, BCL6, BCL9L, NEDD4L, AXIN2, PLAG1, DIXDC1, PTPRD, MAP2K1, MET, SMAD4, HGF, SNAI1, SIRT1, TP73, SEMA6A, EPHA4, NOTCH1, HDAC1, HEYL, RGS6, NEUROD2, KDM4C, MARCKS, TCF12 | 1.741895936 | 0.068673866 | 5.39E-04 | 0.018530437 |
| GO:0046578~regulation of Ras protein signal transduction | 31 | 2.272727273 | 9.46E-06 | RALGPS2, RALGPS1, CYTH4, SHOC2, KITLG, LPAR2, ITSN1, PLEKHG2, TRIM67, RASGRP4, BCL6, RASA4, FGD6, FGD3, ALS2CL, ARHGEF3, ARHGEF33, DAB2IP, ABR, STMN3, MAP2K1, PSD3, ARPP19, ARHGEF9, DGKI, FARP2, MFN2, NOTCH2, GPR55, PDGFRB, DGKZ | 2.433808018 | 0.069621231 | 5.42E-04 | 0.01879549 |
| GO:0001822~kidney development | 43 | 3.152492669 | 9.91E-06 | LZTS2, HNF1A, APH1A, PGF, CTNND1, SOX4, JAG1, LIN28A, CALB1, LGR4, HS2ST1, WNT1, AMER1, ACE, FAT4, BCL2, GATA3, OVOL1, RARB, MYC, COL4A4, RET, PODXL, SMAD4, LEF1, DLL1, TP73, WNT2B, CDKN1C, NOTCH3, EPHA4, ACVR2B, TNS2, NOTCH1, KCNJ8, HEYL, VEGFA, WNT9B, PDGFRA, PDGFRB, GFRA1, CUX1, LRP4 | 2.068034905 | 0.072842516 | 5.64E-04 | 0.019698756 |
| GO:0031327~negative regulation of cellular biosynthetic process | 140 | 10.26392962 | 9.94E-06 | CBX3, MAF1, NLRC5, PTGIS, CRY2, MYOCD, OLIG3, MDFIC, GATA3, RARB, TWIST2, SAMD4A, PID1, SATB1, RBFOX2, SATB2, DAB2IP, SOGA1, MTA2, STRN3, YY1, ZHX2, SIX3, PKIA, FOXN3, PPARGC1B, UHRF2, HNF4A, TRIM32, FOXG1, VEGFA, MNT, AKAP6, TGIF2, ATXN1L, TNRC18, STRAP, DRD2, TFCP2L1, NFKBIA, SRC, OVOL2, LPCAT1, CNOT6L, OVOL1, TFDP2, NAT10, ASXL2, ESRRA, MET, NR4A2, SMAD4, KLF17, SKI, WHSC1, FOXP3, TRIM62, FOXP1, ZBTB42, FOXP2, NOTCH3, CDKN1C, NOTCH2, DLX1, NOTCH1, NR1I2, HDAC1, NEDD4, TRPS1, ATF7, JAZF1, KLF4, NCOR2, CREBRF, OPRM1, HNF1A, ARID4A, CPEB2, CPEB3, MITF, PPARG, ZEB1, CBFA2T3, PDCD4, LGR4, PRMT2, PEX2, RTF1, WWC3, MKX, PLCB1, MYC, IRAK2, TBL1XR1, CYCS, TP53, LEF1, ERLIN1, SPEN, NRIP2, PURB, MXD4, INHBB, ACVR2B, CCND1, GRM7, MDM4, CUX1, NSD1, CLOCK, FRK, ZMYND11, KMT2D, CALCA, XBP1, POU2F1, PER2, GATAD2B, BCL6, NFATC4, INPP5D, AGO4, BAZ2A, CREB1, TNP1, SIRT6, SNAI1, TMPRSS6, SIRT1, TRIM21, TP73, PHF19, DKK1, RPL13A, CDAN1, OBFC1, HEYL, IRF1, NEUROD2, ZBTB4 | 1.432986148 | 0.073077377 | 5.62E-04 | 0.019764734 |
| GO:0032386~regulation of intracellular transport | 68 | 4.985337243 | 9.99E-06 | CREBRF, NCBP2, DPH6, XPO4, LHCGR, SNCA, TNFSF14, FES, CXCL11, CXCL10, TMEM173, ANK2, ATG5, MDFIC, ANK3, NMUR1, MAPT, RAB29, MS4A2, SAR1A, RAB21, DAB2IP, ANP32B, ZDHHC8, EDAR, PRKCE, CDKL2, PKIA, PRKD1, CRHR1, PRKCQ, HNF4A, AKAP6, ATPIF1, VAMP2, SUPT6H, ABLIM3, ATG13, PPM1A, PPP3R1, NFKBIA, SOX4, FKBP1A, SNX4, FKBP1B, SRC, CALCA, OAZ2, XBP1, BCL2, LYPLAL1, AXIN2, ELMOD1, PIK3R2, CNST, MYO1C, MAP2K1, SMAD4, LRRC46, PTPN14, GAS1, SIRT1, UACA, PLCG1, NEDD4, PLN, KCNN3, SVIP | 1.738829218 | 0.073387736 | 5.60E-04 | 0.019851948 |
| GO:1902589~single-organism organelle organization | 210 | 15.39589443 | 1.04E-05 | SYT1, DYNC1LI1, CHMP3, PDLIM7, SNCA, CHMP7, HOOK3, S1PR2, BRPF3, VPS4A, INA, DBNL, ROCK1, RAP1GDS1, MYH9, VTI1A, PPARGC1B, FARP2, LETM1, NAV1, SERPINF2, MCIDAS, VEGFA, PDGFRA, PDGFRB, ATPIF1, ADD2, GATC, SUPT6H, HOXA13, DAG1, DAAM1, RIMS3, NAA50, STX17, PVRL2, NAT10, TNKS, ASXL2, ABR, MAP2K1, SMAD4, MSTN, CDC23, KCNK3, FAM131B, TRPS1, TMOD2, MARCKS, RANBP10, GFAP, LIMA1, LZTS2, HNF1A, CRNKL1, WASF1, MAPT, SEMA3E, ANP32A, TGFA, CAP1, TUBB1, MYC, FMNL3, STX1A, POGZ, TP53, ZCCHC17, MYADM, ELMO1, ZDHHC15, ANKRD23, PLEKHH2, NSD1, CLOCK, SRGAP2, PARVA, PPFIA1, ATG13, BET1, HK1, EEA1, KIT, MPV17L2, XBP1, BCL2, PER2, BCL6, AGO4, BAZ2A, CAPN6, NOS1, SWAP70, LMNA, TNP1, MID1, RPS6KA4, PHF19, OBFC1, KDM8, MEX3C, SLC6A1, HBS1L, SLC2A8, SLC16A1, ATG5, MYOCD, FAM73B, GATA3, SAR1A, DISC1, PID1, SATB1, ATG9A, STMN3, MTA2, TRIM32, VAMP2, STMN1, CLIP3, MAP7D3, MAP6D1, CAV3, SNX9, DRD2, ONECUT2, ULK4, NR2C2, SRC, SLX4, GMIP, AXIN2, TBC1D30, CSF1R, TAF5, MET, ARPP19, WHSC1, WIPI2, FOXP3, FOXP1, CORO1C, MSL2, HDAC1, RHOJ, ARID4A, FAM175B, ARID4B, RHOQ, PEX3, PIP5K1A, FES, SPICE1, JADE2, PRMT2, RSPO1, PEX2, TRIM9, PAX7, RTF1, PLCB1, KDM5D, KMT5B, TBL1XR1, KDM7A, NRXN2, CHST3, PADI2, LEF1, PRKCE, ARHGAP26, VAT1, PRKCB, BBS1, PRKD1, MFN2, PRKCQ, NCOA1, XIRP1, ATG4B, TPPP, CNTN2, MAPRE2, TREM1, ABLIM1, KMT2D, ING3, ABLIM3, CTCFL, CALCOCO2, CALCB, MOAP1, TOR1A, SEC16A, SYNPO, PIK3R2, CHD3, DNM1L, MAP1A, SIRT6, SIRT1, CDC25A, ATXN7L3, GMFB, PPP1R9B, MYPN, ATXN7, KDM4C, IRF4, EVI5L | 1.322111407 | 0.076038531 | 5.77E-04 | 0.020598034 |
| GO:0007264~small GTPase mediated signal transduction | 60 | 4.398826979 | 1.04E-05 | RHOJ, WASF1, SHOC2, RHOQ, LPAR2, RASGEF1C, RHOV, ITSN1, RAB29, ARHGAP1, RRAS, AGAP2, RAB21, ALS2CL, DBNL, ARHGEF3, DAB2IP, STMN3, ROCK1, RAB4A, RAP1GDS1, PSD3, TP53, ARHGEF9, RASL12, FARP2, ELMO1, MFN2, GPR55, PDGFRB, RELN, FKBP11, RALGPS2, RAB3B, USP8, RALGPS1, CYTH4, KITLG, PLEKHG2, RAB43, TRIM67, RASGRP4, BCL6, RASA4, FGD6, FGD3, RHOBTB3, ARHGEF33, ABR, MAP2K1, DOCK9, ARPP19, DOCK8, DGKI, RALGDS, DOCK3, CDH13, NOTCH2, RAB36, DGKZ | 1.811767755 | 0.076332281 | 5.75E-04 | 0.020680844 |
| GO:0030900~forebrain development | 56 | 4.105571848 | 1.05E-05 | NRP2, CRNKL1, NDST1, SLC6A3, BCAN, ZEB1, UQCRQ, HOOK3, WNT1, CNTNAP2, RARB, PLCB1, MYC, DISC1, SATB2, DAB2IP, SIX3, LEF1, SOCS7, NUMBL, BBS1, INHBB, CRHR1, NCOA1, FOXG1, CNTN2, RELN, SRGAP2, DRD2, TH, ULK4, SRC, EPHB2, TAL2, FAT4, BCL11B, DCX, CSF1R, DIXDC1, GNAO1, CREB1, AK3, AXL, NR4A2, SKI, TP73, WNT2B, FOXP2, NOTCH3, PPP1R9B, DLX1, NOTCH1, DKK1, HDAC1, ZIC5, NCOR2 | 1.856993008 | 0.076686273 | 5.74E-04 | 0.020780671 |
| GO:1903507~negative regulation of nucleic acid-templated transcription | 119 | 8.724340176 | 1.06E-05 | CBX3, MAF1, NLRC5, PTGIS, CRY2, MYOCD, OLIG3, MDFIC, GATA3, RARB, TWIST2, RBFOX2, SATB1, SATB2, DAB2IP, MTA2, YY1, STRN3, ZHX2, SIX3, PKIA, PPARGC1B, FOXN3, UHRF2, HNF4A, FOXG1, VEGFA, MNT, TGIF2, ATXN1L, TNRC18, STRAP, TFCP2L1, NFKBIA, SRC, OVOL2, OVOL1, TFDP2, ASXL2, ESRRA, MET, NR4A2, SMAD4, KLF17, WHSC1, SKI, FOXP3, FOXP1, ZBTB42, FOXP2, CDKN1C, NOTCH3, NOTCH2, NOTCH1, DLX1, NR1I2, HDAC1, NEDD4, TRPS1, ATF7, JAZF1, NCOR2, KLF4, CREBRF, HNF1A, ARID4A, CPEB3, MITF, PPARG, ZEB1, CBFA2T3, PDCD4, LGR4, PRMT2, PEX2, WWC3, RTF1, MKX, PLCB1, MYC, IRAK2, TBL1XR1, TP53, LEF1, SPEN, PURB, NRIP2, MXD4, ACVR2B, CCND1, MDM4, CUX1, NSD1, CLOCK, KMT2D, FRK, ZMYND11, CALCA, XBP1, POU2F1, PER2, GATAD2B, BCL6, NFATC4, BAZ2A, CREB1, TNP1, SIRT6, SNAI1, SIRT1, TMPRSS6, TRIM21, TP73, DKK1, PHF19, HEYL, IRF1, NEUROD2, ZBTB4 | 1.484876497 | 0.07750339 | 5.76E-04 | 0.021011246 |
| GO:0022604~regulation of cell morphogenesis | 66 | 4.838709677 | 1.12E-05 | RHOJ, SYT1, SRCIN1, RHOQ, FES, MBP, ATG5, MAPT, CAMSAP1, TBC1D13, SEMA3E, EEF2K, RAB21, DISC1, DAB2IP, FMNL3, RET, LEF1, MYH9, MYADM, TBC1D22A, NUMBL, SEMA4F, VEGFA, CNTN2, WNT9B, SEMA4C, SEMA4B, RELN, CUX1, PARVA, ALDOA, GCNT2, STRAP, DAG1, KIT, SRC, EPHB2, OVOL2, PALM2, BCL9L, NFATC4, NEDD4L, AGO4, AXIN2, TBC1D30, TLX2, TBC1D2, CSF1R, DIXDC1, PTPRD, ZMYM4, MAP2K1, MET, SMAD4, TRIM62, SNAI1, CORO1C, SEMA6A, EPHA4, NOTCH1, NEDD4, CDC42SE2, MARCKS, EVI5L, LRP4 | 1.749281605 | 0.081817464 | 6.05E-04 | 0.022231986 |
| GO:0009890~negative regulation of biosynthetic process | 142 | 10.41055718 | 1.13E-05 | CBX3, MAF1, NLRC5, PTGIS, CRY2, MYOCD, OLIG3, MDFIC, GATA3, RARB, TWIST2, SAMD4A, PID1, SATB1, RBFOX2, SATB2, DAB2IP, SOGA1, MTA2, STRN3, YY1, ZHX2, SIX3, PKIA, FOXN3, PPARGC1B, UHRF2, HNF4A, TRIM32, FOXG1, VEGFA, MNT, AKAP6, TGIF2, ATXN1L, TNRC18, STRAP, DRD2, TFCP2L1, NFKBIA, SRC, OVOL2, LPCAT1, CNOT6L, OVOL1, TFDP2, NAT10, ASXL2, ESRRA, MET, NR4A2, SMAD4, KLF17, SKI, WHSC1, FOXP3, TRIM62, FOXP1, ZBTB42, FOXP2, NOTCH3, CDKN1C, NOTCH2, DLX1, NOTCH1, NR1I2, HDAC1, NEDD4, TRPS1, ATF7, JAZF1, KLF4, NCOR2, CREBRF, OPRM1, HNF1A, ARID4A, CPEB2, CPEB3, MITF, PPARG, ZEB1, CBFA2T3, PDCD4, LGR4, PRMT2, PEX2, RTF1, WWC3, MKX, PLCB1, MYC, IRAK2, TBL1XR1, CYCS, TP53, LEF1, ERLIN1, IL6R, SPEN, NRIP2, PURB, MXD4, INHBB, ACVR2B, CCND1, GRM7, MDM4, CUX1, NSD1, CLOCK, FRK, ZMYND11, KMT2D, CALCA, XBP1, POU2F1, PER2, GATAD2B, BCL6, NFATC4, INPP5D, AGO4, BAZ2A, CREB1, TNP1, MARCH8, SIRT6, SNAI1, TMPRSS6, SIRT1, TRIM21, TP73, PHF19, DKK1, RPL13A, CDAN1, OBFC1, HEYL, IRF1, NEUROD2, ZBTB4 | 1.42434808 | 0.08287702 | 6.09E-04 | 0.022532681 |
| GO:0001655~urogenital system development | 49 | 3.592375367 | 1.20E-05 | LZTS2, HNF1A, APH1A, HOXA13, PGF, SOX4, CTNND1, JAG1, LIN28A, CALB1, LGR4, EPHB2, HS2ST1, WNT1, AMER1, ACE, MYOCD, FAT4, BCL2, GATA3, OVOL1, WDR77, RARB, MYC, COL4A4, PLAG1, RET, PODXL, SMAD4, LEF1, DLL1, TP73, WNT2B, NOTCH3, CDKN1C, EPHA4, ACVR2B, NOTCH1, TNS2, PRLR, KCNJ8, VEGFA, HEYL, WNT9B, PDGFRA, PDGFRB, GFRA1, CUX1, LRP4 | 1.94451522 | 0.087788803 | 6.42E-04 | 0.023931151 |
| GO:0061564~axon development | 57 | 4.17888563 | 1.22E-05 | NRP2, VCL, MBP, SPRY3, GP5, UNC5A, XYLT1, ANK3, MAPT, GATA3, SEMA3E, LRRC55, LGI1, RAB21, DISC1, PTPRM, EFNB3, EFNB1, CHST3, COL25A1, NUMBL, SLITRK4, SEMA4F, FOXG1, VEGFA, TRIM32, CNTN2, SEMA4C, SEMA4B, RELN, STMN1, ABLIM1, DRD2, DAG1, FKBP1B, CALU, EPHB2, ALCAM, NPTX1, PVRL1, RNF165, BCL2, BCL11B, DCX, CSF1R, DIXDC1, MAP2K1, CREB1, SMAD4, NR4A2, GAS1, RAPH1, FOXP1, SEMA6A, EPHA4, NOTCH1, LRP4 | 1.835670235 | 0.088622829 | 6.44E-04 | 0.024169358 |
| GO:0019219~regulation of nucleobase-containing compound metabolic process | 292 | 21.40762463 | 1.25E-05 | LHCGR, RORA, WTAP, DDX17, CRY2, MED27, WDR77, CREB3L1, RARB, TWIST2, DAB2IP, STRN3, YY1, TAF4B, ZHX2, HNF4G, PPARGC1B, CRHR1, MED19, UHRF2, MTF1, HNF4A, SERPINF2, MCIDAS, VEGFA, FOXG1, MC2R, MNT, PDGFRA, PDGFRB, TGIF2, ATPIF1, ATXN1L, SUPT6H, TNRC18, HOXA13, PFKFB3, CRTC1, TFCP2L1, NFKBIA, MYT1, TAL2, PLAGL1, CDA, TNKS, NAT10, PLAG1, ASXL2, KLF6, BMP3, ESRRA, MAP2K1, SMAD4, PTPN14, KLF17, MSTN, SKI, HGF, UBP1, USF1, SFMBT2, ZBTB42, NOTCH3, NOTCH2, ZFHX4, DLX1, NOTCH1, NR1I2, CSRNP3, TRPS1, JAZF1, RBMXL1, AREG, TCF12, FOXI1, KLF4, NCOR2, BMP8B, OPRM1, CREBRF, HNF1A, CPEB3, PPARG, MITF, ZEB1, ANP32A, GUCY1A3, MKX, MYC, AGAP2, CSDC2, TP53, ERLIN1, SPEN, NFAM1, IL6R, FOSB, MXD4, MYCN, FOXR2, CCND1, GRM7, NSD1, CLOCK, ZMYND11, UNG, SCML2, KIT, XBP1, BCL11B, PER2, GATAD2B, MLLT1, BCL6, BCL9L, ZSCAN29, BAZ2A, TLX2, MLLT3, NOS1, LMNA, TNP1, AFF2, ZFP2, ATMIN, TMPRSS6, SNAI1, TP73, TRIM21, PREB, CDH13, RPS6KA4, PHF19, DKK1, PKNOX1, MAPK13, OBFC1, ZBTB4, KDM8, NEUROD2, TP53INP2, LRP4, HTR2A, TP53INP1, NCBP2, RNASEL, CBX3, JAG1, MAF1, CXCL11, CXCL10, NLRC5, WNT1, PTGIS, OLIG3, MYOCD, MDFIC, GATA3, TGS1, SAMD4A, PID1, SATB1, RBFOX2, RET, SATB2, MTA2, SIX3, FOXN2, FGF23, DLL1, GRHL2, PKIA, FOXN3, IGSF1, CELF6, TRIM32, CELF3, AKAP6, NFE2L1, RELN, GLP1R, CAMTA1, DRD2, STRAP, ONECUT2, SOX4, LIN28A, GREM2, SRC, NR2C2, SLX4, FOXQ1, ECE1, OVOL2, RNF165, OVOL1, TFDP2, RNF169, AXIN2, TAF5, MET, NR4A2, HACE1, WHSC1, RNPS1, FOXP3, TRIM62, FOXP1, FOXP2, CDKN1C, PPIE, HDAC1, NEDD4, GTF2F1, ZIC5, ATF7, POU6F1, E2F3, ARID4A, E2F5, ARID4B, RHOQ, CBFA2T3, PDCD4, LGR4, TMEM173, MAZ, PRMT2, PCBP4, PEX2, PAX7, RTF1, WWC3, LTF, LRRFIP1, PLCB1, FOSL1, KMT5B, IRAK2, TBL1XR1, KDM7A, CCDC88A, FOXJ2, PADI2, PRKCH, LEF1, DDN, PURB, NRIP2, PRKCB, PRKD1, PRKCQ, ACVR2B, NCOA1, MYRF, MDM4, JMJD1C, CUX1, CALCR, ABLIM1, FRK, KMT2D, ING3, ABLIM3, CTNND2, PPP3R1, PPM1A, CTCFL, KITLG, CALCA, RGMB, POU2F1, CHD1, NFATC4, TNRC6B, CHD3, PIK3R2, DNM1L, CREB1, CREB5, SIRT6, SIRT1, ATXN7L3, SP2, CDAN1, ATXN7, HEYL, IRF1, KDM4C, IRF4, IGFBP3, RNF41 | 1.249221428 | 0.091288668 | 6.60E-04 | 0.024932213 |
| GO:0040012~regulation of locomotion | 88 | 6.451612903 | 1.26E-05 | ARSB, SRCIN1, SNCA, TNFSF14, FES, CXCL11, AMOTL1, CXCL10, VCL, GATA3, SERPINE1, SEMA3E, GAB1, RRAS, PLCB1, MYC, MTUS1, RECK, DAB2IP, RET, PTPRM, MTA2, TP53, PTPRR, PADI2, LEF1, DAPK2, PRKCE, MYADM, IRS1, BBS1, PRKD1, SEMA4F, VEGFA, TRIM32, PDGFRA, SEMA4C, PDGFRB, SEMA4B, STC1, RELN, SRGAP2, GCNT2, PPP2R3A, DRD2, STRAP, CCR1, ONECUT2, DAG1, KITLG, ULK4, KIT, TRIM10, SRC, ACE, BCL2, MARVELD3, PVRL2, ARHGDIB, CSF1R, MUC2, PLET1, ABR, MYO1C, MAP2K1, SWAP70, PODXL, MET, IL1RN, LMNA, HACE1, HGF, FUCA2, TRIM62, SNAI1, FOXP1, TRIM21, CORO1C, CDH13, SEMA6A, NOTCH1, PLCG1, CXCL16, IGFBP3, HTR2C, KLF4, RNF41, TP53INP1 | 1.597677199 | 0.091701466 | 6.59E-04 | 0.025050539 |
| GO:0070838~divalent metal ion transport | 53 | 3.885630499 | 1.28E-05 | CALHM1, OPRM1, JPH3, TUSC3, LHCGR, SNCA, CXCL11, CXCL10, ATG5, ANK2, NMUR1, PDE4B, SERPINE1, MS4A2, NOS1AP, CACNG4, CACNG2, PRKCE, PRKCB, PRKD1, CRHR1, BSPRY, MAGT1, GRM7, PDGFRB, PLA2G6, AKAP6, STC1, GLP1R, CAV3, DRD2, CCR1, CACNB1, CACNB3, FKBP1A, FKBP1B, TPCN2, ORAI3, CALCA, ACE, BCL2, SLC39A9, SLC30A3, SLC30A6, GNAO1, NOS1, ATP1A2, CACNA2D2, PLCG1, PLN, CACNA1E, HTR2C, HTR2A | 1.882126951 | 0.093285587 | 6.66E-04 | 0.025505114 |
| GO:0007611~learning or memory | 39 | 2.859237537 | 1.30E-05 | JPH3, SLC6A1, CPEB3, DRD2, CRTC1, SYNJ1, TH, CTNND2, KIT, CALB1, EPHB2, NETO1, AP1S2, CHST10, MAPT, CNTNAP2, PLCB1, FOSL1, B4GALT2, NRXN2, CREB1, IL1RN, RCAN1, AFF2, ATP1A2, DGKI, FOXP2, GMFB, CRHR1, PPP1R9B, GRM7, CNTN2, TMOD2, NEUROD2, PLA2G6, CACNA1E, RELN, GLP1R, HTR2A | 2.137544108 | 0.094421673 | 6.70E-04 | 0.02583161 |
| GO:0071345~cellular response to cytokine stimulus | 67 | 4.91202346 | 1.34E-05 | CREBRF, CRNKL1, IL21R, PPARG, CXCR1, RORA, CXCL11, CXCL10, NLRC5, TMEM173, PTGIS, CDIP1, GATA3, SERPINE1, GAB1, PLCB1, MYC, IRAK2, PID1, DAB2IP, GBP5, HYAL3, YY1, ADIPOR2, FGF23, LEF1, PADI2, SOCS4, IL6R, ACKR4, NUMBL, INHBB, RELT, UBE2K, TRIM32, PDGFRA, AKAP6, CPD, CLDN18, RTN4RL1, CCR1, NFKBIA, FKBP1A, KIT, GREM2, CALCA, IRAK4, CCL22, ACSL1, XBP1, IL10RB, SELPLG, CSF1R, THPO, KLF6, MUC2, IL2RB, MET, IL1RN, SMAD4, AXL, SIRT1, RPS6KA4, PRLR, HDAC1, RPL13A, IRF1 | 1.731549213 | 0.097500515 | 6.88E-04 | 0.026718488 |
| GO:0051962~positive regulation of nervous system development | 64 | 4.692082111 | 1.35E-05 | OPRM1, ARSB, SYT1, GFAP, CPEB3, SYNJ1, PPARG, ZEB1, FES, BRINP1, MAPT, EEF2K, RARB, MYC, LRRC7, RAB21, DISC1, DAB2IP, RET, PRKCH, SPEN, NUMBL, PRKD1, NCOA1, SLITRK4, FOXG1, TRIM32, VEGFA, MYRF, RELN, TGIF2, CUX1, DRD2, CRTC1, KIT, LIN28A, FKBP1B, EPHB2, ACE, TRIM67, BCL2, LRRTM2, BCL6, NEDD4L, PLAG1, DIXDC1, PTPRD, MAP2K1, MET, ASIC2, HGF, SIRT1, TP73, SEMA6A, EPHA4, NOTCH1, HDAC1, HEYL, RGS6, NEUROD2, KDM4C, MARCKS, ADGRL1, TCF12 | 1.757197813 | 0.097729374 | 6.85E-04 | 0.026784532 |
| GO:0072511~divalent inorganic cation transport | 53 | 3.885630499 | 1.47E-05 | CALHM1, OPRM1, JPH3, TUSC3, LHCGR, SNCA, CXCL11, CXCL10, ATG5, ANK2, NMUR1, PDE4B, SERPINE1, MS4A2, NOS1AP, CACNG4, CACNG2, PRKCE, PRKCB, PRKD1, CRHR1, BSPRY, MAGT1, GRM7, PDGFRB, PLA2G6, AKAP6, STC1, GLP1R, CAV3, DRD2, CCR1, CACNB1, CACNB3, FKBP1A, FKBP1B, TPCN2, ORAI3, CALCA, ACE, BCL2, SLC39A9, SLC30A3, SLC30A6, GNAO1, NOS1, ATP1A2, CACNA2D2, PLCG1, PLN, CACNA1E, HTR2C, HTR2A | 1.872968183 | 0.106064918 | 7.42E-04 | 0.029201474 |
| GO:0051247~positive regulation of protein metabolic process | 141 | 10.3372434 | 1.50E-05 | SRCIN1, PPP2R5A, SNCA, LPAR2, S1PR2, WNT1, MDFIC, GATA3, GAB1, DISC1, SAMD4A, RET, DAB2IP, FGF23, SOCS4, MYH9, PPARGC1B, GPR55, HNF4A, RELT, SERPINF2, TRIM32, VEGFA, PDGFRA, PDGFRB, AKAP6, ATPIF1, RELN, CLIP3, NEK5, SNX9, FZR1, GCNT2, PPP2R3A, GNAI2, DRD2, SOX4, NFKBIA, LIN28A, SRC, OAZ2, PPP1R16B, AMER1, ACE, TRIM67, LPCAT1, AXIN2, CSF1R, HIP1, ASXL2, DIXDC1, BMP3, UNC119, MAP2K1, IL1RN, MET, SMAD4, MSTN, HGF, OXSR1, FOXP3, HSPBP1, EPHA4, UACA, HDAC1, AKTIP, NEDD4, DNAJB2, MARCKS, KLF4, BMP8B, NCOR2, CREBRF, OPRM1, CPEB3, PPARG, CBFA2T3, RSPO1, PAX7, RTF1, LTF, TGFA, PLCB1, AGAP2, MYC, IRAK2, CCDC88A, ANP32B, NOS1AP, CYCS, TP53, IL6R, PRKCE, PRKD1, INHBB, PRKCQ, ACVR2B, CCND1, KSR2, MAP3K15, PSME1, ATG4B, UBE2K, CNTN2, SEMA4C, PLA2G6, PSME3, KSR1, CCR1, ATG13, CTNND1, KITLG, FKBP1A, KIT, CALCA, CCL22, ACSL1, MPV17L2, XBP1, BCL2, BCL6, NEDD4L, EHD4, THPO, NOS1, MID1, SIRT1, BRAT1, TP73, SGTA, RPS6KA4, PHF19, PRLR, IRF1, SMPD1, SVIP, IRF4, IGFBP3, HTR2C, LRP4, HTR2A | 1.420202276 | 0.108254491 | 7.53E-04 | 0.029840083 |
| GO:0048667~cell morphogenesis involved in neuron differentiation | 63 | 4.618768328 | 1.52E-05 | NRP2, SRCIN1, VCL, MBP, GP5, ANK3, UNC5A, MAPT, GATA3, SEMA3E, EEF2K, LRRC55, LGI1, RAB21, DISC1, RBFOX2, PTPRM, EFNB3, EFNB1, COL25A1, NUMBL, BBS1, SLITRK4, SEMA4F, CLIC5, FOXG1, VEGFA, CNTN2, SEMA4C, SEMA4B, RELN, STMN1, CUX1, ABLIM1, DRD2, DAG1, CTNND2, EPHB2, ALCAM, NPTX1, PVRL1, RNF165, BCL2, BCL11B, NFATC4, DCX, TLX2, CSF1R, DIXDC1, PTPRD, MAP2K1, CREB1, MET, SMAD4, NR4A2, GAS1, RAPH1, FOXP1, SEMA6A, EPHA4, NOTCH1, MARCKS, LRP4 | 1.759679432 | 0.109506513 | 7.58E-04 | 0.030205949 |
| GO:0051253~negative regulation of RNA metabolic process | 123 | 9.017595308 | 1.62E-05 | CBX3, MAF1, NLRC5, PTGIS, CRY2, MYOCD, OLIG3, MDFIC, GATA3, RARB, TWIST2, RBFOX2, SATB1, SATB2, DAB2IP, MTA2, YY1, STRN3, ZHX2, SIX3, PKIA, PPARGC1B, FOXN3, UHRF2, HNF4A, TRIM32, FOXG1, VEGFA, MNT, TGIF2, ATXN1L, TNRC18, STRAP, TFCP2L1, NFKBIA, SRC, OVOL2, OVOL1, TFDP2, ASXL2, ESRRA, MET, NR4A2, SMAD4, KLF17, SKI, RNPS1, WHSC1, FOXP3, TRIM62, FOXP1, ZBTB42, FOXP2, CDKN1C, NOTCH3, NOTCH2, NOTCH1, DLX1, NR1I2, HDAC1, NEDD4, TRPS1, ATF7, JAZF1, NCOR2, KLF4, CREBRF, HNF1A, ARID4A, CPEB3, MITF, PPARG, ZEB1, CBFA2T3, PDCD4, LGR4, PRMT2, PEX2, PCBP4, WWC3, RTF1, MKX, PLCB1, MYC, IRAK2, TBL1XR1, TP53, LEF1, SPEN, PURB, NRIP2, MXD4, ACVR2B, CCND1, MDM4, CUX1, NSD1, CLOCK, KMT2D, FRK, ZMYND11, CALCA, XBP1, POU2F1, PER2, GATAD2B, BCL6, NFATC4, BAZ2A, CREB1, TNP1, SIRT6, SNAI1, SIRT1, TMPRSS6, TRIM21, TP73, PHF19, DKK1, HEYL, IRF1, NEUROD2, ZBTB4 | 1.460747012 | 0.116263001 | 8.02E-04 | 0.032189231 |
| GO:0045934~negative regulation of nucleobase-containing compound metabolic process | 134 | 9.824046921 | 1.64E-05 | CBX3, MAF1, NLRC5, PTGIS, CRY2, MYOCD, OLIG3, MDFIC, GATA3, RARB, TWIST2, PID1, SATB1, RBFOX2, SATB2, DAB2IP, MTA2, STRN3, YY1, ZHX2, SIX3, PKIA, FOXN3, PPARGC1B, UHRF2, HNF4A, TRIM32, FOXG1, VEGFA, MNT, AKAP6, TGIF2, ATXN1L, TNRC18, STRAP, DRD2, TFCP2L1, NFKBIA, SRC, OVOL2, OVOL1, TFDP2, CDA, RNF169, NAT10, TNKS, ASXL2, ESRRA, MET, NR4A2, SMAD4, KLF17, SKI, RNPS1, WHSC1, FOXP3, TRIM62, FOXP1, ZBTB42, FOXP2, NOTCH3, CDKN1C, NOTCH2, NOTCH1, DLX1, NR1I2, HDAC1, NEDD4, TRPS1, ATF7, JAZF1, KLF4, NCOR2, CREBRF, OPRM1, HNF1A, ARID4A, CPEB3, MITF, PPARG, ZEB1, CBFA2T3, PDCD4, LGR4, PRMT2, PEX2, PCBP4, RTF1, WWC3, MKX, PLCB1, MYC, IRAK2, TBL1XR1, TP53, LEF1, SPEN, NRIP2, PURB, MXD4, ACVR2B, CCND1, GRM7, MDM4, CUX1, NSD1, CLOCK, FRK, ZMYND11, KMT2D, CALCA, XBP1, POU2F1, PER2, GATAD2B, BCL6, NFATC4, BAZ2A, CREB1, TNP1, SIRT6, SNAI1, TMPRSS6, SIRT1, TRIM21, TP73, PHF19, DKK1, CDAN1, OBFC1, HEYL, IRF1, NEUROD2, ZBTB4 | 1.433182117 | 0.117685587 | 8.07E-04 | 0.03260874 |
| GO:0016568~chromatin modification | 64 | 4.692082111 | 1.70E-05 | HNF1A, ARID4A, SNCA, ARID4B, JADE2, PRMT2, BRPF3, MYOCD, ATG5, GATA3, PAX7, RTF1, ANP32A, ACTR8, MYC, KDM5D, KMT5B, TBL1XR1, KDM7A, SATB1, SATB2, MTA2, TP53, LEF1, PADI2, PRKCB, PRKD1, NCOA1, UHRF2, VEGFA, JMJD1C, NSD1, CLOCK, SUPT6H, KMT2D, ING3, TNRC18, CTCFL, NAA50, XBP1, PER2, GATAD2B, CHD1, BCL6, BAZ2A, ASXL2, NOS1, TAF5, SMAD4, TNP1, WHSC1, SIRT6, FOXP3, SIRT1, ATXN7L3, MSL2, RPS6KA4, PHF19, HDAC1, TRPS1, ATXN7, KDM8, KDM4C, IRF4 | 1.744010587 | 0.121885794 | 8.33E-04 | 0.033851295 |
| GO:0032844~regulation of homeostatic process | 58 | 4.252199413 | 1.71E-05 | JPH3, ADCY5, LHCGR, SNCA, RORA, SIDT2, CXCL11, CXCL10, ATG5, ANK2, ANK3, MS4A2, MYC, RBFOX2, SCN2B, CFTR, CDK6, DLL1, PRKCE, PPARGC1B, PRKD1, PRKCQ, MAP4K4, HNF4A, UBE2K, VEGFA, AKAP6, PLA2G6, GLP1R, CALCR, CAV3, CLDN18, DRD2, KITLG, FKBP1A, MYT1, FKBP1B, SRC, TPCN2, CALCA, SLX4, BCL2, NAT10, TNKS, SLC30A3, INPP5D, NEDD4L, SLC30A6, CSF1R, SIRT1, PLCG1, RPL13A, OBFC1, PLN, CACNA1E, HTR2C, BMP8B, HTR2A | 1.803879259 | 0.12226405 | 8.30E-04 | 0.033963486 |
| GO:0060627~regulation of vesicle-mediated transport | 54 | 3.958944282 | 1.72E-05 | SYT1, CHMP3, SYNJ1, PPARG, SNCA, SYT9, VPS37B, FES, CD47, RSPO1, ATG5, TRIM9, SERPINE1, EEF2K, VPS4A, MS4A2, LGI3, PLCB1, SAR1A, RAB21, STX1A, RAB4A, DLL1, CFTR, PCLO, CRHR1, VEGFA, PLA2G6, CLIP3, VAMP2, EPN2, CAV3, RAB3B, RAP1GAP, DRD2, SNX4, SRC, RIMS4, RIMS3, LRRTM2, TOR1A, LYPLAL1, NEDD4L, HIP1, EHD4, UNC119, ABR, CNST, MAP2K1, AXL, GAS1, CDH13, NOTCH1, DKK1 | 1.849797786 | 0.122774458 | 8.29E-04 | 0.03411495 |
| GO:0010558~negative regulation of macromolecule biosynthetic process | 134 | 9.824046921 | 1.77E-05 | CBX3, MAF1, NLRC5, PTGIS, CRY2, MYOCD, OLIG3, MDFIC, GATA3, RARB, TWIST2, SAMD4A, PID1, SATB1, RBFOX2, SATB2, DAB2IP, MTA2, STRN3, YY1, ZHX2, SIX3, PKIA, FOXN3, PPARGC1B, UHRF2, HNF4A, TRIM32, FOXG1, VEGFA, MNT, TGIF2, ATXN1L, TNRC18, STRAP, TFCP2L1, NFKBIA, SRC, OVOL2, CNOT6L, OVOL1, TFDP2, NAT10, ASXL2, ESRRA, MET, NR4A2, SMAD4, KLF17, SKI, WHSC1, FOXP3, TRIM62, FOXP1, ZBTB42, FOXP2, NOTCH3, CDKN1C, NOTCH2, NOTCH1, DLX1, NR1I2, HDAC1, NEDD4, TRPS1, ATF7, JAZF1, KLF4, NCOR2, CREBRF, HNF1A, ARID4A, CPEB2, CPEB3, MITF, PPARG, ZEB1, CBFA2T3, PDCD4, LGR4, PRMT2, PEX2, WWC3, RTF1, MKX, PLCB1, MYC, IRAK2, TBL1XR1, TP53, LEF1, IL6R, SPEN, NRIP2, PURB, MXD4, INHBB, ACVR2B, CCND1, MDM4, CUX1, NSD1, CLOCK, FRK, ZMYND11, KMT2D, CALCA, XBP1, POU2F1, PER2, GATAD2B, BCL6, NFATC4, INPP5D, AGO4, BAZ2A, CREB1, TNP1, SIRT6, MARCH8, SNAI1, TMPRSS6, SIRT1, TRIM21, TP73, PHF19, DKK1, RPL13A, CDAN1, OBFC1, HEYL, IRF1, NEUROD2, ZBTB4 | 1.430023009 | 0.126236723 | 8.48E-04 | 0.035144705 |
| GO:0051246~regulation of protein metabolic process | 224 | 16.42228739 | 1.84E-05 | DYNC1LI1, PPP2R5A, SNCA, LPAR2, S1PR2, CRY2, GAB1, SERPINE1, RECK, DAB2IP, ROCK1, PTPRR, MYH9, PPARGC1B, OGFOD1, HNF4A, SERPINF2, VEGFA, PDGFRA, PDGFRB, ATPIF1, WFDC5, NEK5, GATC, SUPT6H, RAD23B, GCNT2, PPP2R3A, GNAI2, DAG1, NFKBIA, BDKRB2, ACE, LPCAT1, HIP1, ASXL2, BMP3, UNC119, MAP2K1, IL1RN, SMAD4, MSTN, CDC23, HGF, OXSR1, GAS1, EPHA4, CSRNP3, TRPS1, MARCKS, KLF4, NCOR2, BMP8B, OPRM1, CREBRF, CPEB2, CPEB3, PPARG, ZYG11B, CCNE2, ANP32A, EEF2K, TGFA, AGAP2, MYC, ANP32B, FLOT2, TP53, IL6R, MYADM, INHBB, CCND1, MAP3K15, KSR2, SERPINB8, SEMA4C, SERPINB2, WNT9B, PLA2G6, KSR1, NSD1, ZER1, ZMYND11, CCR1, ATG13, BCCIP, FKBP1A, KIT, FKBP1B, SF3B3, CCL22, ACSL1, MPV17L2, XBP1, BCL2, PER2, MLLT1, BCL6, AGO4, NOS1, CNST, SWAP70, PPP1R11, MID1, BRAT1, SGTA, TRIM21, TP73, DUSP4, RPS6KA4, PHF19, DKK1, SVIP, HTR2C, LRP4, HTR2A, NCBP2, SRCIN1, PSTK, CTDSPL, TNFSF14, WNT1, ATG5, MYOCD, MDFIC, GATA3, RRAS, CTDSP2, SPRED1, RNF34, DISC1, SAMD4A, PID1, RET, FGF23, SOCS4, PKIA, MAP4K4, GPR55, RELT, TRIM32, AKAP6, RELN, CLIP3, CAV3, SNX9, FZR1, STRAP, DRD2, SOX4, ULK4, LIN28A, SRC, OAZ2, PPP1R16B, AMER1, TRIM67, ECE1, CNOT6L, LMTK3, AXIN2, CSF1R, DIXDC1, MET, ARPP19, FOXP3, CORO1C, CDKN1C, HSPBP1, UACA, HDAC1, AKTIP, NEDD4, DNAJB2, CBFA2T3, PDCD4, RSPO1, KLHL25, PAX7, RTF1, LTF, DLG3, PLCB1, IRAK2, ANKS1A, CCDC88A, NOS1AP, CYCS, RIMBP2, LEF1, PRKCE, PURB, PRKD1, PRKCQ, ACVR2B, PSME1, ATG4B, UBE2K, CNTN2, PSME3, MDM4, RTN4RL1, CTCFL, KITLG, CTNND1, CALCA, MARVELD3, INPP5D, NEDD4L, INPP5B, EHD4, THPO, FETUB, RCAN1, SIRT1, PPP1R9B, PRLR, RPL13A, SMPD1, IRF1, KDM4C, IRF4, IGFBP3, RNF41 | 1.296195917 | 0.130806493 | 8.76E-04 | 0.036510105 |
| GO:0051172~negative regulation of nitrogen compound metabolic process | 142 | 10.41055718 | 1.84E-05 | SNCA, CBX3, MAF1, NLRC5, PTGIS, CRY2, MYOCD, OLIG3, MDFIC, GATA3, RARB, TWIST2, SAMD4A, PID1, SATB1, RBFOX2, SATB2, DAB2IP, MTA2, STRN3, YY1, ZHX2, SIX3, PKIA, FOXN3, PPARGC1B, UHRF2, HNF4A, TRIM32, FOXG1, VEGFA, MNT, AKAP6, TGIF2, ATXN1L, TNRC18, STRAP, DRD2, TFCP2L1, NFKBIA, SRC, OVOL2, LPCAT1, CNOT6L, OVOL1, TFDP2, CDA, RNF169, NAT10, TNKS, ASXL2, ESRRA, MET, NR4A2, SMAD4, KLF17, SKI, RNPS1, WHSC1, FOXP3, TRIM62, FOXP1, ZBTB42, FOXP2, NOTCH3, CDKN1C, NOTCH2, DLX1, NOTCH1, NR1I2, HDAC1, NEDD4, TRPS1, ATF7, JAZF1, KLF4, NCOR2, CREBRF, OPRM1, HNF1A, CPEB2, ARID4A, CPEB3, MITF, PPARG, ZEB1, CBFA2T3, PDCD4, LGR4, PRMT2, PEX2, PCBP4, RTF1, WWC3, MKX, PLCB1, MYC, IRAK2, TBL1XR1, TP53, LEF1, SPEN, NRIP2, PURB, MXD4, ACVR2B, CCND1, GRM7, MDM4, CUX1, NSD1, CLOCK, FRK, ZMYND11, KMT2D, CALCA, XBP1, POU2F1, PER2, GATAD2B, BCL6, NFATC4, AGO4, BAZ2A, CREB1, TNP1, SIRT6, SNAI1, TMPRSS6, SIRT1, TRIM21, TP73, PHF19, DKK1, RPL13A, CDAN1, OBFC1, HEYL, IRF1, NEUROD2, ZBTB4, HTR2C | 1.410708632 | 0.130917665 | 8.71E-04 | 0.036543411 |
| GO:0042325~regulation of phosphorylation | 136 | 9.970674487 | 2.05E-05 | SRCIN1, CTDSPL, PPP2R5A, SNCA, LPAR2, ITSN1, S1PR2, NLRC5, WNT1, MYOCD, MDFIC, GAB1, RRAS, CTDSP2, SPRED1, PID1, DAB2IP, RET, PTPRR, FGF23, SOCS4, PKIA, IRS1, PPARGC1B, MAP4K4, GPR55, HNF4A, RELT, SERPINF2, VEGFA, PDGFRA, PDGFRB, AKAP6, RELN, CLIP3, CAV3, SNX9, GCNT2, GNAI2, STRAP, PFKFB3, DRD2, DAG1, BDKRB2, ULK4, SRC, ACE, ECE1, AXIN2, CSF1R, DIXDC1, UNC119, BMP3, MAP2K1, MET, IL1RN, SMAD4, MSTN, OXSR1, HGF, CORO1C, CDKN1C, EPHA4, HDAC1, AKTIP, MARCKS, AREG, KLF4, BMP8B, OPRM1, CBFA2T3, PDCD4, CCNE2, RSPO1, EEF2K, LTF, TGFA, PLCB1, AGAP2, MYC, IRAK2, CCDC88A, TP53, IL6R, PRKCE, MYADM, PRKD1, INHBB, ACVR2B, CCND1, KSR2, MAP3K15, UBE2K, WNT9B, SEMA4C, PLA2G6, KSR1, NSD1, ZMYND11, RTN4RL1, CCR1, ATG13, KITLG, CTNND1, BCCIP, FKBP1A, KIT, CALCA, CCL22, ACSL1, XBP1, BCL2, MARVELD3, MLLT1, EHD4, THPO, PIK3R2, NOS1, SIRT6, MID1, SIRT1, BRAT1, TP73, PPP1R9B, DUSP4, RPS6KA4, DKK1, PRLR, ATXN7, IRF1, SMPD1, IGFBP3, HTR2C, LRP4, RNF41, HTR2A | 1.420064696 | 0.144663784 | 9.64E-04 | 0.040694727 |
| GO:0010629~negative regulation of gene expression | 144 | 10.55718475 | 2.15E-05 | NCBP2, XPO5, CBX3, MAF1, NLRC5, PTGIS, CRY2, OLIG3, MYOCD, MDFIC, GATA3, SERPINE1, RARB, TWIST2, SAMD4A, SATB1, RBFOX2, SATB2, DAB2IP, MTA2, STRN3, YY1, SIX3, ZHX2, PKIA, FOXN3, PPARGC1B, UHRF2, UCN2, HNF4A, SERPINF2, TRIM32, FOXG1, VEGFA, MNT, TGIF2, ATXN1L, TNRC18, STRAP, TFCP2L1, NFKBIA, LIN28A, MYT1, SRC, ACE, OVOL2, ITGB8, CNOT6L, OVOL1, TFDP2, ASXL2, PLAG1, ESRRA, MAP2K1, MET, NR4A2, SMAD4, KLF17, SKI, RNPS1, WHSC1, GAS1, FOXP3, TRIM62, FOXP1, SFMBT2, ZBTB42, FOXP2, NOTCH3, CDKN1C, NOTCH2, DLX1, NOTCH1, NR1I2, HDAC1, NEDD4, TRPS1, ATF7, JAZF1, KLF4, NCOR2, CREBRF, HNF1A, CPEB2, ARID4A, CPEB3, MITF, PPARG, ZEB1, CBFA2T3, PDCD4, LGR4, PRMT2, PEX2, PCBP4, RTF1, WWC3, MKX, PLCB1, MYC, IRAK2, TBL1XR1, FLOT2, TP53, LEF1, SPEN, MYADM, NRIP2, PURB, MXD4, ACVR2B, CCND1, MDM4, CUX1, NSD1, CLOCK, FRK, ZMYND11, KMT2D, CCR1, CALCA, XBP1, POU2F1, PER2, GATAD2B, BCL6, NFATC4, AGO4, TNRC6B, BAZ2A, CREB1, TNP1, SIRT6, SNAI1, TMPRSS6, SIRT1, TRIM21, TP73, PHF19, DKK1, RPL13A, HEYL, IRF1, ZBTB4 | 1.402752982 | 0.151629939 | 0.001008316 | 0.042823975 |
| GO:0072001~renal system development | 44 | 3.225806452 | 2.18E-05 | LZTS2, HNF1A, APH1A, PGF, CTNND1, SOX4, JAG1, LIN28A, CALB1, LGR4, HS2ST1, WNT1, ACE, AMER1, MYOCD, FAT4, BCL2, GATA3, OVOL1, RARB, MYC, COL4A4, RET, PODXL, SMAD4, LEF1, DLL1, TP73, WNT2B, CDKN1C, NOTCH3, EPHA4, ACVR2B, TNS2, NOTCH1, KCNJ8, HEYL, VEGFA, WNT9B, PDGFRA, PDGFRB, GFRA1, CUX1, LRP4 | 1.984692173 | 0.153400377 | 0.001014896 | 0.0433679 |
| GO:0030334~regulation of cell migration | 78 | 5.718475073 | 2.22E-05 | ARSB, SRCIN1, TNFSF14, AMOTL1, CXCL11, CXCL10, VCL, GATA3, SERPINE1, SEMA3E, GAB1, RRAS, PLCB1, MYC, MTUS1, RECK, DAB2IP, RET, PTPRM, MTA2, TP53, PTPRR, LEF1, PADI2, DAPK2, PRKCE, MYADM, IRS1, PRKD1, SEMA4F, VEGFA, TRIM32, PDGFRA, SEMA4C, PDGFRB, SEMA4B, STC1, RELN, SRGAP2, GCNT2, DRD2, STRAP, CCR1, ONECUT2, DAG1, KITLG, ULK4, KIT, SRC, ACE, BCL2, MARVELD3, ARHGDIB, CSF1R, MUC2, PLET1, ABR, MYO1C, MAP2K1, SWAP70, PODXL, MET, IL1RN, LMNA, HACE1, HGF, SNAI1, FOXP1, CORO1C, CDH13, SEMA6A, NOTCH1, PLCG1, CXCL16, IGFBP3, KLF4, RNF41, TP53INP1 | 1.627727554 | 0.155916954 | 0.001026772 | 0.044143013 |
| GO:0006816~calcium ion transport | 48 | 3.519061584 | 2.22E-05 | CALHM1, OPRM1, CAV3, JPH3, DRD2, CCR1, LHCGR, SNCA, CACNB1, CACNB3, FKBP1A, CXCL11, FKBP1B, TPCN2, CXCL10, ORAI3, CALCA, ACE, ANK2, ATG5, NMUR1, BCL2, PDE4B, SERPINE1, MS4A2, GNAO1, NOS1, NOS1AP, CACNG4, CACNG2, ATP1A2, PRKCE, CACNA2D2, PRKCB, PRKD1, CRHR1, BSPRY, PLCG1, PLN, GRM7, PLA2G6, PDGFRB, AKAP6, CACNA1E, STC1, HTR2C, GLP1R, HTR2A | 1.915297341 | 0.15603014 | 0.001021397 | 0.044177929 |
| GO:2000145~regulation of cell motility | 81 | 5.938416422 | 2.26E-05 | ARSB, SRCIN1, TNFSF14, FES, CXCL11, AMOTL1, CXCL10, VCL, GATA3, SERPINE1, SEMA3E, GAB1, RRAS, PLCB1, MYC, MTUS1, RECK, DAB2IP, RET, PTPRM, MTA2, TP53, PTPRR, LEF1, PADI2, DAPK2, PRKCE, MYADM, IRS1, BBS1, PRKD1, SEMA4F, VEGFA, TRIM32, PDGFRA, SEMA4C, PDGFRB, SEMA4B, STC1, RELN, SRGAP2, GCNT2, PPP2R3A, DRD2, STRAP, CCR1, ONECUT2, DAG1, KITLG, ULK4, KIT, SRC, ACE, BCL2, MARVELD3, ARHGDIB, CSF1R, MUC2, PLET1, ABR, MYO1C, MAP2K1, SWAP70, PODXL, MET, IL1RN, LMNA, HACE1, HGF, SNAI1, FOXP1, CORO1C, CDH13, SEMA6A, NOTCH1, PLCG1, CXCL16, IGFBP3, KLF4, RNF41, TP53INP1 | 1.609399989 | 0.158409422 | 0.001032171 | 0.044912976 |
| GO:0007265~Ras protein signal transduction | 40 | 2.93255132 | 2.76E-05 | RHOJ, RALGPS2, USP8, RALGPS1, WASF1, CYTH4, SHOC2, KITLG, LPAR2, ITSN1, PLEKHG2, TRIM67, RASGRP4, BCL6, RASA4, FGD6, FGD3, ALS2CL, DBNL, ARHGEF3, DAB2IP, ARHGEF33, ABR, STMN3, ROCK1, MAP2K1, RAB4A, ARPP19, PSD3, TP53, ARHGEF9, DGKI, FARP2, ELMO1, MFN2, NOTCH2, CDH13, GPR55, PDGFRB, DGKZ | 2.045681433 | 0.189734867 | 0.00125156 | 0.054788668 |
| GO:0031401~positive regulation of protein modification process | 109 | 7.991202346 | 2.84E-05 | SRCIN1, PPP2R5A, SNCA, LPAR2, S1PR2, WNT1, MDFIC, GATA3, GAB1, DISC1, DAB2IP, RET, FGF23, PPARGC1B, HNF4A, RELT, GPR55, SERPINF2, VEGFA, PDGFRA, PDGFRB, AKAP6, RELN, CLIP3, SNX9, FZR1, GCNT2, GNAI2, DRD2, SOX4, SRC, PPP1R16B, AMER1, ACE, TRIM67, AXIN2, CSF1R, ASXL2, UNC119, BMP3, DIXDC1, MAP2K1, MET, IL1RN, SMAD4, MSTN, OXSR1, HGF, FOXP3, HSPBP1, EPHA4, HDAC1, AKTIP, DNAJB2, MARCKS, BMP8B, NCOR2, OPRM1, RSPO1, PAX7, RTF1, LTF, TGFA, PLCB1, AGAP2, IRAK2, CCDC88A, NOS1AP, TP53, IL6R, PRKCE, PRKD1, INHBB, ACVR2B, CCND1, MAP3K15, KSR2, UBE2K, SEMA4C, PLA2G6, KSR1, CCR1, ATG13, KITLG, CTNND1, FKBP1A, KIT, CALCA, CCL22, ACSL1, XBP1, BCL2, BCL6, THPO, EHD4, NOS1, MID1, SIRT1, BRAT1, TP73, RPS6KA4, PHF19, PRLR, SMPD1, SVIP, HTR2C, IGFBP3, LRP4, HTR2A | 1.482352866 | 0.194929989 | 0.001282172 | 0.056463225 |
| GO:0032268~regulation of cellular protein metabolic process | 210 | 15.39589443 | 2.96E-05 | DYNC1LI1, PPP2R5A, SNCA, LPAR2, S1PR2, CRY2, SERPINE1, GAB1, RECK, DAB2IP, ROCK1, PTPRR, MYH9, PPARGC1B, OGFOD1, HNF4A, SERPINF2, VEGFA, PDGFRA, PDGFRB, ATPIF1, WFDC5, NEK5, GATC, SUPT6H, RAD23B, GCNT2, GNAI2, DAG1, NFKBIA, BDKRB2, ACE, HIP1, ASXL2, BMP3, UNC119, MAP2K1, IL1RN, SMAD4, MSTN, CDC23, HGF, OXSR1, GAS1, EPHA4, CSRNP3, TRPS1, MARCKS, NCOR2, BMP8B, KLF4, OPRM1, CPEB2, CPEB3, PPARG, ZYG11B, CCNE2, EEF2K, ANP32A, TGFA, AGAP2, MYC, ANP32B, TP53, IL6R, MYADM, INHBB, CCND1, MAP3K15, KSR2, SERPINB8, SERPINB2, WNT9B, SEMA4C, PLA2G6, KSR1, NSD1, ZER1, ZMYND11, CCR1, ATG13, FKBP1A, BCCIP, KIT, FKBP1B, CCL22, ACSL1, MPV17L2, XBP1, BCL2, PER2, MLLT1, BCL6, AGO4, NOS1, CNST, SWAP70, PPP1R11, MID1, BRAT1, TRIM21, SGTA, TP73, DUSP4, RPS6KA4, PHF19, DKK1, SVIP, HTR2C, LRP4, HTR2A, NCBP2, SRCIN1, PSTK, CTDSPL, TNFSF14, WNT1, ATG5, MYOCD, MDFIC, GATA3, RRAS, CTDSP2, SPRED1, RNF34, DISC1, SAMD4A, PID1, RET, FGF23, SOCS4, PKIA, MAP4K4, GPR55, RELT, TRIM32, AKAP6, RELN, CLIP3, CAV3, SNX9, FZR1, STRAP, DRD2, SOX4, ULK4, LIN28A, SRC, PPP1R16B, AMER1, TRIM67, ECE1, CNOT6L, LMTK3, AXIN2, CSF1R, DIXDC1, MET, ARPP19, FOXP3, CORO1C, HSPBP1, CDKN1C, UACA, HDAC1, AKTIP, DNAJB2, CBFA2T3, PDCD4, RSPO1, KLHL25, PAX7, RTF1, LTF, DLG3, PLCB1, IRAK2, ANKS1A, CCDC88A, NOS1AP, CYCS, RIMBP2, LEF1, PRKCE, PURB, PRKD1, ACVR2B, PSME1, UBE2K, CNTN2, PSME3, RTN4RL1, CTCFL, KITLG, CTNND1, CALCA, MARVELD3, THPO, EHD4, FETUB, RCAN1, SIRT1, PPP1R9B, PRLR, RPL13A, SMPD1, IRF1, KDM4C, IGFBP3, RNF41 | 1.300132573 | 0.20233643 | 0.00132893 | 0.058869292 |
| GO:0032870~cellular response to hormone stimulus | 67 | 4.91202346 | 3.05E-05 | CREBRF, RNASEL, CPEB2, PGF, LHCGR, PPARG, NAP1L1, RHOQ, CBX3, RORA, SLC2A8, WNT1, PLOD1, SERPINE1, EEF2K, RARB, MYC, MTUS1, PID1, SOGA1, ADIPOR2, FGF23, LEF1, SOCS7, CFTR, FOSB, HNF4G, PRKCE, IRS1, PRKCB, CRHR1, INHBB, PRKCQ, NCOA1, UCN2, HNF4A, VEGFA, STC1, RELN, VAMP2, GLP1R, HMGCS1, PAQR7, PAQR8, KIT, SRC, NR2C2, GPR22, ACSL1, RPL32, XBP1, PIK3R2, ESRRA, CREB1, MET, NR4A2, MSTN, ATP1A2, USF1, SIRT1, PPP1R9B, NOTCH1, NR1I2, UCP3, PRLR, IRF1, MARCKS | 1.686534935 | 0.207531954 | 0.001359327 | 0.060570452 |
| GO:0010721~negative regulation of cell development | 45 | 3.299120235 | 3.07E-05 | CAV3, CLDN18, GFAP, RAP1GAP, STRAP, ADCY5, JAG1, LIN28A, HOOK3, EPHB2, MBP, S1PR3, FAT3, OVOL2, XYLT1, BCL2, RAB29, SEMA3E, ANP32A, NFATC4, TLX2, DIXDC1, DAB2IP, SIX3, TP53, ZHX2, DLL1, SKI, TRIM62, TP73, MYCN, CORO1C, NOTCH3, SEMA6A, EPHA4, MAP4K4, NOTCH1, DLX1, SEMA4F, FOXG1, CNTN2, SEMA4C, SEMA4B, LRP4, SRGAP2 | 1.939451685 | 0.209156768 | 0.001363346 | 0.061104746 |
| GO:0009896~positive regulation of catabolic process | 55 | 4.032258065 | 3.09E-05 | CREBRF, CRNKL1, SLC6A1, CPEB3, SNCA, PEX3, CBFA2T3, ATG5, MYC, DISC1, DAB2IP, CHST3, SOCS4, PRKCE, IRS1, ZCCHC17, ATG4B, TRIM32, ATPIF1, TREM1, SNX9, FZR1, PPP2R3A, ATG13, CALCOCO2, HK1, NR2C2, RIMS3, OAZ2, CALCB, AMER1, TRIM67, LPCAT1, GMIP, XBP1, PAFAH1B2, NEDD4L, TNRC6B, MAP2K1, MAP1A, MSTN, SIRT1, TRIM21, KCNK3, SGTA, FAM131B, HSPBP1, NEDD4, MEX3C, DNAJB2, SVIP, IGFBP3, TP53INP2, TP53INP1, HTR2A | 1.799186035 | 0.2101526 | 0.001362744 | 0.061432751 |
| GO:0009719~response to endogenous stimulus | 169 | 12.39002933 | 3.13E-05 | RNASEL, LDHA, SLC6A1, PGF, SLC6A3, SNCA, LHCGR, FGFRL1, CBX3, RORA, JAG1, SLC2A8, WNT1, CRY2, PLOD1, MYOCD, PAPPA, GATA3, PDE4B, SERPINE1, RARB, NQO1, MTUS1, PID1, DAB2IP, SOGA1, PDXK, STRN3, YY1, FGF23, SOCS7, HNF4G, UBR1, IRS1, PPARGC1B, CRHR1, UCN2, THBD, HNF4A, VEGFA, PDGFRA, PDGFRB, AKAP6, RELN, STC1, VAMP2, GLP1R, CAV3, ALDOA, GCNT2, DRD2, STRAP, ONECUT2, DAG1, HMGCS1, PAQR7, NFKBIA, PAQR8, RRAGD, GREM2, SRC, NR2C2, SLX4, PEA15, PEG10, GPR22, ACE, RNF165, IDH1, CDA, HCN3, KLF6, BMP3, MUC2, ESRRA, MAP2K1, IL1RN, MET, EME1, NR4A2, SMAD4, MSTN, SKI, USF1, CDKN1C, NOTCH2, NOTCH1, NR1I2, UCP3, HDAC1, PLCG1, MARCKS, AREG, KLF4, NCOR2, BMP8B, CREBRF, ARSB, OPRM1, CPEB2, CRNKL1, NDST1, CPEB3, GLRA3, PPARG, NAP1L1, RHOQ, ZEB1, MBP, GPX3, EEF2K, PLCB1, MYC, GPIHBP1, FOSL1, TP53, ADIPOR2, LEF1, CFTR, FOSB, IL6R, PRKCE, PRKCB, INHBB, PRKCQ, ACVR2B, NCOA1, CCND1, DGAT1, CALCR, KMT2D, FUT8, RAP1GAP, TH, PPM1A, FKBP1A, KIT, CALCA, RGMB, ACSL1, RPL32, XBP1, FAT4, BCL2, BCL9L, PIK3R2, SHMT1, NOS1, GNAO1, CREB1, RCAN1, ATP1A2, TMPRSS6, COL5A2, SIRT1, TP73, CDH13, PPP1R9B, DKK1, PRLR, RPL13A, PLN, HEYL, IRF1, SMPD1, DPYD, HTR2C, LRP4, HTR2A | 1.35017225 | 0.212519631 | 0.001372143 | 0.062214056 |
| GO:1901019~regulation of calcium ion transmembrane transporter activity | 16 | 1.173020528 | 3.21E-05 | CAV3, JPH3, NOS1AP, DRD2, CACNB1, CACNB3, FKBP1A, ATP1A2, FKBP1B, CRHR1, ANK2, PLCG1, PLN, PDE4B, AKAP6, PLA2G6 | 3.521051678 | 0.217155419 | 0.001398 | 0.063751037 |
| GO:0032940~secretion by cell | 96 | 7.038123167 | 3.26E-05 | OPRM1, SYT1, KCNC3, HNF1A, CHMP3, SRCIN1, VTCN1, ADCY5, SNCA, PPARG, SYNJ1, SYT9, ILDR2, ILDR1, FES, ITSN1, SIDT2, PNP, LGR4, SLC16A1, CRY2, ATG5, TRIM9, GATA3, VPS4A, CREB3L1, MS4A2, LGI3, PLCB1, RAB21, STX1A, GBP5, NOS1AP, NRXN2, FGF23, CFTR, PFKM, PRKCE, IRS1, PCLO, PRKCB, CRHR1, INHBB, PRKCQ, MAP4K4, ACVR2B, MYRIP, DGAT1, UCN2, HNF4A, LYST, GRM7, PLA2G6, TREM1, VAMP2, GLP1R, CLOCK, CPLX2, RAB3B, DRD2, KCNA2, CCR1, SOX4, SNX4, KIT, MYT1, FKBP1B, RIMS4, SRC, RIMS3, ARHGAP44, XBP1, GOLPH3L, SYN2, PER2, TMEM79, CSF1R, DNM1L, NOS1, ABR, CREB1, IL1RN, SMAD4, AXL, DGKI, FOXP3, SIRT1, FOXP1, NOTCH2, NOTCH1, HDAC1, PLCG1, CACNA1E, ADGRL1, HTR2C, HTR2A | 1.523864989 | 0.220514913 | 0.001414463 | 0.064870551 |
| GO:0006355~regulation of transcription, DNA-templated | 253 | 18.5483871 | 3.31E-05 | RORA, DDX17, CRY2, WDR77, MED27, CREB3L1, RARB, TWIST2, DAB2IP, STRN3, TAF4B, YY1, ZHX2, HNF4G, PPARGC1B, MED19, UHRF2, HNF4A, MTF1, SERPINF2, MCIDAS, VEGFA, FOXG1, MNT, TGIF2, ATXN1L, SUPT6H, TNRC18, HOXA13, CRTC1, TFCP2L1, NFKBIA, MYT1, TAL2, PLAGL1, TNKS, PLAG1, ASXL2, KLF6, BMP3, ESRRA, MAP2K1, SMAD4, KLF17, MSTN, SKI, UBP1, USF1, SFMBT2, ZBTB42, NOTCH3, NOTCH2, DLX1, NOTCH1, ZFHX4, NR1I2, CSRNP3, TRPS1, JAZF1, RBMXL1, TCF12, FOXI1, KLF4, NCOR2, BMP8B, CREBRF, HNF1A, CPEB3, PPARG, MITF, ZEB1, ANP32A, MKX, MYC, AGAP2, CSDC2, TP53, ERLIN1, SPEN, NFAM1, IL6R, FOSB, MYCN, FOXR2, MXD4, CCND1, NSD1, CLOCK, ZMYND11, SCML2, KIT, XBP1, BCL11B, PER2, GATAD2B, MLLT1, BCL6, BCL9L, ZSCAN29, BAZ2A, TLX2, MLLT3, NOS1, LMNA, TNP1, ZFP2, ATMIN, TMPRSS6, SNAI1, TRIM21, TP73, PREB, CDH13, RPS6KA4, PHF19, PKNOX1, DKK1, MAPK13, KDM8, ZBTB4, NEUROD2, TP53INP2, LRP4, TP53INP1, RNASEL, CBX3, JAG1, MAF1, NLRC5, WNT1, PTGIS, OLIG3, MYOCD, MDFIC, GATA3, TGS1, SAMD4A, PID1, SATB1, RBFOX2, RET, SATB2, MTA2, SIX3, FOXN2, FGF23, DLL1, GRHL2, PKIA, FOXN3, IGSF1, TRIM32, NFE2L1, RELN, GLP1R, CAMTA1, DRD2, STRAP, ONECUT2, SOX4, LIN28A, GREM2, SRC, NR2C2, FOXQ1, OVOL2, RNF165, OVOL1, TFDP2, AXIN2, TAF5, MET, NR4A2, HACE1, WHSC1, FOXP3, TRIM62, FOXP1, FOXP2, CDKN1C, PPIE, HDAC1, NEDD4, GTF2F1, ZIC5, ATF7, POU6F1, E2F3, ARID4A, E2F5, ARID4B, RHOQ, CBFA2T3, PDCD4, LGR4, TMEM173, MAZ, PRMT2, PEX2, PAX7, RTF1, WWC3, LTF, LRRFIP1, PLCB1, FOSL1, KMT5B, IRAK2, TBL1XR1, KDM7A, FOXJ2, PADI2, PRKCH, LEF1, DDN, PURB, NRIP2, PRKCB, PRKD1, PRKCQ, ACVR2B, NCOA1, MYRF, MDM4, JMJD1C, CUX1, ABLIM1, FRK, KMT2D, ING3, ABLIM3, CTNND2, PPP3R1, PPM1A, CTCFL, CALCA, RGMB, POU2F1, NFATC4, PIK3R2, CHD3, CREB1, CREB5, SIRT6, SIRT1, ATXN7L3, SP2, ATXN7, HEYL, IRF1, KDM4C, IRF4, RNF41 | 1.26017063 | 0.22333043 | 0.001426892 | 0.065812503 |
| GO:0032270~positive regulation of cellular protein metabolic process | 131 | 9.604105572 | 3.43E-05 | SRCIN1, PPP2R5A, SNCA, LPAR2, S1PR2, WNT1, MDFIC, GATA3, GAB1, DISC1, SAMD4A, RET, DAB2IP, FGF23, SOCS4, MYH9, PPARGC1B, GPR55, HNF4A, RELT, SERPINF2, TRIM32, VEGFA, PDGFRA, PDGFRB, AKAP6, RELN, ATPIF1, CLIP3, NEK5, SNX9, FZR1, GCNT2, GNAI2, DRD2, SOX4, NFKBIA, LIN28A, SRC, PPP1R16B, AMER1, ACE, TRIM67, AXIN2, CSF1R, HIP1, ASXL2, DIXDC1, BMP3, UNC119, MAP2K1, MET, IL1RN, SMAD4, MSTN, HGF, OXSR1, FOXP3, HSPBP1, EPHA4, UACA, HDAC1, AKTIP, DNAJB2, MARCKS, KLF4, BMP8B, NCOR2, OPRM1, CPEB3, PPARG, CBFA2T3, RSPO1, PAX7, RTF1, LTF, TGFA, PLCB1, AGAP2, MYC, IRAK2, CCDC88A, ANP32B, NOS1AP, CYCS, TP53, IL6R, PRKCE, PRKD1, INHBB, ACVR2B, CCND1, KSR2, MAP3K15, PSME1, UBE2K, CNTN2, SEMA4C, PLA2G6, PSME3, KSR1, CCR1, ATG13, KITLG, CTNND1, FKBP1A, KIT, CALCA, CCL22, ACSL1, MPV17L2, XBP1, BCL2, BCL6, EHD4, THPO, NOS1, MID1, SIRT1, BRAT1, TP73, SGTA, RPS6KA4, PHF19, PRLR, SMPD1, SVIP, IGFBP3, HTR2C, LRP4, HTR2A | 1.416744825 | 0.23007446 | 0.001467806 | 0.06808269 |
| GO:2001141~regulation of RNA biosynthetic process | 255 | 18.69501466 | 3.49E-05 | RORA, DDX17, CRY2, WDR77, MED27, CREB3L1, RARB, TWIST2, DAB2IP, STRN3, TAF4B, YY1, ZHX2, HNF4G, PPARGC1B, MED19, UHRF2, HNF4A, MTF1, SERPINF2, MCIDAS, VEGFA, FOXG1, MNT, TGIF2, ATXN1L, SUPT6H, TNRC18, HOXA13, CRTC1, TFCP2L1, NFKBIA, MYT1, TAL2, PLAGL1, TNKS, PLAG1, ASXL2, KLF6, BMP3, ESRRA, MAP2K1, SMAD4, PTPN14, KLF17, MSTN, SKI, UBP1, USF1, SFMBT2, ZBTB42, NOTCH3, NOTCH2, DLX1, NOTCH1, ZFHX4, NR1I2, CSRNP3, TRPS1, JAZF1, RBMXL1, TCF12, FOXI1, KLF4, NCOR2, BMP8B, CREBRF, HNF1A, CPEB3, PPARG, MITF, ZEB1, ANP32A, MKX, MYC, AGAP2, CSDC2, TP53, ERLIN1, SPEN, NFAM1, IL6R, FOSB, MYCN, FOXR2, MXD4, CCND1, NSD1, CLOCK, ZMYND11, SCML2, KIT, XBP1, BCL11B, PER2, GATAD2B, MLLT1, BCL6, BCL9L, ZSCAN29, BAZ2A, TLX2, MLLT3, NOS1, LMNA, TNP1, ZFP2, ATMIN, TMPRSS6, SNAI1, TRIM21, TP73, PREB, CDH13, RPS6KA4, PHF19, PKNOX1, DKK1, MAPK13, KDM8, ZBTB4, NEUROD2, TP53INP2, LRP4, TP53INP1, RNASEL, CBX3, JAG1, MAF1, NLRC5, WNT1, PTGIS, OLIG3, MYOCD, MDFIC, GATA3, TGS1, SAMD4A, PID1, SATB1, RBFOX2, RET, SATB2, MTA2, SIX3, FOXN2, FGF23, DLL1, GRHL2, PKIA, FOXN3, IGSF1, TRIM32, NFE2L1, RELN, GLP1R, CAMTA1, DRD2, STRAP, ONECUT2, SOX4, LIN28A, GREM2, SRC, NR2C2, FOXQ1, OVOL2, RNF165, OVOL1, TFDP2, AXIN2, TAF5, MET, NR4A2, HACE1, WHSC1, FOXP3, TRIM62, FOXP1, FOXP2, CDKN1C, PPIE, HDAC1, NEDD4, GTF2F1, ZIC5, ATF7, POU6F1, E2F3, ARID4A, E2F5, ARID4B, RHOQ, CBFA2T3, PDCD4, LGR4, TMEM173, MAZ, PRMT2, PEX2, PAX7, RTF1, WWC3, LTF, LRRFIP1, PLCB1, FOSL1, KMT5B, IRAK2, TBL1XR1, KDM7A, FOXJ2, PADI2, PRKCH, LEF1, DDN, PURB, NRIP2, PRKCB, PRKD1, PRKCQ, ACVR2B, NCOA1, MYRF, MDM4, JMJD1C, CUX1, ABLIM1, FRK, KMT2D, ING3, ABLIM3, CTNND2, PPP3R1, PPM1A, CTCFL, CALCA, RGMB, POU2F1, CHD1, NFATC4, PIK3R2, CHD3, CREB1, CREB5, SIRT6, SIRT1, ATXN7L3, SP2, ATXN7, HEYL, IRF1, KDM4C, IRF4, RNF41 | 1.257625207 | 0.233837344 | 0.001486942 | 0.069357995 |
| GO:0072359~circulatory system development | 104 | 7.624633431 | 3.50E-05 | PGF, FGFRL1, RORA, JAG1, CXCL10, DDX17, PTGIS, MYOCD, ATG5, ANK2, GATA3, GAB1, TGFBI, SERPINE1, RRAS, RARB, RECK, DAB2IP, PTPRM, ROCK1, DLL1, MYH9, GRHL2, SERPINF2, VEGFA, PDGFRA, PDGFRB, AKAP6, ASB4, CAV3, HOXA13, SOX4, EPHB2, ACE, PPP1R16B, ECE1, OVOL2, TFDP2, AXIN2, ASXL2, MAP2K1, MET, SMAD4, PTPN14, WHSC1, HGF, UBP1, FOXP1, NOTCH3, NOTCH2, NOTCH1, PLCG1, KCNJ8, NEDD4, TRPS1, MARCKS, NCOR2, KLF4, NRP2, RHOJ, NDST1, AP1B1, PPARG, HPSE, SEMA3E, TGFA, FOSL1, SH3PXD2B, ANP32B, ADIPOR2, TP53, LEF1, PRKCB, PRKD1, ACVR2B, XIRP1, MDM4, PARVA, TH, PPP3R1, FKBP1A, BORCS8, CALCA, APLNR, XBP1, SPEG, FAT4, CHM, SLC4A7, NFATC4, PTPRB, CREB1, LMNA, SIRT6, SIRT1, SNAI1, TP73, CDH13, DKK1, PKNOX1, SP2, PLN, HEYL, POFUT1 | 1.491146268 | 0.234575732 | 0.001484037 | 0.06960898 |
| GO:0072358~cardiovascular system development | 104 | 7.624633431 | 3.50E-05 | PGF, FGFRL1, RORA, JAG1, CXCL10, DDX17, PTGIS, MYOCD, ATG5, ANK2, GATA3, GAB1, TGFBI, SERPINE1, RRAS, RARB, RECK, DAB2IP, PTPRM, ROCK1, DLL1, MYH9, GRHL2, SERPINF2, VEGFA, PDGFRA, PDGFRB, AKAP6, ASB4, CAV3, HOXA13, SOX4, EPHB2, ACE, PPP1R16B, ECE1, OVOL2, TFDP2, AXIN2, ASXL2, MAP2K1, MET, SMAD4, PTPN14, WHSC1, HGF, UBP1, FOXP1, NOTCH3, NOTCH2, NOTCH1, PLCG1, KCNJ8, NEDD4, TRPS1, MARCKS, NCOR2, KLF4, NRP2, RHOJ, NDST1, AP1B1, PPARG, HPSE, SEMA3E, TGFA, FOSL1, SH3PXD2B, ANP32B, ADIPOR2, TP53, LEF1, PRKCB, PRKD1, ACVR2B, XIRP1, MDM4, PARVA, TH, PPP3R1, FKBP1A, BORCS8, CALCA, APLNR, XBP1, SPEG, FAT4, CHM, SLC4A7, NFATC4, PTPRB, CREB1, LMNA, SIRT6, SIRT1, SNAI1, TP73, CDH13, DKK1, PKNOX1, SP2, PLN, HEYL, POFUT1 | 1.491146268 | 0.234575732 | 0.001484037 | 0.06960898 |
| GO:0044802~single-organism membrane organization | 77 | 5.64516129 | 3.54E-05 | SYT1, PPP2R5A, GLRA3, SNCA, RHOQ, PIP5K1A, PEX3, KIF13A, ANK2, ANK3, TRIM9, RAB29, FAM73B, VPS4A, DLG3, CNTNAP2, SAR1A, MYC, SH3PXD2B, PID1, STX1A, ATG9A, ROCK1, NRXN2, FLOT2, TP53, SRPRA, ARHGEF9, MYADM, VTI1A, VAT1, BBS1, MFN2, LETM1, ATG4B, CNTN2, MYRF, UBL4A, RELN, ATPIF1, CLIP3, VAMP2, PACS1, CALCR, CAV3, SNX9, PPIL2, PPFIA1, BET1, CACNB1, CALCOCO2, EEA1, ZDHHC23, MOAP1, TSPAN33, FAT4, BCL2, STX17, TOR1A, SEC16A, CHM, LYPLAL1, FCHO2, SEC61A1, PIK3R2, GPR158, PTPRD, DNM1L, CNST, MYO1C, LMNA, TSPAN14, CRB3, TMBIM1, WIPI2, PPP1R9B, LRP4 | 1.61148997 | 0.236529522 | 0.001489943 | 0.070274258 |
| GO:2001257~regulation of cation channel activity | 19 | 1.392961877 | 4.03E-05 | JPH3, NOS1AP, DRD2, CACNB1, ASIC2, CACNB3, FKBP1A, FKBP1B, CRHR1, KCNS2, PLCG1, ANK2, ANK3, PLN, PDE4B, AKAP6, PLA2G6, NEDD4L, VAMP2 | 3.032554124 | 0.264645586 | 0.0016876 | 0.080040668 |
| GO:1903506~regulation of nucleic acid-templated transcription | 254 | 18.62170088 | 4.05E-05 | RORA, DDX17, CRY2, WDR77, MED27, CREB3L1, RARB, TWIST2, DAB2IP, STRN3, TAF4B, YY1, ZHX2, HNF4G, PPARGC1B, MED19, UHRF2, HNF4A, MTF1, SERPINF2, MCIDAS, VEGFA, FOXG1, MNT, TGIF2, ATXN1L, SUPT6H, TNRC18, HOXA13, CRTC1, TFCP2L1, NFKBIA, MYT1, TAL2, PLAGL1, TNKS, PLAG1, ASXL2, KLF6, BMP3, ESRRA, MAP2K1, SMAD4, PTPN14, KLF17, MSTN, SKI, UBP1, USF1, SFMBT2, ZBTB42, NOTCH3, NOTCH2, DLX1, NOTCH1, ZFHX4, NR1I2, CSRNP3, TRPS1, JAZF1, RBMXL1, TCF12, FOXI1, KLF4, NCOR2, BMP8B, CREBRF, HNF1A, CPEB3, PPARG, MITF, ZEB1, ANP32A, MKX, MYC, AGAP2, CSDC2, TP53, ERLIN1, SPEN, NFAM1, IL6R, FOSB, MYCN, FOXR2, MXD4, CCND1, NSD1, CLOCK, ZMYND11, SCML2, KIT, XBP1, BCL11B, PER2, GATAD2B, MLLT1, BCL6, BCL9L, ZSCAN29, BAZ2A, TLX2, MLLT3, NOS1, LMNA, TNP1, ZFP2, ATMIN, TMPRSS6, SNAI1, TRIM21, TP73, PREB, CDH13, RPS6KA4, PHF19, PKNOX1, DKK1, MAPK13, KDM8, ZBTB4, NEUROD2, TP53INP2, LRP4, TP53INP1, RNASEL, CBX3, JAG1, MAF1, NLRC5, WNT1, PTGIS, OLIG3, MYOCD, MDFIC, GATA3, TGS1, SAMD4A, PID1, SATB1, RBFOX2, RET, SATB2, MTA2, SIX3, FOXN2, FGF23, DLL1, GRHL2, PKIA, FOXN3, IGSF1, TRIM32, NFE2L1, RELN, GLP1R, CAMTA1, DRD2, STRAP, ONECUT2, SOX4, LIN28A, GREM2, SRC, NR2C2, FOXQ1, OVOL2, RNF165, OVOL1, TFDP2, AXIN2, TAF5, MET, NR4A2, HACE1, WHSC1, FOXP3, TRIM62, FOXP1, FOXP2, CDKN1C, PPIE, HDAC1, NEDD4, GTF2F1, ZIC5, ATF7, POU6F1, E2F3, ARID4A, E2F5, ARID4B, RHOQ, CBFA2T3, PDCD4, LGR4, TMEM173, MAZ, PRMT2, PEX2, PAX7, RTF1, WWC3, LTF, LRRFIP1, PLCB1, FOSL1, KMT5B, IRAK2, TBL1XR1, KDM7A, FOXJ2, PADI2, PRKCH, LEF1, DDN, PURB, NRIP2, PRKCB, PRKD1, PRKCQ, ACVR2B, NCOA1, MYRF, MDM4, JMJD1C, CUX1, ABLIM1, FRK, KMT2D, ING3, ABLIM3, CTNND2, PPP3R1, PPM1A, CTCFL, CALCA, RGMB, POU2F1, NFATC4, PIK3R2, CHD3, CREB1, CREB5, SIRT6, SIRT1, ATXN7L3, SP2, ATXN7, HEYL, IRF1, KDM4C, IRF4, RNF41 | 1.255677977 | 0.265662291 | 0.001685934 | 0.080400771 |
| GO:0043543~protein acylation | 33 | 2.419354839 | 4.21E-05 | ING3, HNF1A, SNCA, PPM1A, SOX4, ZDHHC23, JADE2, NAA50, MYOCD, ATG5, BRPF3, XBP1, GATA3, ANP32A, NOS1, TAF5, ZDHHC8, SMAD4, LEF1, FOXP3, SIRT1, NMT2, ZDHHC15, MSL2, NCOA1, ZDHHC17, ZDHHC16, RPS6KA4, HDAC1, CLIP3, IRF4, CLOCK, MAP6D1 | 2.188598903 | 0.274567333 | 0.001742974 | 0.083576237 |
| GO:2000113~negative regulation of cellular macromolecule biosynthetic process | 127 | 9.31085044 | 4.32E-05 | CBX3, MAF1, NLRC5, PTGIS, CRY2, MYOCD, OLIG3, MDFIC, GATA3, RARB, SAMD4A, TWIST2, PID1, SATB1, RBFOX2, SATB2, DAB2IP, MTA2, YY1, STRN3, ZHX2, SIX3, PKIA, PPARGC1B, FOXN3, UHRF2, HNF4A, FOXG1, VEGFA, MNT, TGIF2, ATXN1L, TNRC18, STRAP, TFCP2L1, NFKBIA, SRC, OVOL2, CNOT6L, OVOL1, TFDP2, NAT10, ASXL2, ESRRA, MET, NR4A2, SMAD4, KLF17, SKI, WHSC1, FOXP3, FOXP1, ZBTB42, FOXP2, CDKN1C, NOTCH3, NOTCH2, NOTCH1, DLX1, NR1I2, HDAC1, NEDD4, TRPS1, ATF7, JAZF1, KLF4, NCOR2, CREBRF, HNF1A, ARID4A, CPEB2, CPEB3, MITF, PPARG, ZEB1, CBFA2T3, PDCD4, LGR4, PRMT2, PEX2, WWC3, RTF1, MKX, PLCB1, MYC, IRAK2, TBL1XR1, TP53, LEF1, SPEN, PURB, NRIP2, MXD4, ACVR2B, CCND1, MDM4, CUX1, NSD1, CLOCK, KMT2D, FRK, ZMYND11, CALCA, XBP1, POU2F1, PER2, GATAD2B, BCL6, NFATC4, AGO4, BAZ2A, CREB1, TNP1, SIRT6, SNAI1, SIRT1, TMPRSS6, TRIM21, TP73, PHF19, DKK1, RPL13A, CDAN1, OBFC1, HEYL, IRF1, ZBTB4 | 1.418916114 | 0.28058174 | 0.001778483 | 0.085743 |
| GO:0001504~neurotransmitter uptake | 9 | 0.659824047 | 4.44E-05 | RAB3B, NOS1, DRD2, SLC6A3, TOR1A, SNCA, PER2, ATP1A2, NAT8L | 6.22471636 | 0.287143941 | 0.001818107 | 0.088127819 |
| GO:0051252~regulation of RNA metabolic process | 263 | 19.28152493 | 4.74E-05 | RORA, WTAP, DDX17, CRY2, MED27, WDR77, CREB3L1, RARB, TWIST2, DAB2IP, STRN3, TAF4B, YY1, ZHX2, HNF4G, PPARGC1B, MED19, UHRF2, MTF1, HNF4A, SERPINF2, MCIDAS, VEGFA, FOXG1, MNT, TGIF2, ATXN1L, SUPT6H, TNRC18, HOXA13, CRTC1, TFCP2L1, NFKBIA, MYT1, TAL2, PLAGL1, TNKS, PLAG1, ASXL2, KLF6, BMP3, ESRRA, MAP2K1, SMAD4, PTPN14, KLF17, MSTN, SKI, UBP1, USF1, SFMBT2, ZBTB42, NOTCH3, NOTCH2, DLX1, NOTCH1, ZFHX4, NR1I2, CSRNP3, TRPS1, JAZF1, RBMXL1, TCF12, FOXI1, KLF4, NCOR2, BMP8B, CREBRF, HNF1A, CPEB3, PPARG, MITF, ZEB1, ANP32A, MKX, MYC, AGAP2, CSDC2, TP53, ERLIN1, SPEN, NFAM1, IL6R, FOSB, MYCN, FOXR2, MXD4, CCND1, NSD1, CLOCK, ZMYND11, SCML2, KIT, XBP1, BCL11B, PER2, GATAD2B, MLLT1, BCL6, BCL9L, ZSCAN29, BAZ2A, TLX2, MLLT3, NOS1, LMNA, TNP1, AFF2, ZFP2, ATMIN, TMPRSS6, SNAI1, TRIM21, TP73, PREB, CDH13, RPS6KA4, PHF19, PKNOX1, DKK1, MAPK13, KDM8, ZBTB4, NEUROD2, TP53INP2, LRP4, TP53INP1, NCBP2, RNASEL, CBX3, JAG1, MAF1, NLRC5, WNT1, PTGIS, OLIG3, MYOCD, MDFIC, GATA3, TGS1, SAMD4A, PID1, SATB1, RBFOX2, RET, SATB2, MTA2, SIX3, FOXN2, FGF23, DLL1, GRHL2, PKIA, FOXN3, IGSF1, CELF6, TRIM32, CELF3, NFE2L1, RELN, GLP1R, CAMTA1, DRD2, STRAP, ONECUT2, SOX4, LIN28A, GREM2, SRC, NR2C2, FOXQ1, OVOL2, RNF165, OVOL1, TFDP2, AXIN2, TAF5, MET, NR4A2, HACE1, WHSC1, RNPS1, FOXP3, TRIM62, FOXP1, FOXP2, CDKN1C, PPIE, HDAC1, NEDD4, GTF2F1, ZIC5, ATF7, POU6F1, E2F3, ARID4A, E2F5, ARID4B, RHOQ, CBFA2T3, PDCD4, LGR4, TMEM173, MAZ, PRMT2, PCBP4, PEX2, PAX7, RTF1, WWC3, LTF, LRRFIP1, PLCB1, FOSL1, KMT5B, IRAK2, TBL1XR1, KDM7A, FOXJ2, PADI2, PRKCH, LEF1, DDN, PURB, NRIP2, PRKCB, PRKD1, PRKCQ, ACVR2B, NCOA1, MYRF, MDM4, JMJD1C, CUX1, ABLIM1, FRK, KMT2D, ING3, ABLIM3, CTNND2, PPP3R1, PPM1A, CTCFL, CALCA, RGMB, POU2F1, CHD1, NFATC4, TNRC6B, PIK3R2, CHD3, CREB1, CREB5, SIRT6, SIRT1, ATXN7L3, SP2, ATXN7, HEYL, IRF1, KDM4C, IRF4, RNF41 | 1.247110982 | 0.303443429 | 0.001931855 | 0.094147398 |
| GO:0009887~organ morphogenesis | 102 | 7.478005865 | 4.78E-05 | PGF, FGFRL1, ENAM, JAG1, CALB1, HS2ST1, WNT1, GATA3, RARB, TWIST2, SATB2, PTPRM, YY1, SIX3, DLL1, GRHL2, PPARGC1B, FOXN3, FOXG1, VEGFA, PDGFRA, PDGFRB, STC1, CAV3, PPP2R3A, HOXA13, DAG1, LRIG1, SOX4, SRC, EPHB2, OVOL2, PVRL1, MYO15A, AXIN2, ASXL2, PLAG1, ABR, MAP2K1, MET, NTN4, SMAD4, ROGDI, WHSC1, SKI, GAS1, HGF, FOXP2, NOTCH2, NOTCH1, DLX1, HDAC1, NEDD4, TRPS1, AREG, FOXI1, NCOR2, NRP2, HNF1A, E2F5, NDST1, ZEB1, LGR4, PAX7, LTF, TGFA, MYC, SDK2, TP53, LEF1, EDAR, PRKCB, MYCN, BBS1, MFN2, ACVR2B, CLIC5, WNT9B, MDM4, PARVA, TH, CTNND1, FKBP1A, FAT3, FAT4, XBP1, BCL11B, BCL2, POU2F1, SLC4A7, MLLT3, SLC12A2, SIRT6, COL5A2, SNAI1, WNT2B, DKK1, RPL13A, BNC2, HEYL, TMTC3, LRP4 | 1.485940315 | 0.305667499 | 0.001938567 | 0.094979645 |
| GO:0042592~homeostatic process | 150 | 10.99706745 | 5.09E-05 | TUSC3, ADCY5, SNCA, LHCGR, ILDR2, RORA, CXCL11, SIDT2, CALB1, CXCL10, SLC16A1, CRY2, ANK2, ATG5, ANK3, GATA3, PDE4B, SERPINE1, MS4A2, SLC25A27, DISC1, RBFOX2, SCN2B, RAP1GDS1, FGF23, DLL1, PPARGC1B, CRHR1, MAP4K4, TNS2, VSIG1, GPR55, HNF4A, LYST, TRIM32, PGM1, VEGFA, PDGFRA, PDGFRB, AKAP6, NFE2L1, ATPIF1, STC1, GLP1R, ALDOA, CAV3, GCNT2, HOXA13, STRAP, DRD2, SOX4, BDKRB2, MYT1, SRC, CSMD1, SLX4, ACE, LPCAT1, NAT10, TNKS, SLC30A3, SLC31A2, TMEM79, SLC30A6, RPS24, CSF1R, TMEM97, MUC2, MET, IL1RN, SMAD4, MSTN, FOXP3, USF1, KCNK3, PLCG1, CACNA1E, ADRA1D, NCOR2, BMP8B, OPRM1, JPH3, HNF1A, ARID4A, PPARG, LGR4, DNAJC16, PEX2, LTF, MYC, GPIHBP1, TP53, ADIPOR2, CHST3, CFTR, CDK6, PFKM, PRKCE, PRKCB, BBS1, PRKD1, PRKCQ, MAGT1, DGAT1, UBE2K, SLC26A9, PLA2G6, ORMDL3, GPR12, CALCR, CLDN18, GLRX5, CCR1, TH, HK1, KITLG, FKBP1A, KIT, TRIM10, FKBP1B, TPCN2, CALCA, NPTX1, XBP1, BCL2, BCL6, SCNN1G, INPP5D, NEDD4L, PIK3R2, NOS1, NCDN, SLC12A2, SWAP70, MAP1A, PDK3, AXL, SIRT6, ATP1A2, AMPD2, TMPRSS6, SIRT1, ERP44, PKNOX1, OBFC1, PLN, MEX3C, HTR2C, TP53INP2, HTR2A | 1.367640129 | 0.322042523 | 0.002054346 | 0.101190299 |
| GO:0051336~regulation of hydrolase activity | 115 | 8.431085044 | 5.39E-05 | PPP2R5A, SNCA, TNFSF14, RASGEF1C, ITSN1, CRY2, SERPINE1, MS4A2, SPRED1, RNF34, RECK, DAB2IP, RET, STMN3, ROCK1, PSD3, RAP1GDS1, RIC8B, PPARGC1B, FARP2, SGSM2, GPR55, SERPINF2, VEGFA, PDGFRA, PDGFRB, AKAP6, ATPIF1, NEK5, WFDC5, SNX9, RALGPS2, RALGPS1, DRD2, SIPA1, SRC, GMIP, LMTK3, TBC1D30, AXIN2, FGD6, FGD3, HIP1, ARHGEF33, ABR, MAP2K1, DOCK9, ARPP19, DOCK8, HGF, DOCK3, CORO1C, TBC1D25, EPHA4, UACA, CSRNP3, PKP4, RGS6, MARCKS, DNAJB1, KLF4, CPEB2, PPARG, PIP5K1A, NMUR1, ARHGAP1, LTF, DLG3, PLCB1, GPIHBP1, MYC, AGAP2, ALS2CL, ARHGEF3, ANP32B, RAB4A, RIMBP2, CYCS, LEF1, ARHGEF9, ARHGAP26, PRKD1, PSME1, DNAJC24, SERPINB8, SERPINB2, PSME3, SRGAP2, CALCR, RAP1GAP, CYTH4, FKBP1A, FKBP1B, PLEKHG2, CCL22, RASGRP4, ARHGAP44, CHM, BCL6, RASA4, ELMOD1, TBC1D2, GNAO1, CNST, NOS1, FETUB, PPP1R11, RCAN1, DGKI, SIRT1, RALGDS, PPP1R9B, PLN, EVI5L, HTR2A | 1.442399732 | 0.337330102 | 0.002163332 | 0.107125067 |
| GO:0051345~positive regulation of hydrolase activity | 77 | 5.64516129 | 5.45E-05 | SNCA, PPARG, RASGEF1C, PIP5K1A, ITSN1, NMUR1, ARHGAP1, MS4A2, PLCB1, AGAP2, GPIHBP1, MYC, ALS2CL, ARHGEF3, DAB2IP, RET, ANP32B, RAB4A, CYCS, RAP1GDS1, PSD3, ARHGEF9, RIC8B, PPARGC1B, ARHGAP26, FARP2, PRKD1, PSME1, GPR55, SGSM2, DNAJC24, VEGFA, PDGFRA, PDGFRB, AKAP6, PSME3, NEK5, SRGAP2, CALCR, SNX9, RALGPS2, RALGPS1, RAP1GAP, SIPA1, CYTH4, PLEKHG2, CCL22, GMIP, ARHGAP44, RASGRP4, CHM, TBC1D30, AXIN2, FGD6, RASA4, FGD3, ELMOD1, TBC1D2, HIP1, ARHGEF33, ABR, GNAO1, MAP2K1, DOCK9, DOCK8, SIRT1, RALGDS, DOCK3, CORO1C, TBC1D25, UACA, PKP4, RGS6, MARCKS, EVI5L, DNAJB1, HTR2A | 1.590859231 | 0.34001184 | 0.002173203 | 0.108180203 |
| GO:0051926~negative regulation of calcium ion transport | 14 | 1.026392962 | 5.63E-05 | CAV3, NOS1, GNAO1, DRD2, FKBP1A, ATP1A2, PRKCE, FKBP1B, CALCA, CRHR1, ACE, PLN, BCL2, STC1 | 3.765569156 | 0.349343067 | 0.002235896 | 0.111885217 |
| GO:0010605~negative regulation of macromolecule metabolic process | 207 | 15.17595308 | 5.67E-05 | DYNC1LI1, SNCA, CRY2, SERPINE1, RARB, TWIST2, RECK, DAB2IP, ROCK1, YY1, STRN3, ZHX2, PTPRR, PPARGC1B, UHRF2, HNF4A, SERPINF2, FOXG1, VEGFA, MNT, TGIF2, ATXN1L, WFDC5, SUPT6H, TNRC18, TFCP2L1, DAG1, NFKBIA, BDKRB2, MYT1, ACE, NAT10, TNKS, PLAG1, ASXL2, ESRRA, MAP2K1, SMAD4, KLF17, SKI, HGF, GAS1, SFMBT2, ZBTB42, NOTCH3, NOTCH2, DLX1, NOTCH1, NR1I2, CSRNP3, TRPS1, JAZF1, NCOR2, KLF4, CREBRF, HNF1A, CPEB2, CPEB3, MITF, PPARG, ZEB1, ANP32A, MKX, AGAP2, MYC, FLOT2, TP53, IL6R, SPEN, MYADM, MXD4, INHBB, CCND1, SERPINB8, SERPINB2, NSD1, CLOCK, ZMYND11, CCR1, FKBP1A, FKBP1B, SF3B3, XBP1, LRRTM2, PER2, MLLT1, GATAD2B, BCL6, AGO4, BAZ2A, NOS1, CNST, SWAP70, PPP1R11, TNP1, MARCH8, SNAI1, TMPRSS6, TRIM21, TP73, DUSP4, DKK1, PHF19, OBFC1, NEUROD2, ZBTB4, SVIP, NCBP2, SRCIN1, CTDSPL, XPO5, TNFSF14, CBX3, MAF1, NLRC5, WNT1, PTGIS, ATG5, MYOCD, OLIG3, MDFIC, GATA3, CTDSP2, SPRED1, RNF34, SAMD4A, PID1, RBFOX2, SATB1, SATB2, MTA2, SIX3, SOCS4, PKIA, FOXN3, UCN2, TRIM32, CAV3, STRAP, SOX4, LIN28A, SRC, PPP1R16B, OVOL2, ITGB8, CNOT6L, OVOL1, TFDP2, RNF169, LMTK3, MET, NR4A2, WHSC1, RNPS1, FOXP3, TRIM62, FOXP1, FOXP2, CORO1C, CDKN1C, HDAC1, NEDD4, ATF7, DNAJB2, ARID4A, CBFA2T3, PDCD4, LGR4, PRMT2, PEX2, PCBP4, RTF1, WWC3, DLG3, PLCB1, IRAK2, TBL1XR1, ANKS1A, RIMBP2, LEF1, PRKCE, NRIP2, PURB, ACVR2B, MDM4, CUX1, FRK, KMT2D, RTN4RL1, CALCA, MARVELD3, POU2F1, NFATC4, INPP5D, TNRC6B, FETUB, CREB1, SIRT6, SIRT1, PPP1R9B, RPL13A, CDAN1, HEYL, IRF1, SMPD1, KDM4C, IGFBP3 | 1.290359657 | 0.351213504 | 0.002239207 | 0.112634257 |
| GO:0031056~regulation of histone modification | 24 | 1.759530792 | 5.80E-05 | ASXL2, NOS1, SNCA, TP53, SMAD4, CTCFL, FOXP3, SIRT1, PRKD1, PHF19, RPS6KA4, ATG5, MYOCD, XBP1, TRPS1, GATA3, PAX7, VEGFA, RTF1, ANP32A, KDM4C, BCL6, NSD1, SUPT6H | 2.544409607 | 0.35740821 | 0.00227702 | 0.115130475 |
[truncated: 990,010 more chars]
