# Supplementary material for: MicroRNA mediated regulation in early-onset cardiac hypertrophy: Insights from the hypertrophic heart rat model
Source: PLoS One. 2025 Dec 30;20(12):e0338909. doi: 10.1371/journal.pone.0338909 (PMC12752997; doi:10.1371/journal.pone.0338909)
Supplement: S1 File — S1 Tables. MicroRNA and Genes Microarray Results (HHR/NHR, 2-days). S2 Tables. Target genes and pathway enrichment analysis. S1 Fig. Number of KEGG Pathways potentially regulated by miRNAs under investigation either individually or communally. S2 Fig. Number of GO:BP potentially regulated by microRNAs under investigation either individually or communally. S3 Fig. Comparative analysis of predicted target genes regulated by miR-34a, miR-351, and miR-490*. S4 Fig. Raw Ct values from RT-PCR of h9C2-1 transfected with mimics and inhibitors. (ZIP) [file pone.0338909.s001.zip › Supplementary Data_Major Revision/S1_Table.docx]

## Table S1A: MicroRNA Microarray Results (HHR/NHR, 2-days)

**S1A Data — miRNA differential expression (HHR vs NHR).** Platform: [array/platform]; normalization: [method]. Statistics: two-tailed test as described in Methods; multiple testing: Benjamini–Hochberg FDR. Columns include raw *p*-values and adjusted FDR values. Fold change is HHR/NHR (>1 = up in HHR). **Nomenclature:** legacy “miR-490*” corresponds to **miR-490-3p**.

| Column | Probeset ID | p-value | bonferroni(p-value) | stepup(p-value) | stepdown(p-value) | qvalue(p-value) | t | Mean(HHR) | Mean(NHR) | MeanRatio(HHR/NHR) | MeanDiff(HHR-NHR) | FoldChange(HHR/NHR) |
| --- | --- | --- | --- | --- | --- | --- | --- | --- | --- | --- | --- | --- |
| 360 | rno-miR-34a | 3.07E-07 | 0.000207556 | 0.000207556 | 0.000207556 | 0.000181905 | 9.07486 | 7.36547 | 6.07453 | 2.44688 | 1.29094 | 2.44688 |
| 473 | rno-miR-378* | 8.87E-06 | 0.00600292 | 0.00214725 | 0.0059852 | 0.00188189 | 6.78082 | 6.92694 | 6.21944 | 1.63296 | 0.707492 | 1.63296 |
| 472 | rno-miR-378 | 9.52E-06 | 0.00644176 | 0.00214725 | 0.00640375 | 0.00188189 | 6.73713 | 9.8838 | 9.13906 | 1.67567 | 0.744742 | 1.67567 |
| 223 | rno-miR-218 | 7.17E-05 | 0.0485395 | 0.0121349 | 0.0481102 | 0.0106352 | 5.54885 | 5.962 | 5.24904 | 1.63916 | 0.712958 | 1.63916 |
| 522 | rno-miR-466b | 0.000118969 | 0.0805422 | 0.0161084 | 0.0795933 | 0.0141177 | -5.26762 | -1.72413 | 4.04793 | 0.0182994 | -5.77206 | -54.6465 |
| 542 | rno-miR-490* | 0.000176118 | 0.119232 | 0.0170331 | 0.117128 | 0.0149281 | 5.05368 | 7.11909 | 6.56713 | 1.46607 | 0.551958 | 1.46607 |
| 613 | rno-miR-675* | 0.000165838 | 0.112273 | 0.0170331 | 0.11062 | 0.0149281 | 5.08626 | 5.44388 | 4.88357 | 1.47458 | 0.560304 | 1.47458 |
| 367 | rno-miR-351 | 0.000207967 | 0.140794 | 0.0175992 | 0.137897 | 0.0154242 | 4.96399 | 3.95167 | 3.39052 | 1.47545 | 0.561154 | 1.47545 |
| 610 | rno-miR-674-3p | 0.000240099 | 0.162547 | 0.0180608 | 0.158728 | 0.0158288 | 4.8869 | 3.16013 | 2.59726 | 1.47721 | 0.562874 | 1.47721 |
| 294 | rno-miR-3085 | 0.000300063 | 0.203143 | 0.0203143 | 0.197778 | 0.0178038 | -4.76806 | 5.07373 | 5.25318 | 0.883036 | -0.179456 | -1.13246 |
| 214 | rno-miR-214 | 0.000344957 | 0.233536 | 0.0212305 | 0.226687 | 0.0186068 | 4.6942 | 9.24119 | 8.7509 | 1.40473 | 0.490289 | 1.40473 |
| 488 | rno-miR-410 | 0.000521029 | 0.352737 | 0.0293947 | 0.341367 | 0.025762 | 4.47764 | 5.14389 | 4.41239 | 1.66036 | 0.7315 | 1.66036 |
| 558 | rno-miR-503 | 0.000624319 | 0.422664 | 0.0325126 | 0.407813 | 0.0284945 | 4.38352 | 7.39063 | 6.85789 | 1.44668 | 0.532741 | 1.44668 |
| 174 | rno-miR-199a-5p | 0.000937362 | 0.634594 | 0.0453282 | 0.610457 | 0.0397263 | 4.17366 | 8.48297 | 7.9501 | 1.44681 | 0.532875 | 1.44681 |
| 541 | rno-miR-490 | 0.00109041 | 0.73821 | 0.0461381 | 0.70586 | 0.0404362 | 4.0961 | 6.97871 | 6.50572 | 1.38798 | 0.472991 | 1.38798 |
| 501 | rno-miR-431 | 0.00102384 | 0.693137 | 0.0461381 | 0.664766 | 0.0404362 | 4.12837 | 3.83364 | 3.18395 | 1.56883 | 0.649691 | 1.56883 |
| 173 | rno-miR-199a-3p | 0.00218311 | 1 | 0.0869393 | 1 | 0.0761949 | 3.74307 | 10.197 | 9.74309 | 1.3697 | 0.453864 | 1.3697 |
| 574 | rno-miR-542-5p | 0.00265234 | 1 | 0.0997573 | 1 | 0.0874289 | 3.64475 | 7.40791 | 6.96427 | 1.36004 | 0.443645 | 1.36004 |
| 327 | rno-miR-329 | 0.00370529 | 1 | 0.125424 | 1 | 0.109924 | 3.47636 | 4.10398 | 3.50679 | 1.51277 | 0.597193 | 1.51277 |
| 285 | rno-miR-300-3p | 0.00354359 | 1 | 0.125424 | 1 | 0.109924 | 3.49881 | 4.65917 | 4.05541 | 1.51968 | 0.603763 | 1.51968 |
| 497 | rno-miR-423* | 0.00397429 | 1 | 0.128123 | 1 | 0.112289 | 3.44111 | 2.80217 | -1.03812 | 14.3232 | 3.84028 | 14.3232 |
| 530 | rno-miR-483 | 0.00416558 | 1 | 0.128176 | 1 | 0.112335 | 3.41747 | 6.10525 | 5.8877 | 1.16276 | 0.217549 | 1.16276 |
| 573 | rno-miR-542-3p | 0.00435457 | 1 | 0.128176 | 1 | 0.112335 | 3.39516 | 7.06861 | 6.66463 | 1.32315 | 0.403977 | 1.32315 |
| 314 | rno-miR-322* | 0.00459227 | 1 | 0.12954 | 1 | 0.113531 | 3.36845 | 6.79161 | 6.3518 | 1.35643 | 0.439811 | 1.35643 |
| 554 | rno-miR-500 | 0.00555766 | 1 | 0.144713 | 1 | 0.126829 | -3.27255 | 7.15737 | 7.5349 | 0.769755 | -0.377529 | -1.29911 |
| 565 | rno-miR-532-3p | 0.00543585 | 1 | 0.144713 | 1 | 0.126829 | -3.28369 | 4.13872 | 4.5033 | 0.776696 | -0.364578 | -1.28751 |
| 235 | rno-miR-223 | 0.00710212 | 1 | 0.160271 | 1 | 0.140464 | -3.14926 | 5.66897 | 5.8028 | 0.911405 | -0.133836 | -1.09721 |
| 532 | rno-miR-484 | 0.00698404 | 1 | 0.160271 | 1 | 0.140464 | 3.15769 | 5.08696 | 4.77567 | 1.24082 | 0.311292 | 1.24082 |
| 164 | rno-miR-1949 | 0.0069427 | 1 | 0.160271 | 1 | 0.140464 | 3.16068 | 8.0301 | 7.67666 | 1.2776 | 0.353437 | 1.2776 |
| 148 | rno-miR-18a | 0.0070252 | 1 | 0.160271 | 1 | 0.140464 | 3.15474 | 7.29576 | 6.88714 | 1.32742 | 0.408625 | 1.32742 |
| 33 | rno-miR-106b* | 0.00754154 | 1 | 0.161686 | 1 | 0.141705 | 3.11905 | 2.42413 | 2.05587 | 1.29079 | 0.368253 | 1.29079 |
| 512 | rno-miR-450a | 0.00764249 | 1 | 0.161686 | 1 | 0.141705 | 3.11236 | 6.64388 | 6.23294 | 1.32956 | 0.410948 | 1.32956 |
| 7 | rno-let-7b | 0.00930603 | 1 | 0.170275 | 1 | 0.149232 | 3.01313 | 10.5739 | 10.2752 | 1.2301 | 0.298777 | 1.2301 |
| 9 | rno-let-7c | 0.00904918 | 1 | 0.170275 | 1 | 0.149232 | 3.02725 | 11.0688 | 10.7695 | 1.23051 | 0.299251 | 1.23051 |
| 335 | rno-miR-335 | 0.0087278 | 1 | 0.170275 | 1 | 0.149232 | 3.04548 | 8.71438 | 8.31274 | 1.32101 | 0.401643 | 1.32101 |
| 535 | rno-miR-487b | 0.00856299 | 1 | 0.170275 | 1 | 0.149232 | 3.05508 | 5.55733 | 4.98982 | 1.48196 | 0.567505 | 1.48196 |
| 346 | rno-miR-341 | 0.00914246 | 1 | 0.170275 | 1 | 0.149232 | 3.02208 | 3.39799 | -0.195726 | 12.073 | 3.59371 | 12.073 |
| 425 | rno-miR-3584-5p | 0.0136194 | 1 | 0.175292 | 1 | 0.153628 | -2.8205 | -1.68459 | 3.95311 | 0.0200855 | -5.6377 | -49.7872 |
| 450 | rno-miR-362 | 0.0132011 | 1 | 0.175292 | 1 | 0.153628 | -2.83633 | 3.23075 | 3.67229 | 0.736347 | -0.441543 | -1.35806 |
| 666 | rno-miR-92a | 0.0121854 | 1 | 0.175292 | 1 | 0.153628 | 2.87691 | 9.3567 | 9.08005 | 1.21138 | 0.276647 | 1.21138 |
| 680 | rno-miR-99b | 0.0106078 | 1 | 0.175292 | 1 | 0.153628 | 2.94704 | 7.74751 | 7.45976 | 1.22073 | 0.287747 | 1.22073 |
| 13 | rno-let-7e | 0.0130209 | 1 | 0.175292 | 1 | 0.153628 | 2.8433 | 10.0232 | 9.73112 | 1.22438 | 0.292054 | 1.22438 |
| 59 | rno-miR-128 | 0.0114158 | 1 | 0.175292 | 1 | 0.153628 | 2.90993 | 6.39198 | 6.06596 | 1.25354 | 0.326012 | 1.25354 |
| 678 | rno-miR-99a | 0.0126344 | 1 | 0.175292 | 1 | 0.153628 | 2.85857 | 9.42523 | 9.08622 | 1.26489 | 0.339011 | 1.26489 |
| 202 | rno-miR-20a | 0.013265 | 1 | 0.175292 | 1 | 0.153628 | 2.83388 | 9.95872 | 9.5986 | 1.28353 | 0.36012 | 1.28353 |
| 177 | rno-miR-19b | 0.0100302 | 1 | 0.175292 | 1 | 0.153628 | 2.97532 | 10.1316 | 9.76467 | 1.28959 | 0.366908 | 1.28959 |
| 175 | rno-miR-19a | 0.0107777 | 1 | 0.175292 | 1 | 0.153628 | 2.93901 | 7.31459 | 6.94485 | 1.29211 | 0.369733 | 1.29211 |
| 504 | rno-miR-434 | 0.013168 | 1 | 0.175292 | 1 | 0.153628 | 2.8376 | 5.95532 | 5.56324 | 1.31229 | 0.392081 | 1.31229 |
| 57 | rno-miR-127 | 0.013019 | 1 | 0.175292 | 1 | 0.153628 | 2.84337 | 5.85852 | 5.45938 | 1.31872 | 0.399137 | 1.31872 |
| 26 | rno-miR-101b | 0.0139819 | 1 | 0.175292 | 1 | 0.153628 | 2.80716 | 6.06284 | 5.66202 | 1.32025 | 0.400817 | 1.32025 |
| 474 | rno-miR-379 | 0.0109361 | 1 | 0.175292 | 1 | 0.153628 | 2.93164 | 4.80124 | 4.33245 | 1.38394 | 0.468781 | 1.38394 |
| 81 | rno-miR-136 | 0.0130329 | 1 | 0.175292 | 1 | 0.153628 | 2.84283 | 4.70865 | 4.04244 | 1.5869 | 0.666211 | 1.5869 |
| 306 | rno-miR-31 | 0.0138803 | 1 | 0.175292 | 1 | 0.153628 | 2.81086 | 3.78673 | 0.523779 | 9.59941 | 3.26295 | 9.59941 |
| 587 | rno-miR-598-3p | 0.0103507 | 1 | 0.175292 | 1 | 0.153628 | 2.95944 | 3.36322 | -0.169899 | 11.5764 | 3.53312 | 11.5764 |
| 347 | rno-miR-342-3p | 0.0159055 | 1 | 0.186512 | 1 | 0.163462 | 2.74159 | 7.90448 | 7.62968 | 1.20983 | 0.274805 | 1.20983 |
| 140 | rno-miR-185 | 0.0162544 | 1 | 0.186512 | 1 | 0.163462 | 2.73053 | 5.91852 | 5.58892 | 1.25666 | 0.329599 | 1.25666 |
| 125 | rno-miR-17-5p | 0.0159993 | 1 | 0.186512 | 1 | 0.163462 | 2.73859 | 7.87468 | 7.5126 | 1.28528 | 0.362078 | 1.28528 |
| 491 | rno-miR-411* | 0.016169 | 1 | 0.186512 | 1 | 0.163462 | 2.73321 | 4.40599 | 3.98328 | 1.34044 | 0.422711 | 1.34044 |
| 340 | rno-miR-338 | 0.0153054 | 1 | 0.186512 | 1 | 0.163462 | 2.76117 | 4.45919 | 1.50559 | 7.74679 | 2.9536 | 7.74679 |
| 523 | rno-miR-466b-1* | 0.016561 | 1 | 0.186863 | 1 | 0.16377 | -2.721 | 4.30581 | 4.58763 | 0.822553 | -0.28182 | -1.21573 |
| 669 | rno-miR-92b | 0.0177138 | 1 | 0.196594 | 1 | 0.172298 | -2.68664 | 3.30662 | 3.71194 | 0.755066 | -0.405325 | -1.32439 |
| 325 | rno-miR-328a* | 0.0194932 | 1 | 0.206202 | 1 | 0.180719 | -2.63765 | -3.32193 | -0.0503413 | 0.103551 | -3.27159 | -9.65708 |
| 205 | rno-miR-20b-5p | 0.0192364 | 1 | 0.206202 | 1 | 0.180719 | 2.64445 | 8.10946 | 7.75635 | 1.27731 | 0.353104 | 1.27731 |
| 464 | rno-miR-376a | 0.0190653 | 1 | 0.206202 | 1 | 0.180719 | 2.64903 | 4.26763 | 3.56443 | 1.6281 | 0.703194 | 1.6281 |
| 211 | rno-miR-211* | 0.0198488 | 1 | 0.206733 | 1 | 0.181184 | -2.62839 | 0.919327 | 4.22242 | 0.101314 | -3.30309 | -9.87029 |
| 65 | rno-miR-130a | 0.0212013 | 1 | 0.217474 | 1 | 0.190597 | 2.59454 | 11.3487 | 11.0448 | 1.2345 | 0.303925 | 1.2345 |
| 592 | rno-miR-652 | 0.0236085 | 1 | 0.235043 | 1 | 0.205996 | 2.53916 | 8.99739 | 8.73076 | 1.20299 | 0.266628 | 1.20299 |
| 72 | rno-miR-133a* | 0.0233301 | 1 | 0.235043 | 1 | 0.205996 | 2.54528 | 8.52797 | 8.23999 | 1.22093 | 0.287979 | 1.22093 |
| 448 | rno-miR-361 | 0.024568 | 1 | 0.241051 | 1 | 0.211261 | 2.51858 | 6.92472 | 6.63205 | 1.2249 | 0.292667 | 1.2249 |
| 305 | rno-miR-30e* | 0.0249859 | 1 | 0.241649 | 1 | 0.211785 | 2.50986 | 6.48598 | 6.18562 | 1.23146 | 0.300367 | 1.23146 |
| 15 | rno-let-7f | 0.0268341 | 1 | 0.255869 | 1 | 0.224248 | 2.4729 | 11.2686 | 10.9992 | 1.20531 | 0.269401 | 1.20531 |
| 71 | rno-miR-133a | 0.0282762 | 1 | 0.262233 | 1 | 0.229825 | 2.44571 | 8.78712 | 8.5124 | 1.20976 | 0.274719 | 1.20976 |
| 250 | rno-miR-26b | 0.0281034 | 1 | 0.262233 | 1 | 0.229825 | 2.4489 | 8.55309 | 8.26148 | 1.22401 | 0.291618 | 1.22401 |
| 517 | rno-miR-455* | 0.0292449 | 1 | 0.262237 | 1 | 0.229828 | 2.42818 | 4.00058 | 3.82793 | 1.12713 | 0.17265 | 1.12713 |
| 296 | rno-miR-30a* | 0.0296907 | 1 | 0.262237 | 1 | 0.229828 | 2.4203 | 6.3766 | 6.0942 | 1.21621 | 0.282395 | 1.21621 |
| 24 | rno-miR-101a | 0.0290635 | 1 | 0.262237 | 1 | 0.229828 | 2.43142 | 5.06287 | 4.75251 | 1.24002 | 0.310359 | 1.24002 |
| 358 | rno-miR-347 | 0.029826 | 1 | 0.262237 | 1 | 0.229828 | 2.41793 | 6.20772 | 5.77337 | 1.3513 | 0.434351 | 1.3513 |
| 32 | rno-miR-106b | 0.0313256 | 1 | 0.268448 | 1 | 0.235272 | 2.39233 | 9.18239 | 8.89124 | 1.22362 | 0.291158 | 1.22362 |
| 98 | rno-miR-144 | 0.0309729 | 1 | 0.268448 | 1 | 0.235272 | 2.39824 | 5.78611 | 5.38406 | 1.32138 | 0.402046 | 1.32138 |
| 90 | rno-miR-140 | 0.0321962 | 1 | 0.269096 | 1 | 0.23584 | 2.378 | 6.48603 | 6.21996 | 1.20253 | 0.266068 | 1.20253 |
| 318 | rno-miR-324-5p | 0.0321315 | 1 | 0.269096 | 1 | 0.23584 | 2.37905 | 7.4817 | 7.19501 | 1.21984 | 0.286692 | 1.21984 |
| 310 | rno-miR-32* | 0.0338191 | 1 | 0.272455 | 1 | 0.238783 | -2.35223 | -2.42853 | 1.54134 | 0.063819 | -3.96987 | -15.6693 |
| 109 | rno-miR-150 | 0.034641 | 1 | 0.272455 | 1 | 0.238783 | -2.33963 | 5.37936 | 5.67734 | 0.813389 | -0.297982 | -1.22942 |
| 410 | rno-miR-3573-3p | 0.0338317 | 1 | 0.272455 | 1 | 0.238783 | -2.35204 | 4.53591 | 4.66165 | 0.916532 | -0.125743 | -1.09107 |
| 111 | rno-miR-151 | 0.0351213 | 1 | 0.272455 | 1 | 0.238783 | 2.3324 | 7.7361 | 7.48517 | 1.18997 | 0.250929 | 1.18997 |
| 333 | rno-miR-331 | 0.034916 | 1 | 0.272455 | 1 | 0.238783 | 2.33548 | 5.71784 | 5.45409 | 1.20059 | 0.263744 | 1.20059 |
| 313 | rno-miR-322 | 0.0354151 | 1 | 0.272455 | 1 | 0.238783 | 2.32802 | 10.0293 | 9.76001 | 1.20523 | 0.269308 | 1.20523 |
| 231 | rno-miR-221 | 0.0348262 | 1 | 0.272455 | 1 | 0.238783 | 2.33683 | 1.60503 | -1.81362 | 10.6934 | 3.41865 | 10.6934 |
| 54 | rno-miR-125b-5p | 0.0359358 | 1 | 0.273354 | 1 | 0.239572 | 2.32034 | 10.2493 | 9.98986 | 1.19704 | 0.259476 | 1.19704 |
| 256 | rno-miR-28 | 0.0365054 | 1 | 0.274601 | 1 | 0.240665 | 2.31206 | 6.23774 | 5.96314 | 1.20966 | 0.2746 | 1.20966 |
| 246 | rno-miR-25 | 0.0372948 | 1 | 0.277457 | 1 | 0.243167 | 2.30078 | 8.4451 | 8.17392 | 1.2068 | 0.271181 | 1.2068 |
| 20 | rno-miR-1 | 0.0378504 | 1 | 0.278529 | 1 | 0.244108 | 2.29298 | 13.0444 | 12.8108 | 1.17578 | 0.233618 | 1.17578 |
| 676 | rno-miR-98 | 0.0385695 | 1 | 0.280769 | 1 | 0.246071 | 2.28304 | 7.28747 | 7.01445 | 1.20834 | 0.273026 | 1.20834 |
| 119 | rno-miR-15b | 0.0397329 | 1 | 0.281624 | 1 | 0.24682 | 2.26733 | 10.2159 | 9.98002 | 1.17766 | 0.235926 | 1.17766 |
| 671 | rno-miR-93 | 0.0399265 | 1 | 0.281624 | 1 | 0.24682 | 2.26476 | 8.11101 | 7.82844 | 1.21637 | 0.282577 | 1.21637 |
| 516 | rno-miR-455 | 0.0399349 | 1 | 0.281624 | 1 | 0.24682 | 2.26464 | 4.65251 | 4.33412 | 1.24694 | 0.318392 | 1.24694 |
| 317 | rno-miR-324-3p | 0.042823 | 1 | 0.295828 | 1 | 0.259268 | 2.2276 | 8.60312 | 8.25047 | 1.2769 | 0.352649 | 1.2769 |
| 514 | rno-miR-451 | 0.0426575 | 1 | 0.295828 | 1 | 0.259268 | 2.22966 | 8.41217 | 8.04197 | 1.29254 | 0.370205 | 1.29254 |
| 34 | rno-miR-107 | 0.0433761 | 1 | 0.296622 | 1 | 0.259964 | 2.22077 | 10.0872 | 9.82146 | 1.20228 | 0.265773 | 1.20228 |
| 207 | rno-miR-21* | 0.0442768 | 1 | 0.296786 | 1 | 0.260108 | 2.20983 | -0.154516 | -2.66625 | 5.70306 | 2.51174 | 5.70306 |
| 480 | rno-miR-382 | 0.0439654 | 1 | 0.296786 | 1 | 0.260108 | 2.21358 | 0.455868 | -2.54267 | 7.99192 | 2.99854 | 7.99192 |
| 431 | rno-miR-3588 | 0.0454321 | 1 | 0.301545 | 1 | 0.264278 | -2.19609 | -1.57815 | 2.03589 | 0.0816705 | -3.61404 | -12.2443 |
| 67 | rno-miR-130b | 0.0477438 | 1 | 0.303869 | 1 | 0.266316 | 2.16955 | 8.37507 | 8.12277 | 1.1911 | 0.252294 | 1.1911 |
| 107 | rno-miR-148b-3p | 0.0480266 | 1 | 0.303869 | 1 | 0.266316 | 2.16639 | 6.48579 | 6.2282 | 1.19548 | 0.257594 | 1.19548 |
| 298 | rno-miR-30b-5p | 0.0463641 | 1 | 0.303869 | 1 | 0.266316 | 2.18524 | 8.60128 | 8.33201 | 1.2052 | 0.269276 | 1.2052 |
| 113 | rno-miR-152 | 0.0477632 | 1 | 0.303869 | 1 | 0.266316 | 2.16934 | 7.05992 | 6.75666 | 1.23393 | 0.303261 | 1.23393 |
| 280 | rno-miR-29b | 0.0467986 | 1 | 0.303869 | 1 | 0.266316 | 2.18026 | 5.22929 | 4.89872 | 1.25751 | 0.330567 | 1.25751 |
| Column | Probeset ID | p-value | bonferroni(p-value) | stepup(p-value) | stepdown(p-value) | qvalue(p-value) | t | Mean(HHR) | Mean(NHR) | MeanRatio(HHR/NHR) | MeanDiff(HHR-NHR) | FoldChange(HHR/NHR) |

## S1B Table: Genes Microarray results

**S!B Data — mRNA differential expression (HHR vs NHR)**

| Gene Symbol | NHR mean | HHR mean | Raw p value | Adj p value | Δttt-vs-Con | Fold change | FDR |
| --- | --- | --- | --- | --- | --- | --- | --- |
| MED22 | 9.196452 | 7.5823345 | 3.10862e-15 | 1.73337e-11 | -1.61 | -3.06 | 8.66685e-12 |
| LCN2 | 9.300116 | 6.9798326 | 3.10862e-15 | 1.73337e-11 | -2.32 | -4.99 | 8.66685e-12 |
| RTN1 | 10.230621 | 9.327074 | 1.77414e-13 | 9.89081e-10 | -0.90 | -1.87 | 3.29753e-10 |
| --- | 7.5404572 | 5.7876396 | 1.73062e-12 | 9.64645e-09 | -1.75 | -3.37 | 2.41248e-09 |
| --- | 7.4121265 | 9.041661 | 2.40563e-12 | 1.34066e-08 | 1.63 | 3.09 | 2.68276e-09 |
| ANKRD53 | 8.407183 | 6.2483463 | 2.6481e-12 | 1.47552e-08 | -2.16 | -4.47 | 2.46097e-09 |
| --- | 11.074302 | 10.44118 | 5.1521e-12 | 2.87024e-08 | -0.63 | -1.55 | 4.10402e-09 |
| TLE6 | 7.9822607 | 6.973355 | 1.74798e-11 | 9.73625e-08 | -1.01 | -2.01 | 1.21834e-08 |
| SERHL2 | 9.710146 | 8.520801 | 3.33451e-11 | 1.85699e-07 | -1.19 | -2.28 | 2.06591e-08 |
| LAMA4 | 10.959502 | 11.320265 | 5.01466e-11 | 2.79216e-07 | 0.36 | 1.28 | 2.79617e-08 |
| WDR46 | 9.648229 | 8.304768 | 5.19618e-11 | 2.89271e-07 | -1.34 | -2.54 | 2.63399e-08 |
| CD1D1 | 9.545301 | 8.572607 | 8.44136e-11 | 4.69846e-07 | -0.97 | -1.96 | 3.92242e-08 |
| RT1-DMA | 9.5968075 | 8.969004 | 1.00708e-10 | 5.60438e-07 | -0.63 | -1.55 | 4.31958e-08 |
| CHTOP | 9.495099 | 10.142154 | 1.5819e-10 | 8.80167e-07 | 0.65 | 1.57 | 6.30047e-08 |
| CROT | 10.312456 | 9.801996 | 2.578e-10 | 1.43414e-06 | -0.51 | -1.42 | 9.5833e-08 |
| ENDOG | 7.5098705 | 8.722677 | 2.91683e-10 | 1.62234e-06 | 1.21 | 2.32 | 1.01652e-07 |
| SLC16A12 | 11.125715 | 10.326303 | 4.20618e-10 | 2.33906e-06 | -0.80 | -1.74 | 1.37963e-07 |
| AGTPBP1 | 8.606415 | 9.381156 | 4.2755e-10 | 2.37718e-06 | 0.77 | 1.71 | 1.32445e-07 |
| PARD3B | 8.442211 | 8.981394 | 4.6127e-10 | 2.5642e-06 | 0.54 | 1.45 | 1.35371e-07 |
| LOC100911237 | 5.3110566 | 6.856994 | 5.08819e-10 | 2.82801e-06 | 1.55 | 2.92 | 1.41859e-07 |
| HTR2A | 8.202976 | 6.533735 | 6.45207e-10 | 3.58541e-06 | -1.67 | -3.18 | 1.71318e-07 |
| ITGA7 | 7.5856223 | 8.497929 | 6.71682e-10 | 3.73186e-06 | 0.91 | 1.88 | 1.70241e-07 |
| VNN1 | 10.057846 | 9.036256 | 7.99419e-10 | 4.44077e-06 | -1.02 | -2.03 | 1.93807e-07 |
| AKR1B10 | 9.877308 | 8.386092 | 9.5405e-10 | 5.29879e-06 | -1.49 | -2.81 | 2.21658e-07 |
| NOSTRIN | 7.3632345 | 8.022683 | 1.20082e-09 | 6.66817e-06 | 0.66 | 1.58 | 2.67832e-07 |
| BCMO1 | 6.9873977 | 7.7363625 | 1.37339e-09 | 7.62506e-06 | 0.75 | 1.68 | 2.94539e-07 |
| TRPM8 | 5.5941195 | 7.0771 | 1.48338e-09 | 8.23424e-06 | 1.48 | 2.80 | 3.06345e-07 |
| PEX3 | 10.09458 | 8.85657 | 1.58617e-09 | 8.80323e-06 | -1.24 | -2.36 | 3.15874e-07 |
| PLAT | 11.575467 | 11.122178 | 1.72888e-09 | 9.59358e-06 | -0.45 | -1.37 | 3.32423e-07 |
| NIP30 | 7.2273583 | 8.227325 | 1.86235e-09 | 1.03323e-05 | 1.00 | 2.00 | 3.46149e-07 |
| NDUFA10L1 | 11.817343 | 12.319465 | 2.08392e-09 | 1.15595e-05 | 0.50 | 1.42 | 3.74838e-07 |
| DCPS | 9.798649 | 9.45303 | 2.40504e-09 | 1.33359e-05 | -0.35 | -1.27 | 4.06378e-07 |
| MRPL19 | 8.835422 | 7.959924 | 2.37892e-09 | 1.31935e-05 | -0.88 | -1.83 | 4.14527e-07 |
| ITGBL1 | 9.136594 | 7.7676435 | 2.42737e-09 | 1.34574e-05 | -1.37 | -2.58 | 3.98089e-07 |
| PYGL | 7.583342 | 6.7295747 | 2.7417e-09 | 1.51972e-05 | -0.85 | -1.81 | 4.36791e-07 |
| GINM1 | 9.244288 | 8.605594 | 2.99285e-09 | 1.65864e-05 | -0.64 | -1.56 | 4.63559e-07 |
| NCAM1 | 11.434487 | 11.978191 | 4.17316e-09 | 2.31235e-05 | 0.54 | 1.46 | 6.28906e-07 |
| NT5C3A | 10.241245 | 8.864279 | 5.40322e-09 | 2.99338e-05 | -1.38 | -2.60 | 7.92851e-07 |
| CACNA2D2 | 10.027779 | 9.423711 | 6.40355e-09 | 3.54693e-05 | -0.60 | -1.52 | 9.15544e-07 |
| BPHL | 9.7793665 | 8.7095785 | 7.48209e-09 | 4.14358e-05 | -1.07 | -2.10 | 1.043e-06 |
| TRIM55 | 11.899503 | 12.467405 | 7.512e-09 | 4.1594e-05 | 0.57 | 1.48 | 1.02163e-06 |
| GSTT3 | 6.993923 | 7.7204566 | 7.64016e-09 | 4.22959e-05 | 0.73 | 1.65 | 1.01432e-06 |
| SGPP1 | 9.819835 | 9.164109 | 7.70127e-09 | 4.26188e-05 | -0.66 | -1.58 | 9.7596e-07 |
| HCFC1R1 | 9.919163 | 9.580156 | 7.67071e-09 | 4.24574e-05 | -0.34 | -1.26 | 9.94695e-07 |
| RT1-DMB | 9.424288 | 8.688017 | 8.01565e-09 | 4.43506e-05 | -0.74 | -1.67 | 9.93228e-07 |
| SEMA3D | 9.393354 | 8.862589 | 8.42603e-09 | 4.66128e-05 | -0.53 | -1.44 | 1.02138e-06 |
| PTPN13 | 9.254508 | 9.809116 | 8.58946e-09 | 4.75083e-05 | 0.55 | 1.47 | 1.01904e-06 |
| NOP16 | 8.846183 | 8.277815 | 8.8884e-09 | 4.91529e-05 | -0.57 | -1.48 | 1.03254e-06 |
| UHRF1BP1L | 8.930024 | 9.296183 | 9.47717e-09 | 5.23993e-05 | 0.37 | 1.29 | 1.07846e-06 |
| GNG11 | 9.023382 | 9.490152 | 9.87538e-09 | 5.45911e-05 | 0.47 | 1.38 | 1.1013e-06 |
| ACACB | 9.800518 | 10.3200245 | 1.09741e-08 | 6.0654e-05 | 0.52 | 1.43 | 1.19984e-06 |
| PLA2G5 | 10.799519 | 10.024248 | 1.15364e-08 | 6.375e-05 | -0.78 | -1.71 | 1.23705e-06 |
| --- | 12.331586 | 11.718531 | 1.23868e-08 | 6.84372e-05 | -0.61 | -1.53 | 1.30319e-06 |
| ATP6V1D | 10.892199 | 10.04476 | 1.37211e-08 | 7.57952e-05 | -0.85 | -1.80 | 1.41683e-06 |
| NCLN | 8.5669985 | 9.103787 | 1.44955e-08 | 8.00585e-05 | 0.54 | 1.45 | 1.46958e-06 |
| RT1-DB2 | 8.214402 | 6.9563746 | 1.52181e-08 | 8.40342e-05 | -1.26 | -2.39 | 1.51529e-06 |
| SLC38A4 | 10.418651 | 9.664789 | 1.70887e-08 | 9.43465e-05 | -0.75 | -1.69 | 1.67169e-06 |
| --- | 10.70479 | 10.23898 | 1.78043e-08 | 9.82795e-05 | -0.47 | -1.38 | 1.71166e-06 |
| FUOM | 8.421793 | 7.797205 | 1.86008e-08 | 0.000102639 | -0.62 | -1.54 | 1.72864e-06 |
| --- | 9.175125 | 9.481117 | 1.8538e-08 | 0.000102311 | 0.31 | 1.24 | 1.75199e-06 |
| DAP | 11.068121 | 11.485004 | 2.00733e-08 | 0.000110744 | 0.42 | 1.34 | 1.8349e-06 |
| --- | 8.169458 | 7.181369 | 2.14384e-08 | 0.000118254 | -0.99 | -1.98 | 1.92807e-06 |
| ADCY1 | 8.086979 | 8.671874 | 2.40713e-08 | 0.000132753 | 0.58 | 1.50 | 2.1305e-06 |
| THEM4 | 10.302552 | 9.774259 | 2.43391e-08 | 0.000134206 | -0.53 | -1.44 | 2.12054e-06 |
| HEBP2 | 7.7047076 | 6.6997213 | 2.57527e-08 | 0.000141975 | -1.00 | -2.01 | 2.20919e-06 |
| TEX261 | 11.879395 | 11.329164 | 2.60992e-08 | 0.000143859 | -0.55 | -1.46 | 2.20499e-06 |
| IDUA | 10.9184475 | 11.390997 | 2.70683e-08 | 0.000149173 | 0.47 | 1.39 | 2.25273e-06 |
| --- | 9.525759 | 8.546458 | 3.14496e-08 | 0.000173287 | -0.98 | -1.97 | 2.57887e-06 |
| STIM1 | 8.946586 | 9.502931 | 3.40241e-08 | 0.000187439 | 0.56 | 1.47 | 2.74954e-06 |
| PIAS3 | 9.356661 | 8.840903 | 3.89001e-08 | 0.000214262 | -0.52 | -1.43 | 3.09867e-06 |
| PPAPDC1A | 7.860985 | 7.365909 | 4.30372e-08 | 0.000237006 | -0.50 | -1.41 | 3.37993e-06 |
| DYNC1H1 | 10.890987 | 11.208169 | 4.5003e-08 | 0.000247786 | 0.32 | 1.25 | 3.48523e-06 |
| GRM4 | 6.8071995 | 7.0510616 | 5.5272e-08 | 0.000304272 | 0.24 | 1.18 | 4.22187e-06 |
| GLOD5 | 7.955223 | 7.0493507 | 5.79886e-08 | 0.000319169 | -0.91 | -1.87 | 4.36952e-06 |
| AHI1 | 8.452871 | 8.891129 | 6.81474e-08 | 0.000375015 | 0.44 | 1.35 | 5.06653e-06 |
| DSP | 12.235811 | 12.516594 | 7.22334e-08 | 0.000397283 | 0.28 | 1.21 | 5.16376e-06 |
| QSOX1 | 8.253165 | 8.71481 | 6.98676e-08 | 0.000384411 | 0.46 | 1.38 | 5.12607e-06 |
| CCDC92 | 7.6928167 | 7.2724495 | 7.00863e-08 | 0.000385545 | -0.42 | -1.34 | 5.07534e-06 |
| CYSLTR2 | 6.419883 | 5.7579403 | 7.73784e-08 | 0.000425504 | -0.66 | -1.58 | 5.46154e-06 |
| BTBD11 | 7.482695 | 7.896522 | 8.23074e-08 | 0.000452444 | 0.41 | 1.33 | 5.666e-06 |
| RGD1307100 | 10.939701 | 11.1606245 | 7.9146e-08 | 0.000435145 | 0.22 | 1.17 | 5.51648e-06 |
| ALX3 | 8.104334 | 8.453829 | 9.4607e-08 | 0.00051996 | 0.35 | 1.27 | 6.43328e-06 |
| SEMA3C | 8.681963 | 7.9533744 | 9.70349e-08 | 0.000533207 | -0.73 | -1.66 | 6.51888e-06 |
| MACF1 | 11.267206 | 11.510229 | 1.02421e-07 | 0.000562392 | 0.24 | 1.18 | 6.56435e-06 |
| AFTPH | 8.386679 | 8.8538065 | 9.78105e-08 | 0.000537371 | 0.47 | 1.38 | 6.49276e-06 |
| STX17 | 9.133384 | 8.639085 | 9.84914e-08 | 0.000541013 | -0.49 | -1.41 | 6.46103e-06 |
| MYBPC3 | 12.524393 | 12.768169 | 1.00812e-07 | 0.000553657 | 0.24 | 1.18 | 6.53634e-06 |
| LOC100910098 | 8.099377 | 8.301673 | 1.16248e-07 | 0.000638083 | 0.20 | 1.15 | 7.28311e-06 |
| LOC100910934 | 6.3217235 | 5.114926 | 1.12976e-07 | 0.000620239 | -1.21 | -2.31 | 7.15858e-06 |
| RIIAD1 | 4.9473886 | 6.0106134 | 1.1864e-07 | 0.000651098 | 1.06 | 2.09 | 7.35043e-06 |
| ARL6IP6 | 10.585709 | 10.170042 | 1.21881e-07 | 0.000668763 | -0.42 | -1.33 | 7.46825e-06 |
| SUMO2 | 13.008054 | 12.726736 | 1.23819e-07 | 0.00067927 | -0.28 | -1.22 | 7.50449e-06 |
| NSMAF | 9.049277 | 9.327886 | 1.25433e-07 | 0.000687998 | 0.28 | 1.21 | 7.52056e-06 |
| FBXL3-PS1 | 7.1333838 | 7.4854574 | 1.28779e-07 | 0.000706226 | 0.35 | 1.28 | 7.63909e-06 |
| IFI30 | 10.228568 | 9.802651 | 1.30493e-07 | 0.000715494 | -0.43 | -1.34 | 7.65926e-06 |
| FTH1 | 13.103566 | 13.325459 | 1.42947e-07 | 0.000783491 | 0.22 | 1.17 | 8.21722e-06 |
| ISCA1 | 10.079782 | 10.533627 | 1.40035e-07 | 0.000767671 | 0.45 | 1.37 | 8.13369e-06 |
| LMO7 | 10.45668 | 10.845891 | 1.44652e-07 | 0.00079255 | 0.39 | 1.31 | 8.14728e-06 |
| POLE3 | 8.1680975 | 7.7016993 | 1.43222e-07 | 0.000784859 | -0.47 | -1.38 | 8.14906e-06 |
| NAV3 | 9.821737 | 10.431951 | 1.52972e-07 | 0.00083798 | 0.61 | 1.53 | 8.52971e-06 |
| DUSP27 | 8.995172 | 9.4099245 | 1.60382e-07 | 0.000878253 | 0.41 | 1.33 | 8.76756e-06 |
| TTC4 | 7.5101047 | 7.932221 | 1.6026e-07 | 0.000877746 | 0.42 | 1.34 | 8.84764e-06 |
| LOC679782 | 6.018814 | 6.509481 | 1.62242e-07 | 0.000888278 | 0.49 | 1.41 | 8.78315e-06 |
| PIP5K1B | 7.609256 | 8.157251 | 1.66732e-07 | 0.000912688 | 0.55 | 1.46 | 8.93937e-06 |
| AHNAK | 9.948292 | 10.303992 | 1.71311e-07 | 0.000937584 | 0.36 | 1.28 | 9.09742e-06 |
| GSTK1 | 10.585458 | 10.094704 | 1.82151e-07 | 0.000996733 | -0.49 | -1.41 | 9.58185e-06 |
| NMRAL1 | 7.9408636 | 8.509572 | 1.84479e-07 | 0.001009284 | 0.57 | 1.48 | 9.61359e-06 |
| LOC102556952 | 9.684808 | 9.118885 | 1.87902e-07 | 0.001027827 | -0.57 | -1.48 | 9.70134e-06 |
| LOC100359752 | 5.7425714 | 4.9283786 | 2.00673e-07 | 0.001097479 | -0.81 | -1.76 | 1.02656e-05 |
| ZFYVE26 | 7.9998803 | 8.450327 | 2.05843e-07 | 0.00112555 | 0.45 | 1.37 | 1.04344e-05 |
| ALDH1A1 | 6.226587 | 5.8359594 | 2.21223e-07 | 0.001209426 | -0.39 | -1.31 | 1.1113e-05 |
| KCNE1L | 8.974249 | 8.311703 | 2.26063e-07 | 0.001235658 | -0.66 | -1.58 | 1.12547e-05 |
| CYB5R1 | 8.651947 | 9.075983 | 2.33298e-07 | 0.001274742 | 0.42 | 1.34 | 1.14111e-05 |
| CAR4 | 8.000295 | 9.035868 | 2.31374e-07 | 0.00126446 | 1.04 | 2.05 | 1.14172e-05 |
| GPR83 | 6.468145 | 7.210346 | 2.43168e-07 | 0.001328428 | 0.74 | 1.67 | 1.17905e-05 |
| ABCA4 | 6.6066904 | 6.1144514 | 2.45041e-07 | 0.001338412 | -0.49 | -1.41 | 1.17788e-05 |
| FRRS1 | 9.422919 | 9.012072 | 2.49748e-07 | 0.001363871 | -0.41 | -1.33 | 1.19025e-05 |
| ARHGEF5 | 7.756139 | 8.259307 | 2.79862e-07 | 0.001528047 | 0.50 | 1.42 | 1.32247e-05 |
| SPTAN1 | 10.989853 | 11.240639 | 3.10027e-07 | 0.001692435 | 0.25 | 1.19 | 1.4527e-05 |
| ZFP347 | 7.4468746 | 6.172405 | 3.13329e-07 | 0.00171015 | -1.27 | -2.42 | 1.45594e-05 |
| PCSK6 | 8.714376 | 8.329027 | 3.14062e-07 | 0.001713836 | -0.39 | -1.31 | 1.44728e-05 |
| TEP1 | 9.201345 | 9.581713 | 3.27991e-07 | 0.00178952 | 0.38 | 1.30 | 1.49908e-05 |
| CXADR | 12.281618 | 11.799686 | 3.3356e-07 | 0.001819571 | -0.48 | -1.40 | 1.51214e-05 |
| ADAR | 9.468182 | 9.84371 | 3.51328e-07 | 0.001916144 | 0.38 | 1.30 | 1.57984e-05 |
| FAM69B | 9.0919 | 8.509503 | 3.5675e-07 | 0.001945356 | -0.58 | -1.50 | 1.59139e-05 |
| TMEM128 | 10.39341 | 10.0636 | 3.5729e-07 | 0.001947942 | -0.33 | -1.26 | 1.58115e-05 |
| FBXO38 | 9.230391 | 9.676544 | 3.7207e-07 | 0.002028153 | 0.45 | 1.36 | 1.63359e-05 |
| LRBA | 9.197462 | 9.430483 | 3.95302e-07 | 0.002153606 | 0.23 | 1.18 | 1.69554e-05 |
| ZC3H7A | 8.583909 | 8.909246 | 3.91439e-07 | 0.002132949 | 0.33 | 1.25 | 1.69199e-05 |
| ATP5G2 | 12.332418 | 12.146557 | 3.8686e-07 | 0.00210839 | -0.19 | -1.14 | 1.68526e-05 |
| PSMB9 | 7.369558 | 6.5544167 | 4.10909e-07 | 0.00223822 | -0.82 | -1.76 | 1.74903e-05 |
| BMP4 | 9.098457 | 8.2636175 | 4.15883e-07 | 0.002264897 | -0.83 | -1.78 | 1.75679e-05 |
| ENPP1 | 10.734014 | 10.369867 | 4.22291e-07 | 0.002299375 | -0.36 | -1.29 | 1.77045e-05 |
| RGD1563520 | 8.5837 | 8.310575 | 4.29635e-07 | 0.002338934 | -0.27 | -1.21 | 1.7878e-05 |
| LOC102547503 | 8.088159 | 8.456189 | 4.54424e-07 | 0.002473432 | 0.37 | 1.29 | 1.87694e-05 |
| TFAM | 9.177491 | 8.893112 | 4.66194e-07 | 0.002537028 | -0.28 | -1.22 | 1.9114e-05 |
| NHEJ1 | 7.717357 | 7.38249 | 4.68384e-07 | 0.00254848 | -0.33 | -1.26 | 1.90636e-05 |
| GPRC5C | 7.5913777 | 7.1553173 | 4.78859e-07 | 0.002604517 | -0.44 | -1.35 | 1.92095e-05 |
| FGF12 | 8.51943 | 8.124773 | 4.85306e-07 | 0.002639095 | -0.39 | -1.31 | 1.93291e-05 |
| FTH1 | 13.094779 | 13.307764 | 4.77368e-07 | 0.002596884 | 0.21 | 1.16 | 1.92884e-05 |
| LGALS1 | 12.6039715 | 12.280596 | 5.00467e-07 | 0.002721038 | -0.32 | -1.25 | 1.97915e-05 |
| STX4 | 9.802538 | 10.130008 | 5.1871e-07 | 0.00281919 | 0.33 | 1.25 | 2.02261e-05 |
| SUMO2L // SUMO2 | 10.948736 | 10.606075 | 5.17e-07 | 0.002810411 | -0.34 | -1.27 | 2.03013e-05 |
| LRRK2 | 8.650394 | 9.047719 | 5.32297e-07 | 0.002892503 | 0.40 | 1.32 | 2.06117e-05 |
| POP5 | 7.5790377 | 8.838769 | 5.43986e-07 | 0.002955475 | 1.26 | 2.39 | 2.09191e-05 |
| MNDA | 7.4274006 | 6.746208 | 5.5075e-07 | 0.002991676 | -0.68 | -1.60 | 2.10341e-05 |
| MAP1A | 7.865323 | 8.416617 | 5.59112e-07 | 0.003036536 | 0.55 | 1.47 | 2.12082e-05 |
| KCNT2 | 5.978503 | 6.6573806 | 5.66996e-07 | 0.003078787 | 0.68 | 1.60 | 2.13619e-05 |
| UBC // UBB | 9.987602 | 10.940694 | 5.72734e-07 | 0.00310937 | 0.95 | 1.94 | 2.14333e-05 |
| TMCO4 | 7.517955 | 7.831277 | 5.93393e-07 | 0.003220936 | 0.31 | 1.24 | 2.20584e-05 |
| DNAJB2 | 9.803582 | 10.13983 | 6.31476e-07 | 0.003427019 | 0.34 | 1.26 | 2.33186e-05 |
| PRR16 | 9.480521 | 8.7231245 | 6.83732e-07 | 0.003709247 | -0.76 | -1.69 | 2.49182e-05 |
| B4GALT6 | 10.825698 | 11.051336 | 6.80534e-07 | 0.003692579 | 0.23 | 1.17 | 2.49649e-05 |
| TSPYL2 | 8.0020485 | 8.551863 | 6.92664e-07 | 0.00375701 | 0.55 | 1.46 | 2.50798e-05 |
| PIGN | 9.28945 | 8.717175 | 7.39325e-07 | 0.004008621 | -0.57 | -1.49 | 2.64261e-05 |
| CDH13 | 11.195865 | 10.603629 | 7.35458e-07 | 0.003988391 | -0.59 | -1.51 | 2.64575e-05 |
| RMDN1 | 9.621505 | 9.123819 | 7.43546e-07 | 0.004030764 | -0.50 | -1.41 | 2.64077e-05 |
| HEXB | 9.620882 | 9.093806 | 7.50801e-07 | 0.00406934 | -0.53 | -1.44 | 2.64966e-05 |
| GANC | 8.834892 | 9.366109 | 7.5726e-07 | 0.00410359 | 0.53 | 1.45 | 2.65565e-05 |
| CCNE2 | 9.599725 | 9.044984 | 7.61692e-07 | 0.004125326 | -0.55 | -1.47 | 2.62173e-05 |
| PDPN | 9.939703 | 9.546302 | 7.60303e-07 | 0.004118562 | -0.39 | -1.31 | 2.6332e-05 |
| TXLNB | 11.232025 | 11.902616 | 7.57499e-07 | 0.004104131 | 0.67 | 1.59 | 2.63989e-05 |
| LOC100363068 // LOC100366030 | 6.9286118 | 7.317533 | 7.70558e-07 | 0.004172573 | 0.39 | 1.31 | 2.63597e-05 |
| FKBP8 | 10.1982765 | 10.456349 | 7.93702e-07 | 0.0042971 | 0.26 | 1.20 | 2.69859e-05 |
| CASP12 | 7.047124 | 6.5656447 | 8.02669e-07 | 0.004344846 | -0.48 | -1.40 | 2.71253e-05 |
| KTI12 | 9.868358 | 9.553316 | 8.55237e-07 | 0.004627686 | -0.32 | -1.24 | 2.85557e-05 |
| --- | 11.252486 | 11.963654 | 8.50937e-07 | 0.004605271 | 0.71 | 1.64 | 2.85833e-05 |
| CDH8 | 5.0978575 | 5.721212 | 8.60718e-07 | 0.004656485 | 0.62 | 1.54 | 2.85676e-05 |
| CXXC4 | 8.243349 | 9.086365 | 8.70051e-07 | 0.004706108 | 0.84 | 1.79 | 2.87065e-05 |
| AXL | 9.776362 | 9.377737 | 8.71853e-07 | 0.00471498 | -0.40 | -1.32 | 2.85968e-05 |
| TMEFF1 | 9.59609 | 9.905221 | 8.74586e-07 | 0.004728886 | 0.31 | 1.24 | 2.85187e-05 |
| MTR | 8.96826 | 9.250707 | 9.12304e-07 | 0.004931003 | 0.28 | 1.22 | 2.94047e-05 |
| SERPINB6 | 8.282796 | 8.752407 | 9.07523e-07 | 0.004906069 | 0.47 | 1.38 | 2.94206e-05 |
| RALY | 10.858049 | 11.049502 | 9.31665e-07 | 0.005034719 | 0.19 | 1.14 | 2.98561e-05 |
| HEY2 | 10.683478 | 11.292837 | 1.00409e-06 | 0.005425104 | 0.61 | 1.53 | 3.19932e-05 |
| RNF39 | 7.4675775 | 7.810063 | 1.02414e-06 | 0.00553239 | 0.34 | 1.27 | 3.24465e-05 |
| MED12L | 8.62088 | 8.35144 | 1.08449e-06 | 0.005857312 | -0.27 | -1.21 | 3.41644e-05 |
| PLEC | 9.166965 | 9.526959 | 1.10555e-06 | 0.005969949 | 0.36 | 1.28 | 3.46322e-05 |
| PLSCR1 | 7.537378 | 7.033655 | 1.10721e-06 | 0.00597785 | -0.50 | -1.42 | 3.44907e-05 |
| SHISA4 | 11.893019 | 11.555631 | 1.12577e-06 | 0.006076896 | -0.34 | -1.26 | 3.48738e-05 |
| CLIC5 | 11.642771 | 11.234562 | 1.13722e-06 | 0.006136422 | -0.41 | -1.33 | 3.48413e-05 |
| --- | 8.899417 | 8.53726 | 1.13231e-06 | 0.006111102 | -0.36 | -1.29 | 3.48828e-05 |
| L3HYPDH | 8.507028 | 9.11861 | 1.15386e-06 | 0.006225067 | 0.61 | 1.53 | 3.5158e-05 |
| AARSD1 | 9.976402 | 9.162762 | 1.16194e-06 | 0.006267494 | -0.81 | -1.76 | 3.52118e-05 |
| ROBO1 | 9.076901 | 8.706955 | 1.17792e-06 | 0.006351338 | -0.37 | -1.29 | 3.53122e-05 |
| EFNB3 | 10.373609 | 10.61911 | 1.16967e-06 | 0.006308006 | 0.25 | 1.19 | 3.52544e-05 |
| PTPRS | 10.517834 | 10.782505 | 1.20174e-06 | 0.006477353 | 0.26 | 1.20 | 3.5643e-05 |
| DDC | 11.162795 | 11.558553 | 1.20296e-06 | 0.006482774 | 0.40 | 1.32 | 3.54906e-05 |
| EMP2 | 12.255113 | 11.55123 | 1.20841e-06 | 0.006509724 | -0.70 | -1.63 | 3.52781e-05 |
| TRAP1 | 10.055343 | 10.368059 | 1.23341e-06 | 0.006643139 | 0.31 | 1.24 | 3.58202e-05 |
| --- | 8.336813 | 8.024829 | 1.19421e-06 | 0.006438009 | -0.31 | -1.24 | 3.56093e-05 |
| GNG5 | 11.298296 | 11.04755 | 1.20665e-06 | 0.006501449 | -0.25 | -1.19 | 3.54121e-05 |
| SBF1 | 9.2203245 | 9.412195 | 1.25605e-06 | 0.006763835 | 0.19 | 1.14 | 3.62888e-05 |
| CPVL | 5.7491837 | 5.0207815 | 1.2782e-06 | 0.00688185 | -0.73 | -1.66 | 3.67385e-05 |
| AQP3 | 7.003215 | 7.521612 | 1.3304e-06 | 0.007161543 | 0.52 | 1.43 | 3.80426e-05 |
| CYP2U1 | 8.623629 | 8.338781 | 1.33423e-06 | 0.007180848 | -0.28 | -1.22 | 3.79576e-05 |
| NPDC1 | 9.2741585 | 9.602981 | 1.39502e-06 | 0.007505207 | 0.33 | 1.26 | 3.9286e-05 |
| MGC95208 | 10.038619 | 9.385269 | 1.39233e-06 | 0.007492153 | -0.65 | -1.57 | 3.94094e-05 |
| NCF1 | 7.284493 | 7.7364435 | 1.41983e-06 | 0.007634414 | 0.45 | 1.37 | 3.93879e-05 |
| CCNDBP1 | 8.286767 | 8.810909 | 1.42402e-06 | 0.007655532 | 0.52 | 1.44 | 3.93086e-05 |
| RGD1304622 | 8.34845 | 8.677412 | 1.41956e-06 | 0.007634396 | 0.33 | 1.26 | 3.95773e-05 |
| SECISBP2 | 9.0667305 | 9.378356 | 1.43256e-06 | 0.007700033 | 0.31 | 1.24 | 3.93497e-05 |
| ATP1A3 | 11.609556 | 11.303005 | 1.48433e-06 | 0.007976796 | -0.31 | -1.24 | 4.05717e-05 |
| HUWE1 | 10.941788 | 11.076502 | 1.39513e-06 | 0.007504406 | 0.13 | 1.10 | 3.90917e-05 |
| REXO4 | 8.505271 | 9.151595 | 1.51987e-06 | 0.008166263 | 0.65 | 1.57 | 4.13405e-05 |
| RGS6 | 9.459798 | 8.828024 | 1.53267e-06 | 0.008233491 | -0.63 | -1.55 | 4.14862e-05 |
| PIEZO2 | 6.8544083 | 7.3433614 | 1.55329e-06 | 0.008341168 | 0.49 | 1.40 | 4.16401e-05 |
| RGD1562629 | 9.616231 | 9.984305 | 1.54517e-06 | 0.008299125 | 0.37 | 1.29 | 4.16226e-05 |
| DNAJA4 | 8.41282 | 9.005888 | 1.57453e-06 | 0.008453667 | 0.59 | 1.51 | 4.20076e-05 |
| HMGCR | 9.069134 | 9.470068 | 1.59194e-06 | 0.008545521 | 0.40 | 1.32 | 4.22697e-05 |
| CABP1 | 6.6864886 | 7.2136507 | 1.60987e-06 | 0.008640191 | 0.53 | 1.44 | 4.25434e-05 |
| GSAP | 7.459058 | 7.856672 | 1.64827e-06 | 0.008844641 | 0.40 | 1.32 | 4.33527e-05 |
| CEP131 | 7.4335155 | 7.8146214 | 1.6517e-06 | 0.008861355 | 0.38 | 1.30 | 4.32388e-05 |
| --- | 12.839517 | 12.51166 | 1.67867e-06 | 0.009004411 | -0.33 | -1.26 | 4.37397e-05 |
| NEK6 | 9.219949 | 8.805928 | 1.72062e-06 | 0.009227682 | -0.41 | -1.33 | 4.46241e-05 |
| RGD1309762 | 9.485391 | 9.718946 | 1.72811e-06 | 0.0092661 | 0.23 | 1.18 | 4.46107e-05 |
| GSTT1 | 7.3884764 | 7.6968374 | 1.75336e-06 | 0.009399755 | 0.31 | 1.24 | 4.5054e-05 |
| --- | 8.501139 | 9.594768 | 1.77636e-06 | 0.009521293 | 1.09 | 2.13 | 4.54357e-05 |
| RAVER2 | 8.3699465 | 8.039405 | 1.78142e-06 | 0.009546607 | -0.33 | -1.26 | 4.5357e-05 |
| NAT8B | 6.8123884 | 7.4142914 | 1.80569e-06 | 0.009674861 | 0.60 | 1.52 | 4.57659e-05 |
| RFK | 9.93878 | 9.444959 | 1.8206e-06 | 0.009751143 | -0.49 | -1.41 | 4.57283e-05 |
| SMOC2 | 9.193543 | 8.626399 | 1.81973e-06 | 0.009748288 | -0.57 | -1.48 | 4.59132e-05 |
| SEC31B | 7.931714 | 8.208437 | 1.85213e-06 | 0.009918154 | 0.28 | 1.21 | 4.63115e-05 |
| --- | 8.650421 | 8.851234 | 1.88691e-06 | 0.010102511 | 0.20 | 1.15 | 4.69706e-05 |
| GRTP1 // ADPRHL1 | 9.016716 | 9.8722105 | 1.93052e-06 | 0.01033405 | 0.86 | 1.81 | 4.78425e-05 |
| MGC94199 | 9.202948 | 8.760494 | 2.04551e-06 | 0.010947569 | -0.44 | -1.36 | 5.0468e-05 |
| --- | 10.569607 | 11.114339 | 2.05738e-06 | 0.011006975 | 0.54 | 1.46 | 5.03155e-05 |
| SLC4A3 | 9.815681 | 10.087724 | 2.05438e-06 | 0.010992972 | 0.27 | 1.21 | 5.04635e-05 |
| MREG | 6.8757944 | 6.5752563 | 2.07237e-06 | 0.011085117 | -0.30 | -1.23 | 5.04609e-05 |
| SMU1 | 9.638313 | 9.325854 | 2.09996e-06 | 0.011230574 | -0.31 | -1.24 | 5.09103e-05 |
| CHD3 | 8.557723 | 8.879269 | 2.13862e-06 | 0.011435195 | 0.32 | 1.25 | 5.16231e-05 |
| PTGR1 | 10.239323 | 9.74343 | 2.17479e-06 | 0.011624233 | -0.50 | -1.41 | 5.20455e-05 |
| RSBN1L | 9.997884 | 9.670123 | 2.16467e-06 | 0.01157233 | -0.33 | -1.26 | 5.20267e-05 |
| PKIA | 12.507007 | 11.851223 | 2.19212e-06 | 0.011714683 | -0.66 | -1.58 | 5.22361e-05 |
| PTGS1 | 8.420929 | 7.905078 | 2.22172e-06 | 0.011870629 | -0.52 | -1.43 | 5.27161e-05 |
| MGC95208 | 10.0343685 | 9.388945 | 2.28821e-06 | 0.012223613 | -0.65 | -1.56 | 5.40638e-05 |
| DOK5 | 9.828021 | 8.88088 | 2.3044e-06 | 0.012307776 | -0.95 | -1.93 | 5.42165e-05 |
| AEBP1 | 8.4291525 | 8.96595 | 2.32368e-06 | 0.012408475 | 0.54 | 1.45 | 5.44406e-05 |
| PLEKHA2 | 8.274767 | 7.79049 | 2.35827e-06 | 0.012590791 | -0.48 | -1.40 | 5.50197e-05 |
| --- | 8.950853 | 8.226176 | 2.40471e-06 | 0.01283632 | -0.72 | -1.65 | 5.58693e-05 |
| TMEM69 | 9.887997 | 9.498558 | 2.41624e-06 | 0.012895472 | -0.39 | -1.31 | 5.59044e-05 |
| PGAM2 | 10.370867 | 9.807898 | 2.43485e-06 | 0.01299234 | -0.56 | -1.48 | 5.61021e-05 |
| ANKRD13C | 10.055878 | 9.73387 | 2.46588e-06 | 0.013153001 | -0.32 | -1.25 | 5.63514e-05 |
| USP19 | 10.352113 | 10.544347 | 2.45102e-06 | 0.013076179 | 0.19 | 1.14 | 5.62423e-05 |
| PMPCB | 11.152014 | 10.900461 | 2.55835e-06 | 0.013643662 | -0.25 | -1.19 | 5.82259e-05 |
| OBSCN | 7.6457367 | 7.861764 | 2.60556e-06 | 0.013892851 | 0.22 | 1.16 | 5.90594e-05 |
| CHID1 // LOC100911881 | 9.537397 | 8.706803 | 2.61414e-06 | 0.013935969 | -0.83 | -1.78 | 5.90139e-05 |
| ZFP9 | 8.321533 | 8.630175 | 2.6169e-06 | 0.013948084 | 0.31 | 1.24 | 5.88381e-05 |
| FAM86A | 7.0312347 | 7.361878 | 2.7329e-06 | 0.014563615 | 0.33 | 1.26 | 6.11994e-05 |
| CKM | 12.950574 | 13.195189 | 2.81221e-06 | 0.014972179 | 0.24 | 1.18 | 6.17357e-05 |
| RFK | 9.784255 | 9.301546 | 2.78261e-06 | 0.014822947 | -0.48 | -1.40 | 6.1816e-05 |
| PLA2R1 | 7.3311515 | 7.7774863 | 2.7896e-06 | 0.014857383 | 0.45 | 1.36 | 6.17253e-05 |
| CD300LE | 7.21861 | 7.800131 | 2.7955e-06 | 0.014886011 | 0.58 | 1.50 | 6.16114e-05 |
| PITPNC1 | 10.650862 | 11.036202 | 2.77878e-06 | 0.014805338 | 0.39 | 1.31 | 6.19779e-05 |
| LIN52 | 9.209464 | 8.813429 | 2.94144e-06 | 0.0156573 | -0.40 | -1.32 | 6.43196e-05 |
| PHLDB1 | 9.056801 | 9.41039 | 2.94934e-06 | 0.015696399 | 0.35 | 1.28 | 6.42404e-05 |
| KRTAP1-1 | 8.172356 | 7.6177235 | 2.95441e-06 | 0.01572042 | -0.55 | -1.47 | 6.41004e-05 |
| AGPAT9 | 8.153709 | 7.736564 | 2.98239e-06 | 0.01586631 | -0.42 | -1.34 | 6.44566e-05 |
| EPS15 | 10.0009365 | 9.585975 | 3.05395e-06 | 0.016243957 | -0.41 | -1.33 | 6.57484e-05 |
| SLC38A2 | 12.115494 | 11.858383 | 3.15845e-06 | 0.016796652 | -0.26 | -1.20 | 6.77367e-05 |
| --- | 8.357222 | 9.100455 | 3.23661e-06 | 0.017205799 | 0.74 | 1.67 | 6.88829e-05 |
| OTUD6B | 8.934559 | 8.373528 | 3.23649e-06 | 0.017208422 | -0.56 | -1.48 | 6.91443e-05 |
| --- | 8.182553 | 7.3806753 | 3.25931e-06 | 0.017323218 | -0.80 | -1.74 | 6.91023e-05 |
| SDCBP | 11.9429 | 11.752996 | 3.34518e-06 | 0.017769596 | -0.19 | -1.14 | 7.0123e-05 |
| TM4SF1 | 10.745158 | 10.302971 | 3.31177e-06 | 0.017598754 | -0.44 | -1.36 | 6.99486e-05 |
| GNG5 | 11.238813 | 10.989863 | 3.38187e-06 | 0.017957747 | -0.25 | -1.19 | 7.03632e-05 |
| FHOD3 | 10.944496 | 11.30879 | 3.33942e-06 | 0.01774233 | 0.36 | 1.29 | 7.02664e-05 |
| GDAP2 | 9.558214 | 9.05906 | 3.36368e-06 | 0.017864512 | -0.50 | -1.41 | 7.02468e-05 |
| CHRM2 | 12.240865 | 11.957476 | 3.41546e-06 | 0.018125871 | -0.28 | -1.22 | 7.02754e-05 |
| KIF16B | 7.723445 | 8.173714 | 3.40645e-06 | 0.018084865 | 0.45 | 1.37 | 7.06111e-05 |
| POLB | 10.095514 | 9.632892 | 3.4496e-06 | 0.018303577 | -0.46 | -1.38 | 7.07168e-05 |
| SART1 | 8.644193 | 8.929064 | 3.41324e-06 | 0.018117493 | 0.28 | 1.22 | 7.04898e-05 |
| MGC95208 | 9.778639 | 9.040499 | 3.45161e-06 | 0.018310815 | -0.74 | -1.67 | 7.04989e-05 |
| KTN1 | 9.61356 | 9.844773 | 3.57627e-06 | 0.018968541 | 0.23 | 1.17 | 7.27784e-05 |
| DNM2 | 10.192424 | 10.408317 | 3.57856e-06 | 0.018977106 | 0.22 | 1.16 | 7.25602e-05 |
| TNFRSF11B | 8.243535 | 8.84753 | 3.72719e-06 | 0.01976158 | 0.60 | 1.52 | 7.53001e-05 |
| ADORA2A | 8.719325 | 8.437577 | 3.8023e-06 | 0.02015599 | -0.28 | -1.22 | 7.65402e-05 |
| BDH1 | 8.590538 | 7.9280624 | 3.867e-06 | 0.020491224 | -0.66 | -1.58 | 7.72845e-05 |
| RFK | 9.74449 | 9.284327 | 3.86304e-06 | 0.020474123 | -0.46 | -1.38 | 7.74832e-05 |
| SIVA1 | 9.969574 | 9.575505 | 3.87135e-06 | 0.020510422 | -0.39 | -1.31 | 7.70952e-05 |
| RAB6B | 8.14233 | 7.892232 | 3.92145e-06 | 0.020771941 | -0.25 | -1.19 | 7.7815e-05 |
| MRPL35 | 11.561067 | 11.077885 | 3.93921e-06 | 0.02086207 | -0.48 | -1.40 | 7.78903e-05 |
| CORO1A | 6.7785964 | 7.1284494 | 3.94277e-06 | 0.02087695 | 0.35 | 1.27 | 7.7685e-05 |
| CYP2D4 | 7.5478263 | 7.166066 | 4.02726e-06 | 0.0213203 | -0.38 | -1.30 | 7.90704e-05 |
| CEP68 | 9.519478 | 9.231849 | 4.14069e-06 | 0.021912517 | -0.29 | -1.22 | 8.07289e-05 |
| --- | 13.339817 | 13.0172825 | 4.071e-06 | 0.021547787 | -0.32 | -1.25 | 7.96487e-05 |
| FAM212B | 8.367741 | 9.205549 | 4.15763e-06 | 0.021998009 | 0.84 | 1.79 | 8.07768e-05 |
| GPATCH8 | 10.488186 | 10.744874 | 4.18028e-06 | 0.022113686 | 0.26 | 1.19 | 8.09349e-05 |
| --- | 9.4177475 | 8.870866 | 4.18952e-06 | 0.022158368 | -0.55 | -1.46 | 8.08331e-05 |
| MAN2C1 | 8.6315 | 8.893801 | 4.26422e-06 | 0.022536399 | 0.26 | 1.20 | 8.11512e-05 |
| ENOX1 | 6.964547 | 7.226678 | 4.19602e-06 | 0.022188567 | 0.26 | 1.20 | 8.06794e-05 |
| LOC691984 | 10.619924 | 10.158381 | 4.22138e-06 | 0.022318427 | -0.46 | -1.38 | 8.0888e-05 |
| MKKS | 8.261348 | 7.4468846 | 4.24315e-06 | 0.022429287 | -0.81 | -1.76 | 8.10267e-05 |
| SMAD6 | 9.425645 | 8.966252 | 4.27022e-06 | 0.022563841 | -0.46 | -1.37 | 8.09889e-05 |
| LOC362473 | 8.603811 | 8.332296 | 4.32013e-06 | 0.022818945 | -0.27 | -1.21 | 8.1382e-05 |
| UGP2 | 10.979819 | 10.600776 | 4.30103e-06 | 0.022722343 | -0.38 | -1.30 | 8.12968e-05 |
| ABI2 | 10.578308 | 10.446793 | 4.47177e-06 | 0.023601983 | -0.13 | -1.10 | 8.31152e-05 |
| DAD1 | 12.921934 | 12.609495 | 4.39398e-06 | 0.023204584 | -0.31 | -1.24 | 8.24943e-05 |
| APTX | 7.9801397 | 8.257478 | 4.40144e-06 | 0.023239618 | 0.28 | 1.21 | 8.23572e-05 |
| ZBTB20 | 10.533804 | 10.914378 | 4.47154e-06 | 0.023605283 | 0.38 | 1.30 | 8.33891e-05 |
| RPL18A | 14.112875 | 13.878228 | 4.65803e-06 | 0.024561765 | -0.23 | -1.18 | 8.51579e-05 |
| --- | 8.324555 | 8.978979 | 4.58902e-06 | 0.024216278 | 0.65 | 1.57 | 8.50113e-05 |
| PNPLA7 | 6.94256 | 7.256236 | 4.63242e-06 | 0.024436023 | 0.31 | 1.24 | 8.52488e-05 |
| CLIP4 | 9.791122 | 10.086023 | 4.64517e-06 | 0.024498617 | 0.29 | 1.23 | 8.52022e-05 |
| --- | 9.951446 | 9.478404 | 4.62705e-06 | 0.024412323 | -0.47 | -1.39 | 8.54319e-05 |
| USP28 | 11.001574 | 11.216868 | 4.71098e-06 | 0.024836281 | 0.22 | 1.16 | 8.58445e-05 |
| --- | 5.8352356 | 6.382933 | 4.7253e-06 | 0.02490707 | 0.55 | 1.46 | 8.5825e-05 |
| PAQR6 | 9.057886 | 9.386772 | 4.76314e-06 | 0.025096986 | 0.33 | 1.26 | 8.59523e-05 |
| CLDN5 | 8.401675 | 8.799938 | 4.76631e-06 | 0.02510893 | 0.40 | 1.32 | 8.57321e-05 |
| CHKB | 9.276296 | 9.635205 | 4.76004e-06 | 0.0250854 | 0.36 | 1.28 | 8.61752e-05 |
| SLC29A1 | 9.54471 | 9.20396 | 4.85809e-06 | 0.025577858 | -0.34 | -1.27 | 8.65455e-05 |
| --- | 8.595697 | 8.988401 | 4.80934e-06 | 0.025330769 | 0.39 | 1.31 | 8.62278e-05 |
| --- | 9.709048 | 9.146276 | 4.83838e-06 | 0.025478924 | -0.56 | -1.48 | 8.64706e-05 |
| --- | 7.891321 | 7.5187845 | 4.86054e-06 | 0.025585856 | -0.37 | -1.29 | 8.63132e-05 |
| HAGHL | 7.278512 | 6.8420243 | 4.90432e-06 | 0.025811417 | -0.44 | -1.35 | 8.68142e-05 |
| OARD1 | 8.60316 | 8.03263 | 4.91205e-06 | 0.025847204 | -0.57 | -1.49 | 8.66759e-05 |
| CCNE1 | 9.296179 | 8.694505 | 4.91351e-06 | 0.025849994 | -0.60 | -1.52 | 8.64282e-05 |
| --- | 7.122856 | 7.6282897 | 4.92101e-06 | 0.025884487 | 0.51 | 1.42 | 8.62878e-05 |
| PRRG4 | 9.301516 | 8.6408205 | 4.93413e-06 | 0.025948593 | -0.66 | -1.58 | 8.62468e-05 |
| ACOX2 | 5.9197044 | 6.5693045 | 4.94288e-06 | 0.025989674 | 0.65 | 1.57 | 8.61297e-05 |
| ANXA5 | 11.4700165 | 11.147112 | 5.00462e-06 | 0.026309285 | -0.32 | -1.25 | 8.69338e-05 |
| TANK | 9.519432 | 9.030318 | 5.05469e-06 | 0.026562413 | -0.49 | -1.40 | 8.726e-05 |
| TRAM1 | 11.272929 | 10.954574 | 5.10478e-06 | 0.026815433 | -0.32 | -1.25 | 8.75824e-05 |
| ABCA2 | 8.226239 | 8.458478 | 5.05067e-06 | 0.026546305 | 0.23 | 1.17 | 8.74612e-05 |
| MCMDC2 | 6.532307 | 7.1937537 | 5.09112e-06 | 0.02674872 | 0.66 | 1.58 | 8.76175e-05 |
| LOC102556096 | 6.5520515 | 6.122532 | 5.14651e-06 | 0.027024336 | -0.43 | -1.35 | 8.77583e-05 |
| CLTA | 10.365583 | 10.588079 | 5.10917e-06 | 0.026833335 | 0.22 | 1.17 | 8.73887e-05 |
| --- | 9.346304 | 10.19054 | 5.18432e-06 | 0.027212484 | 0.84 | 1.80 | 8.78655e-05 |
| SNX1 | 8.261715 | 8.564158 | 5.18045e-06 | 0.027197381 | 0.30 | 1.23 | 8.80677e-05 |
| LRSAM1 | 7.4748216 | 7.943546 | 5.26844e-06 | 0.027643496 | 0.47 | 1.38 | 8.87517e-05 |
| MCM2 | 10.46542 | 10.073568 | 5.25564e-06 | 0.027581573 | -0.39 | -1.31 | 8.88043e-05 |
| --- | 10.817695 | 10.318726 | 5.36682e-06 | 0.028154343 | -0.50 | -1.41 | 9.01367e-05 |
| DHFR | 7.7850294 | 7.3539295 | 5.50541e-06 | 0.02887588 | -0.43 | -1.35 | 9.21867e-05 |
| MOCS2 | 8.502102 | 8.910984 | 5.54632e-06 | 0.02908488 | 0.41 | 1.33 | 9.25936e-05 |
| WNK3 | 6.2169447 | 5.833446 | 5.5534e-06 | 0.029116493 | -0.38 | -1.30 | 9.24352e-05 |
| NDUFAB1 | 12.545857 | 12.425858 | 5.55697e-06 | 0.029129632 | -0.12 | -1.09 | 9.22192e-05 |
| AKR1C14 | 7.6766896 | 8.716731 | 5.77866e-06 | 0.030285966 | 1.04 | 2.06 | 9.56137e-05 |
| MAPK8 | 10.16701 | 9.738748 | 5.78578e-06 | 0.030317461 | -0.43 | -1.35 | 9.54482e-05 |
| --- | 7.3292317 | 7.603502 | 5.8169e-06 | 0.030474743 | 0.27 | 1.21 | 9.56786e-05 |
| --- | 6.9480133 | 4.929379 | 5.82427e-06 | 0.030507524 | -2.02 | -4.05 | 9.5518e-05 |
| LOC100909889 | 9.291128 | 9.577254 | 5.96099e-06 | 0.0311998 | 0.29 | 1.22 | 9.66234e-05 |
| RERG | 9.993932 | 9.68336 | 5.83604e-06 | 0.030563358 | -0.31 | -1.24 | 9.54304e-05 |
| DCLK2 | 8.091287 | 7.6523476 | 5.91812e-06 | 0.030987289 | -0.44 | -1.36 | 9.64896e-05 |
| ECHS1 | 11.102609 | 10.723006 | 5.94387e-06 | 0.031116165 | -0.38 | -1.30 | 9.66269e-05 |
| MRPS21 | 12.398793 | 12.238851 | 6.17565e-06 | 0.032298643 | -0.16 | -1.12 | 9.89524e-05 |
| TMEM119 | 7.7968674 | 7.409388 | 6.06169e-06 | 0.031720802 | -0.39 | -1.31 | 9.79709e-05 |
| RAB3GAP2 | 8.565571 | 8.7176895 | 6.14572e-06 | 0.03214825 | 0.15 | 1.11 | 9.87565e-05 |
| MPP5 | 8.799564 | 8.460074 | 6.13537e-06 | 0.032100234 | -0.34 | -1.27 | 9.88752e-05 |
| GRTP1 // ADPRHL1 | 9.121112 | 9.887505 | 6.22588e-06 | 0.032555144 | 0.77 | 1.70 | 9.94714e-05 |
| NUMA1 | 8.326563 | 8.753159 | 6.29307e-06 | 0.032893885 | 0.43 | 1.34 | 9.9972e-05 |
| LOC691920 | 8.473736 | 8.897824 | 6.3033e-06 | 0.032941017 | 0.42 | 1.34 | 9.98499e-05 |
| MTHFD2 | 9.361395 | 8.805 | 6.33404e-06 | 0.033095382 | -0.56 | -1.47 | 0.000100053 |
| ZDHHC2 | 11.288554 | 11.040623 | 6.39972e-06 | 0.033432115 | -0.25 | -1.19 | 0.000100805 |
| STK39 | 10.830381 | 11.067614 | 6.28738e-06 | 0.032870416 | 0.24 | 1.18 | 0.000100167 |
| LAMA3 | 6.353171 | 6.6259003 | 6.44265e-06 | 0.033649944 | 0.27 | 1.21 | 0.000101195 |
| RUNDC1 | 8.592049 | 8.990171 | 6.48607e-06 | 0.033870243 | 0.40 | 1.32 | 0.000101591 |
| CTNNAL1 | 9.67745 | 9.205119 | 6.49706e-06 | 0.033921164 | -0.47 | -1.39 | 0.000101478 |
| ATP13A1 | 9.0576935 | 9.239849 | 6.51444e-06 | 0.034005377 | 0.18 | 1.13 | 0.000101465 |
| CTSD | 10.725613 | 11.050523 | 6.72205e-06 | 0.035055477 | 0.32 | 1.25 | 0.000103257 |
| RMI1 | 9.7633505 | 9.524961 | 6.67848e-06 | 0.034841653 | -0.24 | -1.18 | 0.000103156 |
| MYCBP | 9.800256 | 9.409725 | 6.59756e-06 | 0.034432653 | -0.39 | -1.31 | 0.000102473 |
| B3GALNT2 | 9.874722 | 9.561645 | 6.66715e-06 | 0.034789164 | -0.31 | -1.24 | 0.000103267 |
| TMA7 | 9.915775 | 9.613102 | 6.82495e-06 | 0.035571635 | -0.30 | -1.23 | 0.000103978 |
| SPTBN1 | 12.755796 | 12.876878 | 6.69951e-06 | 0.034944635 | 0.12 | 1.09 | 0.000103195 |
| CNTF | 7.8299513 | 8.091109 | 6.7485e-06 | 0.035179935 | 0.26 | 1.20 | 0.000103095 |
| MED12 | 10.110693 | 10.294352 | 6.72232e-06 | 0.03505016 | 0.18 | 1.14 | 0.000102977 |
| PACSIN2 | 11.3127165 | 11.001823 | 6.88756e-06 | 0.035884187 | -0.31 | -1.24 | 0.000104362 |
| GTF2A1 | 10.350855 | 10.110425 | 6.89958e-06 | 0.035939895 | -0.24 | -1.18 | 0.00010426 |
| ZC3H13 | 8.946105 | 9.319051 | 6.90636e-06 | 0.03596834 | 0.37 | 1.29 | 0.000104081 |
| PRKCB | 6.4168577 | 6.621119 | 6.8812e-06 | 0.035857957 | 0.20 | 1.15 | 0.000104549 |
| OBSCN | 11.478647 | 11.9389925 | 7.12951e-06 | 0.037123356 | 0.46 | 1.38 | 0.000107154 |
| FCNB | 7.7322593 | 8.062042 | 7.22123e-06 | 0.03758648 | 0.33 | 1.26 | 0.000107951 |
| --- | 7.6482983 | 7.266083 | 7.21293e-06 | 0.037550505 | -0.38 | -1.30 | 0.000108116 |
| SNX1 | 8.787007 | 9.103197 | 7.22709e-06 | 0.03760979 | 0.32 | 1.25 | 0.000107749 |
| CLCN1 | 6.985566 | 7.2458644 | 7.33259e-06 | 0.038122147 | 0.26 | 1.20 | 0.00010788 |
| ASNA1 | 11.516589 | 11.285266 | 7.27685e-06 | 0.03785415 | -0.23 | -1.17 | 0.000107914 |
| DST | 10.2210655 | 10.510596 | 7.26267e-06 | 0.037787654 | 0.29 | 1.22 | 0.000107991 |
| PREX2 | 8.738749 | 9.112734 | 7.31858e-06 | 0.0380566 | 0.37 | 1.30 | 0.000107959 |
| LOC102556724 | 5.1297026 | 5.440879 | 7.31771e-06 | 0.038059402 | 0.31 | 1.24 | 0.000108232 |
| AIG1 | 8.916167 | 8.634772 | 7.37829e-06 | 0.038352344 | -0.28 | -1.22 | 0.000108267 |
| SYNE2 | 8.986337 | 9.449789 | 7.44936e-06 | 0.03871432 | 0.46 | 1.38 | 0.000109023 |
| ATP7B | 7.346892 | 7.652193 | 7.46551e-06 | 0.03879081 | 0.31 | 1.24 | 0.000108973 |
| FAM84B | 6.586463 | 6.9728117 | 7.57949e-06 | 0.03937544 | 0.39 | 1.31 | 0.000110348 |
| OASL2 | 6.7115088 | 6.057865 | 7.59671e-06 | 0.039457314 | -0.65 | -1.57 | 0.000110311 |
| EEF1A2 | 11.602474 | 11.931812 | 7.71342e-06 | 0.040048096 | 0.33 | 1.26 | 0.000111425 |
| TRMT10C | 8.730776 | 8.292305 | 7.65611e-06 | 0.039758172 | -0.44 | -1.36 | 0.000110884 |
| SLC11A1 | 7.1023316 | 6.4565306 | 7.71723e-06 | 0.040052406 | -0.65 | -1.56 | 0.000110905 |
| --- | 4.18563 | 4.7282224 | 7.71527e-06 | 0.040049978 | 0.54 | 1.46 | 0.000111164 |
| CACNA1H | 10.404476 | 10.836242 | 7.7303e-06 | 0.0401125 | 0.43 | 1.35 | 0.000110808 |
| --- | 6.324403 | 6.970513 | 7.78919e-06 | 0.040402513 | 0.65 | 1.56 | 0.000111081 |
| --- | 6.9279666 | 6.4173403 | 7.7906e-06 | 0.040402066 | -0.51 | -1.42 | 0.000110817 |
| CDK10 | 8.623921 | 8.8797035 | 7.78904e-06 | 0.04040956 | 0.26 | 1.19 | 0.000111363 |
| --- | 9.206145 | 9.605152 | 7.86333e-06 | 0.040771373 | 0.40 | 1.32 | 0.000111567 |
| IFNAR1 | 10.00901 | 10.310453 | 7.93887e-06 | 0.04115512 | 0.30 | 1.23 | 0.000112353 |
| LOC687130 | 6.267531 | 6.7928567 | 8.02586e-06 | 0.04158999 | 0.53 | 1.44 | 0.000113011 |
| PAXBP1 | 9.988528 | 10.53877 | 8.02875e-06 | 0.041596975 | 0.55 | 1.46 | 0.000112767 |
| MSL1 | 9.604616 | 9.856311 | 8.15688e-06 | 0.042236313 | 0.25 | 1.19 | 0.000113707 |
| MYOM2 | 10.76655 | 10.299195 | 8.09928e-06 | 0.041946176 | -0.47 | -1.38 | 0.000113187 |
| HIP1R | 7.752314 | 8.136673 | 8.09052e-06 | 0.041908886 | 0.38 | 1.31 | 0.000113349 |
| TNIK | 9.265636 | 9.555519 | 8.01028e-06 | 0.041517273 | 0.29 | 1.22 | 0.000113077 |
| ST3GAL3 | 9.297163 | 9.105125 | 8.23373e-06 | 0.04260954 | -0.19 | -1.14 | 0.000113924 |
| PDE8B | 5.9709115 | 6.3113065 | 8.18385e-06 | 0.0423596 | 0.34 | 1.27 | 0.000113515 |
| MED6 | 9.294383 | 9.003771 | 8.16296e-06 | 0.04225965 | -0.29 | -1.22 | 0.000113508 |
| CHEK1 | 9.077647 | 8.416396 | 8.24477e-06 | 0.04265846 | -0.66 | -1.58 | 0.000113794 |
| ALDH1A7 | 5.1550493 | 4.5415287 | 8.24699e-06 | 0.042661678 | -0.61 | -1.53 | 0.000113544 |
| LOC100362798 | 6.7219863 | 6.473007 | 8.3316e-06 | 0.04309106 | -0.25 | -1.19 | 0.000114426 |
| --- | 10.284097 | 9.959633 | 8.46461e-06 | 0.043762054 | -0.32 | -1.25 | 0.000115683 |
| SON | 11.082533 | 11.346085 | 8.35318e-06 | 0.043194313 | 0.26 | 1.20 | 0.000114441 |
| PRELP | 9.24967 | 8.702537 | 8.55087e-06 | 0.044190902 | -0.55 | -1.46 | 0.000116292 |
| NRBP2 | 10.106756 | 10.476383 | 8.54659e-06 | 0.044177316 | 0.37 | 1.29 | 0.000116518 |
| EPHB2 | 7.7312303 | 7.311424 | 8.59802e-06 | 0.044425957 | -0.42 | -1.34 | 0.000116649 |
| FZD5 | 9.225944 | 8.903835 | 8.69584e-06 | 0.044922724 | -0.32 | -1.25 | 0.000117689 |
| MMP23 | 8.401291 | 8.033065 | 8.76612e-06 | 0.045276996 | -0.37 | -1.29 | 0.000118353 |
| PHLDA3 | 9.956608 | 9.496613 | 8.80617e-06 | 0.045475077 | -0.46 | -1.38 | 0.000118607 |
| MAGI1 | 10.002075 | 10.353765 | 8.90442e-06 | 0.045955736 | 0.35 | 1.28 | 0.000119067 |
| OBSCN | 9.210903 | 9.690162 | 8.87382e-06 | 0.045815554 | 0.48 | 1.39 | 0.00011923 |
| --- | 7.6702113 | 8.415946 | 8.89903e-06 | 0.0459368 | 0.75 | 1.68 | 0.000119281 |
| ZIM1 | 8.6977215 | 8.317209 | 8.96431e-06 | 0.04625584 | -0.38 | -1.30 | 0.000119581 |
| GNA13 | 9.518843 | 9.144538 | 9.01133e-06 | 0.046489473 | -0.37 | -1.30 | 0.000119922 |
| LIPE | 9.612112 | 9.981039 | 9.23006e-06 | 0.04760867 | 0.37 | 1.29 | 0.00012254 |
| TPMT | 9.968546 | 9.547865 | 9.29439e-06 | 0.04793117 | -0.42 | -1.34 | 0.000123101 |
| --- | 6.9619646 | 6.199332 | 9.31056e-06 | 0.048005216 | -0.76 | -1.70 | 0.000123023 |
| TMEM234 | 7.8900356 | 7.4071064 | 9.39058e-06 | 0.048399054 | -0.48 | -1.40 | 0.000123495 |
| SNRNP200 | 10.114379 | 10.292011 | 9.36763e-06 | 0.048290107 | 0.18 | 1.13 | 0.000123484 |
| SNRK | 9.665357 | 10.199335 | 9.45455e-06 | 0.04870986 | 0.53 | 1.45 | 0.000123753 |
| FAM160B1 | 8.523586 | 8.761865 | 9.43873e-06 | 0.048637778 | 0.24 | 1.18 | 0.000123836 |
| MTMR9 | 9.742512 | 9.496649 | 9.68937e-06 | 0.049909946 | -0.25 | -1.19 | 0.000126529 |
