# Supplementary material for: MicroRNA mediated regulation in early-onset cardiac hypertrophy: Insights from the hypertrophic heart rat model
Source: PLoS One. 2025 Dec 30;20(12):e0338909. doi: 10.1371/journal.pone.0338909 (PMC12752997; doi:10.1371/journal.pone.0338909)
Supplement: Attachment — Submitted filename: Response to Reviewers.docx [file pone.0338909.s002.docx]

Rebuttal Letter

Re: PONE-D-25-28567

# **Title:** MicroRNA-mediated regulation in early-onset cardiac hypertrophy: insights from the hypertrophic heart rat model

Corresponding author: Shahzad Sadiq

Dear Dr. Ai,

Thank you for the opportunity to revise our manuscript. We appreciate the constructive comments from you and the reviewer. In response, we have refined the manuscript’s framing, integrated the suggested literature with precise citations, standardised statistical reporting, and clarified gene-level interpretations—particularly for *GANC*. A **Changes Table** summarising section-by-section edits is included with this submission.

## 1) Framing and contribution

We recast the contribution as **model-specific**, defining miRNA–mRNA signalling in neonatal, normotensive HHRs, and removed/tempered “first/novel” phrasing in the Abstract, Introduction and Discussion.

## 2) Literature integration (per reviewer guidance; PMIDs shown)

1. **miR-34a.** We expanded the background and Discussion to integrate three key sources and to calibrate claims about therapeutic modulation and mechanism:

- **Review (PMID 35155600):** Summarises miR-34a’s roles in maladaptive remodelling/fibrosis and apoptosis; details repression of *SIRT1, ALDH2, BCL2, PNUTS* and links to genome-stability/telomere maintenance pathways. We used this to clarify mechanism and scope.
- **Post-MI study (PMID 26082557):** Shows miR-34a upregulation in injured myocardium and that anti-miR-34a improves LV function and reduces fibrosis and cardiomyocyte death after myocardial infarction, supporting a causal role in adverse remodelling.
- **Diabetic cardiomyopathy study (PMID 36231079):** Demonstrates that in established type 1 diabetic cardiomyopathy, anti-miR-34a confers only modest benefit, underscoring that efficacy varies by disease setting and stage.
- **Manuscript changes:** text revised in the miR-34a section of the Discussion to add these citations, articulate the model-/stage-dependence of therapeutic effects, and retain a conservative, context-specific framing of our neonatal HHR findings (no novelty claims).

2. **miR-351.** We incorporated evidence that miR-351, in concert with the long non-coding RNA LncSync, regulates cardiomyocyte homeostasis and hypertrophic growth in mice (**PMID 36929003**). This study provides developmental and hypertrophy-relevant context for miR-351 regulation, reinforcing its plausibility in our neonatal model.

- **Manuscript changes:** Expanded the miR-351 paragraph in the Discussion to cite **36929003** and to state that miR-351 participates in hypertrophic responses in experimental systems, with LncSync coordination in murine hearts.

3. **miR-490***. We retained our cardiac statement that miR-490 constrains phenylephrine/RAS-driven hypertrophy via c-FOS/AP-1 (existing refs) and added non-cardiac mechanistic data showing that miR-490* represses telomere-maintenance genes (TERF2, TNKS2, SMG1), inducing telomere dysfunction and DNA-damage signalling (**PMID 32970185**). This broadens biological plausibility for miR-490 family involvement in growth-control programs.
   - **Manuscript changes:** Added **32970185** and a one-sentence summary of the telomere-programme mechanism to the miR-490* section in discussion.
4. **HTR2A (5-HT2A).** We integrated mechanistic and human-genetic context: receptor activation engages PI3K–PDK1–AKT–mTOR signalling in an isoproterenol-induced hypertrophy model (PMID 32519137), and a large community-based study reported associations between HTR2A variants (including rs6313/T102C) and hypertension risk, with inter-cohort heterogeneity in effect direction (**PMID 31906879**).

- **Manuscript changes:** In the HTR2A subsection of the Discussion, added **32519137** to support the pathway statement and **31906879** to anchor human relevance, noting the cohort-to-cohort variability; retained our conservative interpretation of *HTR2A* upregulation and miR-34a regulation in our system (no priority claims).

5. **ITGA7.** We added translational evidence that ITGA7 loss of function is associated with adult-onset cardiomyopathy in humans and that Itga7⁻/⁻ mice recapitulate conduction delays and systolic dysfunction, consistent with impaired α7β1-integrin mechanotransduction (**PMID 36444867**).

- **Manuscript changes:** In the ITGA7 subsection, cited **36444867** and added a concise sentence linking our observed downregulation to mechanotransduction pathways and the human–murine phenotype, underscoring clinical relevance.

## 3) Results and statistics (precision & transparency)

- **Exact p-values.** Standardised to four decimals for all *p* ≥ 0.001 and defined star thresholds; updated throughout text and tables. (Example: Table 4 now shows GANC p = 0.0492.)
- **GANC cross-platform note.** Results now include a brief, non-interpretive statement flagging directional discordance (microarray ↑ vs RT-qPCR ↓) with a pointer to the Discussion comparison (classification as nominal/cautious).

## 4) Gene-level interpretation (selected examples)

- **HTR2A.** Increased in neonatal HHR hearts; cell experiments show miR-34a regulation of *HTR2A*. We integrate ISO-induced and pressure-overload contexts (mechanistic cascade and TAB model) and human cohort data, avoiding priority claims.
- **SGPP1.** Framed as consistent with increased sphingolipid metabolism/apoptosis in early remodelling.
- **ITGA7.** Downregulation aligned with perturbed mechano-transduction; added translational link via human–mouse genetics (PMID **36444867**).
- **GANC.** Small-effect, borderline decrease by RT-qPCR (*p* = 0.0492) opposite to array estimate; limited human-genetic support and cross-platform mismatch → interpreted cautiously, with protein-level and functional validation deferred to future work.

## 5) Figures, tables, and nomenclature

- **Figure 3 caption.** Now reports star thresholds.  Please note: Caption for Fig 3 was in the first manuscript (line: 208)
- **Figure 4 caption.** Please note: Caption for Fig 3 was in the first manuscript (line: 270)
- **Global style fixes.** Corrected RT-PCR → RT-qPCR, H9C2 → H9c2(2-1), unified star notation, corrected a miRNA table entry (rno-miR-490* *p*=0.0327), previously 0.327), and standardised gene/protein formatting and abbreviations at first use.

**6) Data transparency and scope**

**Feasibility.** We did not add new wet-lab experiments because we currently lack lab access, animals/samples, and funding; additional assays would require new approvals, reagents, and time beyond the revision window. Instead, to address the request for more gene-expression/genetic evidence while avoiding bias, we strengthened transparency and reporting as follows:

- **Complete Supporting Information provided.** We now include full differential-expression outputs as S1A Data — miRNA differential expression (HHR vs NHR) and S1B Data — mRNA differential expression (HHR vs NHR). Each table identifies the platform and normalization method, reports raw p-values and Benjamini–Hochberg FDR, and gives fold change (HHR/NHR; >1 = up in HHR). S1A also includes a nomenclature note mapping legacy “miR-490*” → miR-490-3p.
- **Statistical transparency.** We standardised exact *p*-values (for *p* ≥ 0.001) across text and tables, defined star thresholds once, and kept fold-change precision conservative. The GANC result is reported with exact statistics (p = 0.0492) and cross-referenced for array vs RT–qPCR directionality.
- **Literature context and limitations.** We integrated the key mechanistic and human-genetic papers highlighted by the reviewer, clarified limitations (including the small-effect/discordant GANC signal), and outlined feasible next steps (protein-level assays, functional perturbation, evaluation in human cardiomyopathy datasets).
- **Bias control.** We **did not** perform ad-hoc re-analysis of external RNA-seq datasets to avoid selective or inconsistently processed comparisons; instead, we prioritised complete reporting of our primary data in S1A/S1B.

We believe these revisions address the editorial and reviewer concerns and materially improve clarity, rigour, and transparency, consistent with PLOS ONE’s criteria. We appreciate your consideration.

Sincerely,

Shahzad Sadiq (on behalf of all authors)

| ****Section**** | ****Line**** | ****Change**** |
| --- | --- | --- |
| Novelty | | |
| Abstract | 60 | These findings provide insight into the early molecular drivers of cardiac hypertrophy in neonatal HHR and delineate miRNA–mRNA relationships involved in remodelling. |
| Introduction | 147 | The HHR is a genetically driven model that enables investigation of miRNA expression in primary cardiac hypertrophy without the confounding effects of hypertension. This study profiles miRNAs in neonatal HHRs and validates miRNA–mRNA interactions previously implicated in hypertrophic remodelling. The findings provide further insights into miRNA-mediated regulation and established hypertrophic pathways, highlighting potential therapeutic targets for pathological cardiac hypertrophy. |
| Discussion | 558 | This study characterises differential expression of miR-34a, miR-351, and miR-490 in neonatal HHRs, defining a distinct miRNA signature and associated pathways. The findings generalise established hypertrophic mechanisms to a normotensive, genetically predisposed model at an early developmental stage. |
| Discussion- HTR2A | 765 | Extending prior reports implicating HTR2A in hypertrophy, this study demonstrates upregulation of HTR2A in 2-day-old HHR hearts and identifies the gene as a regulatory target of miR-34a. |
| Discussion - SGPP1 | 793 | This study reports SGPP1 upregulation in 2-day-old HHRs, which may drive apoptosis and reduce cardiomyocyte proliferation during foetal development. |
| GANC | | |
| Abstract | 57 | Additionally, elevated SGPP1 expression suggested increased sphingolipid metabolism contributing to apoptosis, while ITGA7 was reduced and GANC showed a modest decrease, suggesting dysregulation in mechano-signal transduction and glycogen metabolism. These findings provide insight into the early molecular drivers of cardiac hypertrophy in neonatal HHR and delineate miRNA–mRNA relationships implicated in remodelling. |
| Results | 458 | In contrast, Integrin Subunit Alpha 7 (ITGA7) was downregulated (fold change −1.80; p < 0.01). For Glucosidase Alpha, Neutral C (GANC), RT-PCR indicated a modest reduction (fold change −1.47; p = 0.0492), whereas the microarray estimated an increase (fold change +1.45); see Discussion for cross-platform comparison. Given the small effect size and cross-platform discordance, expression level of GANC was considered a nominal finding and interpreted cautiously. |
| Results |  | P-values mentioned in text and Table 4. P-values updated to 4-decimal points |
| Discussion- GANC | 799 | GANC belongs to glycoside hydrolase family 31 and encodes neutral α-glucosidase C, an enzyme implicated in glycogen metabolism [59]. The gene remains understudied; functional annotations in Rattus norvegicus are largely computational predictions; alpha-1,4-glucosidase, broader hydrolase activity and roles in carbohydrate metabolism; (National Center for Biotechnology Information, U.S. National Library of Medicine). During cardiac hypertrophy, myocardial metabolism shifts from fatty-acid oxidation to glycolysis, resembling the foetal metabolic state [8] . The potential role of neutral α-glucosidase C in these altered states remains unexplored.  Bioinformatic predictions suggest that GANC may be targeted by miR-351 and miR-490. However, RT-PCR after transfection of H9c2-1 cells yielded inconclusive changes in GANC expression. In neonatal HHR hearts, RT-PCR indicated a modest reduction in GANC, whereas microarray estimated an increase, indicating directional discordance. Together with the lack of supportive human genetic associations, these observations do not support a firm conclusion about GANC dysregulation in neonatal HHRs and should be interpreted with caution until confirmed by protein-level assays and functional validation studies. |
| Future Directions | 924 | Evidence for GANC dysregulation remains uncertain in neonatal HHRs. It should be considered for protein-level confirmation and functional validation, with parallel evaluation in human cardiomyopathy datasets. |
| Gene-expression data / genetic evidence | | |
| Future Directions | 919 | Validation by RT–PCR was limited to a subset of microRNAs and genes because of resource constraints, with additional microarray-identified candidates deferred for future work. In place of external RNA-seq reanalysis, full gene-level results are provided in the Supplementary Data to ensure transparency and enable re-use. |
| Acknowledging established roles of miRs/Genes using suggested references | | |
| Discussion | 573 | **miR-34a:**  miR-34a is consistently implicated in maladaptive ventricular remodelling, hypertrophy and fibrosis, acting through anti-apoptotic and stress-response pathways that include direct repression of Sirtuin 1 (SIRT1), Aldehyde Dehydrogenase 2 (ALDH2), B-cell CLL/Lymphoma 2 (BCL2), and Phosphatase 1 Nuclear Targeting Subunit (PNUTS), and with links to telomere biology and ageing [13,26–28]. Therapeutic inhibition of the miR-34 family can attenuate remodelling, though efficacy is variable across models, with only modest benefit reported in established type 1 diabetic cardiomyopathy [26,29]. Moreover, following myocardial infarction, miR-34a expression increases in the injured myocardium, and its inhibition improves ventricular function, reduces fibrosis and cell death, and enhances myocardial repair [30]. |
| Discussion | 583 | miR-351  miR-351 is linked to hypertrophic responses in experimental models. In mice, the long non-coding RNA LncSync coordinates with miR-351 to regulate cardiomyocyte homeostasis and hypertrophic growth [31]. In phenylephrine-stimulated cardiomyocytes, miR-351 targets E2F Transcription Factor 3 (E2F3), increasing proliferation and apoptosis resistance and thereby facilitating hypertrophic growth [32,33] |
| Discussion | 588 | miR-490*  miR-490 inhibits cellular proliferation by targeting c-FOS, which is upregulated during phenylephrine-induced hypertrophy via the RAS pathway [25,34]. miR-490* constrains proliferative capacity in glioblastoma by repressing telomere-maintenance genes Telomeric Repeat-binding Factor 2 (TERF2), Tankyrase 2 (TNKS2) and Serine/Threonine-protein kinase, (SMG1), inducing telomere dysfunction and DNA-damage signalling [35] . |
| Discussion | 735 | HTR2A  Accumulating evidence indicates that HTR2A engages pro-hypertrophic signalling via the PI3K–PDK1–AKT–mTOR pathway in an isoproterenol (ISO)–induced mouse model of cardiac hypertrophy [56] |
| Discussion | 760 | HTR2A  A more recent study also reported associations between several HTR2A single nucleotide polymorphisms (SNPs), including rs6313/T102C, and hypertension risk, though the direction and magnitude of effect varied between cohorts [61]. |
| Discussion | 859 | ITGA7  Human–murine evidence implicates ITGA7 loss of function in adult-onset cardiomyopathy; Itga7⁻/⁻ mice recapitulate conduction delays and systolic dysfunction, consistent with impaired α7β1-integrin mechano-transduction [70]. |
| Others | | |
| Results | 286 | P-Values added  Expression of established biomarkers of cardiac hypertrophy were quantified using RT-qPCR. NPPA (p=0.0218), NPPB (p=0.0004) and Mitogen-Activated Protein Kinase 1 (MAPK1) (p=0.0005) levels were significantly elevated in HHRs compared to controls. |
| General |  | P-values updated throughout the manuscript to reflect actual values upto 4 decimal places. |
| Figures- ALL |  | Asterisk in figure captions updated to reflect (*) p<0.05, (**) p<0.01, (***) p<0.001 where applicable. |
| Table 2 |  | p-value of rno-miR-490* corrected to 0.0327, entered previously as 0.327 |
| General |  | RT-PCR corrected to RT-qPCR |
| General |  | H9C2 correct to H9C2(2-1) |
